# Nickel-catalyzed enantioselective 1,2-vinylboration of styrenes†

**DOI:** 10.1039/d1sc04071e

**Published:** 2021-09-07

**Authors:** Yang Ye, Jiandong Liu, Bing Xu, Songwei Jiang, Renren Bai, Shijun Li, Tian Xie, Xiang-Yang Ye

**Affiliations:** School of Pharmacy, Hangzhou Normal University Hangzhou Zhejiang 311121 PR China yeyang0711@163.com xbs@hznu.edu.cn xyye@hznu.edu.cn; Key Laboratory of Elemene Class Anti-Cancer Chinese Medicines, Engineering Laboratory of Development and Application of Traditional Chinese Medicines, Collaborative Innovation Center of Traditional Chinese Medicines of Zhejiang Province, Hangzhou Normal University Hangzhou Zhejiang 311121 PR China; Center for Supramolecular Chemistry and Catalysis, Department of Chemistry, Shanghai University Shanghai 200444 PR China; College of Material, Chemistry and Chemical Engineering, Hangzhou Normal University Hangzhou Zhejiang 311121 PR China

## Abstract

A novel nickel-catalyzed asymmetric 1,2-vinylboration reaction has been developed to afford benzylic alkenylboration products with high yields and excellent enantioselectivities by using a chiral bisoxazoline ligand. Under optimized conditions, a wide variety of chiral 2-boryl-1,1-arylvinylalkanes are efficiently prepared from readily available olefins and vinyl halides in the presence of bis(pinacolato)diboron as the boron source in a mild and easy-to-operate manner. This three-component cascade protocol furnishes exceptional chemo- and stereoselectivity, and its usefulness is illustrated by its application in asymmetric modifications of several structurally complex natural products and pharmaceuticals.

## Abstract 2

A novel nickel-catalyzed asymmetric 1,2-vinylboration reaction has been developed to afford benzylic alkenylboration products with high yields and excellent enantioselectivities by using a chiral bisoxazoline ligand.

## Graphical abstract

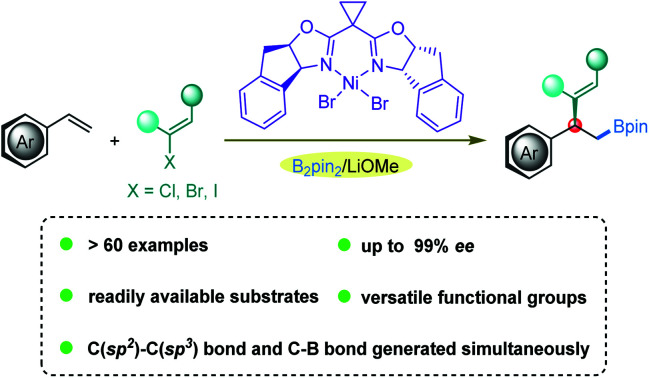

## Introduction

**Fig. 1:**
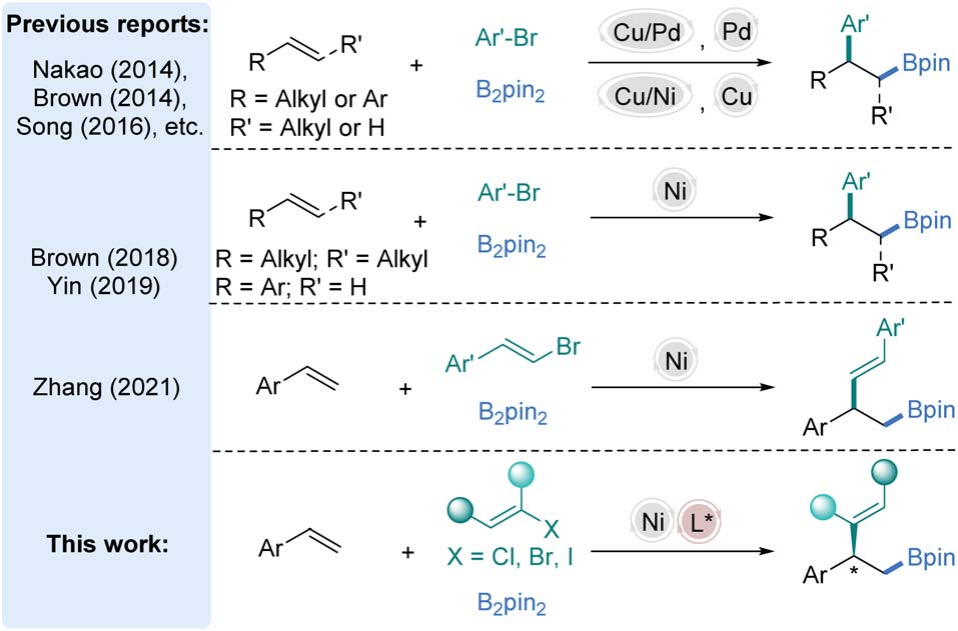
Transition-metal catalyzed carboboration of alkenes.

Difunctionalization of alkenes through a transition-metal catalyzed process has gradually evolved as a significant opportunity for establishing two newly formed stereocenters in a single step, thus allowing rapid access to molecular complexity.^1^ Of these methods, the carboboration of alkene precursors is emerging as a versatile and efficient approach to prepare aliphatic boron intermediates.^2^ Due to the excellent accessibility of diverse functional groups from the carbon–boron bond, this method provides researchers compelling approaches to obtain boron-containing intermediates and substantial opportunities for downstream diversifications.^3^ In the past few decades, many efforts have been devoted to the study of several classes of alkene carboboration reactions,^4^ in which the reactions vary based on the electrophiles and catalysts employed,^5^ especially in the 1,2-arylboration of vinylarenes by an elegant dual metal synergistic catalysis.^6–8^ For incorporation of aryl groups, Cu/Pd-,^7^ Cu/Ni-,^8^ Pd-,^9^ or Cu-catalysis^10^ has been proven to be effective (Fig. 1, top). Recently, stimulated by the special reactivity of nickel,^11^ the transient alkyl-Ni species generated from olefin insertion into a Ni–B bond have also been investigated by Brown^12^ and Yin,^13^ affording racemic 1,2-arylboration examples (Fig. 1, middle). The majority of these methods utilize activated alkenes (*e.g.*, alkenyl arenes, vinyl-silanes, and strained alkenes) to increase the rate of an insertion event prior to the direct reaction between the electrophiles and nucleophiles (*e.g.*, B_2_pin_2_ or [M]-Bpin). In the nickel-catalyzed cross-coupling of C(sp^2^)-electrophiles, the formation of stable C(sp^2^)-Ni intermediates by oxidative addition of the electrophiles to low-stage Ni is generally involved.^14^ Thus, special alkyl benzylic substrates are competent for vinylation with vinyl halides, possibly due to matched reactivities.

The nickel-catalyzed vinylboration of alkenylarenes is worth being developed. Recently, Zhang^15*f*^ reported a nickel-catalyzed alkenylboration of alkenylarenes and vinyl bromides to obtain the racemic 2-boryl-1,1-arylvinylalkanes (Fig. 1, middle). We reasonably questioned whether the general strategy described above could be expanded to asymmetric vinylation when a classic cross-coupling partner, a vinyl halide,^14,15^ is used. Furthermore, carboboration of asymmetric alkenes is infrequent and the enantioselectivity and chemoselectivity of different classes of olefins are rarely discussed in a single system concurrently. From this perspective we explored a process for enantioselective 1,2-vinylboration of alkenes catalyzed by a chiral Ni-complex with intriguing results (Fig. 1, bottom).^11*a*,16–19^ More specifically, these reactions are highly chemo- and stereoselective, operated with near equimolar quantities of the alkenes and vinyl halides, and occur at mild temperature. Structurally complex substrates derived from natural products and approved drugs are well tolerated. The preliminary mechanistic studies are also discussed.

## Results and discussion

### Optimization of the reaction conditions

We initiated our investigation by exploring the enantioselective vinylboration of styrene (**1**) with (*E*)-(2-bromovinyl)benzene (**2**) and bis(pinacolato)diboron (B_2_pin_2_) as model substrates using chiral bis(oxazoline) ligands (Table 1). An extensive screening of the reaction parameters revealed that the use of NiBr_2_·DME (10 mol%), **L1** (10 mol%), and B_2_pin_2_ (1.2 equiv.) as the boron source and LiOMe (1.5 equiv.) as the base in 1,4-dioxane at 10 °C delivered compound **3** in 85% yield and 94% ee (entry 1). Under the selected conditions, ligands appeared to have great influence on this asymmetric transformation, while replacing **L1** with other chiral dibox ligands **L2–L5** did not result in better outcomes in both yields and ees (entries 2–5). With other ligands such as 2,6-bis((*S*)-4-isopropyl-4,5-dihydrooxazol-2-yl)pyridine (iPr-Box), less desired vinylation products were produced (Table S1,† entries 8–12). Replacement of LiOMe by LiO*t*Bu led to a decrease in yield and ee (entry 6). THF was shown to be an unsuitable solvent (entry 7). Reaction at 0 °C (entry 8) led to a comparable yield and ee, while somewhat lower yield and ee were obtained at 25 °C (entry 9). The addition of a pre-coordinated **L1**·NiBr_2_ complex (10 mol%) as the catalyst to replace NiBr_2_·DME (10 mol%) and **L1** (10 mol%) led to evidently higher yield and ee (entry 10). Change of other parameters such as nickel sources led to inferior yields and enantioselectivities (Table S1,† entries 4–7).

**Table 1:** Optimization for the formation of **3**

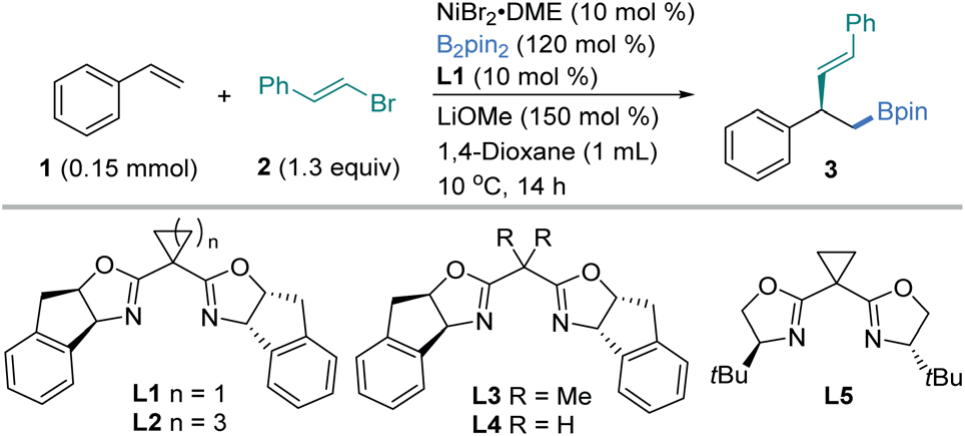			
Entry	Variation from standard conditions^a^	Yield^b^ [%]	ee^c^ [%]
1	None	85	94
2	**L2** instead of **L1**	54	81
3	**L3** instead of **L1**	34	48
4	**L4** instead of **L1**	26	17
5	**L5** instead of **L1**	20	73
6	LiO*t*Bu instead of LiOMe	50	85
7	THF instead of 1,4-dioxane	38	89
8	0 °C	80	93
9	25 °C	53	80
10	**L1**·NiBr_2_	92 (93)	96

a Standard conditions: **1** (0.150 mmol, 1.0 equiv.), **2** (0.195 mmol, 1.3 equiv.), B_2_pin_2_ (0.180 mmol, 1.2 equiv.), NiBr_2_·DME (10 mol%), **L1** (10 mol%), LiOMe (0.225 mmol, 1.5 equiv.), 1,4-dioxane (1.0 mL), 10 °C, 14 h.

b Yields determined by crude ^1^H NMR using 2,5-dimethylfuran as the internal standard. The yield in parentheses is the isolated yield.

c The ee values were determined by HPLC on a chiral stationary phase.

Enantiomerically pure chiral ligands were used to explore the nickel-catalyzed vinylboration listed in Tables 1 and S1.† The ligand effects appear to be pivotally important to the yields and ees. Consequently, we tentatively speculate that **L1**, **L2** and **L5** adopting a rigid cycloalkyl linker features a larger bond angle of the C(oxazoline)–C(methylene)–C(oxazoline) bond than those in **L3**, **L4** (entries 3 and 4) and **L8** (Table S1,† entry 10), which appears to have a crucial effect on the enantioselectivity. Further comparison of the results arising from **L1**, **L2** and **L5** (entries 1, 2 and 5) provides a reasonable conclusion that the rigid tricyclic fused rings in **L1** offer an optimal steric and rigidity requirement for enantioselective control. Systematic evaluation of various chiral ligands showed that both high enantiomeric excess and yields were obtained with a chiral nickel-bis(oxazoline) **L1**·NiBr_2_ (ref. 15*a* and 15*b*) for a wide range of vinyl halides with styrenes.^20^

### Substrate scope

**Fig. 2:**
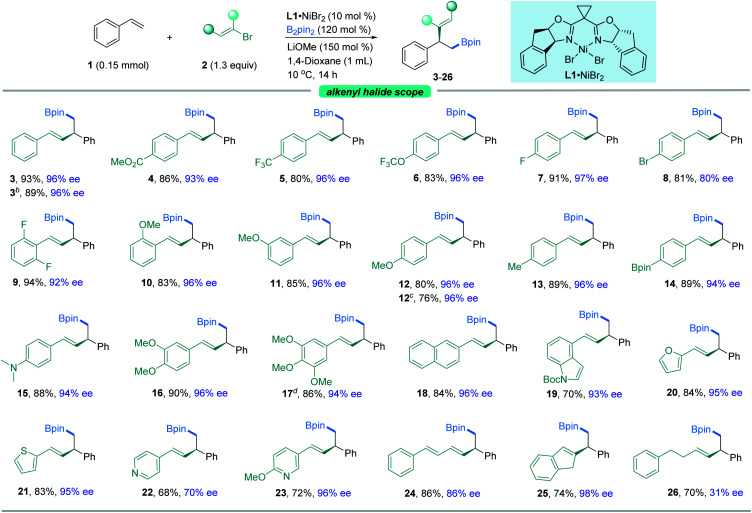
The scope of alkenyl halides. ^*a*^The standard reaction conditions; isolated yields are provided (average of 2 independent runs); ee is determined by chiral HPLC analysis. ^*b*^(*E*)-(2-Chlorovinyl)benzene instead of **2**. ^*c*^(*Z*)-1-(2-Bromovinyl)-4-methoxybenzene instead of **2**. ^*d*^(*E*)-5-(2-Iodovinyl)-1,2,3-trimethoxybenzene instead of **2**.

With the optimal reaction conditions in hand, we sought to explore the generality of this three-component reaction. Firstly, a wide range of β-aryl-substituted (*E*)-alkenyl bromides bearing electron-poor (**4–9**) or electron-rich substituents (**10–17**) on the arene afforded the desired product smoothly (Fig. 2). The electronic properties of alkenyl halides did not show an obvious effect on the efficiency of this transformation. Emphatically, (*E*)-alkenyl chloride (**3b**), (*E*)-alkenyl iodide (**17**) and (*Z*)-alkenyl bromide (**12b**) were shown to participate in the reaction to provide satisfactory results. In addition, β-naphthyl-substituted (*E*)-alkenyl bromide (**18**) was also proved to be compatible. Good coupling results with excellent enantioselectivities were also observed for β-heteroaromatic-substituted (*E*)-alkenyl bromides containing various functional groups or moieties such as indole (**19**), furan (**20**), thiophene (**21**) or pyridine (**22**, **23**). However, a slight decrease in ee was observed when 1,3-dienyl bromide (**24**) was used as the substrate. Notably, α-alkyl substituted alkenyl bromides, such as 2-bromoindene (**25**), are also competent coupling partners, while β-alkyl-substituted (*E*)-alkenyl bromide (**26**) is an active substrate but with reduced ee. Nonetheless, aryl bromides such as methyl 4-bromobenzoate are not suitable in this asymmetric system for lack of reactivity.

**Fig. 3:**
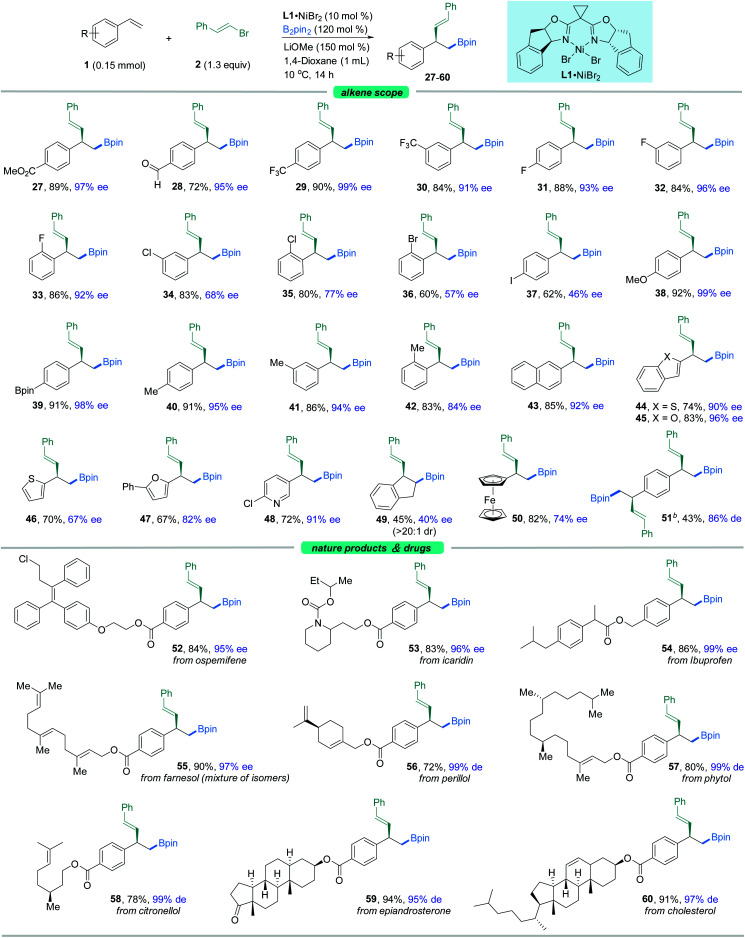
The scope of alkenes. ^*a*^The standard reaction conditions; isolated yields are provided (average of 2 independent runs); ee is determined by chiral HPLC analysis. ^*b*^1,4-Divinylbenzene instead of styrene, 2.6 equiv. of **2** and 2.4 equiv. of B_2_pin_2_ were used.

Our attention then shifted to the scope of the alkene partner. As shown in Fig. 3, styrenes bearing a variety of substituents on the aromatic ring, both electron-deficient ones such as esters, aldehydes, trifluoromethyl moieties and halides (**27–37**) and electron-rich ones (**38–42**), underwent this vinylboration smoothly with high levels of enantiocontrol. Signally, this asymmetric catalyst system couples an alkenyl bromide in the presence of an aryl halide (F, Cl, Br, or I) with moderate to high selectivity (**31–37**). Notably, the existence of the fluorine group gave excellent ee compared to its Cl^−^, Br^−^, and I^−^ counterparts. Under these exceptionally mild reaction conditions, even a sensitive functional group like a boronic acid pinacol ester (**39**) remained intact. It was found that *ortho*-substituted styrenes (**33**, **35**, **36**, **42**) were also suitable substrates but with slightly lower ees. Moreover, the naphthyl-containing olefin derived product (**43**) could be prepared by this method as well in good enantioselectivity. Besides, heterocycles such as benzothiophene (**44**), benzofuran (**45**), thiophene (**46**), furan (**47**), and pyridine (**48**) were also competent coupling partners. An internal alkene, such as indene (**49**), could also give rise to the *cis*-product in moderate yield with lower ee. Alkenylferrocene (**50**) could also undergo the reaction, providing access to good enantio-enriched ferrocene derivatives. Interestingly, substrates containing two symmetric vinyl groups such as 1,4-divinylbenzene could still make the transformation feasible to afford the double vinylboration product in good yield and ee (**51**). In this case, two chiral centers are established simultaneously in an efficient manner.

To further expand the usefulness of this tactic, we attached the styrene moiety to a variety of more structurally complex drugs and natural products. Remarkably, all of these substrates examined in our work underwent the transformation in excellent yields and enantioselectivities (**52–60**). The reaction system is capable of distinguishing the activated olefin over the unactivated olefin, providing the site specific transformation and enantiocontrol. As shown in Fig. 3, the alkenyl derivatives from pharmaceuticals and repellents such as ospemifene (**52**), icaridin (**53**), and ibuprofen (**54**) were amenable to this reaction, demonstrating the viability of this nickel-catalyzed asymmetric 1,2-vinylboration for modifications of bioactive molecules. Derivatives from naturally occurring alcohols such as farnesol (**55**), perillol (**56**), phytol (**57**), citronellol (**58**), epiandrosterone (**59**), and cholesterol (**60**) were also competent substrates, enabling access to the desired adducts in synthetically useful yields and ees. It is worth noting that many of these substrates exhibit extremely high enantioselectivity, up to 99% ee.

### Mechanistic consideration

**Fig. 4:**
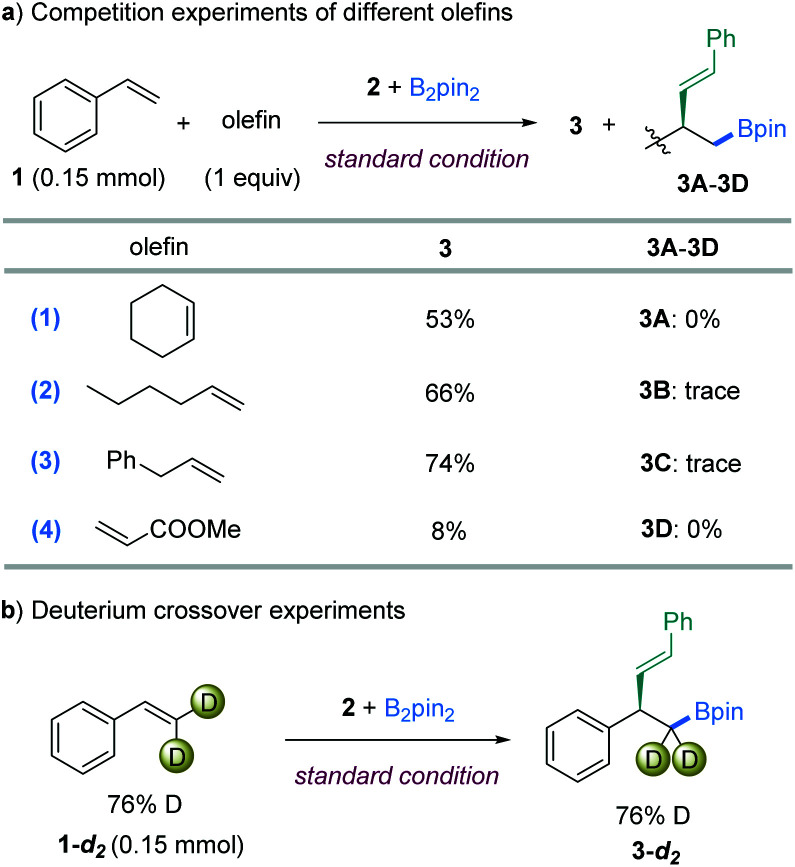
Competition and deuterium experiments.

Several competition experiments were conducted to compare the relative reactivities of different types of C=C bond (Fig. 4a). The vinylboration protocol described here exhibited excellent chemoselectivity when equal amounts of two olefins were present. In the first competition experiment with cyclohexene, only the product of **3** was isolated in 53% yield (Fig. 4a-1). In an independent reaction, cyclohexene showed no reactivity under the standard conditions either. These results indicated that the nitrogen-based ligand enabled a different reactivity from the prior ligand-free system.^12*b*^ Furthermore, only product **3** from styrene was observed in 66% yield in the competition reaction with an unactivated monosubstituted olefin (Fig. 4a-2). Intriguingly, a high selectivity was also displayed in the competition reaction with allylbenzene (Fig. 4a-3). Accordingly, the addition of three olefins as above all sparingly inhibited the styrene vinylboration. However, the addition of a strong electron-deficient olefin, methyl acrylate, highly influenced the reactivity (Fig. 4a-4).

To gain insight into the details of the olefin insertion step, a terminal D-labeled olefin **1-d2** was synthesized and examined in the vinylboration reaction. The product **3-d2** was isolated in 90% yield, with almost no deuterium lost at the terminal position (Fig. 4b). These results combined with the exclusive *cis*-diastereoselectivity reveal that reversible β-H elimination does not likely occur in the transformation.

**Fig. 5:**
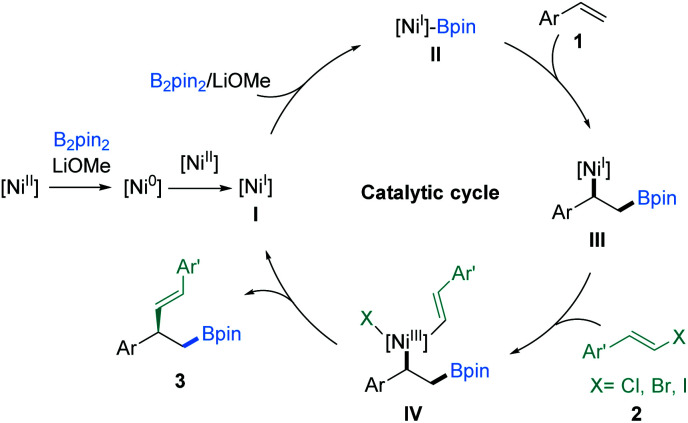
Proposed catalytic pathway.

Next, intermolecular protoboration studies^12*b*^ (Fig. S1 and S2†) supported the generation of the Ni(i) complex (**III**) (Fig. 5). The long-lived radical intermediates might not be involved in the reaction processes according to the control experiment results^12*c*,15*f*^ (Fig. S3†). However, the electron paramagnetic resonance (EPR) experiment result was consistent with Yin's report.^13*b*^ Accordingly, a proposed catalytic cycle was considered based on the present related literature^12,13^ and the preliminary mechanism researched to rationalize this transformation. As illustrated in Fig. 5, the reaction is initiated by the generation of a Ni(i) species (**I**) from the Ni(ii) precatalyst through a comproportionation process, which undergoes transmetalation with B_2_pin_2_ to generate the Ni(i)-Bpin (**II**). Then, olefin migratory insertion in an *anti*-Markovnikov fashion leads to the formation of a stable benzyl-Ni(i) species (**III**),^21^ which subsequently reacts with a vinyl halide to deliver a Ni(iii) intermediate **IV**. Eventually, reductive elimination from **IV** generates the asymmetric vinylboration product **3** and regenerates the Ni(i) species (**I**). However, we cannot exclude the pathway of bimetallic transmetalation between two nickel species at this stage.^22,23^

## Conclusions

In summary, we have developed a chiral bisoxazoline ligand-enabled, Ni-catalyzed asymmetric *anti*-Markovnikov vinylboration reaction. A set of 2-boryl-1,1-arylvinylalkanes, significant pharmaceutically interesting scaffolds, are efficiently synthesized from simple alkenes and vinyl halides in the presence of a diboron reagent under mild conditions. The present method is tolerant of a variety of functional groups, affording the coupling products generally in modest to high yields and excellent enantioselectivities. Significantly, this work provides a convenient tool for asymmetric functionalization of natural products and drugs by facile introduction of chiral benzylic alkenylboration subunits. The preliminary mechanistic study indicates that a reversible β-H elimination is less likely to occur after the formation of a stable benzylic nickel species. Further mechanistic studies are currently in progress in our laboratory.

## Data availability

All experimental data and detailed procedures are available in the ESI.†

## Author contributions

Y. Y. and J. L. designed the research. Y. Y. performed the experiments, analyzed the data, and wrote the paper. B. X. and S. J. synthesized some of the vinyl halides. R. B., S. L., T. X. and X.-Y. Y. revised the paper.

## Conflicts of interest

There are no conflicts to declare.

## Supplementary Material

SC-012-D1SC04071E-s001
