# Supplementary material for: Nickel-catalyzed enantioselective 1,2-vinylboration of styrenes
Source: Chem Sci. 2021 Sep 7;12(39):13209–15. doi: 10.1039/d1sc04071e (PMC8513998; doi:10.1039/d1sc04071e)
Supplement: SC-012-D1SC04071E-s001 [file SC-012-D1SC04071E-s001.pdf]

Electronic Supplementary Material (ESI) for Chemical Science.
This journal is © The Royal Society of Chemistry 2021

Supporting Information

Nickel-Catalyzed Enantioselective 1,2-Vinylboration of Styrenes

Yang Ye,*[,a,b,†] Jiandong Liu,[c,†] Bing Xu,[a,b] Songwei Jiang,[a,b] Renren Bai,[a,b] Shijun Li,[d] Tian Xie*[,a,b] and Xiang-Yang Ye*[,a,b]

[a]School of Pharmacy, Hangzhou Normal University, Hangzhou, Zhejiang 311121, PR China

[b]Key Laboratory of Elemene Class Anti-Cancer Chinese Medicines; Engineering Laboratory of Development and Application of Traditional Chinese Medicines; Collaborative Innovation Center of Traditional Chinese Medicines of Zhejiang Province, Hangzhou Normal University, Hangzhou, Zhejiang 311121, China

[c]Center for Supramolecular Chemistry and Catalysis and Department of Chemistry, Shanghai University, Shanghai 200444, PR China

[d]College of Material, Chemistry and Chemical Engineering, Hangzhou Normal University, Hangzhou, Zhejiang 311121, PR China

yeyang0711@163.com

xyye@hznu.edu.cn

xbs@hznu.edu.cn

Table of Contents

I. General Information……………………………..…………………S2

II. Nickel-Catalyzed Enantioselective 1,2-Vinylboration S3-S40

III. Preparation of Vinyl Halides……………………...……..........……S41-S42

IV. Preparation Part of Alkenes……………………...………............…S43-S44

V. Competition Experiments S45

VI. Mechanistic Investigations………..…………....…..........….............S45-S49

VII. References…………………………………………………..........S49

VIII. Spectroscopic Data (NMR Spectrum) ……………………….........S50-S113

IX. Spectroscopic Data (HPLC Trace) ……………...................................S114-S171

I. General Information

1. Chemicals and Reagents

All manipulations were carried out under an atmosphere of nitrogen using standard Schlenk or glove box techniques. Anhydrous THF was distilled by sodium/benzophenone ketyl prior to use. 1,4-Dioxane (99.5%, extra dry, Acros) was purchased and used directly. Deuterated solvents were used as received ($CDCl_3$ from Maclin Co., China). $NiBr_2$ (Alfa Aesar), $NiCl_2$ (Alfa Aesar), $Ni(COD)_2$ (Alfa Aesar), $NiCl_2$•DME (Alfa Aesar), $NiBr_2$•DME (Alfa Aesar)，LiOMe (J＆K), B_2pin_2 (Alfa Aesar) were used as received. **L1**•$NiBr_2$ were synthesized according to literature procdures[1,2]. 2,9-Dimethyl-1,10-phenanthroline (>99%, Alfa Aesar)were purchased and used directly. Unless otherwise noted, all other reagents and starting materials were purchased from commercial sources and used without further purification.

2. Physical Methods

Column chromatography was performed using silica gel 200-300 mesh (purchased from Qingdao-Haiyang Co., China) as the solid support. All NMR spectra were recorded on Bruker Avance 500 MHz spectrometers. ^{1}H NMR and ^{13}C NMR chemical shifts are reported in δ units, parts per million (ppm) relative to the chemical shift of residual solvent. Reference peaks for chloroform in ^{1}H NMR and ^{13}C NMR spectra were set at 7.26 ppm and 77.16 ppm, respectively. High-resolution mass spectra (HRMS) were obtained using a Bruker APEXIII 7.0 or IonSpec 4.7 TESLA FTMS instruments. Melting points were recorded on a micro melting point apparatus (X-4, YUHUA Co., Ltd, Gongyi, China). GC chromatograms were recorded on a GCMS-QP2010 SE (SHIMADZU) using an Agilent column CP7502 and Rxi-5 ms (Restek). Ultra Fast liquid chromatography was performed on Shimadzu Chromatographs (LC-2030 Plus) using Daicel Chiralcel columns (250 mm). Optical rotation analyses were performed on an Anton Paar MCP-500 optical instrument, using a 100 mm pathlength cell at 589 nm with [α]D values reported in degrees; concentration (c) is in g/100 mL.

II. Nickel-Catalyzed Enantioselective 1,2-Vinylboration

1. Reaction Conditions Optimization

Table S1. Optimization for the reaction of **1** with **2**.

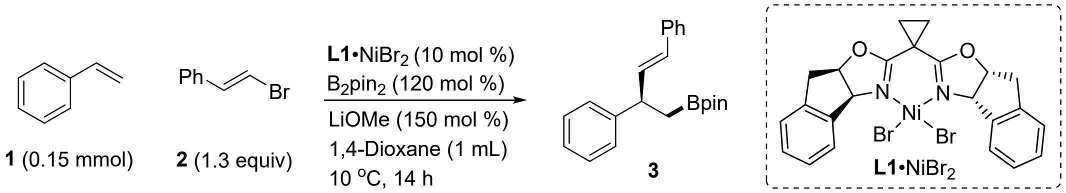

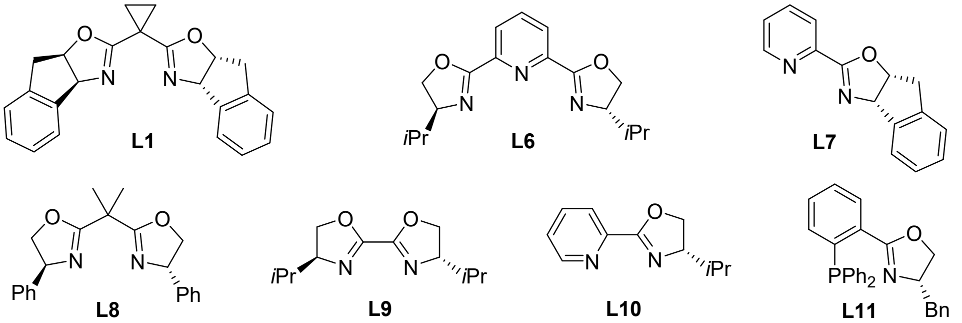

entry	Variation from standard conditions[a]	Yield [%][b]	*ee* [%][c]
1	none	92 (93)	96
2	**2** (1 equiv)	78	96
3	DMA instead of 1,4-dioxane	41	93
4	$NiCl_2$, **L1** instead of **L1**•$NiBr_2$	60	82
5	$NiBr_2$, **L1** instead of **L1**•$NiBr_2$	39	83
6	$Ni(COD)_2$, **L1** instead of **L1**•$NiBr_2$	27	80
7	$NiCl_2$•DME, **L1** instead of **L1**•$NiBr_2$	76	94
8	$NiBr_2$•DME, **L6** instead of **L1**•$NiBr_2$	0	0
9	$NiBr_2$•DME, **L7** instead of **L1**•$NiBr_2$	50	71
10	$NiBr_2$•DME, **L8** instead of **L1**•$NiBr_2$	30	33
11	$NiBr_2$•DME, **L9** instead of **L1**•$NiBr_2$	26	8
12	$NiBr_2$•DME, **L10** instead of **L1**•$NiBr_2$	60	57
13	$NiBr_2$•DME, **L11** instead of **L1**•$NiBr_2$	32	57

[a]Standard conditions: **1** (0.150 mmol, 1.0 equiv), **2** (0.195 mmol, 1.3 equiv), B_2pin_2 (0.180 mmol, 1.2 equiv), **L1**•$NiBr_2$ (10 mol%), LiOMe (0.225 mmol, 1.5 equiv), 1,4-dioxane (1.0 mL), 10 °C, 14 h. [b]Yields determined by crude ^1H NMR using 2,5-dimethylfuran as the internal standard. The yield in parentheses is the isolated yield. [c]The *ee* values were determined by HPLC on a chiral stationary phase.

Ineffective Substrates:

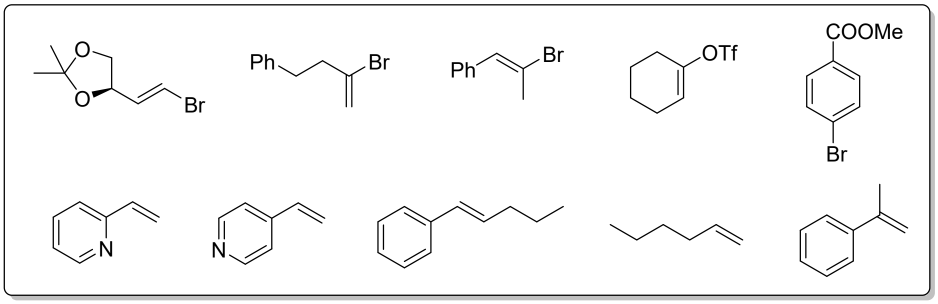

2. General Procedure for Nickel-Catalyzed Enantioselective 1,2-Vinylboration

To an oven-dried 8 mL screw-cap vial equipped with a magnetic stir bar was charged with alkenyl bromide (0.195 mmol, 1.3 equiv, if solid)), alkene (0.150 mmol, 1.0 equiv, if solid), **L1**•$NiBr_2$ (8.6 mg, 0.015 mmol, 10 mol%). The vial was introduced into a glove box, to which LiOMe (8.6 mg, 0.225 mmol, 1.5 equiv) and B_2pin_2 (45.7 mg, 0.180 mmol, 1.2 equiv) was added. The tube was sealed with a teflon-lined screw cap, removed from the glove box. Alkenyl bromide (0.195 mmol, 1.3 equiv, if liquid), alkene (0.150 mmol, 1.0 equiv, if liquid), and 1,4-dioxane (1.0 mL) were added via a syringe. The reaction mixture was allowed to stir at 10 °C for 14 h. After the reaction was complete, the reaction mixture was directly filtered through a short pad of silica gel (using ethyl acetate in petroleum ether) to give the product. All yields were an average of two runs.

3. Details of the Experimental Data

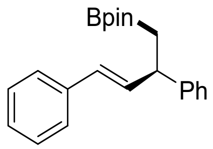

(*R,E*)-2-(2,4-Diphenylbut-3-en-1-yl)-4,4,5,5-tetramethyl-1,3,2-dioxaborolane (3).

The title compound was prepared following the general procedure using styrene (15.6 mg, 0.150 mmol, 1.0 equiv), (*E*)-(2-bromovinyl)benzene (35.6 mg, 0.195 mmol, 1.3 equiv). After purification by column chromatography (using 1% ethyl acetate in petroleum ether), the title compound was isolated in 93% yield (46.6 mg, 96% *ee*) as a colorless oil.

This compound was also prepared according to the general procedure using styrene (15.6 mg, 0.150 mmol, 1.0 equiv), (*E*)-(2-chlorovinyl)benzene (27.0 mg, 0.195 mmol, 1.3 equiv). After purification by column chromatography (using 1% ethyl acetate in petroleum ether), the title compound was isolated in 89% yield (44.6 mg, 96% *ee*) as a colorless oil.

^{1}H NMR (500 MHz, $CDCl_3$): δ 7.30 (d, J = 7.4 Hz, 2H), 7.26–7.20 (m, 6H), 7.14 (dt, J = 14.4, 5.5 Hz, 2H), 6.43–6.29 (m, 2H), 3.76 (dd, J = 14.6, 7.8 Hz, 1H), 1.36 (dd, J = 16.7, 7.3 Hz, 2H), 1.11 (s, 12H).

^{13}C NMR (126 MHz, $CDCl_3$): δ 145.88, 137.74, 135.76, 128.53, 128.47, 128.43, 127.59, 127.04, 126.29, 126.23, 83.29, 44.51, 29.83, 24.88 (d, J = 16.3 Hz).

HRMS (ESI) m/z ($[M+H]^+$) calcd for $C_{22}H_{28}BO_2$: 335.2177. Found: 335.2179.

HPLC analysis: CHIRALCEL OD-H column, 0.5% *i*PrOH in hexane, 0.5 mL/min, 254 nm UV detector, t_R (minor) = 9.5 min, t_R (major) = 10.5 min.

$[\alpha]_D^{20}$ = –2 (c = 0.18, $CHCl_3$).

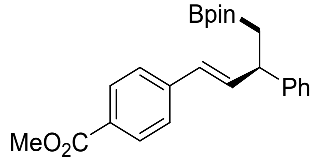

Methyl-(*R,E*)-4-(3-phenyl-4-(4,4,5,5-tetramethyl-1,3,2-dioxaborolan-2-yl)but-1-en-1-yl)benzoate (4).

The title compound was prepared following the general procedure using styrene (15.6 mg, 0.150 mmol, 1.0 equiv), methyl (*E*)-4-(2-bromovinyl)benzoate (47.0 mg, 0.195 mmol, 1.3 equiv). After purification by column chromatography (using 1% ethyl acetate in petroleum ether), the title compound was isolated in 86% yield (50.5 mg, 93% *ee*) as a colorless oil.

^{1}H NMR (500 MHz, $CDCl_3$): δ 7.94 (d, J = 8.4 Hz, 2H), 7.38 (d, J = 8.4 Hz, 2H), 7.32–7.27 (m, 4H), 7.22–7.17 (m, 1H), 6.49 (dt, J = 33.6, 11.5 Hz, 2H), 3.89 (s, 3H), 3.80 (q, J = 7.7 Hz, 1H), 1.42–1.34 (m, 2H), 1.13 (s, 12H).

^{13}C NMR (126 MHz, $CDCl_3$): δ 167.11, 145.31, 142.31, 138.66, 129.96, 128.58, 128.54, 127.68, 127.60, 126.44, 126.15, 83.37, 52.12, 44.67, 29.83, 24.87 (d, J = 15.3 Hz).

HRMS (ESI) m/z ($[M+H]^+$) calcd for $C_{24}H_{30}BO_4$: 393.2232. Found: 393.2229.

HPLC analysis: CHIRALCEL OD-H column, 0.5% *i*PrOH in hexane, 0.5 mL/min, 254 nm UV detector, t_R (minor) = 17.6 min, t_R (major) = 19.1 min.

$[\alpha]_D^{20}$ = +2 (c = 0.26, $CHCl_3$).

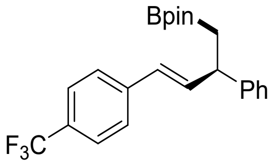

(*R,E*)-4,4,5,5-Tetramethyl-2-(2-phenyl-4-(4-(trifluoromethyl)phenyl)but-3-en-1-yl)-1,3,2-dioxaborolane (5).

The title compound was prepared following the general procedure using styrene (15.6 mg, 0.150 mmol, 1.0 equiv), (*E*)-1-(2-bromovinyl)-4-(trifluoromethyl)benzene (48.9 mg, 0.195 mmol, 1.3 equiv). After purification by column chromatography (using 1% ethyl acetate in petroleum ether), the title compound was isolated in 80% yield (48.2 mg, 96% *ee*) as a colorless oil.

^{1}H NMR (500 MHz, $CDCl_3$): δ 7.52 (d, J = 8.2 Hz, 2H), 7.41 (d, J = 8.2 Hz, 2H), 7.30 (tt, J = 8.2, 3.9 Hz, 4H), 7.22–7.18 (m, 1H), 6.56–6.39 (m, 2H), 3.81 (dd, J = 15.0, 7.6 Hz, 1H), 1.43–1.36 (m, 2H), 1.14 (s, 12H).

^{13}C NMR (126 MHz, $CDCl_3$): δ 145.30, 141.25, 138.58, 129.02, 128.76, 128.60, 127.61, 127.25, 126.47, 126.40, 125.53 (q, J = 3.7 Hz), 83.39, 44.58, 29.85, 24.88 (d, J = 16.3 Hz).

HRMS (ESI) m/z ($[M+H]^+$) calcd for $C_{23}H_{27}BF_3O_2$: 403.2051. Found: 403.2050.

HPLC analysis: CHIRALCEL OD-H column, 0.5% *i*PrOH in hexane, 0.5 mL/min, 254 nm UV detector, t_R (minor) = 9.1 min, t_R (major) = 9.6 min.

$[\alpha]_D^{20}$ = +2 (c = 0.24, $CHCl_3$).

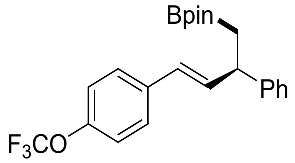

(*R,E*)-4,4,5,5-Tetramethyl-2-(2-phenyl-4-(4-(trifluoromethoxy)phenyl)but-3-en-1-yl)-1,3,2-dioxaborolane (6).

The title compound was prepared following the general procedure using styrene (15.6 mg, 0.150 mmol, 1.0 equiv), (*E*)-1-(2-bromovinyl)-4-(trifluoromethoxy)benzene (52.1 mg, 0.195 mmol, 1.3 equiv). After purification by column chromatography (using 1% ethyl acetate in petroleum ether), the title compound was isolated in 83% yield (52.0 mg, 96% *ee*) as a colorless oil.

^{1}H NMR (500 MHz, $CDCl_3$): δ 7.34–7.31 (m, 2H), 7.28 (d, J = 6.5 Hz, 3H), 7.25 (s, 1H), 7.21–7.16 (m, 1H), 7.11 (d, J = 8.1 Hz, 2H), 6.42–6.32 (m, 2H), 3.78 (dd, J = 13.9, 8.0 Hz, 1H), 1.41–1.34 (m, 2H), 1.13 (s, 12H).

^{13}C NMR (126 MHz, $CDCl_3$): δ 148.18, 145.53, 136.96, 136.56, 128.55, 127.59, 127.44, 127.01, 126.38, 121.11, 83.35, 44.49, 29.84, 24.87 (d, J = 15.5 Hz).

HRMS (ESI) m/z ($[M+H]^+$) calcd for $C_{23}H_{27}BF_3O_3$: 419.2000. Found: 419.2003.

HPLC analysis: CHIRALCEL OD-H column, 0.5% *i*PrOH in hexane, 0.5 mL/min, 254 nm UV detector, t_R (minor) = 9.0 min, t_R (major) = 9.9 min.

$[\alpha]_D^{20}$ = +4 (c = 0.20, $CHCl_3$).

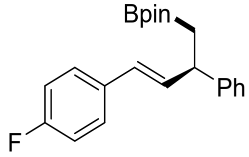

(*R,E*)-2-(4-(4-Fluorophenyl)-2-phenylbut-3-en-1-yl)-4,4,5,5-tetramethyl-1,3,2-dioxaborolane (7).

The title compound was prepared following the general procedure using styrene (15.6 mg, 0.150 mmol, 1.0 equiv), (*E*)-1-(2-bromovinyl)-4-fluorobenzene (39.2 mg, 0.195 mmol, 1.3 equiv). After purification by column chromatography (using 1% ethyl acetate in petroleum ether), the title compound was isolated in 91% yield (48.0 mg, 97% *ee*) as a colorless oil.

^{1}H NMR (500 MHz, $CDCl_3$): δ 7.33–7.27 (m, 6H), 7.21–7.17 (m, 1H), 6.96 (t, J = 8.7 Hz, 2H), 6.32 (dt, J = 15.8, 11.5 Hz, 2H), 3.77 (q, J = 7.7 Hz, 1H), 1.39 (dd, J = 14.8, 6.6 Hz, 2H), 1.14 (s, 12H).

^{13}C NMR (126 MHz, $CDCl_3$): δ 145.78, 135.55, 133.87, 128.52, 127.73, 127.57, 127.28, 126.30, 115.49, 115.32, 83.32, 44.50, 29.84, 24.88 (d, J = 16.0 Hz).

HRMS (ESI) m/z ($[M+H]^+$) calcd for $C_{22}H_{27}BFO_2$: 353.2083. Found: 353.2086.

HPLC analysis: CHIRALCEL OD-H column, 0.5% *i*PrOH in hexane, 0.5 mL/min, 254 nm UV detector, t_R (minor) = 9.0 min, t_R (major) = 9.6 min.

$[\alpha]_D^{20}$ = –2 (c = 0.25, $CHCl_3$).

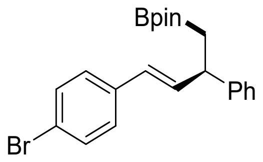

(*R,E*)-2-(4-(4-Bromophenyl)-2-phenylbut-3-en-1-yl)-4,4,5,5-tetramethyl-1,3,2-dioxaborolane (8).

The title compound was prepared following the general procedure using styrene (15.6 mg, 0.150 mmol, 1.0 equiv), (*E*)-1-bromo-4-(2-bromovinyl)benzene (51.1 mg, 0.195 mmol, 1.3 equiv). After purification by column chromatography (using 1% ethyl acetate in petroleum ether), the title compound was isolated in 81% yield (50.2 mg, 80% *ee*) as a colorless oil.

^{1}H NMR (500 MHz, $CDCl_3$): δ 7.39 (d, J = 8.5 Hz, 2H), 7.32–7.27 (m, 4H), 7.19 (dd, J = 7.4, 3.5 Hz, 3H), 6.43–6.31 (m, 2H), 3.77 (dd, J = 13.7, 7.9 Hz, 1H), 1.38 (dt, J = 18.7, 7.5 Hz, 2H), 1.14 (s, 12H).

^{13}C NMR (126 MHz, $CDCl_3$): δ 145.55, 136.68, 136.63, 131.62, 128.54, 127.83, 127.56, 127.32, 126.36, 120.70, 83.33, 44.54, 29.83, 24.88 (d, J = 16.6 Hz).

HRMS (ESI) m/z ($[M+Na]^+$) calcd for $C_{22}H_{26}BBrNaO_2$: 435.1101. Found: 435.1084.

HPLC analysis: CHIRALCEL OD-H column, 0.5% *i*PrOH in hexane, 0.5 mL/min, 254 nm UV detector, t_R (minor) = 10.1 min, t_R (major) = 10.8 min.

$[\alpha]_D^{20}$ = –1 (c = 0.36, $CHCl_3$).

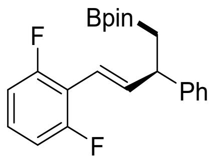

(*R,E*)-2-(4-(2,6-Difluorophenyl)-2-phenylbut-3-en-1-yl)-4,4,5,5-tetramethyl-1,3,2-dioxaborolane (9).

The title compound was prepared following the general procedure using styrene (15.6 mg, 0.150 mmol, 1.0 equiv), (*E*)-2-(2-bromovinyl)-1,3-difluorobenzene (42.7 mg, 0.195 mmol, 1.3 equiv). After purification by column chromatography (using 1% ethyl acetate in petroleum ether),

the title compound was isolated in 94% yield (52.2 mg, 92% *ee*) as a colorless oil.

^{1}H NMR (500 MHz, $CDCl_3$): δ 7.32–7.28 (m, 4H), 7.18 (ddd, *J* = 6.8, 5.2, 3.4 Hz, 1H), 7.09–7.05 (m, 1H), 6.82 (t, *J* = 8.4 Hz, 2H), 6.72 (dd, *J* = 16.2, 7.7 Hz, 1H), 6.46 (dd, *J* = 16.3, 0.9 Hz, 1H), 3.80 (q, *J* = 7.8 Hz, 1H), 1.42–1.36 (m, 2H), 1.15 (s, 12H).

^{13}C NMR (126 MHz, $CDCl_3$): δ 161.95, (d, *J* = 8.0 Hz), 159.96 (d, *J* = 8.1 Hz), 145.51, 142.91 (t, *J* = 7.5 Hz), 128.53, 127.61, 127.40 (t, *J* = 10.1 Hz), 126.33, 115.08, 111.46 (dd, *J* = 20.7, 6.0 Hz), 83.34, 45.84, 29.84, 24.85 (d, *J* = 14.1 Hz).

HRMS (ESI) m/z ($[M+H]^+$) calcd for $C_{22}H_{26}BF_2O_2$: 371.1988. Found: 371.1990.

HPLC analysis: CHIRALCEL OD-H column, 0.5% *i*PrOH in hexane, 0.5 mL/min, 254 nm UV detector, t_R (minor) = 8.2 min, t_R (major) = 9.6 min.

$[\alpha]_D^{20}$ = –4 (c = 0.31, $CHCl_3$).

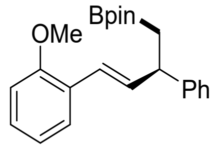

(*R,E*)-2-(4-(2-Methoxyphenyl)-2-phenylbut-3-en-1-yl)-4,4,5,5-tetramethyl-1,3,2-dioxaborolane (10).

The title compound was prepared following the general procedure using styrene (15.6 mg, 0.150 mmol, 1.0 equiv), (*E*)-1-(2-bromovinyl)-2-methoxybenzene (41.6 mg, 0.195 mmol, 1.3 equiv). After purification by column chromatography (using 1% ethyl acetate in petroleum ether), the title compound was isolated in 83% yield (45.3 mg, 96% *ee*) as a colorless oil.

^{1}H NMR (500 MHz, $CDCl_3$): δ 7.41 (dd, *J* = 7.6, 1.6 Hz, 1H), 7.33–7.26 (m, 4H), 7.20–7.14 (m, 2H), 6.87 (t, *J* = 7.5 Hz, 1H), 6.85–6.78 (m, 2H), 6.37 (dd, *J* = 15.9, 7.6 Hz, 1H), 3.86–3.77 (m, 4H), 1.44–1.36 (m, 2H), 1.15 (d, *J* = 0.7 Hz, 12H).

^{13}C NMR (126 MHz, $CDCl_3$): δ 156.60, 146.27, 136.13, 128.42, 128.02, 127.61, 126.82, 126.63, 126.09, 122.98, 120.65, 110.88, 83.25, 55.50, 44.96, 29.84, 24.87 (d, *J* = 12.3 Hz).

HRMS (ESI) m/z ($[M+H]^+$) calcd for $C_{23}H_{30}BO_3$: 365.2283. Found: 365.2284.

HPLC analysis: CHIRALCEL OD-H column, 0.5% *i*PrOH in hexane, 0.5 mL/min, 254 nm UV detector, t_R (minor) = 11.6 min, t_R (major) = 14.3 min.

$[\alpha]_D^{20}$ = –10 (c = 0.25, $CHCl_3$).

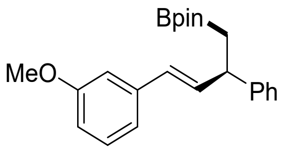

(*R*,*E*)-2-(4-(3-Methoxyphenyl)-2-phenylbut-3-en-1-yl)-4,4,5,5-tetramethyl-1,3,2-dioxaborolane (11).

The title compound was prepared following the general procedure using styrene (15.6 mg, 0.150 mmol, 1.0 equiv), (*E*)-1-(2-bromovinyl)-3-methoxybenzene (41.6 mg, 0.195 mmol, 1.3 equiv). After purification by column chromatography (using 1% ethyl acetate in petroleum ether), the title compound was isolated in 85% yield (46.4 mg, 96% *ee*) as a colorless oil.

^{1}H NMR (500 MHz, $CDCl_3$): δ 7.29 (d, J = 4.3 Hz, 4H), 7.22–7.15 (m, 2H), 6.93 (d, J = 7.7 Hz, 1H), 6.91–6.85 (m, 1H), 6.74 (dd, J = 7.9, 2.2 Hz, 1H), 6.39 (d, J = 4.9 Hz, 2H), 3.83–3.74 (m, 4H), 1.40 (dd, J = 16.6, 7.4 Hz, 2H), 1.15 (s, 12H).

^{13}C NMR (126 MHz, $CDCl_3$): δ 159.86, 145.82, 139.23, 136.07, 129.49, 128.49, 128.36, 127.62, 126.26, 119.05, 112.86, 111.44, 83.31, 55.30, 44.47, 31.72, 24.89 (d, J = 16.0 Hz).

HRMS (ESI) m/z ([M+H]$^+$) calcd for $C_{23}H_{30}BO_3$: 365.2283. Found: 365.2287.

HPLC analysis: CHIRALCEL OD-H column, 0.5% *i*PrOH in hexane, 0.5 mL/min, 254 nm UV detector, t_R (minor) = 12.5 min, t_R (major) = 14.9 min.

$[\alpha]_D^{20}$ = –2 (c =0.29, $CHCl_3$).

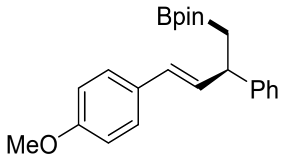

(*R*,*E*)-2-(4-(4-Methoxyphenyl)-2-phenylbut-3-en-1-yl)-4,4,5,5-tetramethyl-1,3,2-dioxaborolane (12).

The title compound was prepared following the general procedure using styrene (15.6 mg, 0.150 mmol, 1.0 equiv), (*E*)-1-(2-bromovinyl)-4-methoxybenzene (41.6 mg, 0.195 mmol, 1.3 equiv). After purification by column chromatography (using 1% ethyl acetate in petroleum ether), the title compound was isolated in 80% yield (43.7 mg, 96% *ee*) as a colorless oil.

This compound was also prepared according to the general procedure using styrene (15.6 mg, 0.150 mmol, 1.0 equiv), (*Z*)-1-(2-bromovinyl)-4-methoxybenzene (41.6 mg, 0.195 mmol, 1.3 equiv). After purification by column chromatography (using 1% ethyl acetate in petroleum ether), the title compound was isolated in 76% yield (41.5 mg, 96% *ee*) as a colorless oil.

^{1}H NMR (500 MHz, $CDCl_3$): δ 7.29 (d, J = 4.5 Hz, 4H), 7.28–7.25 (m, 2H), 7.18 (dt, J = 12.4, 4.1 Hz, 1H), 6.82 (d, J = 8.8 Hz, 2H), 6.37 (d, J = 15.8 Hz, 1H), 6.24 (dd, J = 15.8, 7.3 Hz, 1H), 3.79 (s, 3H), 3.78–

3.74 (m, 1H), 1.41–1.34 (m, 2H), 1.14 (s, 12H).

^{13}C NMR (126 MHz, $CDCl_3$): δ 158.85, 146.17, 133.66, 130.58, 128.45, 127.83, 127.58, 127.38, 126.16, 113.97, 83.27, 55.39, 44.51, 29.83, 24.89 (d, J = 16.7 Hz).

HRMS (ESI) m/z ([M+H]$^+$) calcd for $C_{23}H_{30}BO_3$: 365.2283. Found: 365.2287.

HPLC analysis: CHIRALCEL OD-H column, 0.5% *i*PrOH in hexane, 0.5 mL/min, 254 nm UV detector, t_R (minor) = 11.2 min, t_R (major) = 12.1 min.

[α]$_D^{20}$ = +127 (c = 0.25, $CHCl_3$).

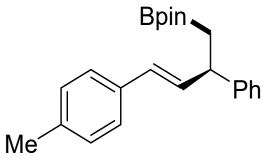

(*R,E*)-4,4,5,5-Tetramethyl-2-(2-phenyl-4-(p-tolyl)but-3-en-1-yl)-1,3,2-dioxaborolane (13).

The title compound was prepared following the general procedure using styrene (15.6 mg, 0.150 mmol, 1.0 equiv), (*E*)-1-(2-bromovinyl)-4-methylbenzene (38.4 mg, 0.195 mmol, 1.3 equiv). After purification by column chromatography (using 1% ethyl acetate in petroleum ether), the title compound was isolated in 89% yield (46.4 mg, 96% *ee*) as a pale yellow oil.

^{1}H NMR (500 MHz, $CDCl_3$): δ 7.29 (d, J = 4.5 Hz, 4H), 7.23 (d, J = 8.0 Hz, 2H), 7.20–7.17 (m, 1H), 7.08 (d, J = 8.0 Hz, 2H), 6.35 (dt, J = 15.8, 11.4 Hz, 2H), 3.78 (dd, J = 15.4, 7.6 Hz, 1H), 2.31 (s, 3H), 1.40–1.34 (m, 2H), 1.15 (s, 12H).

^{13}C NMR (126 MHz, $CDCl_3$): δ 146.08, 136.74, 134.97, 134.73, 129.24, 128.46, 128.29, 127.60, 126.38, 126.20, 83.29, 44.50, 29.84, 24.90 (d, J = 17.5 Hz), 21.27.

HRMS (ESI) m/z ([M+H]$^+$) calcd for $C_{23}H_{30}BO_2$: 349.2333. Found: 349.2332.

HPLC analysis: CHIRALCEL OD-H column, 0.5% *i*PrOH in hexane, 0.5 mL/min, 254 nm UV detector, t_R (minor) = 8.7 min, t_R (major) = 9.3 min.

[α]$_D^{20}$ = –1 (c = 0.22, $CHCl_3$).

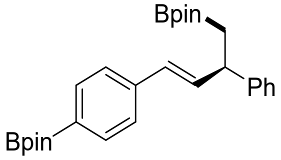

(*R,E*)-4,4,5,5-Tetramethyl-2-(4-(3-phenyl-4-(4,4,5,5-tetramethyl-1,3,2-dioxaborolan-2-yl)but-1-en-1-yl)phenyl)-1,3,2-dioxaborolane (14).

The title compound was prepared following the general procedure using styrene (15.6 mg, 0.150 mmol, 1.0 equiv), (*E*)-2-(4-(2-bromovinyl)phenyl)-4,4,5,5-tetramethyl-1,3,2-dioxaborolane (60.3

mg, 0.195 mmol, 1.3 equiv). After purification by column chromatography (using 1% ethyl acetate in petroleum ether), the title compound was isolated in 89% yield (61.4 mg, 94% *ee*) as a white solid.

^{1}H NMR (500 MHz, $CDCl_3$): δ 7.72 (d, J = 8.1 Hz, 2H), 7.33 (d, J = 8.1 Hz, 2H), 7.29 (d, J = 4.4 Hz, 4H), 7.21–7.16 (m, 1H), 6.50–6.39 (m, 2H), 3.80 (dd, J = 13.4, 8.0 Hz, 1H), 1.43–1.37 (m, 2H), 1.34 (s, 12H), 1.14 (s, 12H).

^{13}C NMR (126 MHz, $CDCl_3$): δ 145.75, 140.51, 136.82, 135.07, 128.55, 128.50, 127.59, 126.28, 125.84, 125.63, 83.80, 83.31, 44.62, 29.83, 24.99, 24.88 (d, J = 17.6 Hz).

HRMS (ESI) m/z ($[M+H]^+$) calcd for $C_{28}H_{39}B_2O_4$: 461.3029. Found: 461.3028.

M.p.: 119-120 ºC.

HPLC analysis: CHIRALCEL OD-H column, 0.5% *i*PrOH in hexane, 0.5 mL/min, 254 nm UV detector, t_R (minor) = 9.9 min, t_R (major) = 10.4 min.

$[\alpha]_D^{20}$ = –3 (c = 0.24, $CHCl_3$).

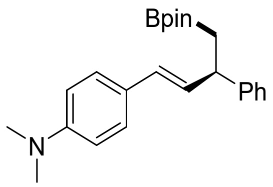

(*R,E*)-N,N-Dimethyl-4-(3-phenyl-4-(4,4,5,5-tetramethyl-1,3,2-dioxaborolan-2-yl)but-1-en-1-yl)aniline (15).

The title compound was prepared following the general procedure using styrene (15.6 mg, 0.150 mmol, 1.0 equiv), (*E*)-4-(2-bromovinyl)-N,N-dimethylaniline (44.1 mg, 0.195 mmol, 1.3 equiv). After purification by column chromatography (using 1% ethyl acetate in petroleum ether), the title compound was isolated in 88% yield (49.7 mg, 94% *ee*) as a pale yellow solid.

^{1}H NMR (500 MHz, $CDCl_3$): δ 7.30 (t, J = 6.8 Hz, 4H), 7.24 (d, J = 8.8 Hz, 2H), 7.20–7.15 (m, 1H), 6.68 (d, J = 8.5 Hz, 2H), 6.36 (d, J = 15.8 Hz, 1H), 6.19 (dd, J = 15.8, 7.3 Hz, 1H), 3.77 (q, J = 7.7 Hz, 1H), 2.94 (s, 6H), 1.42–1.36 (m, 2H), 1.16 (s, 12H).

^{13}C NMR (126 MHz, $CDCl_3$): δ 146.53, 131.77, 130.91, 128.37, 128.20, 127.58, 127.31, 127.17, 126.03, 112.82, 83.22, 44.49, 40.85, 29.82, 24.89 (d, J = 17.6 Hz).

HRMS (ESI) m/z ($[M+H]^+$) calcd for $C_{24}H_{33}BNO_2$: 378.2599. Found: 378.2597.

M.p.: 81-82 ºC.

HPLC analysis: CHIRALCEL OD-H column, 0.5% *i*PrOH in hexane, 0.5 mL/min, 254 nm UV

detector, t_R (minor) = 12.7 min, t_R (major) = 14.9 min.

[α]$_D$^{20} = +2 (c = 0.29, $CHCl_3$).

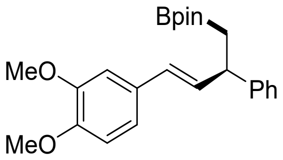

(*R,E*)-2-(4-(3,4-Dimethoxyphenyl)-2-phenylbut-3-en-1-yl)-4,4,5,5-tetramethyl-1,3,2-dioxaborolane (16).

The title compound was prepared following the general procedure using styrene (15.6 mg, 0.150 mmol, 1.0 equiv), (*E*)-4-(2-bromovinyl)-1,2-dimethoxybenzene (47.4 mg, 0.195 mmol, 1.3 equiv). After purification by column chromatography (using 5% ethyl acetate in petroleum ether), the title compound was isolated in 90% yield (53.2 mg, 96% *ee*) as a colorless oil.

^{1}H NMR (500 MHz, $CDCl_3$): δ 7.29 (d, *J* = 4.3 Hz, 4H), 7.20–7.15 (m, 1H), 6.89 (d, *J* = 1.8 Hz, 1H), 6.85 (dd, *J* = 8.3, 1.9 Hz, 1H), 6.77 (d, *J* = 8.3 Hz, 1H), 6.35 (d, *J* = 15.8 Hz, 1H), 6.24 (dd, *J* = 15.8, 7.2 Hz, 1H), 3.86 (s, 3H), 3.85 (s, 3H), 3.77 (q, *J* = 7.7 Hz, 1H), 1.41–1.34 (m, 2H), 1.14 (s, 12H).

^{13}C NMR (126 MHz, $CDCl_3$): δ 149.05, 148.42, 146.05, 133.85, 130.88, 128.47 128.12, 127.61, 126.21, 119.35, 111.18, 108.67, 83.29, 56.02, 55.90, 44.47, 29.82, 24.89 (d, *J* = 14.6 Hz).

HRMS (ESI) m/z ($[M+H]^+$) calcd for $C_{24}H_{32}BO_4$: 395.2388. Found: 395.2387.

HPLC analysis: CHIRALCEL OD-H column, 5% *i*PrOH in hexane, 1.0 mL/min, 254 nm UV detector, t_R (minor) = 6.6 min, t_R (major) = 7.3 min.

[α]$_D$^{20} = –1 (c = 0.17, $CHCl_3$).

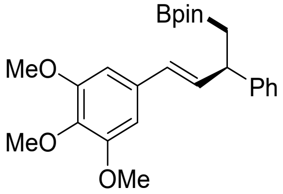

(*R,E*)-4,4,5,5-Tetramethyl-2-(2-phenyl-4-(3,4,5-trimethoxyphenyl)but-3-en-1-yl)-1,3,2-dioxaborolane (17).

The title compound was prepared following the general procedure using styrene (15.6 mg, 0.150 mmol, 1.0 equiv), (*E*)-5-(2-iodovinyl)-1,2,3-trimethoxybenzene (62.4 mg, 0.195 mmol, 1.3 equiv). After purification by column chromatography (using 5% ethyl acetate in petroleum ether), the title compound was isolated in 86% yield (54.7 mg, 94% *ee*) as a white solid.

^{1}H NMR (500 MHz, $CDCl_3$): δ 7.31–7.28 (m, 4H), 7.19 (ddd, *J* = 8.6, 5.7, 3.1 Hz, 1H), 6.55 (s, 2H), 6.37–6.26 (m, 2H), 3.84 (s, 6H), 3.82 (s, 3H), 3.78 (dd, *J* = 14.8, 8.5 Hz, 1H), 1.41–1.35 (m, 2H), 1.15 (s, 12H).

^{13}C NMR (126 MHz, $CDCl_3$): δ 153.35, 145.77, 137.45, 135.33, 133.51, 128.51, 128.37, 127.67, 126.30, 103.30, 83.33, 61.04, 56.17, 44.39, 29.83, 24.90 (d, J = 12.6 Hz).

HRMS (ESI) m/z ([M+H$^+$) calcd for $C_{25}H_{34}BO_5$: 425.2494. Found: 425.2495.

M.p.: 111-112 ºC.

HPLC analysis: CHIRALCEL OD-H column, 5% *i*PrOH in hexane, 0.5 mL/min, 254 nm UV detector, t_R (minor) = 14.2 min, t_R (major) = 15.1 min.

[α]$_D^{20}$ = –3 (c = 0.16, $CHCl_3$).

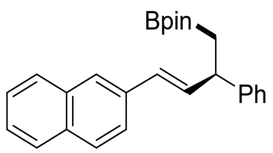

(*R,E*)-4,4,5,5-Tetramethyl-2-(4-(naphthalen-2-yl)-2-phenylbut-3-en-1-yl)-1,3,2-dioxaborolane (18).

The title compound was prepared following the general procedure using styrene (15.6 mg, 0.150 mmol, 1.0 equiv), (*E*)-2-(2-bromovinyl)naphthalene (45.4 mg, 0.195 mmol, 1.3 equiv). After purification by column chromatography (using 1% ethyl acetate in petroleum ether), the title compound was isolated in 84% yield (48.4 mg, 96% *ee*) as a white solid.

^{1}H NMR (500 MHz, $CDCl_3$): δ 7.75 (dd, J = 15.1, 8.8 Hz, 3H), 7.68 (s, 1H), 7.56 (dd, J = 8.6, 1.6 Hz, 1H), 7.45–7.39 (m, 2H), 7.35–7.29 (m, 4H), 7.22–7.18 (m, 1H), 6.64–6.48 (m, 2H), 3.85 (dd, J = 15.5, 7.6 Hz, 1H), 1.48–1.39 (m, 2H), 1.15 (s, 12H).

^{13}C NMR (126 MHz, $CDCl_3$): δ 145.89, 136.23, 135.24, 133.81, 132.86, 128.61, 128.54, 128.12, 127.97, 127.74, 127.63, 126.30, 126.21, 125.83, 125.62, 123.89, 83.35, 44.70, 29.84, 24.91 (d, J = 18.2 Hz).

HRMS (ESI) m/z ([M+H]$^+$) calcd for $C_{26}H_{30}BO_2$: 385.2333. Found: 385.2352.

M.p.: 75-76 ºC.

HPLC analysis: CHIRALCEL OD-H column, 0.1% *i*PrOH in hexane, 0.1 mL/min, 254 nm UV detector, t_R (major) = 13.2 min, t_R (minor) = 14.1 min.

[α]$_D^{20}$ = –3 (c = 0.10, $CHCl_3$).

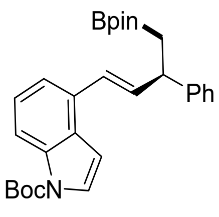

***tert*-Butyl-(*R,E*)-4-(3-phenyl-4-(4,4,5,5-tetramethyl-1,3,2-dioxaborolan-2-yl)but-1-en-1-yl)-1H-indole-1-carboxylate (19)**.

The title compound was prepared following the general procedure using

styrene (15.6 mg, 0.150 mmol, 1.0 equiv), *tert*-butyl (*E*)-4-(2-bromovinyl)-1H-indole-1-carboxylate (62.8 mg, 0.195 mmol, 1.3 equiv). After purification by column chromatography (using 1% ethyl acetate in petroleum ether), the title compound was isolated in 70% yield (49.7 mg, 93% *ee*) as a yellow oil.

^{1}H NMR (500 MHz, $CDCl_3$): δ 8.01 (d, J = 7.7 Hz, 1H), 7.60 (d, J = 3.6 Hz, 1H), 7.35–7.29 (m, 5H), 7.25–7.16 (m, 2H), 6.78 (dd, J = 22.8, 9.8 Hz, 2H), 6.53 (dd, J = 15.8, 7.5 Hz, 1H), 3.87 (q, J = 7.8 Hz, 1H), 1.68 (s, 9H), 1.44 (dt, J = 15.2, 7.3 Hz, 2H), 1.15 (s, 12H).

^{13}C NMR (126 MHz, $CDCl_3$): δ 149.92, 145.95, 137.07, 135.60, 130.35, 128.87, 128.52, 127.59, 126.26, 125.76, 125.55, 124.40, 119.38, 113.81, 105.72, 83.75, 83.33, 44.92, 29.83, 28.32, 24.91 (d, J = 12.6 Hz).

HRMS (ESI) m/z ($[M+H]^+$) calcd for $C_{29}H_{37}BNO_4$: 474.2810. Found: 474.2812.

HPLC analysis: CHIRALCEL OD-H column, 0.5% *i*PrOH in hexane, 0.5 mL/min, 254 nm UV detector, t_R (minor) = 9.5 min, t_R (major) = 10.4 min.

$[\alpha]_D^{20}$ = –9 (c = 0.26, $CHCl_3$).

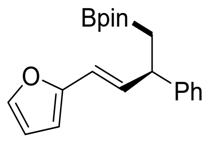

(*R,E*)-2-(4-(Furan-2-yl)-2-phenylbut-3-en-1-yl)-4,4,5,5-tetramethyl-1,3,2-dioxaborolane (20).

The title compound was prepared following the general procedure using styrene (15.6 mg, 0.150 mmol, 1.0 equiv), (*E*)-2-(2-bromovinyl)furan (33.7 mg, 0.195 mmol, 1.3 equiv). After purification by column chromatography (using 1% ethyl acetate in petroleum ether), the title compound was isolated in 84% yield (40.8 mg, 95% *ee*) as a yellow oil.

^{1}H NMR (500 MHz, $CDCl_3$): δ 7.32–7.26 (m, 5H), 7.20–7.15 (m, 1H), 6.39–6.31 (m, 2H), 6.20 (d, J = 15.9 Hz, 1H), 6.13 (d, J = 3.2 Hz, 1H), 3.74 (q, J = 7.5 Hz, 1H), 1.36 (dd, J = 8.1, 3.7 Hz, 2H), 1.14 (s, 12H).

^{13}C NMR (126 MHz, $CDCl_3$): δ 153.29, 145.53, 141.47, 134.87, 128.48, 127.68, 126.29, 117.25, 111.20, 106.69, 83.31, 44.26, 29.84, 24.84 (d, J = 8.6 Hz).

HRMS (ESI) m/z ($[M+H]^+$) calcd for $C_{20}H_{26}BO_3$: 325.1970. Found: 325.1975.

HPLC analysis: CHIRALCEL OD-H column, 0.5% *i*PrOH in hexane, 0.5 mL/min, 254 nm UV

detector, t_R (minor) = 8.9 min, t_R (major) = 9.9 min.

$[\alpha]_D^{20}$ = +8 (c = 0.15, $CHCl_3$).

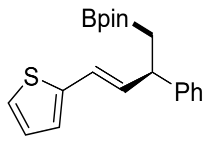

(*R,E*)-4,4,5,5-Tetramethyl-2-(2-phenyl-4-(thiophen-2-yl)but-3-en-1-yl)-1,3,2-dioxaborolane (21).

The title compound was prepared following the general procedure using styrene (15.6 mg, 0.150 mmol, 1.0 equiv), (*E*)-2-(2-bromovinyl)thiophene (36.8 mg, 0.195 mmol, 1.3 equiv). After purification by column chromatography (using 1% ethyl acetate in petroleum ether), the title compound was isolated in 83% yield (42.3 mg, 95% *ee*) as a yellow oil.

^{1}H NMR (500 MHz, $CDCl_3$): δ 7.32–7.26 (m, 4H), 7.22–7.17 (m, 1H), 7.08 (d, J = 5.1 Hz, 1H), 6.91 (dd, J = 5.0, 3.6 Hz, 1H), 6.86 (d, J = 3.3 Hz, 1H), 6.52 (d, J = 15.7 Hz, 1H), 6.24 (dd, J = 15.7, 7.1 Hz, 1H), 3.75 (q, J = 7.6 Hz, 1H), 1.40–1.33 (m, 2H), 1.15 (s, 12H).

^{13}C NMR (126 MHz, $CDCl_3$): δ 145.55, 143.02, 135.69, 128.51, 127.66, 127.29, 126.32, 124.76, 123.48, 121.90, 83.34, 44.31, 29.84, 24.88 (d, J = 14.0 Hz).

HRMS (ESI) m/z ($[M+H^+]$) calcd for $C_{20}H_{26}BO_2S$: 341.1741. Found: 341.1740.

HPLC analysis: CHIRALCEL OD-H column, 0.5% *i*PrOH in hexane, 0.5 mL/min, 254 nm UV detector, t_R (minor) = 9.5 min, t_R (major) = 10.4 min.

$[\alpha]_D^{20}$ = –1 (c = 0.24, $CHCl_3$).

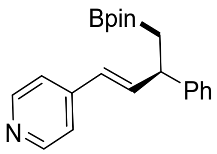

(*R,E*)-4-(3-Phenyl-4-(4,4,5,5-tetramethyl-1,3,2-dioxaborolan-2-yl)but-1-en-1-yl)pyridine (22).

The title compound was prepared following the general procedure using styrene (15.6 mg, 0.150 mmol, 1.0 equiv), (*E*)-4-(2-bromovinyl)pyridine (35.9 mg, 0.195 mmol, 1.3 equiv). After purification by column chromatography (using 1% ethyl acetate in petroleum ether), the title compound was isolated in 68% yield (34.2 mg, 70% *ee*) as a yellow oil.

^{1}H NMR (500 MHz, $CDCl_3$): δ 7.57 (d, J = 8.4 Hz, 2H), 7.27 (dd, J = 12.0, 5.0 Hz, 4H), 7.19–7.16 (m, 1H), 7.05 (d, J = 8.4 Hz, 2H), 6.42–6.28 (m, 2H), 3.75 (dd, J = 15.3, 7.6 Hz, 1H), 1.39–1.33 (m, 2H), 1.12 (s, 12H).

^{13}C NMR (126 MHz, $CDCl_3$): δ 145.54, 137.60, 137.29, 136.77, 128.55, 128.12, 127.50 (d, J = 17.3

Hz), 126.37, 92.09, 83.35, 44.54, 29.84, 24.89 (d, J = 17.0 Hz).

HRMS (ESI) m/z ($[M+H]^+$) calcd for $C_{21}H_{27}BNO_2$: 336.2129. Found: 336.2180.

HPLC analysis: CHIRALCEL OD-H column, 0.5% *i*PrOH in hexane, 0.5 mL/min, 254 nm UV detector, t_R (minor) = 9.6 min, t_R (major) = 10.0 min.

$[\alpha]_D^{20}$ = +1 (c = 0.09, $CHCl_3$).

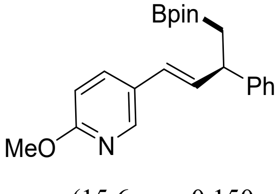

(*R,E*)-2-Methoxy-5-(3-phenyl-4-(4,4,5,5-tetramethyl-1,3,2-dioxaborolan-2-yl)but-1-en-1-yl)pyridine (23).

The title compound was prepared following the general procedure using styrene (15.6 mg, 0.150 mmol, 1.0 equiv), (*E*)-5-(2-bromovinyl)-2-methoxypyridine (41.7 mg, 0.195 mmol, 1.3 equiv). After purification by column chromatography (using 2% ethyl acetate in petroleum ether), the title compound was isolated in 72% yield (39.4 mg, 96% *ee*) as a colorless oil.

^{1}H NMR (500 MHz, $CDCl_3$): δ 8.04 (d, J = 2.3 Hz, 1H), 7.62 (dd, J = 8.7, 2.4 Hz, 1H), 7.32–7.25 (m, 4H), 7.20–7.16 (m, 1H), 6.67 (d, J = 8.6 Hz, 1H), 6.35 (d, J = 15.9 Hz, 1H), 6.27 (dd, J = 15.9, 7.1 Hz, 1H), 3.92 (s, 3H), 3.77 (q, J = 7.6 Hz, 1H), 1.42–1.32 (m, 2H), 1.14 (s, 12H).

^{13}C NMR (126 MHz, $CDCl_3$): δ 163.26, 145.73, 144.99, 135.74, 135.35, 128.55, 127.52, 126.95, 126.34, 124.43, 110.93, 83.34, 53.74, 44.58, 29.83, 24.89 (d, J = 17.3 Hz).

HRMS (ESI) m/z ($[M+H]^+$) calcd for $C_{22}H_{29}BNO_3$: 366.2235. Found: 366.2236.

HPLC analysis: CHIRALCEL OD-H column, 3% *i*PrOH in hexane, 0.5 mL/min, 254 nm UV detector, t_R (minor) = 9.1 min, t_R (major) = 9.6 min.

$[\alpha]_D^{20}$ = –3 (c = 0.32, $CHCl_3$).

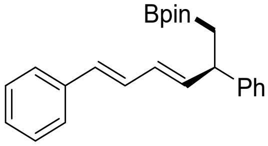

2-((*R,3E,5E*)-2,6-Diphenylhexa-3,5-dien-1-yl)-4,4,5,5-tetramethyl-1,3,2-dioxaborolane (24).

The title compound was prepared following the general procedure using styrene (15.6 mg, 0.150 mmol, 1.0 equiv), (*1E,3E*)-4-bromobuta-1,3-dien-1-yl)benzene (40.8 mg, 0.195 mmol, 1.3 equiv). After purification by column chromatography (using 1% ethyl acetate in petroleum ether), the title compound was isolated in 86% yield (46.4 mg, 86% *ee*) as a yellow oil.

^{1}H NMR (500 MHz, $CDCl_3$): δ 7.37–7.33 (m, 2H), 7.31–7.27 (m, 3H), 7.27–7.25 (m, 3H), 7.20–7.16

(m, 2H), 6.74 (dd, J = 15.6, 10.4 Hz, 1H), 6.45 (d, J = 15.7 Hz, 1H), 6.22 (dd, J = 15.2, 10.4 Hz, 1H), 5.99 (dd, J = 15.1, 7.4 Hz, 1H), 3.71 (q, J = 7.8 Hz, 1H), 1.34 (t, J = 7.6 Hz, 2H), 1.15 (s, 12H).

^{13}C NMR (126 MHz, $CDCl_3$): δ 145.83, 140.41, 137.73, 133.65, 130.89, 129.38, 129.16, 128.67, 128.49, 127.56, 127.28, 126.29, 83.32, 44.42, 29.84, 24.90 (d, J = 15.4 Hz).

HRMS (ESI) m/z ($[M+H]^+$) calcd for $C_{24}H_{30}BO_2$: 361.2333. Found: 361.2332.

HPLC analysis: CHIRALCEL OD-H column, 0.5% *i*PrOH in hexane, 0.5 mL/min, 254 nm UV detector, t_R (minor) = 12.8 min, t_R (major) = 14.1 min.

$[\alpha]_D^{20}$ = –2 (c = 0.19, $CHCl_3$).

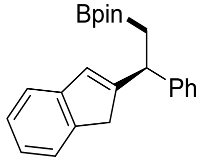

(*R*)-2-(2-(1*H*-Inden-2-yl)-2-phenylethyl)-4,4,5,5-tetramethyl-1,3,2-dioxaborolane (25).

The title compound was prepared following the general procedure using styrene (15.6 mg, 0.150 mmol, 1.0 equiv), 2-bromo-1H-indene (38.0 mg, 0.195 mmol, 1.3 equiv). After purification by column chromatography (using 1% ethyl acetate in petroleum ether), the title compound was isolated in 74% yield (38.4 mg, 98% *ee*) as a colorless oil.

^{1}H NMR (500 MHz, $CDCl_3$): δ 7.27 (dd, J = 7.3, 3.9 Hz, 2H), 7.24 (t, J = 3.6 Hz, 4H), 7.16 (ddd, J = 13.1, 11.8, 5.9 Hz, 2H), 7.06 (t, J = 7.4 Hz, 1H), 6.63 (s, 1H), 4.05 (t, J = 8.2 Hz, 1H), 3.27–3.10 (m, 2H), 1.52 (ddd, J = 59.6, 15.3, 8.3 Hz, 2H), 1.09 (d, J = 5.5 Hz, 12H).

^{13}C NMR (126 MHz, $CDCl_3$): δ 155.19, 145.93, 145.35, 143.57, 128.43, 127.80, 126.30, 126.29, 125.91, 123.92, 123.56, 120.40, 83.31, 43.38, 40.23, 29.84, 24.78 (d, J = 6.8 Hz).

HRMS (ESI) m/z ($[M+H]^+$) calcd for $C_{23}H_{28}BO_2$: 347.2177. Found: 347.2179.

HPLC analysis: CHIRALCEL AD-H column, 0.5% *i*PrOH in hexane, 1.0 mL/min, 254 nm UV detector, t_R (major) = 5.0 min, t_R (minor) = 5.5 min.

$[\alpha]_D^{20}$ = +2 (c = 0.22, $CHCl_3$).

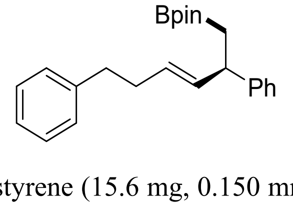

(*R,E*)-2-(2,6-Diphenylhex-3-en-1-yl)-4,4,5,5-tetramethyl-1,3,2-dioxaborolane (26).

The title compound was prepared following the general procedure using styrene (15.6 mg, 0.150 mmol, 1.0 equiv), (*E*)-(4-bromobut-3-en-1-yl)benzene (41.1 mg, 0.195 mmol,

1.3 equiv). After purification by column chromatography (using 1% ethyl acetate in petroleum ether), the title compound was isolated in 70% yield (38.0 mg, 31% *ee*) as a colorless oil.

^{1}H NMR (500 MHz, $CDCl_3$): δ 7.27 (d, J = 6.2 Hz, 4H), 7.22–7.15 (m, 6H), 5.65 (dd, J = 15.3, 7.1 Hz, 1H), 5.54–5.49 (m, 1H), 3.58 (q, J = 7.7 Hz, 1H), 2.73–2.64 (m, 2H), 2.32 (dd, J = 15.2, 7.0 Hz, 2H), 1.28–1.24 (m, 2H), 1.16 (d, J = 1.8 Hz, 12H).

^{13}C NMR (126 MHz, $CDCl_3$): δ 146.57, 142.22, 136.25, 128.60, 128.38, 128.33, 128.07, 127.50, 125.97, 125.83, 83.19, 44.11, 36.07, 34.49, 29.84, 24.87 (d, J = 13.6 Hz).

HRMS (ESI) m/z ($[M+H]^+$) calcd for $C_{24}H_{32}BO_2$: 363.2490. Found: 363.2492.

HPLC analysis: CHIRALCEL AS-H column, 0.5% *i*PrOH in hexane, 1.0 mL/min, 254 nm UV detector, t_R (major) = 3.5 min, t_R (minor) = 4.4 min.

$[\alpha]_D^{20}$ = +2 (c = 0.21, $CHCl_3$).

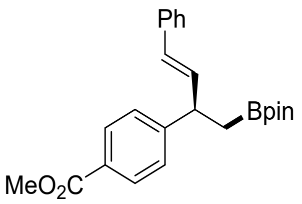

Methyl-(*R,E*)-4-(4-phenyl-1-(4,4,5,5-tetramethyl-1,3,2-dioxaborolan-2-yl)but-3-en-2-yl)benzoate (27).

The title compound was prepared following the general procedure using methyl 4-vinylbenzoate (24.3 mg, 0.150 mmol, 1.0 equiv), (*E*)-(2-bromovinyl)benzene (35.6 mg, 0.195 mmol, 1.3 equiv). After purification by column chromatography (using 1% ethyl acetate in petroleum ether), the title compound was isolated in 89% yield (52.3 mg, 97% *ee*) as a white solid.

^{1}H NMR (500 MHz, $CDCl_3$): δ 7.96 (d, J = 8.3 Hz, 2H), 7.36 (d, J = 8.3 Hz, 2H), 7.32 (d, J = 7.2 Hz, 2H), 7.27 (dd, J = 10.6, 4.4 Hz, 2H), 7.18 (t, J = 7.2 Hz, 1H), 6.42 (d, J = 15.9 Hz, 1H), 6.34 (dd, J = 15.8, 7.0 Hz, 1H), 3.90 (s, 3H), 3.84 (q, J = 7.6 Hz, 1H), 1.45–1.34 (m, 2H), 1.13 (d, J = 4.3 Hz, 12H).

^{13}C NMR (126 MHz, $CDCl_3$): δ 167.28, 151.34, 137.47, 134.78, 129.90, 129.13, 128.62, 128.20, 127.69, 127.28, 126.33, 83.44, 52.12, 44.54, 29.84, 24.89 (d, J = 10.9 Hz).

HRMS (ESI) m/z ($[M+H]^+$) calcd for $C_{24}H_{30}BO_4$: 393.2232. Found: 393.2233.

M.p.: 119-120 ºC.

HPLC analysis: CHIRALCEL OD-H column, 0.5% *i*PrOH in hexane, 1.0 mL/min, 254 nm UV detector, t_R (major) = 8.4 min, t_R (minor) = 9.5 min.

[α]D20 = +6 (c = 0.16, $CHCl_3$).

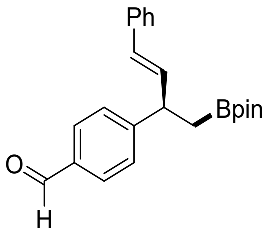

(*R,E*)-4-(4-Phenyl-1-(4,4,5,5-tetramethyl-1,3,2-dioxaborolan-2-yl)but-3-en-2-yl)benzaldehyde (28).

The title compound was prepared following the general procedure using 4-ethenylbenzaldehyde (19.8 mg, 0.150 mmol, 1.0 equiv), (*E*)-(2-bromovinyl)benzene (35.6 mg, 0.195 mmol, 1.3 equiv). After purification by column chromatography (using 3% ethyl acetate in petroleum ether), the title compound was isolated in 72% yield (39.1 mg, 95% *ee*) as a colorless oil.

^{1}H NMR (500 MHz, $CDCl_3$): δ 9.97 (s, 1H), 7.82 (d, *J* = 8.2 Hz, 2H), 7.46 (d, *J* = 8.1 Hz, 2H), 7.33 (d, *J* = 7.3 Hz, 2H), 7.28 (d, *J* = 7.4 Hz, 2H), 7.20 (t, *J* = 7.2 Hz, 1H), 6.50–6.27 (m, 2H), 3.87 (q, *J* = 7.7 Hz, 1H), 1.40 (dd, *J* = 14.2, 6.1 Hz, 2H), 1.14 (d, *J* = 4.1 Hz, 12H).

^{13}C NMR (126 MHz, $CDCl_3$): δ 192.19, 153.30, 137.35, 134.89, 134.36, 130.17, 129.43, 128.65, 128.36, 127.40, 126.35, 83.50, 44.73, 29.84, 24.89 (d, *J* = 11.6 Hz).

HRMS (ESI) m/z ($[M+H]^+$) calcd for $C_{23}H_{28}BO_3$: 363.2126. Found: 363.2125.

HPLC analysis: CHIRALCEL OD-H column, 5% *i*PrOH in hexane, 0.5 mL/min, 254 nm UV detector, t_R (major) = 12.1 min, t_R (minor) = 12.8 min.

[α]D20 = +6 (c = 0.09, $CHCl_3$).

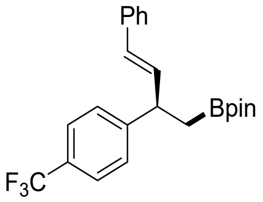

(*R,E*)-4,4,5,5-Tetramethyl-2-(4-phenyl-2-(4-(trifluoromethyl)phenyl)but-3-en-1-yl)-1,3,2-dioxaborolane (29).

The title compound was prepared following the general procedure using 4-(trifloromethyl)styrene (25.8 mg, 0.150 mmol, 1.0 equiv), (*E*)-(2-bromovinyl)benzene (35.6 mg, 0.195 mmol, 1.3 equiv). After purification by column chromatography (using 1% ethyl acetate in petroleum ether), the title compound was isolated in 90% yield (54.3 mg, 99% *ee*) as a colorless oil.

^{1}H NMR (500 MHz, $CDCl_3$): δ 7.55 (d, *J* = 8.1 Hz, 2H), 7.40 (d, *J* = 8.1 Hz, 2H), 7.33 (d, *J* = 7.2 Hz, 2H), 7.29 (d, *J* = 7.4 Hz, 2H), 7.20 (t, *J* = 7.2 Hz, 1H), 6.37 (dt, *J* = 15.8, 11.5 Hz, 2H), 3.85 (q, *J* = 7.6 Hz, 1H), 1.39 (dd, *J* = 18.5, 8.0 Hz, 2H), 1.14 (d, *J* = 2.1 Hz, 12H).

^{13}C NMR (126 MHz, $CDCl_3$): δ 150.05, 137.38, 134.60, 129.27, 128.64, 127.98, 127.36, 126.34, 125.44 (dd, J = 7.3, 3.6 Hz), 112.20, 100.12, 83.48, 44.36, 29.85, 24.88 (d, J = 14.6 Hz).

HRMS (ESI) m/z ($[M+Na]^+$) calcd for $C_{23}H_{26}BF_3NaO_2$: 425.1870. Found: 425.1889.

HPLC analysis: CHIRALCEL OD-H column, 0.5% *i*PrOH in hexane, 0.5 mL/min, 254 nm UV detector, t_R (minor) = 9.8 min, t_R (major) = 10.4 min.

$[\alpha]_D^{20}$ = –1 (c = 0.26, $CHCl_3$).

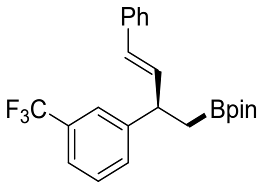

(*R,E*)-4,4,5,5-Tetramethyl-2-(4-phenyl-2-(3-(trifluoromethyl)phenyl)but-3-en-1-yl)-1,3,2-dioxaborolane (30).

The title compound was prepared following the general procedure using 3-(trifluoromethyl)styrene (25.8 mg, 0.150 mmol, 1.0 equiv), (*E*)-(2-bromovinyl)benzene (35.6 mg, 0.195 mmol, 1.3 equiv). After purification by column chromatography (using 1% ethyl acetate in petroleum ether), the title compound was isolated in 84% yield (50.6 mg, 91% *ee*) as a colorless oil.

^{1}H NMR (500 MHz, $CDCl_3$): δ 7.54 (s, 1H), 7.44 (dd, J = 12.4, 7.3 Hz, 2H), 7.38 (t, J = 5.4 Hz, 1H), 7.33–7.31 (m, 2H), 7.26 (t, J = 7.6 Hz, 2H), 7.20–7.16 (m, 1H), 6.36 (dt, J = 15.8, 11.5 Hz, 2H), 3.83 (q, J = 7.6 Hz, 1H), 1.42–1.33 (m, 2H), 1.11 (d, J = 3.3 Hz, 12H).

^{13}C NMR (126 MHz, $CDCl_3$): δ 146.85, 137.40, 134.73, 132.96, 131.09, 129.19, 128.91, 128.63, 127.33, 126.36, 124.53 (q, J = 3.8 Hz), 123.15 (q, J = 3.7 Hz), 83.45, 44.36, 29.84, 24.84 (d, J = 13.4 Hz).

HRMS (ESI) m/z ($[M+K]^+$) calcd for $C_{23}H_{26}BF_3KO_2$: 441.1610. Found: 441.1623.

HPLC analysis: CHIRALCEL AD-H column, 0.5% *i*PrOH in hexane, 1.0 mL/min, 254 nm UV detector, t_R (minor) = 4.2 min, t_R (major) = 4.8 min.

$[\alpha]_D^{20}$ = –1 (c = 0.15, $CHCl_3$).

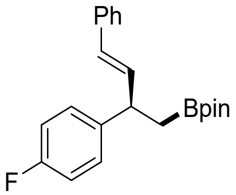

(*R,E*)-2-(2-(4-Fluorophenyl)-4-phenylbut-3-en-1-yl)-4,4,5,5-tetramethyl-1,3,2-dioxaborolane (31).

The title compound was prepared following the general procedure using 4-fluorostyrene (18.3 mg, 0.150 mmol, 1.0 equiv), (*E*)-(2-bromovinyl)benzene (35.6 mg, 0.195 mmol, 1.3 equiv). After purification by column chromatography (using 1% ethyl

acetate in petroleum ether), the title compound was isolated in 88% yield (46.5 mg, 93% *ee*) as a colorless oil.

^{1}H NMR (500 MHz, $CDCl_3$): δ 7.33 (d, J = 7.4 Hz, 2H), 7.31–7.27 (m, 2H), 7.26–7.22 (m, 2H), 7.19 (t, J = 7.2 Hz, 1H), 6.98 (t, J = 8.7 Hz, 2H), 6.36 (dt, J = 15.8, 11.3 Hz, 2H), 3.78 (dd, J = 14.8, 7.7 Hz, 1H), 1.38–1.33 (m, 2H), 1.15 (d, J = 2.6 Hz, 12H).

^{13}C NMR (126 MHz, $CDCl_3$): δ 162.47, 160.53, 141.50 (d, J = 3.1 Hz), 137.60, 135.61, 129.02 (d, J = 7.8 Hz), 128.56 (d, J = 6.8 Hz), 127.17, 126.30, 115.15 (d, J = 10.7 Hz), 83.36, 43.74, 29.84, 24.88 (d, J = 10.7 Hz).

HRMS (ESI) m/z ($[M+H]^+$) calcd for $C_{22}H_{27}BFO_2$: 353.2083. Found: 353.2084.

HPLC analysis: CHIRALCEL OJ-H column, 0.1% *i*PrOH in hexane, 0.5 mL/min, 254 nm UV detector, t_R (minor) = 14.0 min, t_R (major) = 17.2 min.

$[\alpha]_D^{20}$ = –2 (c = 0.27, $CHCl_3$).

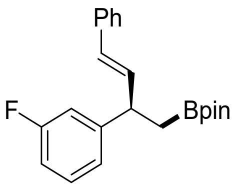

(*R,E*)-2-(2-(3-Fluorophenyl)-4-phenylbut-3-en-1-yl)-4,4,5,5-tetramethyl-1,3,2-dioxaborolane (32).

The title compound was prepared following the general procedure using 3-fluorostyrene (18.3 mg, 0.150 mmol, 1.0 equiv), (*E*)-(2-bromovinyl)benzene (35.6 mg, 0.195 mmol, 1.3 equiv). After purification by column chromatography (using 1% ethyl acetate in petroleum ether), the title compound was isolated in 84% yield (44.4 mg, 96% *ee*) as a colorless oil.

^{1}H NMR (500 MHz, $CDCl_3$): δ 7.34–7.31 (m, 2H), 7.28 (t, J = 6.7 Hz, 2H), 7.23 (d, J = 7.9 Hz, 1H), 7.19 (dd, J = 10.3, 4.3 Hz, 1H), 7.07 (d, J = 7.7 Hz, 1H), 7.02–6.97 (m, 1H), 6.87 (ddd, J = 8.2, 2.5, 1.7 Hz, 1H), 6.37 (dt, J = 15.8, 11.5 Hz, 2H), 3.78 (q, J = 7.7 Hz, 1H), 1.39–1.35 (m, 2H), 1.15 (d, J = 2.2 Hz, 12H).

^{13}C NMR (126 MHz, $CDCl_3$): δ 164.03, 162.08, 148.61 (d, J = 6.6 Hz), 137.52, 134.95, 129.87 (d, J = 8.2 Hz), 128.61, 127.24, 126.34, 123.24 (d, J = 2.7 Hz), 114.52 (d, J = 10.1 Hz), 113.06 (d, J = 10.7 Hz), 83.41, 44.27, 29.84, 24.89 (d, J = 14.2 Hz).

HRMS (ESI) m/z ($[M+H]^+$) calcd for $C_{22}H_{27}BFO_2$: 353.2083. Found: 353.2081.

HPLC analysis: CHIRALCEL OJ-3 column, 0.5% *i*PrOH in hexane, 0.1 mL/min, 254 nm UV

detector, t_R (major) = 58.7 min, t_R (minor) = 62.2 min.

[α]$_D^{20}$ = –4 (c = 0.24, $CHCl_3$).

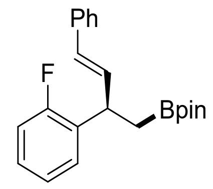

(*R,E*)-2-(2-(2-Fluorophenyl)-4-phenylbut-3-en-1-yl)-4,4,5,5-tetramethyl-1,3,2-dioxaborolane (33).

The title compound was prepared following the general procedure using 2-fluorostyrene (18.3 mg, 0.150 mmol, 1.0 equiv), (*E*)-(2-bromovinyl)benzene (35.6 mg, 0.195 mmol, 1.3 equiv). After purification by column chromatography (using 1% ethyl acetate in petroleum ether), the title compound was isolated in 86% yield (45.4 mg, 92% *ee*) as a pale yellow solid.

^{1}H NMR (500 MHz, $CDCl_3$): δ 7.33 (d, J = 7.5 Hz, 2H), 7.27 (dd, J = 10.6, 3.1 Hz, 3H), 7.19–7.14 (m, 2H), 7.08 (t, J = 7.5 Hz, 1H), 6.99 (dd, J = 14.0, 5.6 Hz, 1H), 6.48–6.34 (m, 2H), 4.10 (dd, J = 14.9, 7.7 Hz, 1H), 1.44–1.37 (m, 2H), 1.13 (d, J = 6.2 Hz, 12H).

^{13}C NMR (126 MHz, $CDCl_3$): δ 137.65, 134.19, 129.00, 128.82, 128.56, 127.73, 127.14, 126.52, 126.33, 124.14, 115.63, 115.45, 83.34, 37.78, 29.84, 24.86 (d, J = 14.1 Hz).

HRMS (ESI) m/z ([M+H]$^+$) calcd for $C_{22}H_{27}BFO_2$: 353.2083. Found: 353.2080.

M.p.: 90-91 ºC.

HPLC analysis: CHIRALCEL OD-H column, 0.5% *i*PrOH in hexane, 0.3 mL/min, 254 nm UV detector, t_R (minor) = 14.7 min, t_R (major) = 15.5 min.

[α]$_D^{20}$ = +2 (c = 0.28, $CHCl_3$).

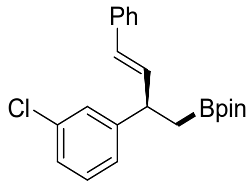

(*R,E*)-2-(2-(3-Clorophenyl)-4-phenylbut-3-en-1-yl)-4,4,5,5-tetramethyl-1,3,2-dioxaborolane (34).

The title compound was prepared following the general procedure using 3-chlorostyrene (20.8 mg, 0.150 mmol, 1.0 equiv), (*E*)-(2-bromovinyl)benzene (35.6 mg, 0.195 mmol, 1.3 equiv). After purification by column chromatography (using 1% ethyl acetate in petroleum ether), the title compound was isolated in 83% yield (45.9 mg, 68% *ee*) as a colorless oil.

^{1}H NMR (500 MHz, $CDCl_3$): δ 7.33 (d, J = 7.3 Hz, 2H), 7.31–7.27 (m, 3H), 7.23–7.14 (m, 4H), 6.37

(dt, J = 15.8, 11.5 Hz, 2H), 3.76 (q, J = 7.6 Hz, 1H), 1.40–1.32 (m, 2H), 1.15 (d, J = 1.9 Hz, 12H).

^{13}C NMR (126 MHz, $CDCl_3$): δ 148.02, 137.47, 134.81, 134.19, 129.74, 129.02, 128.60, 127.89, 127.25, 126.39, 126.34, 125.81, 83.41, 44.22, 29.83, 24.88 (d, J = 11.8 Hz).

HRMS (ESI) m/z ($[M+H]^+$) calcd for $C_{22}H_{27}BClO_2$: 369.1787. Found: 369.1788.

HPLC analysis: CHIRALCEL AD-H column, 10% EtOH in hexane, 0.5 mL/min, 254 nm UV detector, t_R (minor) = 7.7 min, t_R (major) = 8.2 min.

$[\alpha]_D^{20}$ = +2 (c = 0.26 $CHCl_3$).

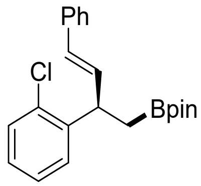

(*R,E*)-2-(2-(2-Chlorophenyl)-4-phenylbut-3-en-1-yl)-4,4,5,5-tetramethyl-1,3,2-dioxaborolane (35).

The title compound was prepared following the general procedure using 2-chlorostyrene (20.8 mg, 0.150 mmol, 1.0 equiv), (*E*)-(2-bromovinyl)benzene (35.6 mg, 0.195 mmol, 1.3 equiv). After purification by column chromatography (using 1% ethyl acetate in petroleum ether), the title compound was isolated in 80% yield (44.2 mg, 77% *ee*) as a yellow solid.

^{1}H NMR (500 MHz, $CDCl_3$): δ 7.36–7.33 (m, 4H), 7.29 (d, J = 6.2 Hz, 2H), 7.22 (d, J = 7.6 Hz, 1H), 7.18 (t, J = 7.3 Hz, 1H), 7.12 (td, J = 7.7, 1.6 Hz, 1H), 6.44 (d, J = 16.0 Hz, 1H), 6.35 (dd, J = 15.9, 6.8 Hz, 1H), 4.34 (q, J = 7.6 Hz, 1H), 1.39 (s, 1H), 1.34 (d, J = 10.6 Hz, 1H), 1.14 (d, J = 8.6 Hz, 12H).

^{13}C NMR (126 MHz, $CDCl_3$): δ 143.08, 137.65, 134.00, 133.83, 132.95, 129.67, 129.17, 128.79, 128.56, 127.36, 127.04, 126.32, 83.34, 40.30, 29.84, 24.83 (d, J = 13.6 Hz).

HRMS (ESI) m/z ($[M+H]^+$) calcd for $C_{22}H_{27}BClO_2$: 369.1787. Found: 369.1788.

M.p.: 81-82 ºC.

HPLC analysis: CHIRALCEL OD-H column, 0.5% *i*PrOH in hexane, 0.5 mL/min, 254 nm UV detector, t_R (minor) = 9.9 min, t_R (major) = 12.2 min.

$[\alpha]_D^{20}$ = +24 (c = 0.18, $CHCl_3$).

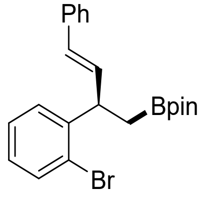

(*R,E*)-2-(2-(2-Bromophenyl)-4-phenylbut-3-en-1-yl)-4,4,5,5-tetramethyl-

1,3,2-dioxaborolane (36).

The title compound was prepared following the general procedure using 2-bromostyrene (27.5 mg, 0.150 mmol, 1.0 equiv), (*E*)-(2-bromovinyl)benzene (35.6 mg, 0.195 mmol, 1.3 equiv). After purification by column chromatography (using 1% ethyl acetate in petroleum ether), the title compound was isolated in 60% yield (37.2 mg, 57% *ee*) as a yellow solid.

^{1}H NMR (500 MHz, $CDCl_3$): δ 7.54 (dd, J = 8.0, 1.1 Hz, 1H), 7.35–7.32 (m, 3H), 7.28 (d, J = 7.6 Hz, 3H), 7.20–7.18 (m, 1H), 7.05 (td, J = 7.9, 1.7 Hz, 1H), 6.44 (d, J = 16.0 Hz, 1H), 6.34 (dd, J = 15.9, 6.8 Hz, 1H), 4.31 (q, J = 7.5 Hz, 1H), 1.33 (dd, J = 10.4, 6.4 Hz, 2H), 1.14 (d, J = 8.0 Hz, 12H).

^{13}C NMR (126 MHz, $CDCl_3$): δ 144.70, 137.65, 134.05, 132.99, 129.18, 128.67, 128.56, 127.70, 127.15, 126.51, 126.33, 124.70, 83.35, 42.90, 29.84, 24.83 (d, J = 11.4 Hz).

HRMS (ESI) m/z ($[M+H]^+$) calcd for $C_{22}H_{27}BBrO_2$: 413.1282. Found: 413.1280.

M.p.: 63-64 ºC.

HPLC analysis: CHIRALCEL OD-H column, 0.5% *i*PrOH in hexane, 0.5 mL/min, 254 nm UV detector, t_R (minor) = 10.3 min, t_R (major) = 14.2 min.

$[\alpha]_D^{20}$ = +25 (c = 0.20, $CHCl_3$).

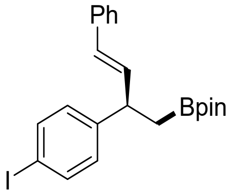

(*R,E*)-2-(2-(4-Iodophenyl)-4-phenylbut-3-en-1-yl)-4,4,5,5-tetramethyl-1,3,2-dioxaborolane (37).

The title compound was prepared following the general procedure using 4-iodostyrene (34.5 mg, 0.150 mmol, 1.0 equiv), (*E*)-(2-bromovinyl)benzene (35.6 mg, 0.195 mmol, 1.3 equiv). After purification by column chromatography (using 1% ethyl acetate in petroleum ether), the title compound was isolated in 62% yield (42.8 mg, 46% *ee*) as a colorless oil.

^{1}H NMR (500 MHz, $CDCl_3$): δ 7.57 (d, J = 8.3 Hz, 1H), 7.37 (d, J = 8.4 Hz, 1H), 7.28 (d, J = 7.2 Hz, 2H), 7.25–7.22 (m, 2H), 7.14 (dd, J = 14.0, 7.7 Hz, 2H), 7.01 (d, J = 8.3 Hz, 1H), 6.41–6.24 (m, 2H), 3.70 (dd, J = 15.6, 7.8 Hz, 1H), 1.37–1.29 (m, 2H), 1.12 (d, J = 2.4 Hz, 12H).

^{13}C NMR (126 MHz, $CDCl_3$): δ 145.63, 137.50, 134.98, 131.51, 129.77, 129.43, 128.90, 128.60, 127.23, 126.30, 83.41, 44.01, 29.83, 24.89 (d, J = 13.1 Hz).

HRMS (ESI) m/z ($[M+H]^+$) calcd for $C_{22}H_{27}BIO_2$: 461.1143. Found: 461.1140.

HPLC analysis: CHIRALCEL OJ-3 column, 5% EtOH in hexane, 0.3 mL/min, 254 nm UV detector, t_R (minor) = 14.7 min, t_R (major) = 15.6 min.

$[\alpha]_D^{20}$ = +2 (c = 0.26 $CHCl_3$).

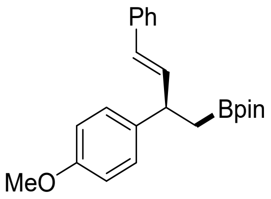

(*R,E*)-2-(2-(4-Methoxyphenyl)-4-phenylbut-3-en-1-yl)-4,4,5,5-tetramethyl-1,3,2-dioxaborolane (38).

The title compound was prepared following the general procedure using 4-methoxystyrene (20.1 mg, 0.150 mmol, 1.0 equiv), (*E*)-(2-bromovinyl)benzene (35.6 mg, 0.195 mmol, 1.3 equiv). After purification by column chromatography (using 1% ethyl acetate in petroleum ether), the title compound was isolated in 92% yield (50.2 mg, 99% *ee*) as a colorless oil.

^{1}H NMR (500 MHz, $CDCl_3$): δ 7.32 (d, J = 7.2 Hz, 2H), 7.26 (t, J = 7.6 Hz, 2H), 7.20 (d, J = 8.6 Hz, 2H), 7.16 (t, J = 7.3 Hz, 1H), 6.83 (d, J = 8.7 Hz, 2H), 6.43–6.31 (m, 2H), 3.78 (s, 3H), 3.74 (dd, J = 14.3, 7.9 Hz, 1H), 1.37–1.32 (m, 2H), 1.14 (s, 12H).

^{13}C NMR (126 MHz, $CDCl_3$): δ 158.08, 138.04, 137.81, 136.13, 128.54, 128.53, 128.14, 127.00, 126.29, 113.86, 83.30, 55.42, 43.65, 29.84, 24.91 (d, J = 13.4 Hz).

HRMS (ESI) m/z ($[M+H]^+$) calcd for $C_{23}H_{30}BO_3$: 365.2283. Found: 365.2280.

HPLC analysis: CHIRALCEL OJ-H column, 0.5% *i*PrOH in hexane, 1.0 mL/min, 254 nm UV detector, t_R (major) = 8.3 min, t_R (minor) = 9.5 min.

$[\alpha]_D^{20}$ = +14 (c = 0.09, $CHCl_3$).

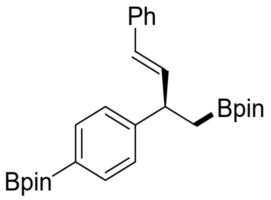

(*R,E*)-4,4,5,5-Tetramethyl-2-(4-(4-phenyl-1-(4,4,5,5-tetramethyl-1,3,2-dioxaborolan-2-yl)but-3-en-2-yl)phenyl)-1,3,2-dioxaborolane (39).

The title compound was prepared following the general procedure using 4,4,5,5-tetramethyl-2-(4-vinylphenyl)-1,3,2-dioxaborolane (34.5 mg, 0.150 mmol, 1.0 equiv), (*E*)-(2-bromovinyl)benzene (35.6 mg, 0.195 mmol, 1.3 equiv). After purification by column chromatography (using 1% ethyl acetate in petroleum ether), the title compound was isolated in 91% yield (62.8 mg, 98% *ee*) as a white solid.

<u>^{1}H NMR</u> (500 MHz, $CDCl_3$): δ 7.74 (d, J = 8.0 Hz, 2H), 7.31 (t, J = 8.0 Hz, 4H), 7.26 (s, 2H), 7.17 (t, J = 7.2 Hz, 1H), 6.38 (dt, J = 15.8, 11.3 Hz, 2H), 3.80 (dd, J = 15.3, 7.2 Hz, 1H), 1.39 (dd, J = 14.6, 5.6 Hz, 2H), 1.33 (s, 12H), 1.15 (d, J = 1.8 Hz, 12H).

<u>^{13}C NMR</u> (126 MHz, $CDCl_3$): δ 149.32, 137.73, 135.39, 135.26, 135.10, 128.70, 128.54, 127.06, 127.04, 126.31, 83.76, 83.34, 44.65, 29.84, 24.97, 24.93 (d, J = 17.9 Hz).

<u>HRMS</u> (ESI) m/z ($[M+H]^+$) calcd for $C_{28}H_{39}B_2O_4$: 461.3029. Found: 461.3027.

<u>M.p.</u>: 162-163 ºC.

<u>HPLC analysis</u>: CHIRALCEL OD-H column, 0.5% *i*PrOH in hexane, 0.25 mL/min, 254 nm UV detector, t_R (minor) = 18.1 min, t_R (major) = 18.5 min.

$[\alpha]_D^{20}$ = +2 (c = 0.28, $CHCl_3$).

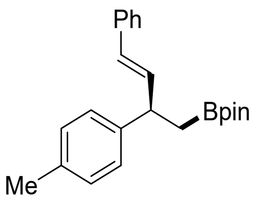

(*R,E*)-4,4,5,5-Tetramethyl-2-(4-phenyl-2-(*p*-tolyl)but-3-en-1-yl)-1,3,2-dioxaborolane (40).

The title compound was prepared following the general procedure using 4-methylstyrene (17.7 mg, 0.150 mmol, 1.0 equiv), (*E*)-(2-bromovinyl)benzene (35.6 mg, 0.195 mmol, 1.3 equiv). After purification by column chromatography (using 1% ethyl acetate in petroleum ether), the title compound was isolated in 91% yield (47.5 mg, 95% *ee*) as a colorless oil.

<u>^{1}H NMR</u> (500 MHz, $CDCl_3$): δ 7.33 (d, J = 7.3 Hz, 2H), 7.27 (dd, J = 9.6, 5.7 Hz, 2H), 7.18 (t, J = 8.5 Hz, 3H), 7.11 (d, J = 7.8 Hz, 2H), 6.39 (dt, J = 15.8, 11.3 Hz, 2H), 3.76 (dd, J = 15.6, 7.2 Hz, 1H), 2.32 (s, 3H), 1.41–1.35 (m, 2H), 1.16 (s, 12H).

<u>^{13}C NMR</u> (126 MHz, $CDCl_3$): δ 142.93, 137.82, 135.92, 135.67, 129.16, 128.52, 128.29, 127.43, 126.98, 126.29, 83.29, 44.08, 29.84, 24.91 (d, J = 18.2 Hz), 21.13.

<u>HRMS</u> (ESI) m/z ($[M+H]^+$) calcd for $C_{23}H_{30}BO_2$: 349.2333. Found: 349.2329.

<u>HPLC analysis</u>: CHIRALCEL OJ-H column, 0.5% *i*PrOH in hexane, 0.1 mL/min, 254 nm UV detector, t_R (minor) = 47.6 min, t_R (major) = 52.7 min.

$[\alpha]_D^{20}$ = –3 (c = 0.25, $CHCl_3$).

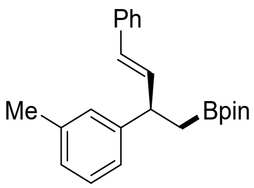

(*R,E*)-4,4,5,5-Tetramethyl-2-(4-phenyl-2-(*m*-tolyl)but-3-en-1-yl)-1,3,2-

dioxaborolane (41).

The title compound was prepared following the general procedure using 3-methylstyrene (17.7 mg, 0.150 mmol, 1.0 equiv), (*E*)-(2-bromovinyl)benzene (35.6 mg, 0.195 mmol, 1.3 equiv). After purification by column chromatography (using 1% ethyl acetate in petroleum ether), the title compound was isolated in 86% yield (44.9 mg, 94% *ee*) as a colorless oil.

^{1}H NMR (500 MHz, $CDCl_3$): δ 7.35–7.32 (m, 2H), 7.28–7.24 (m, 2H), 7.17 (dt, J = 7.2, 2.7 Hz, 2H), 7.08 (d, J = 7.9 Hz, 2H), 6.99 (d, J = 7.5 Hz, 1H), 6.47–6.32 (m, 2H), 3.75 (dd, J = 15.2, 7.5 Hz, 1H), 2.32 (s, 3H), 1.39–1.32 (m, 2H), 1.14 (s, 12H).

^{13}C NMR (150 MHz, $CDCl_3$): δ 145.87, 137.87 (d, J = 16.0 Hz), 135.82, 132.95, 129.38, 128.79, 128.53, 128.38, 127.01, 126.51, 126.31, 124.59, 83.27, 44.45, 29.84, 24.89 (d, J = 16.5 Hz), 21.61.

HRMS (ESI) m/z ($[M+H]^+$) calcd for $C_{23}H_{30}BO_2$: 349.2333. Found: 349.2331.

HPLC analysis: CHIRALCEL OD-H column, 0.5% *i*PrOH in hexane, 0.3 mL/min, 254 nm UV detector, t_R (minor) = 13.9 min, t_R (major) = 14.6 min.

$[\alpha]_D^{20}$ = –2 (c = 0.24, $CHCl_3$).

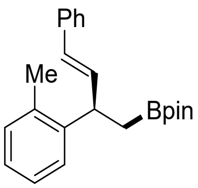

(*R,E*)-4,4,5,5-Tetramethyl-2-(4-phenyl-2-(*o*-tolyl)but-3-en-1-yl)-1,3,2-dioxaborolane (42).

The title compound was prepared following the general procedure using 2-methylstyrene (17.7 mg, 0.150 mmol, 1.0 equiv), (*E*)-(2-bromovinyl)benzene (35.6 mg, 0.195 mmol, 1.3 equiv). After purification by column chromatography (using 1% ethyl acetate in petroleum ether), the title compound was isolated in 83% yield (43.3 mg, 84% *ee*) as a colorless oil.

^{1}H NMR (500 MHz, $CDCl_3$): δ 7.32 (dd, J = 8.3, 1.1 Hz, 2H), 7.28–7.24 (m, 3H), 7.20–7.16 (m, 2H), 7.14–7.08 (m, 2H), 6.38–6.28 (m, 2H), 4.02 (td, J = 8.1, 5.4 Hz, 1H), 2.42 (s, 3H), 1.39 (dd, J = 8.0, 5.3 Hz, 2H), 1.12 (d, J = 11.0 Hz, 12H).

^{13}C NMR (126 MHz, $CDCl_3$): δ 143.60, 137.81, 135.95, 135.52, 132.96, 130.33, 128.53, 128.28, 126.99, 126.68, 126.25, 126.04, 83.25, 39.92, 31.58, 24.82 (d, J = 16.1 Hz), 19.84.

HRMS (ESI) m/z ($[M+H]^+$) calcd for $C_{23}H_{30}BO_2$: 349.2333. Found: 349.2335.

HPLC analysis: CHIRALCEL OD-H column, 0.5% *i*PrOH in hexane, 0.5 mL/min, 254 nm UV detector, t_R (minor) = 8.5 min, t_R (major) = 10.5 min.

[α]$_D$^{20} = –1 (c = 0.21, $CHCl_3$).

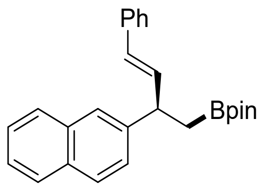

(*R,E*)-4,4,5,5-Tetramethyl-2-(2-(naphthalen-2-yl)-4-phenylbut-3-en-1-yl)-1,3,2-dioxaborolane (43).

The title compound was prepared following the general procedure using 2-vinylnaphthalene (23.1 mg, 0.150 mmol, 1.0 equiv), (*E*)-(2-bromovinyl)benzene (35.6 mg, 0.195 mmol, 1.3 equiv). After purification by column chromatography (using 1% ethyl acetate in petroleum ether), the title compound was isolated in 85% yield (48.9 mg, 92% *ee*) as a yellow solid.

^{1}H NMR (500 MHz, $CDCl_3$): δ 7.80 (dd, *J* = 12.6, 4.7 Hz, 3H), 7.74 (s, 1H), 7.48–7.41 (m, 3H), 7.37–7.33 (m, 2H), 7.28 (t, *J* = 7.7 Hz, 2H), 7.19 (dd, *J* = 10.4, 4.2 Hz, 1H), 6.52–6.43 (m, 2H), 3.98 (dd, *J* = 12.7, 7.9 Hz, 1H), 1.55–1.45 (m, 2H), 1.14 (s, 12H).

^{13}C NMR (126 MHz, $CDCl_3$): δ 143.35, 137.73, 135.58, 133.73, 132.37, 128.78, 128.58, 128.07, 127.81, 127.69, 127.11, 126.63, 126.33, 125.95, 125.56, 125.36, 83.35, 44.55, 29.84, 24.90 (d, *J* = 12.8 Hz).

HRMS (ESI) m/z ($[M+H]^+$) calcd for $C_{26}H_{30}BO_2$: 385.2333. Found: 385.2331.

M.p.: 76-77 ºC.

HPLC analysis: CHIRALCEL OD-H column, 0.1% *i*PrOH in hexane, 0.2 mL/min, 254 nm UV detector, t_R (minor) = 30.5 min, t_R (major) = 32.1 min.

[α]$_D$^{20} = –5 (c = 0.19, $CHCl_3$).

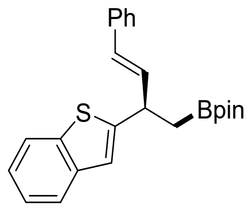

(*R,E*)-2-(2-(Benzo[*b*]thiophen-2-yl)-4-phenylbut-3-en-1-yl)-4,4,5,5-tetramethyl-1,3,2-dioxaborolane (44).

The title compound was prepared following the general procedure using 2-vinylbenzo[*b*]thiophene (24.0 mg, 0.150 mmol, 1.0 equiv), (*E*)-(2-bromovinyl)benzene (35.6 mg, 0.195 mmol, 1.3 equiv). After purification by column chromatography (using 1% ethyl acetate in petroleum ether), the title compound was isolated in 74% yield (43.3 mg, 90% *ee*) as a colorless oil.

^{1}H NMR (500 MHz, $CDCl_3$): δ 7.76 (d, J = 8.0 Hz, 1H), 7.67 (d, J = 7.7 Hz, 1H), 7.37 (d, J = 7.3 Hz, 2H), 7.30 (d, J = 7.3 Hz, 3H), 7.25 (d, J = 6.9 Hz, 1H), 7.21 (t, J = 7.3 Hz, 1H), 7.10 (s, 1H), 6.54 (d, J = 15.8 Hz, 1H), 6.39 (dd, J = 15.7, 7.8 Hz, 1H), 4.11 (dd, J = 15.5, 7.8 Hz, 1H), 1.52 (dt, J = 28.0, 7.6 Hz, 2H), 1.18 (d, J = 2.4 Hz, 12H).

^{13}C NMR (126 MHz, $CDCl_3$): δ 151.36, 140.19, 139.51, 137.35, 134.10, 129.70, 128.63, 127.39, 126.47, 124.13, 123.61, 123.09, 122.33, 119.70, 83.54, 40.73, 29.84, 24.94 (d, J = 13.8 Hz).

HRMS (ESI) m/z ([M+H]$^+$) calcd for $C_{24}H_{28}BO_2S$: 391.1898. Found: 391.1891.

HPLC analysis: CHIRALCEL OD-H column, 0.5% *i*PrOH in hexane, 0.3 mL/min, 254 nm UV detector, t_R (minor) = 18.1 min, t_R (major) = 18.8 min.

$[\alpha]_D^{20}$ = +9 (c = 0.04, $CHCl_3$).

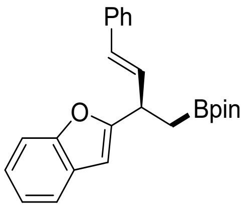

(*R,E*)-2-(2-(Benzofuran-2-yl)-4-phenylbut-3-en-1-yl)-4,4,5,5-tetramethyl-1,3,2-dioxaborolane (45).

The title compound was prepared following the general procedure using 2-vinylbenzofuran (21.6 mg, 0.150 mmol, 1.0 equiv), (*E*)-(2-bromovinyl)benzene (35.6 mg, 0.195 mmol, 1.3 equiv). After purification by column chromatography (using 1% ethyl acetate in petroleum ether), the title compound was isolated in 83% yield (46.6 mg, 96% *ee*) as a yellow solid.

^{1}H NMR (500 MHz, $CDCl_3$): δ 7.49–7.47 (m, 1H), 7.41 (d, J = 8.1 Hz, 1H), 7.36 (d, J = 7.3 Hz, 2H), 7.29 (d, J = 7.3 Hz, 2H), 7.23–7.15 (m, 3H), 6.54 (d, J = 15.8 Hz, 1H), 6.45 (s, 1H), 6.37 (dd, J = 15.8, 7.9 Hz, 1H), 3.98 (q, J = 7.9 Hz, 1H), 1.51 (dd, J = 15.5, 7.1 Hz, 1H), 1.39 (dd, J = 15.5, 8.3 Hz, 1H), 1.20 (d, J = 6.0 Hz, 12H).

^{13}C NMR (126 MHz, $CDCl_3$): δ 162.06, 154.90, 137.38, 132.96, 131.73, 130.47, 128.62, 127.39, 126.44, 123.37, 122.50, 120.54, 111.06, 101.51, 83.51, 38.73, 29.84, 25.95 (d, J = 10.7 Hz).

HRMS (ESI) m/z ([M+H]$^+$) calcd for $C_{24}H_{28}BO_3$: 375.2126. Found: 375.2129.

M.p.: 84-85 ºC.

HPLC analysis: CHIRALCEL OJ-3 column, 0.5% *i*PrOH in hexane, 0.1 mL/min, 254 nm UV detector, t_R (minor) = 85.7 min, t_R (major) = 92.0 min.

[α]$_D$^{20} = –1 (c = 0.13, $CHCl_3$).

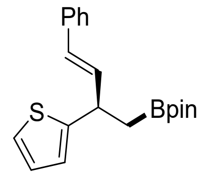

(*R,E*)-4,4,5,5-Tetramethyl-2-(4-phenyl-2-(thiophen-2-yl)but-3-en-1-yl)-1,3,2-dioxaborolane (46).

The title compound was prepared following the general procedure using 2-vinylthiophene (16.5 mg, 0.150 mmol, 1.0 equiv), (*E*)-(2-bromovinyl)benzene (35.6 mg, 0.195 mmol, 1.3 equiv). After purification by column chromatography (using 1% ethyl acetate in petroleum ether), the title compound was isolated in 70% yield (35.7 mg, 67% *ee*) as a yellow solid.

^{1}H NMR (500 MHz, $CDCl_3$): δ 7.35 (d, *J* = 7.3 Hz, 2H), 7.29 (t, *J* = 7.6 Hz, 2H), 7.20 (t, *J* = 7.3 Hz, 1H), 7.14 (dd, *J* = 5.1, 1.1 Hz, 1H), 6.93 (dd, *J* = 5.1, 3.5 Hz, 1H), 6.89 (d, *J* = 3.4 Hz, 1H), 6.48 (d, *J* = 15.8 Hz, 1H), 6.35 (dd, *J* = 15.7, 7.8 Hz, 1H), 4.06 (dd, *J* = 15.6, 7.8 Hz, 1H), 1.52–1.39 (m, 2H), 1.18 (d, *J* = 2.8 Hz, 12H).

^{13}C NMR (126 MHz, $CDCl_3$): δ 150.37, 137.49, 134.94, 129.01, 128.58, 127.24, 126.71, 126.42, 123.38, 123.27, 83.43, 40.05, 29.84, 24.92 (d, *J* = 13.8 Hz).

HRMS (ESI) m/z ($[M+H]^+$) calcd for $C_{20}H_{26}BO_2S$: 341.1741. Found: 341.1748.

M.p.: 93-94 ºC.

HPLC analysis: CHIRALCEL OD-H column, 0.5% *i*PrOH in hexane, 0.5 mL/min, 254 nm UV detector, t_R (minor) = 15.5 min, t_R (major) = 16.0 min.

[α]$_D$^{20} = –5 (c = 0.19, $CHCl_3$).

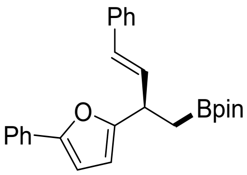

(*R,E*)-4,4,5,5-Tetramethyl-2-(4-phenyl-2-(5-phenylfuran-2-yl)but-3-en-1-yl)-1,3,2-dioxaborolane (47).

The title compound was prepared following the general procedure using 2-phenyl-5-vinylfuran (25.5 mg, 0.150 mmol, 1.0 equiv), (*E*)-(2-bromovinyl)benzene (35.6 mg, 0.195 mmol, 1.3 equiv). After purification by column chromatography (using 1% ethyl acetate in petroleum ether), the title compound was isolated in 67% yield (40.2 mg, 82% *ee*) as a colorless oil.

^{1}H NMR (500 MHz, $CDCl_3$): δ 7.65 (d, *J* = 7.2 Hz, 2H), 7.36 (dd, *J* = 14.6, 7.4 Hz, 4H), 7.30 (t, *J* =

7.6 Hz, 2H), 7.24–7.18 (m, 2H), 6.57 (d, *J* = 3.3 Hz, 1H), 6.51 (d, *J* = 15.8 Hz, 1H), 6.35 (dd, *J* = 15.8, 7.8 Hz, 1H), 6.17–6.13 (m, 1H), 3.92 (q, *J* = 7.8 Hz, 1H), 1.50 (dd, *J* = 15.5, 7.1 Hz, 1H), 1.36 (dd, *J* = 15.5, 8.5 Hz, 1H), 1.20 (d, *J* = 7.0 Hz, 12H).

^{13}C NMR (126 MHz, $CDCl_3$): *δ* 158.53, 152.58, 137.57, 132.57, 131.35, 129.83, 128.67, 128.59, 127.25, 126.92, 126.41, 123.61, 106.71, 105.77, 83.43, 38.53, 29.84, 24.95 (d, *J* = 15.0 Hz).

HRMS (ESI) m/z ($[M+H]^+$) calcd for $C_{26}H_{30}BO_3$: 401.2283. Found: 401.2281.

HPLC analysis: CHIRALCEL OD-H column, 0.5% *i*PrOH in hexane, 0.3 mL/min, 254 nm UV detector, t_R (minor) = 17.8 min, t_R (major) = 18.9 min.

$[\alpha]_D^{20}$ = +7 (c = 0.28, $CHCl_3$).

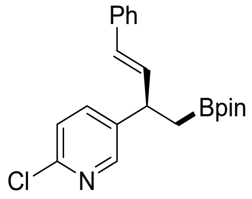

(*R,E*)-2-Chloro-5-(4-phenyl-1-(4,4,5,5-tetramethyl-1,3,2-dioxaborolan-2-yl)but-3-en-2-yl)pyridine (48).

The title compound was prepared following the general procedure using 2-chloro-5-vinylpyridine (20.9 mg, 0.150 mmol, 1.0 equiv), (*E*)-(2-bromovinyl)benzene (35.6 mg, 0.195 mmol, 1.3 equiv). After purification by column chromatography (using 2% ethyl acetate in petroleum ether), the title compound was isolated in 72% yield (39.8 mg, 91% *ee*) as a white solid.

^{1}H NMR (500 MHz, $CDCl_3$): *δ* 8.34 (d, *J* = 2.4 Hz, 1H), 7.59 (dd, *J* = 8.3, 2.4 Hz, 1H), 7.31 (dd, *J* = 6.7, 5.8 Hz, 3H), 7.28 (d, *J* = 1.6 Hz, 1H), 7.28–7.26 (m, 1H), 7.21 (t, *J* = 7.0 Hz, 1H), 6.42 (d, *J* = 15.9 Hz, 1H), 6.29 (dd, *J* = 15.8, 7.1 Hz, 1H), 3.81 (q, *J* = 7.6 Hz, 1H), 1.44–1.37 (m, 1H), 1.33 (t, *J* = 3.8 Hz, 1H), 1.15 (d, *J* = 3.5 Hz, 12H).

^{13}C NMR (126 MHz, $CDCl_3$): *δ* 149.01, 148.93, 140.39, 138.43, 137.04, 133.68, 129.92, 128.70, 127.59, 126.37, 124.24, 83.65, 41.25, 29.84, 24.91 (d, *J* = 6.3 Hz).

HRMS (ESI) m/z ($[M+H]^+$) calcd for $C_{21}H_{26}BClNO_2$: 370.1740. Found: 370.1745.

M.p.: 90-91 ºC.

HPLC analysis: CHIRALCEL OD-H column, 3% *i*PrOH in hexane, 0.5 mL/min, 254 nm UV detector, t_R (major) = 12.6 min, t_R (minor) = 14.0 min.

$[\alpha]_D^{20}$ = +4 (c = 0.24, $CHCl_3$).

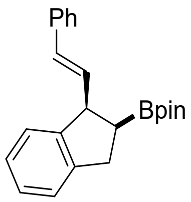

4,4,5,5-Tetramethyl-2-((1*R*,2*S*)-1-((*E*)-styryl)-2,3-dihydro-1*H*-inden-2-yl)-1,3,2-dioxaborolane (49).

The title compound was prepared following the general procedure using indene (17.4 mg, 0.150 mmol, 1.0 equiv), (*E*)-(2-bromovinyl)benzene (35.6 mg, 0.195 mmol, 1.3 equiv). After purification by column chromatography (using 1% ethyl acetate in petroleum ether), the title compound was isolated in 45% yield (23.3 mg, 40% *ee*) as a colorless oil.

^{1}H NMR (500 MHz, $CDCl_3$): δ 7.42–7.34 (m, 2H), 7.31 (dd, J = 14.8, 7.3 Hz, 2H), 7.26–7.12 (m, 5H), 6.58 (d, J = 15.7 Hz, 1H), 6.24 (dd, J = 15.7, 8.7 Hz, 1H), 4.05–3.92 (m, 1H), 3.02 (ddd, J = 26.7, 15.6, 10.1 Hz, 2H), 1.76 (dd, J = 19.9, 10.7 Hz, 1H), 1.26 (d, J = 3.6 Hz, 12H).

^{13}C NMR (126 MHz, $CDCl_3$): δ 146.60, 144.50, 137.84, 133.26, 130.88, 128.62, 127.16, 126.66, 126.38, 126.28, 124.38, 124.35, 83.47, 52.14, 34.53, 32.08, 29.85, 29.52, 24.94 (d, J = 13.5 Hz), 22.85, 14.27.

HRMS (ESI) m/z ($[M+H]^+$) calcd for $C_{23}H_{28}BO_2$: 347.2177. Found: 347.2178.

HPLC analysis: CHIRALCEL OD-H column, 0.2% *i*PrOH in hexane, 0.2 mL/min, 254 nm UV detector, t_R (major) = 25.5 min, t_R (minor) = 26.7 min.

$[\alpha]_D^{20}$ = –1 (c = 0.24, $CHCl_3$).

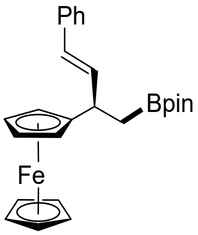

(*R,E*)-2-(2,4-Di-ferrocene-but-3-en-1-yl)-4,4,5,5-tetramethyl-1,3,2-dioxaborolane (50).

The title compound was prepared following the general procedure using alkene (31.8 mg, 0.150 mmol, 1.0 equiv), (*E*)-(2-bromovinyl)benzene (35.6 mg, 0.195 mmol, 1.3 equiv). After purification by column chromatography (using 1% ethyl acetate in petroleum ether), the title compound was isolated in 82% yield (54.4 mg, 74% *ee*) as a brown oil.

^{1}H NMR (500 MHz, $CDCl_3$): δ 7.38 (d, J = 7.6 Hz, 2H), 7.31 (t, J = 7.7 Hz, 2H), 7.20 (t, J = 7.3 Hz, 1H), 6.39 (dt, J = 15.8, 11.9 Hz, 2H), 4.15 (s, 4H), 4.11 (s, 2H), 4.08 (s, 3H), 3.47 (ddd, J = 10.1, 8.0, 5.1 Hz, 1H), 1.40–1.33 (m, 2H), 1.21 (d, J = 1.7 Hz, 12H).

^{13}C NMR (126 MHz, $CDCl_3$): δ 137.96, 135.38, 128.62, 128.44, 126.99, 126.25, 95.03, 83.31, 69.58,

68.52, 67.35, 67.15, 67.04, 66.72, 38.36, 25.06 (d, J = 12.0 Hz).

HRMS (ESI) m/z ([M+H]$^+$) calcd for $C_{26}H_{32}BFeO_2$: 443.1839. Found: 443.1833.

HPLC analysis: CHIRALCEL OD-H column, 0.5% *i*PrOH in hexane, 0.5 mL/min, 254 nm UV detector, t_R (minor) = 10.3 min, t_R (major) = 12.6 min.

[α]$_D^{20}$ = +25 (c = 0.31, CHCl$_3$).

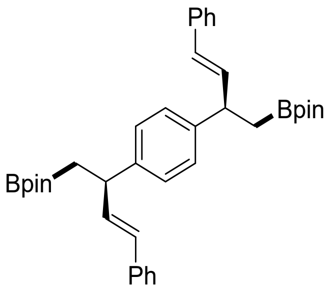

1,4-Bis((*R,E*)-4-phenyl-1-(4,4,5,5-tetramethyl-1,3,2-dioxaborolan-2-yl)but-3-en-2-yl)benzene (51).

The title compound was prepared following the general procedure using alkene (19.5 mg, 0.150 mmol, 1.0 equiv), (*E*)-(2-bromovinyl)benzene (71.2 mg, 0.39 mmol, 2.6 equiv). After purification by column chromatography (using 2% ethyl acetate in petroleum ether), the title compound was isolated in 43% yield (36.3 mg, 86% *de*) as a colorless oil.

^{1}H NMR (500 MHz, CDCl$_3$): δ 7.31 (d, J = 7.5 Hz, 4H), 7.25 (dd, J = 9.2, 6.0 Hz, 4H), 7.20 (s, 4H), 7.16 (t, J = 7.3 Hz, 2H), 6.47–6.28 (m, 4H), 3.74 (dd, J = 15.7, 7.0 Hz, 2H), 1.38–1.30 (m, 4H), 1.13 (s, 24H).

^{13}C NMR (126 MHz, CDCl$_3$): δ 143.72, 137.84, 135.91, 128.52, 128.29, 127.54, 126.98, 126.28, 83.29, 44.14, 29.84, 24.91 (d, J = 11.3 Hz).

HRMS (ESI) m/z ([M+H]$^+$) calcd for $C_{38}H_{49}B_2O_4$: 591.3811. Found: 591.3808.

HPLC analysis: CHIRALCEL AD-H column, 5% EtOH in hexane, 0.5 mL/min, 254 nm UV detector, t_R (major) = 9.0 min, t_R (medium) = 10.8 min, t_R (minor) = 12.9 min.

[α]$_D^{20}$ = +15 (c = 0.30, CHCl$_3$).

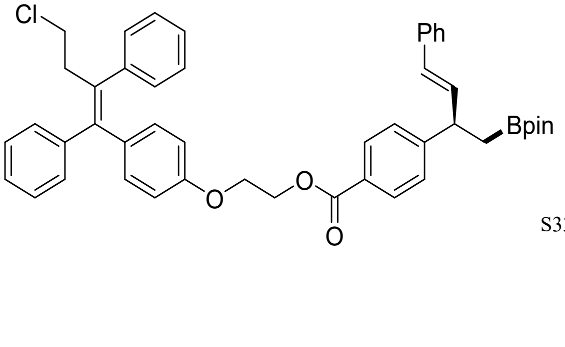

2-(4-((*Z*)-4-Chloro-1,2-diphenylbut-1-en-1-yl)phenoxy)ethyl-4-((*R,E*)-4-phenyl-1-(4,4,5,5-tetramethyl-1,3,2-dioxaborolan-2-yl)but-3-en-

2-yl)benzoate (52).

The title compound was prepared following the general procedure using alkene (76.4 mg, 0.150 mmol, 1.0 equiv), (*E*)-(2-bromovinyl)benzene (35.6 mg, 0.195 mmol, 1.3 equiv). After purification by column chromatography (using 3% ethyl acetate in petroleum ether), the title compound was isolated in 84% yield (93.1 mg, 95% *ee*) as a white solid.

^{1}H NMR (500 MHz, $CDCl_3$): δ 7.95 (d, J = 8.3 Hz, 2H), 7.39–7.34 (m, 3H), 7.34–7.30 (m, 3H), 7.30 – 7.27 (m, 4H), 7.26 (d, J = 2.0 Hz, 1H), 7.22–7.17 (m, 3H), 7.16–7.12 (m, 3H), 6.82–6.77 (m, 2H), 6.62–6.57 (m, 2H), 6.36 (dt, J = 15.8, 11.4 Hz, 2H), 4.59–4.52 (m, 2H), 4.19–4.13 (m, 2H), 3.83 (q, J = 7.6 Hz, 1H), 3.42 (t, J = 7.5 Hz, 2H), 2.92 (t, J = 7.5 Hz, 2H), 1.38 (dd, J = 16.4, 8.0 Hz, 2H), 1.14 (d, J = 4.2 Hz, 12H).

^{13}C NMR (126 MHz, $CDCl_3$): δ 166.64, 156.90, 151.60, 142.98, 141.79, 141.06, 137.45, 135.45, 135.41, 134.70, 131.89, 130.06, 129.66, 129.52, 129.18, 128.69, 128.50, 128.38, 127.90, 127.67, 127.29, 127.10, 126.77, 126.33, 113.74, 83.45, 65.92, 63.29, 44.53, 42.99, 38.74, 29.84, 24.90 (d, J = 11.7 Hz).

HRMS (ESI) m/z ($[M+H]^+$) calcd for $C_{47}H_{49}BClO_5$: 739.3356. Found: 739.3351.

M.p.: 122-123 ºC.

HPLC analysis: CHIRALCEL OD-H column, 5% *i*PrOH in hexane, 0.1 mL/min, 254 nm UV detector, t_R (major) = 98.4 min, t_R (minor) = 104.4 min.

$[\alpha]_D^{20}$ = +4 (c = 0.43, $CHCl_3$).

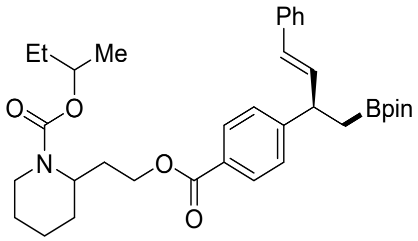

***sec*-Butyl-2-(2-((4-((*R,E*)-4-phenyl-1-(4,4,5,5-tetramethyl-1,3,2-dioxaborolan-2-yl)but-3-en-2-yl)benzoyl)oxy)ethyl)piperidine-1-carboxylate (53)**.

The title compound was prepared following the general procedure using alkene (53.9 mg, 0.150 mmol, 1.0 equiv), (*E*)-(2-bromovinyl)benzene (35.6 mg, 0.195 mmol, 1.3 equiv). After purification by column chromatography (using 5% ethyl acetate in petroleum ether), the title compound was isolated in 83% yield (73.3 mg, 96% *ee*) as a colorless oil.

^{1}H NMR (500 MHz, $CDCl_3$): δ 7.97 (d, J = 8.2 Hz, 2H), 7.35 (d, J = 8.3 Hz, 2H), 7.31 (d, J = 7.2 Hz,

2H), 7.26 (dd, J = 9.2, 6.1 Hz, 2H), 7.18 (t, J = 7.2 Hz, 1H), 6.36 (dt, J = 15.8, 11.4 Hz, 2H), 4.73 (dd, J = 12.5, 6.2 Hz, 1H), 4.51 (s, 1H), 4.30 (dd, J = 11.4, 6.4 Hz, 2H), 4.08 (s, 1H), 3.84 (q, J = 7.6 Hz, 1H), 2.86 (t, J = 12.9 Hz, 1H), 2.19 (tt, J = 15.4, 6.4 Hz, 1H), 1.89 (dt, J = 17.6, 5.1 Hz, 1H), 1.72–1.53 (m, 8H), 1.38 (dd, J = 15.4, 8.0 Hz, 2H), 1.16 (d, J = 6.2 Hz, 3H), 1.14 (d, J = 4.2 Hz, 12H), 0.87 (d, J = 7.3 Hz, 3H).

^{13}C NMR (126 MHz, $CDCl_3$): δ 166.67, 155.63, 151.32, 137.45, 134.74, 129.90, 129.11, 128.58, 128.34, 127.61, 127.25, 126.29, 83.41, 73.07, 62.65, 48.19, 44.50, 39.12, 29.18, 29.04, 28.71, 25.61, 24.87 (d, J = 12.2 Hz), 19.89, 19.18, 14.31, 9.86.

HRMS (ESI) m/z ($[M+H]^+$) calcd for $C_{35}H_{49}BNO_6$: 590.3647. Found: 590.3648.

HPLC analysis: CHIRALCEL AS-H column, 2% *i*PrOH in hexane, 0.1 mL/min, 254 nm UV detector, t_R (minor) = 8.8 min, t_R (major) = 9.6 min.

$[\alpha]_D^{20}$ = +3 (c = 0.34, $CHCl_3$).

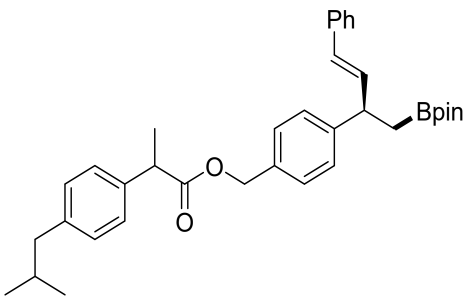

4-((*R*,*E*)-4-Phenyl-1-(4,4,5,5-tetramethyl-1,3,2-dioxaborolan-2-yl)but-3-en-2-yl)benzyl-2-(4-isobutylphenyl)propanoate (54).

The title compound was prepared following the general procedure using alkene (48.4 mg, 0.150 mmol, 1.0 equiv), (*E*)-(2-bromovinyl)benzene (35.6 mg, 0.195 mmol, 1.3 equiv). After purification by column chromatography (using 1% ethyl acetate in petroleum ether), the title compound was isolated in 86% yield (71.2 mg, 99% *ee*) as a colorless oil.

^{1}H NMR (500 MHz, $CDCl_3$): δ 7.34 (d, J = 7.2 Hz, 2H), 7.27 (dd, J = 14.3, 7.1 Hz, 3H), 7.24 (s, 1H), 7.22–7.16 (m, 5H), 7.09 (d, J = 8.1 Hz, 2H), 6.38 (dt, J = 15.8, 11.5 Hz, 2H), 5.08 (q, J = 12.5 Hz, 2H), 3.78 (dd, J = 14.5, 6.5 Hz, 1H), 3.74 (t, J = 7.2 Hz, 1H), 2.45 (d, J = 7.2 Hz, 2H), 1.86 (td, J = 13.5, 6.8 Hz, 1H), 1.51 (d, J = 7.2 Hz, 3H), 1.42–1.32 (m, 2H), 1.15 (s, 11H), 0.91 (d, J = 6.6 Hz, 7H).

^{13}C NMR (150 MHz, $CDCl_3$): δ 174.69, 145.88, 140.66, 137.72 (d, J = 15.3 Hz), 135.43, 133.97, 129.43, 128.61,128.56, 128.12, 128.11, 127.67, 127.34, 127.11, 126.29, 83.34, 66.37, 45.29, 45.16, 44.23, 30.31, 29.83, 24.89 (d, J = 18.2 Hz), 22.51, 18.58.

HRMS (ESI) m/z ($[M+Na]^+$) calcd for $C_{36}H_{45}BNaO_4$: 575.3303. Found: 575.3298.

HPLC analysis: CHIRALCEL AD-H column, 0.5% *i*PrOH in hexane, 1.0 mL/min, 254 nm UV detector, t_R (minor) = 6.1 min, t_R (major) = 7.9 min.

$[\alpha]_D^{20}$ = +1 (c = 0.33, $CHCl_3$).

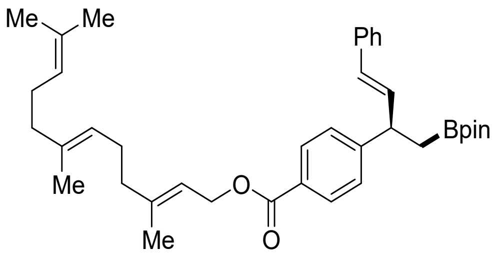

3,7,11-Trimethyldodeca-2,6,10-trien-1-yl-4-(*R*-4-phenyl-1-(4,4,5,5-tetramethyl-1,3,2-dioxaborolan-2-yl)but-3-en-2-yl)benzoate (55).

The title compound was prepared following the general procedure using alkene (mixture of isomers) (53.0 mg, 0.150 mmol, 1.0 equiv), (*E*)-(2-bromovinyl)benzene (35.6 mg, 0.195 mmol, 1.3 equiv). After purification by column chromatography (using 1% ethyl acetate in petroleum ether), the title compound was isolated in 90% yield (78.6 mg, 97% *ee*) as a colorless oil.

^{1}H NMR (500 MHz, $CDCl_3$): δ 8.03–7.91 (m, 2H), 7.38–7.30 (m, 4H), 7.27 (dd, J = 10.3, 4.9 Hz, 2H), 7.19 (t, J = 7.2 Hz, 1H), 6.37 (dt, J = 15.8, 11.4 Hz, 2H), 5.47 (t, J = 7.0 Hz, 1H), 5.10 (ddd, J = 9.0, 6.9, 3.5 Hz, 2H), 4.83 (d, J = 7.0 Hz, 2H), 3.84 (q, J = 7.6 Hz, 1H), 2.16–1.95 (m, 8H), 1.76 (s, 3H), 1.68 (s, 3H), 1.60 (d, J = 6.0 Hz, 6H), 1.43–1.36 (m, 2H), 1.14 (d, J = 4.1 Hz, 12H).

^{13}C NMR (126 MHz, $CDCl_3$): δ 166.82, 151.20, 142.32, 137.48, 135.58, 134.81, 131.43, 129.93, 129.11, 128.60, 127.60, 127.25, 126.32, 124.60, 124.46, 123.78, 118.66, 83.43, 61.89, 44.52, 39.82 (t, J = 18.9 Hz), 32.09 (d, J = 6.2 Hz), 29.84, 29.50, 26.78 (d, J = 15.2 Hz), 26.30 (d, J = 15.1 Hz), 25.85 (d, J = 3.6 Hz), 24.89 (d, J = 11.6 Hz), 23.51, 22.83, 17.80 (d, J = 5.5 Hz), 16.71, 16.17, 14.26, 1.16.

HRMS (ESI) m/z ($[M+H]^+$) calcd for $C_{38}H_{52}BO_4$: 583.3953. Found: 583.3949.

HPLC analysis: CHIRALCEL AD-H column, 5% *i*PrOH in hexane, 1.0 mL/min, 254 nm UV detector, t_R (major) = 5.0 min, t_R (minor) = 5.4 min, t_R (major) = 5.7 min, t_R (minor) = 6.1 min.

$[\alpha]_D^{20}$ = +5 (c = 0.48, $CHCl_3$).

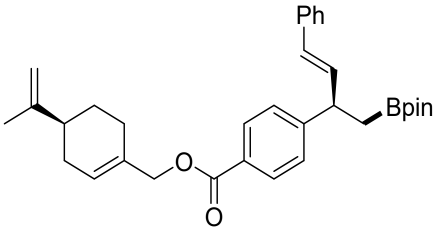

((*S*)-4-(Prop-1-en-2-yl)cyclohex-1-en-1-yl)methyl-4-((*R*,*E*)-4-phenyl-1-(4,4,5,5-tetramethyl-1,3,2-

dioxaborolan-2-yl)but-3-en-2-yl)benzoate (56).

The title compound was prepared following the general procedure using alkene (42.4 mg, 0.150 mmol, 1.0 equiv), (*E*)-(2-bromovinyl)benzene (35.6 mg, 0.195 mmol, 1.3 equiv). After purification by column chromatography (using 1% ethyl acetate in petroleum ether), the title compound was isolated in 72% yield (55.3 mg, 99% *de*) as a colorless oil.

^{1}H NMR (500 MHz, $CDCl_3$): δ 7.99 (d, J = 8.2 Hz, 2H), 7.36 (d, J = 8.3 Hz, 2H), 7.32 (d, J = 7.3 Hz, 2H), 7.27 (dd, J = 10.4, 4.8 Hz, 2H), 7.19 (t, J = 7.2 Hz, 1H), 6.37 (dt, J = 15.8, 11.4 Hz, 2H), 5.83 (s, 1H), 4.73 (d, J = 5.7 Hz, 2H), 4.70 (s, 2H), 3.84 (q, J = 7.6 Hz, 1H), 2.23–2.12 (m, 4H), 2.06–1.96 (m, 1H), 1.90–1.84 (m, 1H), 1.74 (s, 3H), 1.52 (ddd, J = 11.8, 6.9, 3.2 Hz, 1H), 1.43–1.35 (m, 2H), 1.14 (d, J = 3.6 Hz, 12H).

^{13}C NMR (126 MHz, $CDCl_3$): δ 166.64, 151.36, 149.79, 137.47, 134.76, 132.91, 129.96, 129.15, 128.61, 128.43, 127.66, 127.28, 126.33, 125.59, 108.91, 83.45, 68.80, 44.54, 41.02, 30.61, 29.84, 27.48, 26.58, 24.90 (d, J = 12.5 Hz), 20.90.

HRMS (ESI) m/z ($[M+H]^+$) calcd for $C_{33}H_{42}BO_4$: 513.3171. Found: 513.3171.

HPLC analysis: CHIRALCEL OD-H column, 0.5% *i*PrOH in hexane, 0.2 mL/min, 254 nm UV detector, t_R (minor) = 36.3 min, t_R (major) = 39.5 min.

$[\alpha]_D^{20}$ = –12 (c = 0.16, $CHCl_3$).

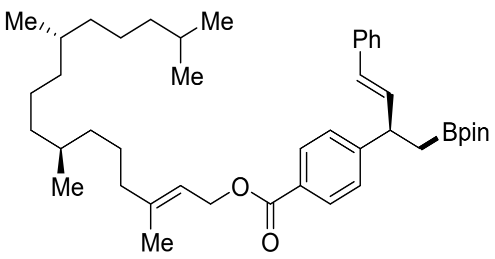

(7*S*,11*R*,*E*)-3,7,11,15-Tetramethylhexadec-2-en-1-yl-4-((*R*,*E*)-4-phenyl-1-(4,4,5,5-tetramethyl-1,3,2-dioxaborolan-2-yl)but-3-en-2-yl)benzoate (57).

The title compound was prepared following the general procedure using alkene (64.0 mg, 0.150 mmol, 1.0 equiv), (*E*)-(2-bromovinyl)benzene (35.6 mg, 0.195 mmol, 1.3 equiv). After purification by column chromatography (using 1% ethyl acetate in petroleum ether), the title compound was isolated in 80% yield (78.7 mg, 99% *de*) as a colorless oil.

^{1}H NMR (500 MHz, $CDCl_3$): δ 7.98 (d, J = 8.3 Hz, 2H), 7.35 (d, J = 8.3 Hz, 2H), 7.32 (d, J = 7.4 Hz, 2H), 7.29–7.26 (m, 2H), 7.19 (t, J = 7.2 Hz, 1H), 6.37 (dt, J = 15.8, 11.4 Hz, 2H), 5.46 (t, J = 6.6 Hz, 1H),

4.82 (d, J = 7.0 Hz, 2H), 3.84 (q, J = 7.6 Hz, 1H), 2.03 (t, J = 9.1 Hz, 2H), 1.75 (s, 3H), 1.52 (dt, J = 13.3, 6.7 Hz, 1H), 1.45–1.33 (m, 7H), 1.31–1.24 (m, 11H), 1.14 (d, J = 4.0 Hz, 12H), 0.90–0.82 (m, 14H).

^{13}C NMR (126 MHz, $CDCl_3$): δ 166.84, 151.19, 142.81, 137.49, 134.81, 131.04, 129.93, 129.11, 128.60, 127.60, 127.25, 126.32, 118.38, 83.42, 61.92, 44.52, 40.03, 39.51, 37.56, 37.49, 37.42, 36.79, 32.92, 32.80, 29.84, 28.11, 25.20, 24.90 (d, J = 11.6 Hz), 24.60, 22.86, 22.77, 19.89, 19.86, 16.61.

HRMS (ESI) m/z ($[M+H]^+$) calcd for $C_{43}H_{66}BO_4$: 657.5049. Found: 657.5048.

HPLC analysis: CHIRALCEL OD-H column, 0.5% *i*PrOH in hexane, 0.5 mL/min, 254 nm UV detector, t_R (minor) = 9.6 min, t_R (major) = 10.2 min.

$[\alpha]_D^{20}$ = +2 (c = 0.29, $CHCl_3$).

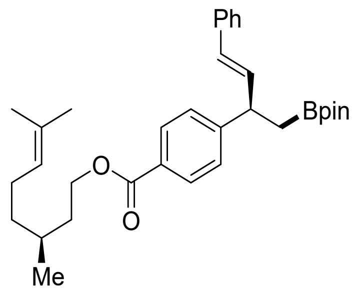

(*S*)-3,7-Dimethyloct-6-en-1-yl-4-((*R,E*)-4-phenyl-1-(4,4,5,5-tetramethyl-1,3,2-dioxaborolan-2-yl)but-3-en-2-yl)benzoate (58).

The title compound was prepared following the general procedure using alkene (43.0 mg, 0.150 mmol, 1.0 equiv), (*E*)-(2-bromovinyl)benzene (35.6 mg, 0.195 mmol, 1.3 equiv). After purification by column chromatography (using 1% ethyl acetate in petroleum ether), the title compound was isolated in 78% yield (60.4 mg, 99% *de*) as a colorless oil.

^{1}H NMR (500 MHz, $CDCl_3$): δ 7.96 (d, J = 8.2 Hz, 2H), 7.35 (d, J = 8.2 Hz, 2H), 7.32 (d, J = 7.5 Hz, 2H), 7.29–7.25 (m, 2H), 7.18 (t, J = 7.2 Hz, 1H), 6.36 (dt, J = 15.8, 11.4 Hz, 2H), 5.09 (t, J = 7.0 Hz, 1H), 4.33 (dt, J = 11.8, 5.9 Hz, 2H), 3.84 (q, J = 7.6 Hz, 1H), 1.99 (ddd, J = 21.6, 15.0, 7.2 Hz, 2H), 1.80 (td, J = 12.5, 7.0 Hz, 1H), 1.67 (s, 3H), 1.65–1.62 (m, 1H), 1.60 (s, 3H), 1.55 (dd, J = 14.1, 6.8 Hz, 1H), 1.41 (ddd, J = 23.0, 9.7, 5.0 Hz, 3H), 1.34 (d, J = 4.7 Hz, 1H), 1.14 (d, J = 3.6 Hz, 12H), 0.96 (d, J = 6.6 Hz, 3H).

^{13}C NMR (126 MHz, $CDCl_3$): δ 166.84, 151.23, 137.48, 134.78, 131.49, 129.87, 129.12, 128.60, 128.57, 127.63, 127.26, 126.32, 124.72, 83.43, 63.48, 44.52, 37.13, 35.65, 29.83, 29.72, 25.84, 25.54, 24.89 (d, J = 12.5 Hz), 19.64, 17.80.

HRMS (ESI) m/z ($[M+H]^+$) calcd for $C_{33}H_{46}BO_4$: 517.3484. Found: 517.3488.

HPLC analysis: CHIRALCEL OD-H column, 0.5% *i*PrOH in hexane, 0.5 mL/min, 254 nm UV

detector, t_R (minor) = 12.4 min, t_R (major) = 13.4 min.

[α]$_D$^{20} = +2 (c = 0.22, $CHCl_3$).

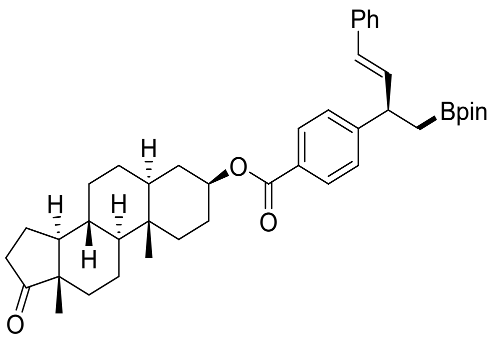

(*3S,5S,8R,9S,10S,13S,14S*)-10,13-Dimethyl-17-oxohexadecahydro-1*H*-cyclopenta[a]phenanthren-3-yl-4-((*R,E*)-4-phenyl-1-(4,4,5,5-tetramethyl-1,3,2-dioxaborolan-2-yl)but-3-en-2-yl)benzoate (59).

The title compound was prepared following the general procedure using alkene (63.1 mg, 0.150 mmol, 1.0 equiv), (*E*)-(2-bromovinyl)benzene (35.6 mg, 0.195 mmol, 1.3 equiv). After purification by column chromatography (using 5% ethyl acetate in petroleum ether), the title compound was isolated in 94% yield (91.6 mg, 95% *de*) as a white solid.

^{1}H NMR (500 MHz, $CDCl_3$): *δ* 7.95 (d, *J* = 8.3 Hz, 2H), 7.33 (dd, *J* = 13.7, 7.8 Hz, 4H), 7.28–7.26 (m, 2H), 7.18 (t, *J* = 7.2 Hz, 1H), 6.36 (dt, *J* = 15.8, 11.4 Hz, 2H), 4.96–4.88 (m, 1H), 3.87–3.79 (m, 1H), 2.44 (dd, *J* = 19.3, 8.7 Hz, 1H), 2.11–2.03 (m, 1H), 1.99–1.90 (m, 2H), 1.85–1.71 (m, 5H), 1.70–1.63 (m, 2H), 1.56–1.46 (m, 3H), 1.46–1.27 (m, 10H), 1.14 (d, *J* = 3.7 Hz, 12H), 0.90 (s, 3H), 0.87 (s, 3H).

^{13}C NMR (126 MHz, $CDCl_3$): *δ* 166.29, 151.17, 137.49, 134.80, 129.86, 129.12, 128.91, 128.61, 127.56, 127.26, 126.32, 100.12, 83.45, 74.06, 54.48, 51.52, 47.95, 44.87, 44.51, 36.91, 36.01, 35.86, 35.20, 34.20, 31.68, 30.98, 29.84, 28.45, 27.68, 24.91 (d, *J* = 12.4 Hz), 21.93, 20.63, 13.97, 12.43.

HRMS (ESI) m/z ([M+H]$^+$) calcd for $C_{42}H_{56}BO_5$: 651.4215. Found: 651.4218.

M.p.: 159-160 ºC.

HPLC analysis: CHIRALCEL OD-H column, 5% *i*PrOH in hexane, 0.5 mL/min, 254 nm UV detector, t_R (major) = 27.4 min, t_R (minor) = 30.3 min.

[α]$_D$^{20} = +36 (c = 0.54, $CHCl_3$).

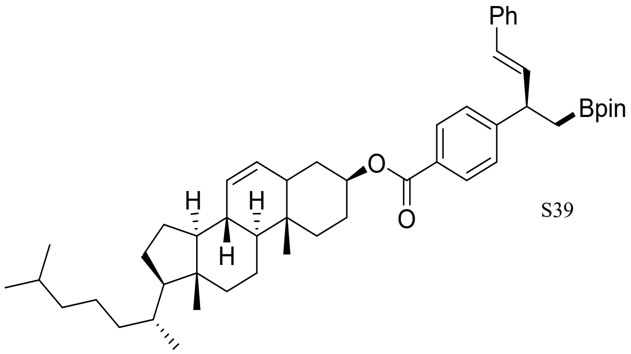

(*3S,8S,9S,10S,13R,14S,17R*)-10,13-Dimethyl-17-((*R*)-6-methylheptan-2-yl)-

2,3,4,5,8,9,10,11,12,13,14,15,16,17-tetradecahydro-1*H*-cyclopenta[*a*]phenanthren-3-yl-4-((*R,E*)-4-phenyl-1-(4,4,5,5-tetramethyl-1,3,2-dioxaborolan-2-yl)but-3-en-2-yl)benzoate (60).

The title compound was prepared following the general procedure using alkene (77.5 mg, 0.150 mmol, 1.0 equiv), (*E*)-(2-bromovinyl)benzene (35.6 mg, 0.195 mmol, 1.3 equiv). After purification by column chromatography (using 1% ethyl acetate in petroleum ether), the title compound was isolated in 91% yield (101.8 mg, 97% *de*) as a white solid.

^{1}H NMR (500 MHz, $CDCl_3$): δ 7.97 (d, J = 8.3 Hz, 2H), 7.33 (dd, J = 15.1, 7.8 Hz, 4H), 7.29–7.26 (m, 2H), 7.18 (t, J = 7.2 Hz, 1H), 6.36 (dt, J = 15.8, 11.4 Hz, 2H), 5.41 (d, J = 3.9 Hz, 1H), 4.88–4.80 (m, 1H), 3.84 (q, J = 7.5 Hz, 1H), 2.45 (d, J = 7.8 Hz, 2H), 2.06–1.95 (m, 3H), 1.94–1.81 (m, 2H), 1.77–1.68 (m, 1H), 1.64–1.32 (m, 15H), 1.24–1.17 (m, 3H), 1.14 (d, J = 3.8 Hz, 12H), 1.07 (s, 3H), 1.05–0.96 (m, 4H), 0.92 (d, J = 6.5 Hz, 3H), 0.87 (dd, J = 6.6, 2.2 Hz, 6H), 0.69 (s, 3H).

^{13}C NMR (126 MHz, $CDCl_3$): δ 166.17, 151.16, 139.89, 137.50, 134.83, 129.88, 129.11, 128.90, 128.61, 127.58, 127.26, 126.33, 122.85, 83.45, 74.53, 56.85, 56.28, 50.19, 44.51, 42.47, 39.89, 39.67, 38.38, 37.19, 36.80, 36.33, 35.95, 32.09, 32.03, 29.85, 28.39, 28.16, 28.04, 24.91 (d, J = 11.9 Hz), 24.44, 23.98, 22.97, 22.71, 21.20, 19.53, 18.87, 12.01.

HRMS (ESI) m/z ($[M+H]^+$) calcd for $C_{50}H_{72}BO_4$: 747.5518. Found: 747.5517.

M.p.: 186-187 ºC.

HPLC analysis: CHIRALCEL OD-H column, 0.5% *i*PrOH in hexane, 0.1 mL/min, 254 nm UV detector, t_R (major) = 66.8 min, t_R (minor) = 85.3 min.

$[\alpha]_D^{20}$ = +3 (c = 0.51, $CHCl_3$).

III. Preparation of Alkenyl Bromides

A general procedure for the preparation of vinyl bromides. [3-5]

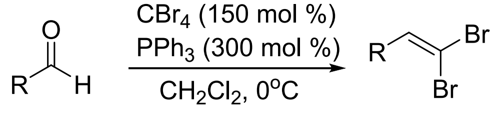

Step 1: To a flame-dried flask was added aldehyde (20 mmol, 100 mol%), CBr_4 (30 mmol, 150 mol%), and CH_2Cl_2 (80 mL). The flask was cooled to 0 °C, at which point a solution of PPh_3 (60 mmol, 300 mol%) in CH_2Cl_2 (70 mL) was added dropwise via addition funnel over 30 min. The solution was stirred at 0 °C under N_2 for 1 h. About half of the volume of CH_2Cl_2 was removed under reduced pressure. Pentane (100 mL) was added, and triphenylphosphine oxide (TPPO) precipitated out. After filtration and evaporation of the solvent, the residue was dissolved in pentane (50 mL) which led to further precipitation of TPPO. Filtration and evaporation of the solvent afforded the crud dibromide which was directly used for the next step.

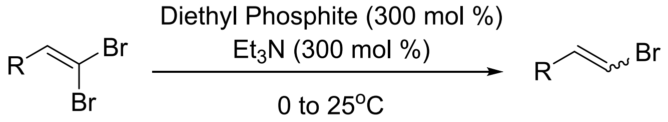

Step 2: To a solution of the crude dibromide (~ 20.0 mmol, 100 mol%) and NEt_3 (60 mmol, 300 mol%) in DMF (20 mL) was added dimethyl phosphonate (60.0 mmol, 300 mol%). The solution was stirred over night at room temperature. Water (60 mL) was added to the mixture, which was extracted with pentane (2 × 100 mL). The combined organic phases were washed with an aqueous solution of HCl (1 M, 55 mL) and dried over Na_2SO_4, filtered, and concentrated. The crude material was purified by flash chromatography.

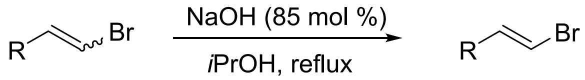

Step 3: The crude product (~20.0 mmol, 100 mol%) from the previous step was dissolved in *i*PrOH (30 mL). Solid NaOH (17.0 mmol, 85 mol%) was added and the mixture was heated to reflux for 1.5 hours. The reaction mixture was cooled to room temperature, diluted with pentane (100 mL), and partitioned with distillated H_2O (2 × 100 mL). The organic phase was collected, and washed with an aqueous solution of HCl (1 M, 75 mL), dried over Na_2SO_4. The solvent was removed under reduced pressure. The crude material was purified by flash chromatography.

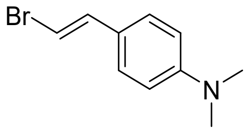

(*E*)-4-(2-Bromovinyl)-N,N-dimethylaniline.

This compound was prepared from according to general procedure. The

crude residue was purified by silicagel chromatography (hexanes) to give the title compound in 84% yield (3.8 g) as a white solid.

<u>^{1}H NMR</u> (500 MHz, $CDCl_3$): δ 7.22–7.14 (m, 2H), 7.00 (d, J = 13.9 Hz, 1H), 6.68 (d, J = 8.5 Hz, 2H), 6.52 (d, J = 13.9 Hz, 1H), 2.97 (s, 6H).

<u>^{13}C NMR</u> (126 MHz, $CDCl_3$): δ 150.52, 137.10, 127.28, 124.45, 112.38, 101.65, 40.49.

<u>HRMS</u> (ESI) m/z ($[M+H]^+$) calcd for $C_{10}H_{13}BrN$: 226.0226. Found: 226.0227.

<u>M.p.</u>: 118-119 ºC.

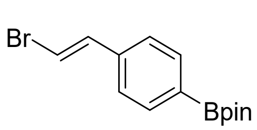

(*E*)-2-(4-(2-Bromovinyl)phenyl)-4,4,5,5-tetramethyl-1,3,2-dioxaborolane.

This compound was prepared according to general procedure. The crude residue was purified by silicagel chromatography (hexanes) to give the title compound in 65% yield (4.0 g) as a white solid.

<u>^{1}H NMR</u> (500 MHz, $CDCl_3$): δ 7.76 (d, J = 8.1 Hz, 2H), 7.30 (d, J = 8.0 Hz, 2H), 7.11 (d, J = 14.0 Hz, 1H), 6.85 (d, J = 14.0 Hz, 1H), 1.34 (s, 12H).

<u>^{13}C NMR</u> (126 MHz, $CDCl_3$): δ 138.54, 137.34, 135.36, 125.50, 107.86, 84.04, 25.00.

<u>HRMS</u> (ESI) m/z ($[M+H]^+$) calcd for $C_{14}H_{19}BBrO_2$: 309.0656. Found: 309.0651.

<u>M.p.</u>: 36-37 ºC.

IV. Preparation Part of Alkenes

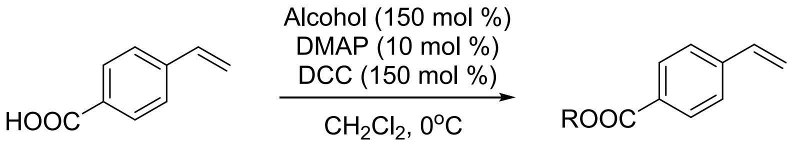

To a flame-dried flask was added acid (10 mmol, 100 mol%), DMAP (1 mmol, 10 mol%), DCC

(15 mmol, 150 mol%), and CH_2Cl_2 (30 mL). The flask was cooled to 0 °C, at which point a solution of alcohol (15 mmol, 150 mol%) in CH_2Cl_2 (30 mL) was added dropwise via addition funnel over 30 min. The solution was stirred at 0 °C under N_2 over night. After the reaction was finshed the CH_2Cl_2 was removed under reduced pressure. And The crude residue was purified by silicagel chromatography (hexanes) to give the target compound.

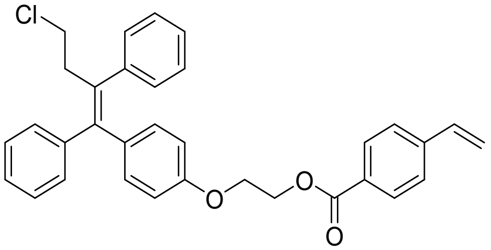

(*Z*)-2-(4-(4-Chloro-1,2-diphenylbut-1-en-1-yl)phenoxy)ethyl 4-vinylbenzoate.

The title compound was prepared following the general procedure using 4-vinylbenzoic acid (1.5 g, 10 mmol, 1.0 equiv), ospemifene (5.7 g, 15 mmol, 1.5 equiv). After purification by column chromatography (using 5% ethyl acetate in petroleum ether), the title compound was isolated in 90% yield (4.6 g) as a white solid.

^{1}H NMR (500 MHz, $CDCl_3$): δ 7.98 (d, J = 8.3 Hz, 2H), 7.44 (d, J = 8.3 Hz, 2H), 7.40–7.35 (m, 2H), 7.30 (dd, J = 7.2, 5.5 Hz, 3H), 7.23–7.18 (m, 2H), 7.17–7.13 (m, 3H), 6.85–6.79 (m, 2H), 6.75 (dd, J = 17.6, 10.9 Hz, 1H), 6.60 (d, J = 8.8 Hz, 2H), 5.86 (d, J = 17.6 Hz, 1H), 5.39 (d, J = 10.9 Hz, 1H), 4.62–4.49 (m, 2H), 4.26–4.12 (m, 2H), 3.42 (t, J = 7.5 Hz, 2H), 2.93 (t, J = 7.5 Hz, 2H).

^{13}C NMR (126 MHz, $CDCl_3$): δ 166.40, 156.89, 142.98, 142.24, 141.78, 141.06, 136.14, 135.48, 135.45, 131.90, 130.18, 129.67, 129.53, 129.11, 128.50, 128.38, 127.11, 126.77, 126.23, 116.74, 113.75, 65.89, 63.43, 42.98, 38.73.

HRMS (ESI) m/z ($[M+H]^+$) calcd for $C_{33}H_{30}ClO_3$: 509.1878. Found: 509.1882.

M.p.: 112-113 ºC.

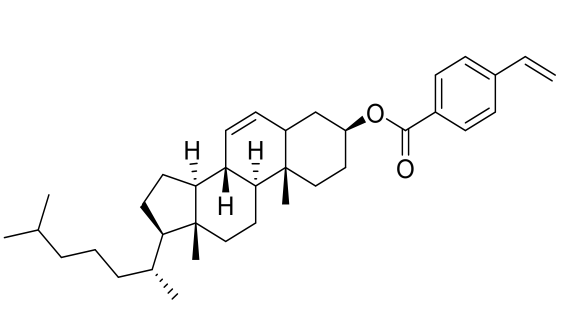

(*3S,8S,9S,10S,13R,14S,17R*)-10,13-Dimethyl-17-((*R*)-6-methylheptan-2-yl)-2,3,4,5,8,9,10,11,12,13,14,15,16,17-tetradecahydro-1*H*-cyclopenta[*a*]phenanthren-3-yl-4-

vinylbenzoate.

The title compound was prepared following the general procedure using 4-vinylbenzoic acid (1.5 g, 10 mmol, 1.0 equiv), cholesterol (5.8 g, 15 mmol, 1.5 equiv). After purification by column chromatography (using 5% ethyl acetate in petroleum ether), the title compound was isolated in 85% yield (4.4 g) as a white solid.

^{1}H NMR (500 MHz, $CDCl_3$): δ 7.99 (d, J = 8.3 Hz, 2H), 7.45 (d, J = 8.3 Hz, 2H), 6.75 (dd, J = 17.6, 10.9 Hz, 1H), 5.86 (d, J = 17.9 Hz, 1H), 5.42 (d, J = 3.8 Hz, 1H), 5.37 (d, J = 11.1 Hz, 1H), 4.95–4.77 (m, 1H), 2.46 (d, J = 7.7 Hz, 2H), 2.07–1.95 (m, 3H), 1.95–1.69 (m, 3H), 1.65–1.42 (m, 7H), 1.42–1.09 (m, 10H), 1.07 (s, 3H), 1.05–0.96 (m, 3H), 0.92 (d, J = 6.5 Hz, 3H), 0.87 (dd, J = 6.6, 2.3 Hz, 6H), 0.69 (s, 3H).

^{13}C NMR (126 MHz, $CDCl_3$): δ 165.91, 141.88, 139.83, 136.24, 130.13, 130.00, 126.16, 122.92, 116.46, 74.71, 56.85, 56.29, 50.20, 42.47, 39.89, 39.67, 38.38, 37.19, 36.81, 36.34, 35.95, 32.09, 32.04, 28.39, 28.17, 28.05, 24.45, 23.98, 22.97, 22.72, 21.21, 19.54, 18.87, 12.02.

HRMS (ESI) m/z ($[M+H]^+$) calcd for $C_{36}H_{53}O_2$: 517.4040. Found: 517.4042.

M.p.: 156-157 ºC.

V. Competition Experiments

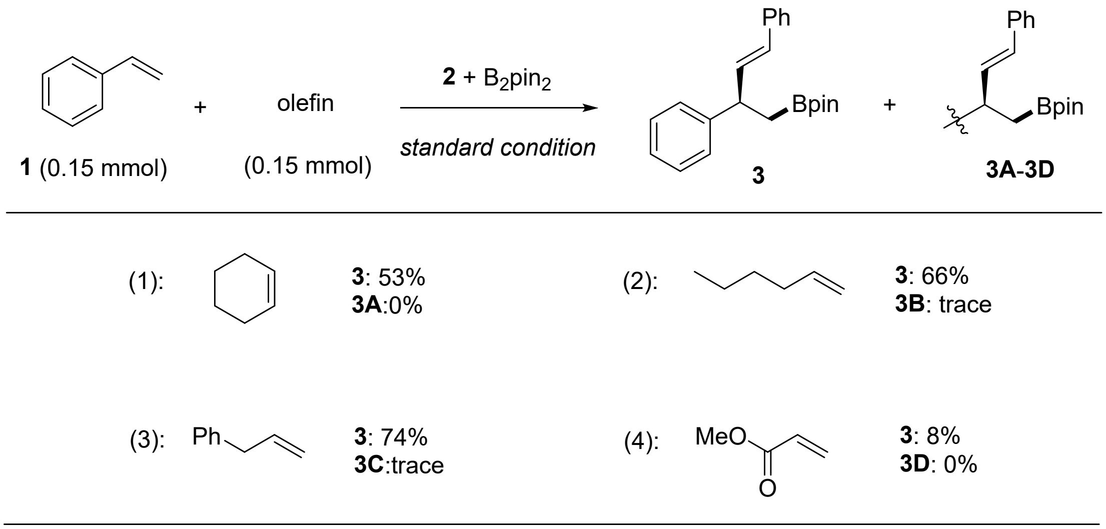

Under N_2 atmosphere, an oven-dried 10 mL reaction tube which equipped with a magnetic stir bar and sealed with a rubber stopper sequentially was added **L1**•$NiBr_2$ (8.6 mg, 0.015 mmol, 10 mol%), LiOMe (8.6 mg, 0.225 mmol, 1.5 equiv), bis(pinacolato)diboron (45.7 mg, 0.180 mmol, 1.2 equiv). Then anhydrous 1,4-dioxane (1 mL), styrene (**1**, 0.150 mmol, 1 equiv), olefin (0.150 mmol, 1 equiv), (*E*)-(2-bromovinyl)benzene (**2**, 25 µL, 0.195 mmol, 1.3 equiv) were added and the mixture was stirred. After 14 h of stirring at 10 °C, the mixture was analyzed by NMR and purified by column chromatography.

VI. Mechanistic Investigations

1. Deuterium Crossover Experiment

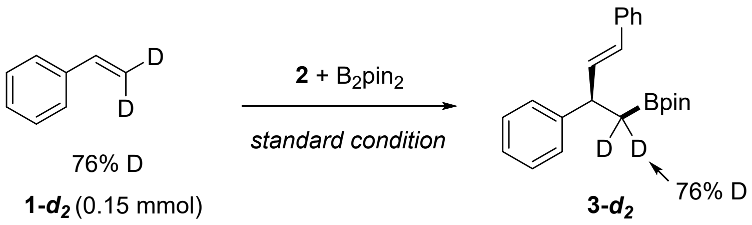

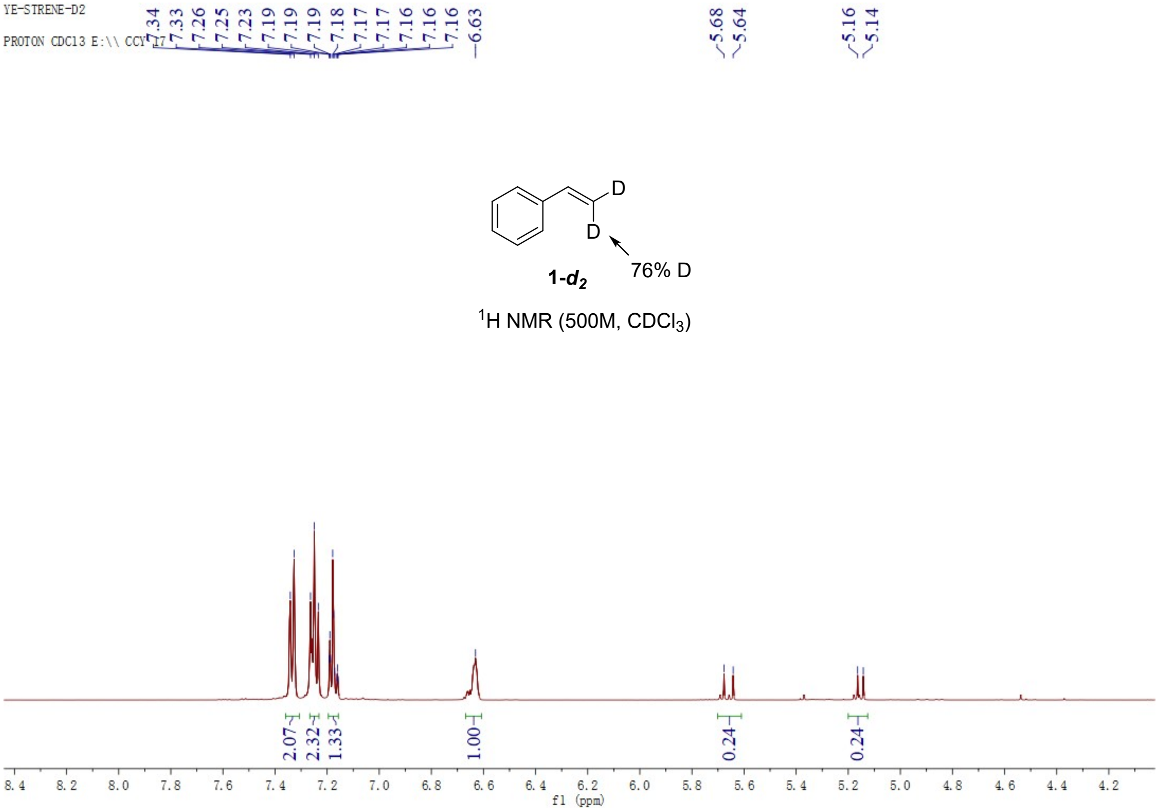
YE-STRENE-D2
PROTON CDCl3 E:\\ CCY 17
D
D
76% D
1-*d*$_2$
^{1}H NMR (500M, $CDCl_3$)
f1 (ppm)

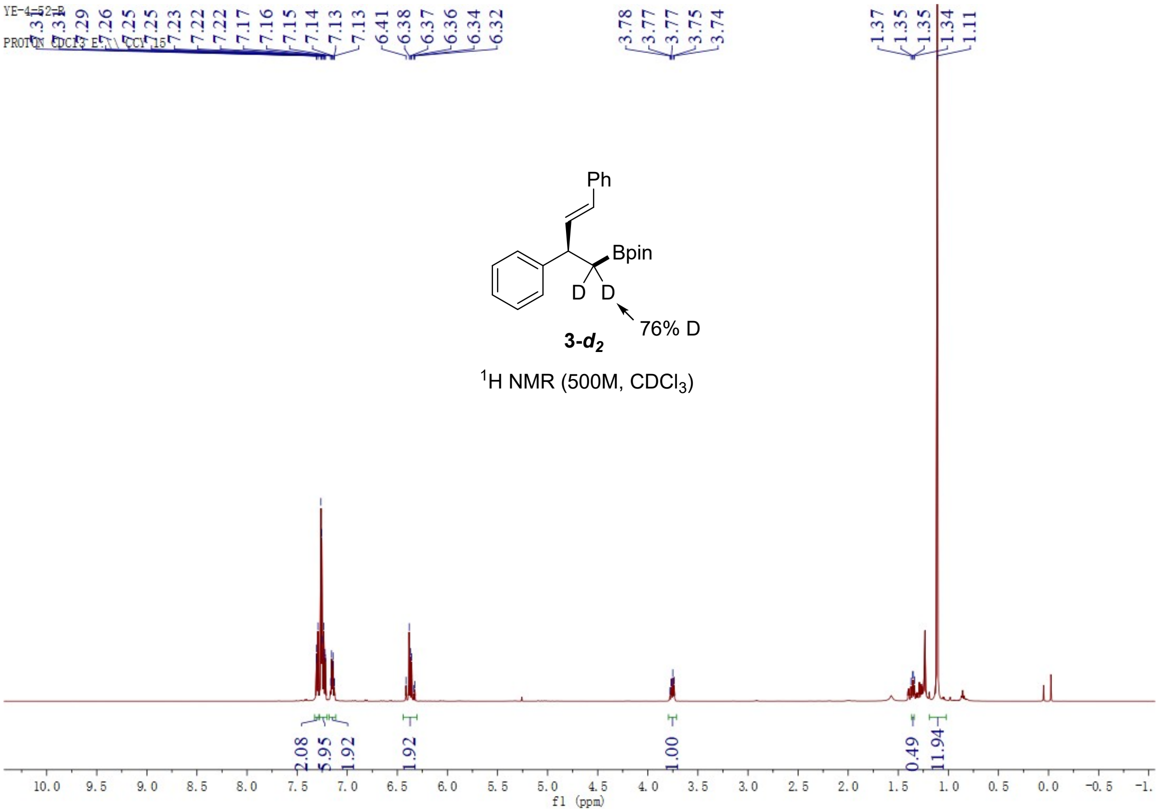
YE-4-52-B
PROTON CDCl3 E:\\ CCY 15
Ph
Bpin
D
D
76% D
3-*d*$_2$
^{1}H NMR (500M, $CDCl_3$)
f1 (ppm)

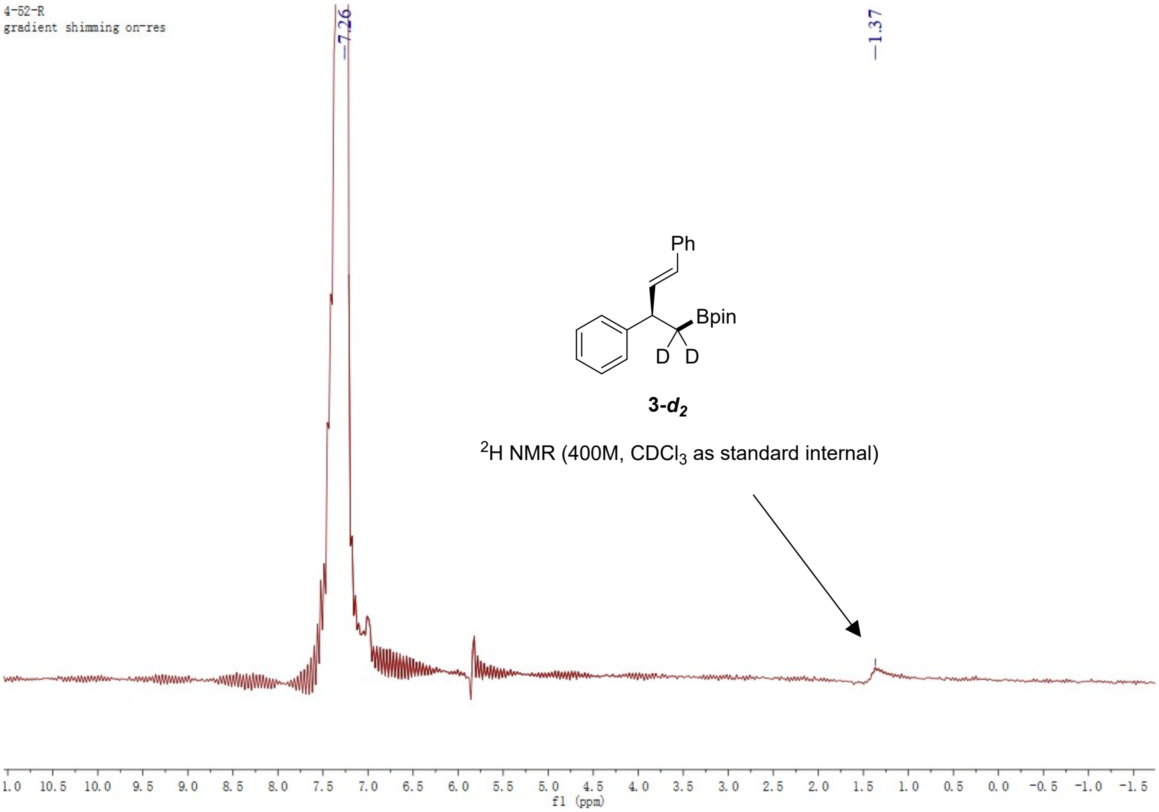

Under N_2 atmosphere, an oven-dried 10 mL reaction tube which equipped with a magnetic stir bar and sealed with a rubber stopper sequentially was added **L1**•$NiBr_2$ (8.6 mg, 0.015 mmol, 10 mol%), LiOMe (8.6 mg, 0.225 mmol, 1.5 equiv), bis(pinacolato)diboron (45.7 mg, 0.180 mmol, 1.2 equiv). Then anhydrous 1,4-dioxane (1 mL), 4-(vinyl-2,2-d_2)benzene[6] (**1-*d*$_2$**, 17 μL, 0.150 mmol, 1 equiv), (*E*)-(2-bromovinyl)benzene (**2**, 25 μL, 0.195 mmol, 1.3 equiv) were added and the mixture was stirred. After 14 h of stirring at 10 °C, the mixture was analyzed by NMR and purified by column chromatography.

2. Intermolecular Protoboration Studies

With respect to the mechanism, during our investigations it was observed that performing the vinylboration of alkene **1** in the presence of MeOH (2 equiv) led to formation of **3** and adduct **61** (Figure S1). This observation led to the hypothesis that addition of Ni(I)-Bpin (**II**) to the alkene occurs to generate benzyl-Ni(I) complex (**III**). This complex can undergo reaction with a vinyl halide to generate **3** or, in the presence of MeOH, undergo protonation to provide **61** according to the catalytic cycle shown in Figure 5. Further support for this catalytic cycle was found was the addition of 5 equiv of MeOH resulted in increased amounts of **61** relative to **3**.

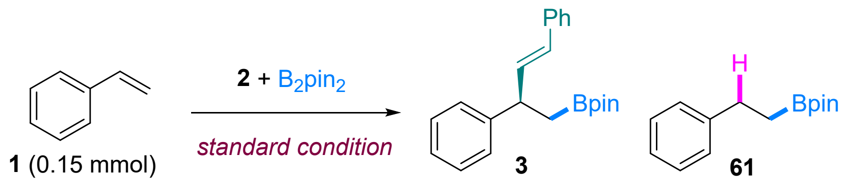

		3	61
**(1)**	no change	89%, 96% ee	trace
**(2)**	with MeOH (2 equiv)	51%, 96% ee	7%
**(3)**	with MeOH (5 equiv)	23%, 96% ee	30%

Figure S1. Intermolecular protoboration studies. (Yields determined by crude ^{1}H NMR using 2,5-dimethylfuran as the internal standard. The *ee* values were determined by HPLC on a chiral stationary phase.)

In addition, reaction of alkenes with polar functional groups such as OH, NH_2, and COOH, which increases the proximity of acidic hydrogens near the Ni-alkyl bond in Ni(I) complex (**III**) and thus give rise to an increasing generation of the protonation adduct, which did indeed result in exclusive formation of protonated product with trace amount of vinylboration product (Figure S2). Notably, the catalytic cycle was completely prevented when the alkenes with COOH group were used.

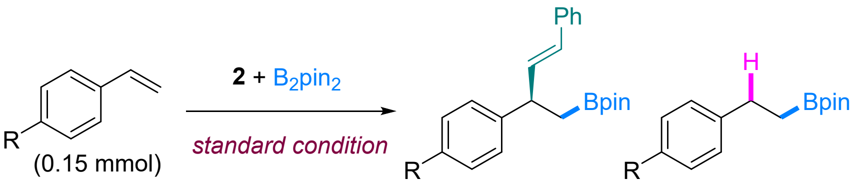

**(1)**	R = CH_2OH	trace	15%
**(2)**	R = NH_2	trace	18%
**(3)**	R = COOH	0%	0%

Figure S2. The alkenes with polar functional groups. (Yields determined by crude ^{1}H NMR using 2,5-dimethylfuran as the internal standard.)

3. Control Experiments

To elucidate the possible reaction mechanism, several control experiments were designed (Figure S3). Firstly, radical clock experiment was carried out, but this reaction did not work with this kind of alkene (eq 1). It may be due to the steric effect, which made it difficult for Ni(I) complex (**III**) reacted with vinyl halide to undergo oxidative addition process. However, in the presence of TEMPO and BHT, the reaction was not inhibited (eq 2-3). The above results suggested long-lived radical

intermediates might not be involved in the reaction processes. When the reaction was carried out under aerobic condition, it did not work (eq 4). These data indicated that the reaction is sensitive to O_2.

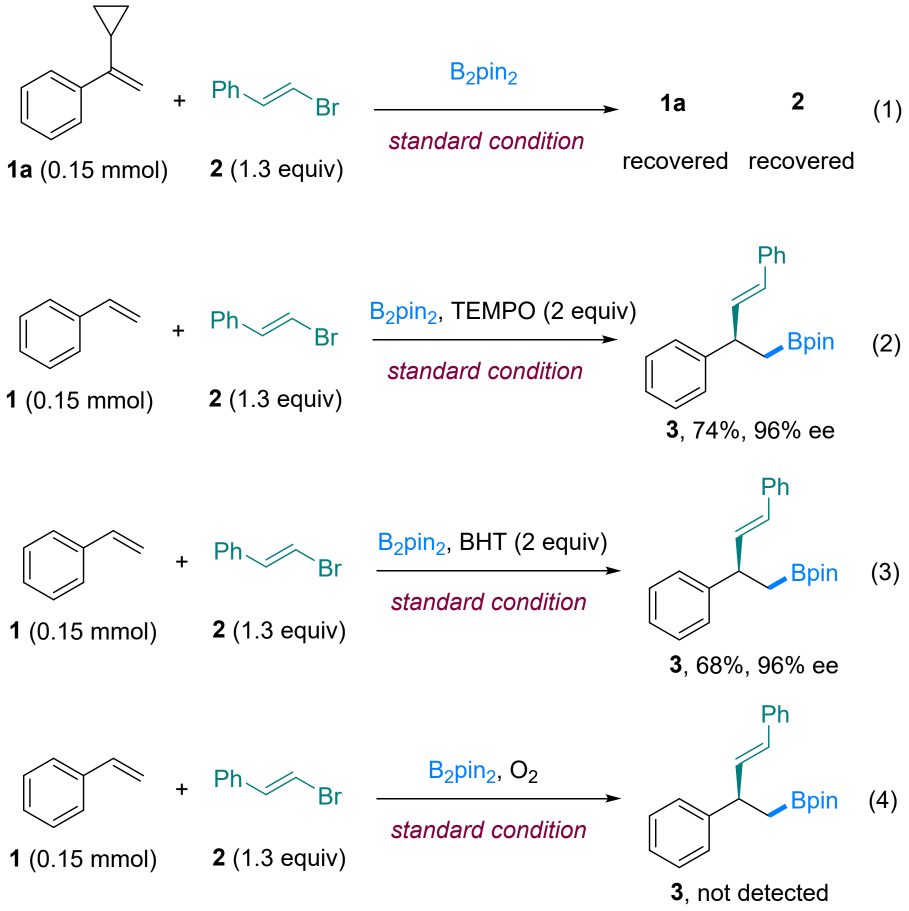

Figure S3. Control experiments. (Yields determined by crude ^{1}H NMR using 2,5-dimethylfuran as the internal standard. The *ee* values were determined by HPLC on a chiral stationary phase.)

VII. References

[1] Serrano, E.; Martin, R. *Angew. Chem., Int. Ed.* **2016**, *55*, 11207.

[2] Suzuki, N.; Hofstra, J.; Poremba, K.; Reisman, S. *Org. Lett.* **2017**, *19*, 2150.

[3] Ramirez, F.; Desai, N. B.; Kelvie, N. *J. Am. Chem. Soc.* **1962**, *84*, 1745.

[4] Abbas, S.; Hayes, C. J.; Worden, S. *Tetrahedron Lett.* **2000**, *41*, 3215.

[5] Dolby, L. J.; Wilkins, D. C.; Frey, T. G. *J. Org. Chem.* **1966**, *31*, 1110.

[6] Yao, Y.-H.; Yang, H.-Y.; Chen, M.; Wu, F.; Xu, X.-X.; Guan, Z.-H. *J. Am. Chem. Soc.* **2021**, *143*, 85.

VIII. Spectral Data (NMR Spectrum)

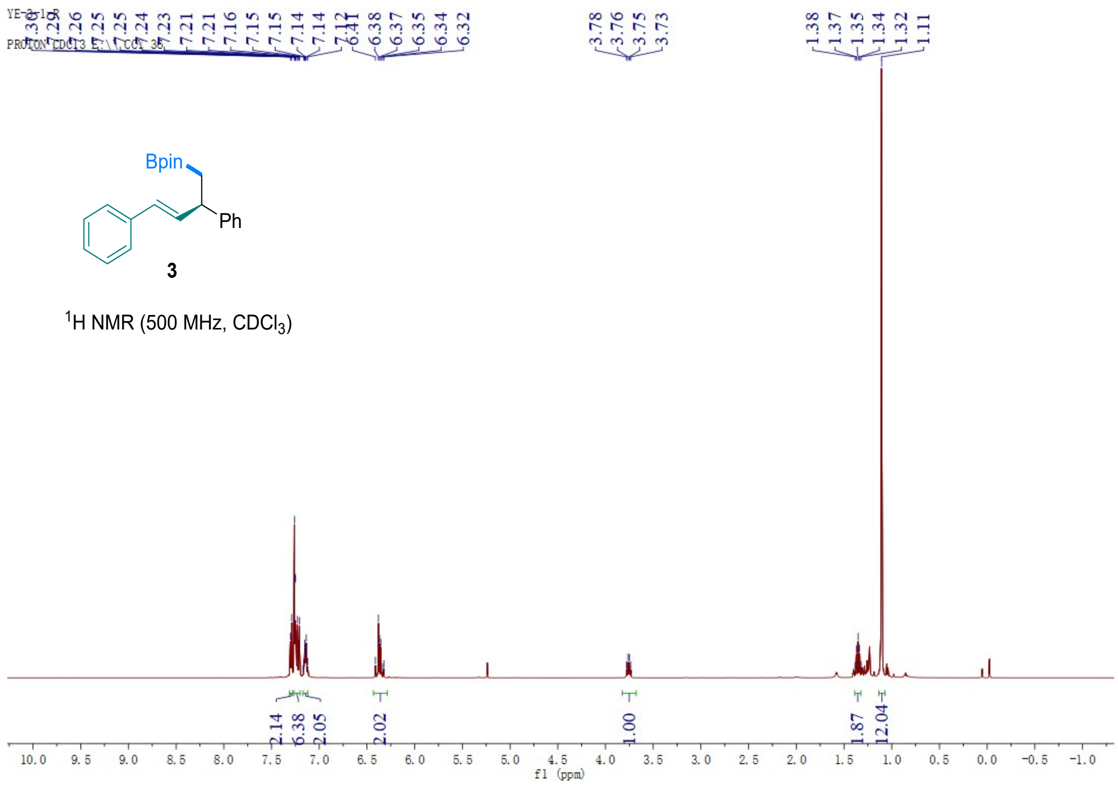

3

^{1}H NMR (500 MHz, $CDCl_3$)

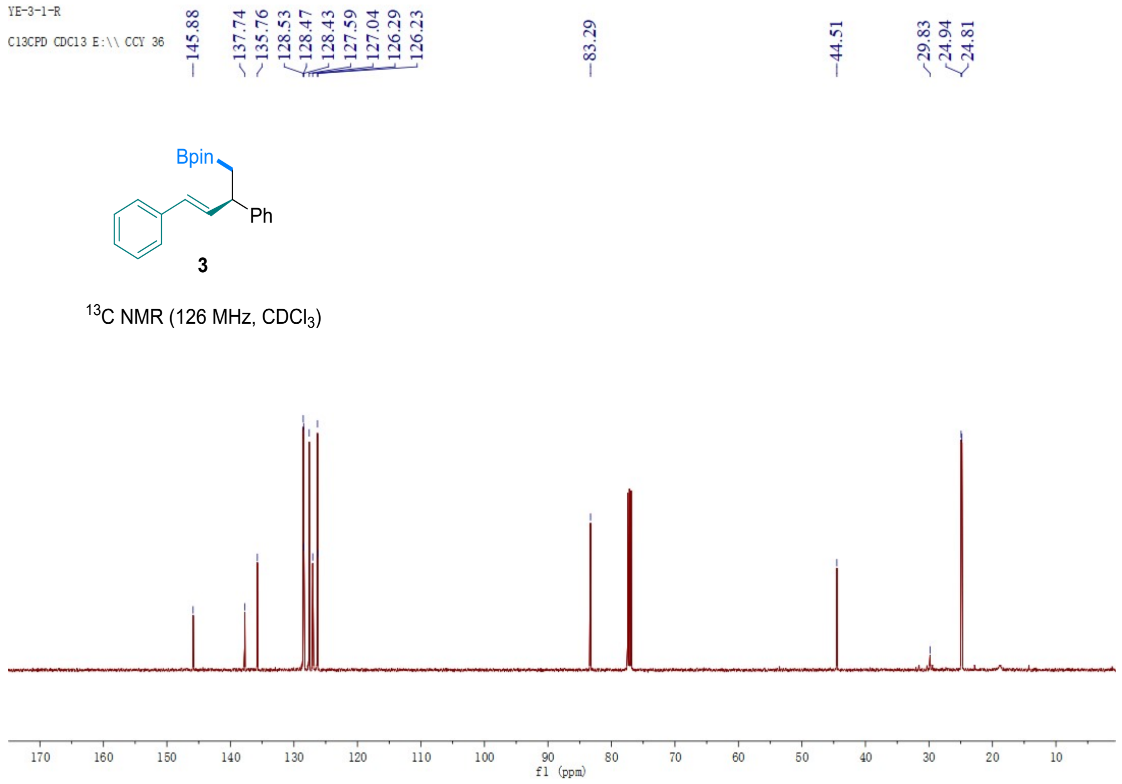

3

^{13}C NMR (126 MHz, $CDCl_3$)

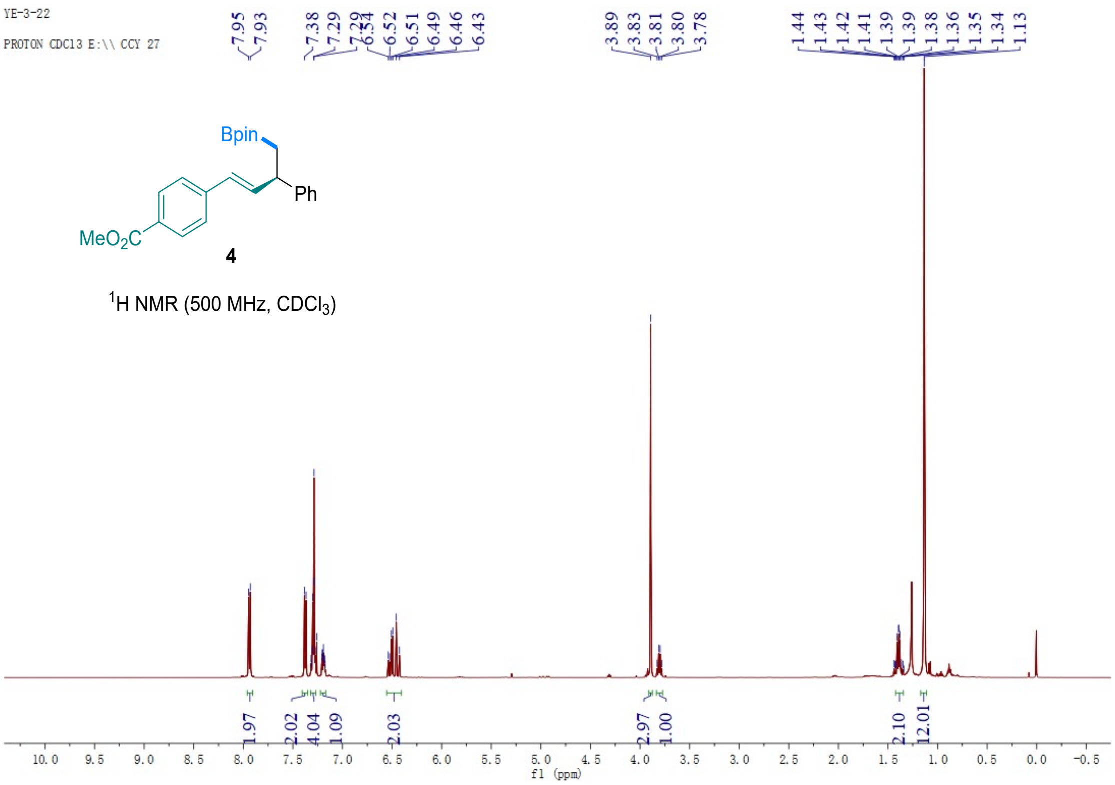
YE-3-22
PROTON CDCl3 E:\\ CCY 27
Bpin
Ph
MeO2C
4
1H NMR (500 MHz, CDCl3)
f1 (ppm)

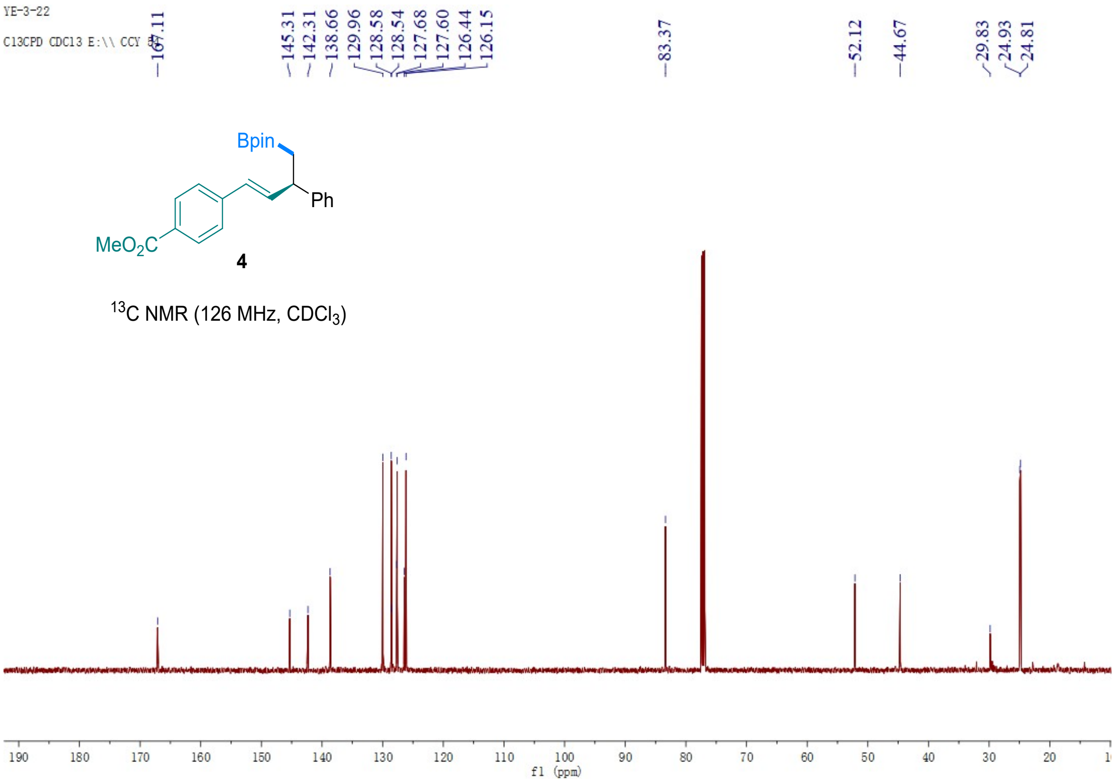
YE-3-22
C13CPD CDCl3 E:\\ CCY
Bpin
Ph
MeO2C
4
13C NMR (126 MHz, CDCl3)
f1 (ppm)

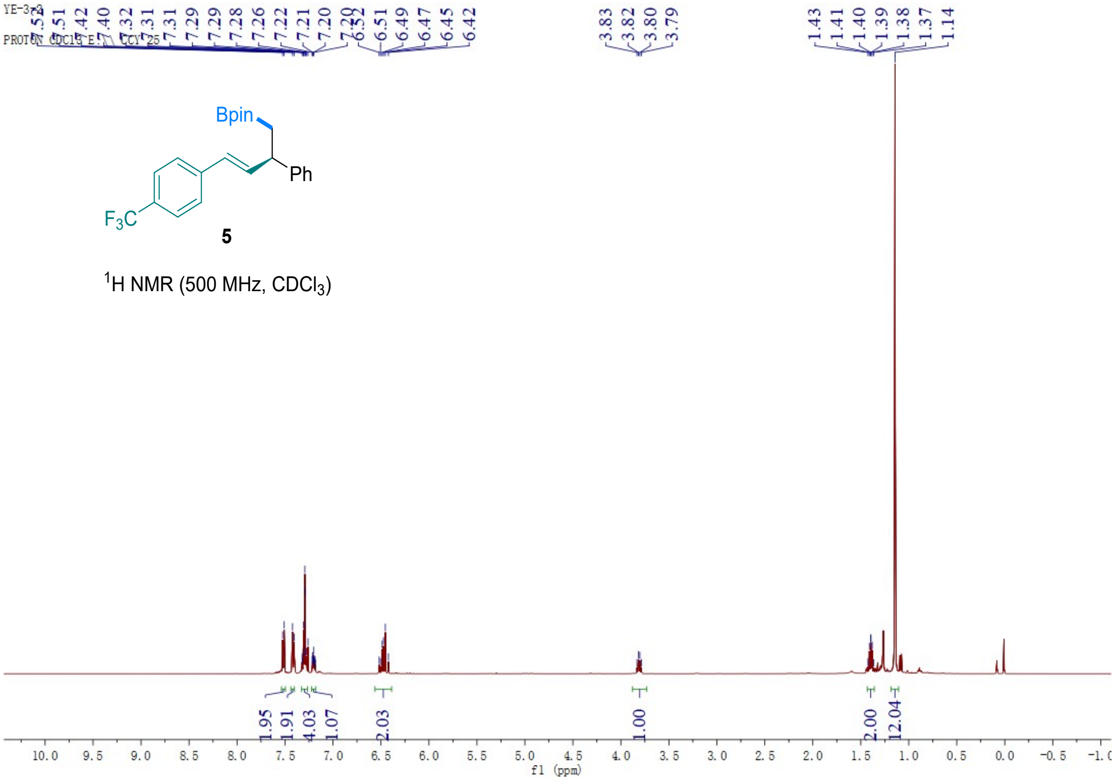
YE-3-2
PROTON CDCl3 E:\\ CCY 25
7.52
7.51
7.42
7.40
7.32
7.31
7.31
7.29
7.29
7.28
7.26
7.22
7.21
7.20
7.20
6.52
6.51
6.49
6.47
6.45
6.42
3.83
3.82
3.80
3.79
1.43
1.41
1.40
1.39
1.38
1.37
1.14
Bpin
Ph
F_3C
5
^1H NMR (500 MHz, $CDCl_3$)
1.95
1.91
4.03
1.07
2.03
1.00
2.00
12.04
f1 (ppm)

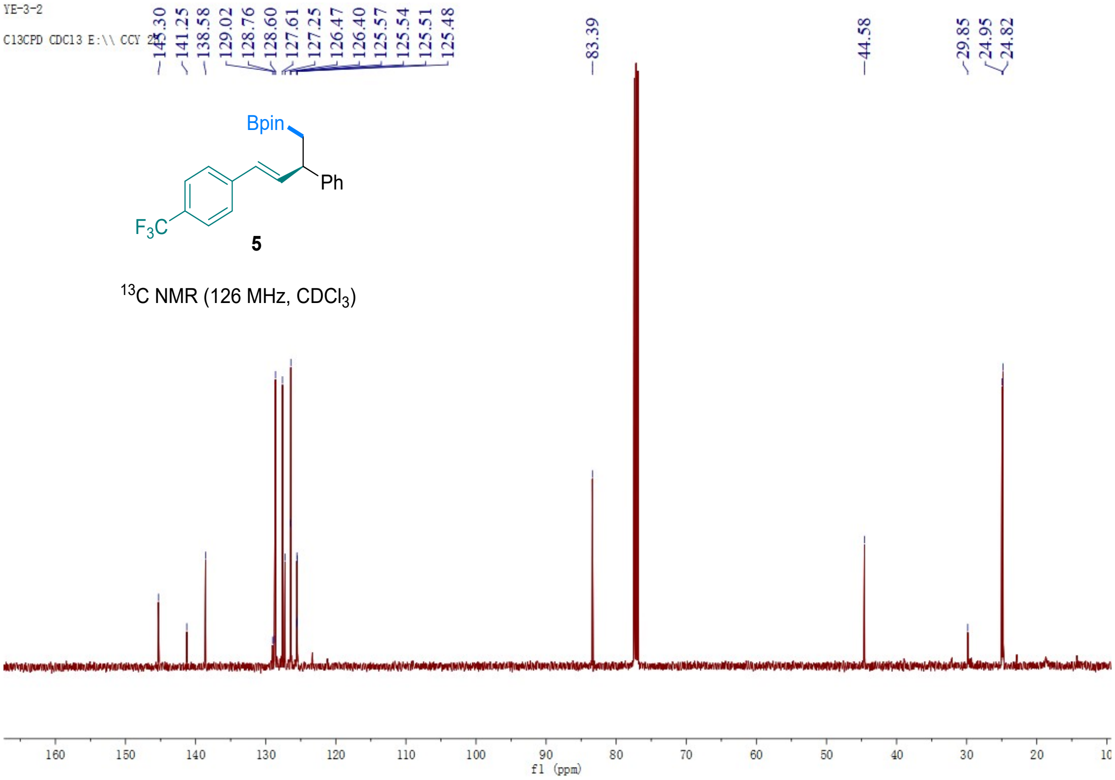
YE-3-2
C13CPD CDCl3 E:\\ CCY 25
145.30
141.25
138.58
129.02
128.76
128.60
127.61
127.25
126.47
126.40
125.57
125.54
125.51
125.48
83.39
44.58
29.85
24.95
24.82
Bpin
Ph
F_3C
5
^{13}C NMR (126 MHz, $CDCl_3$)
f1 (ppm)

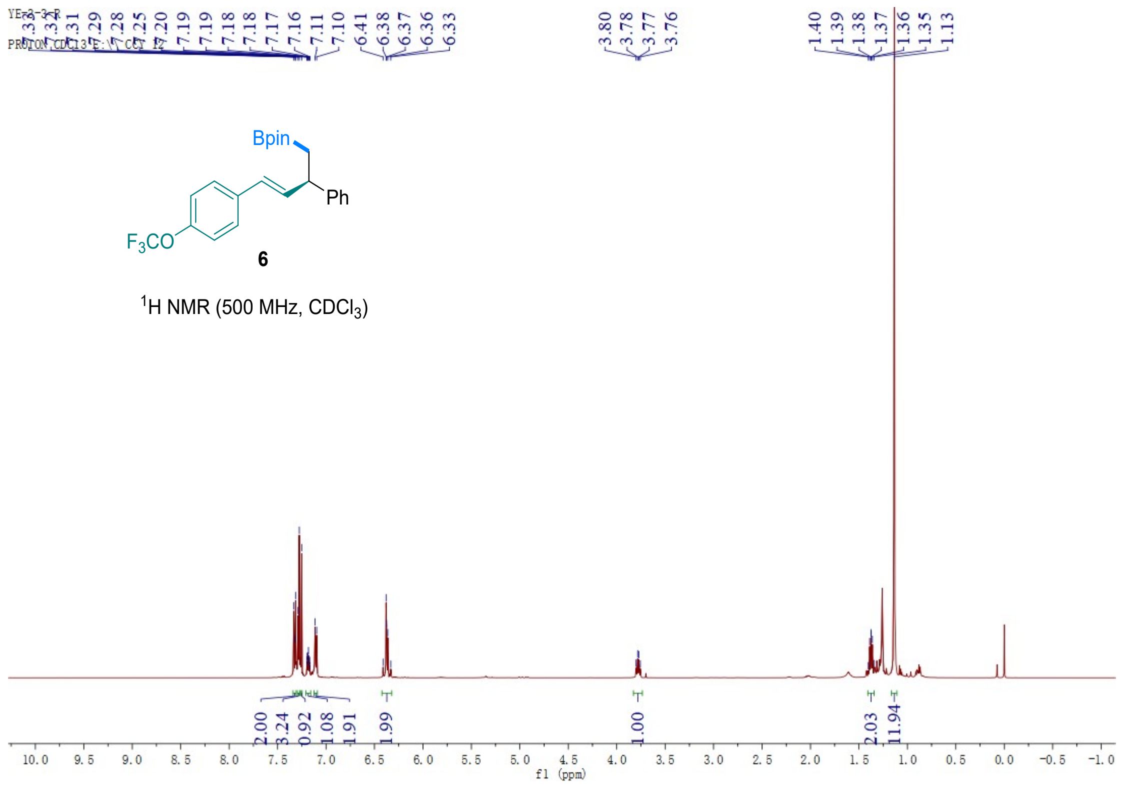
YE-3-3-R
7.33
7.32
7.31
7.29
7.28
7.25
7.20
7.19
7.19
7.18
7.18
7.17
7.16
7.11
7.10
6.41
6.38
6.37
6.36
6.33
3.80
3.78
3.77
3.76
1.40
1.39
1.38
1.37
1.36
1.35
1.13
Bpin
Ph
F_3CO
6
^1H NMR (500 MHz, $CDCl_3$)
2.00
3.24
0.92
1.08
1.91
1.99
1.00
2.03
11.94
f1 (ppm)

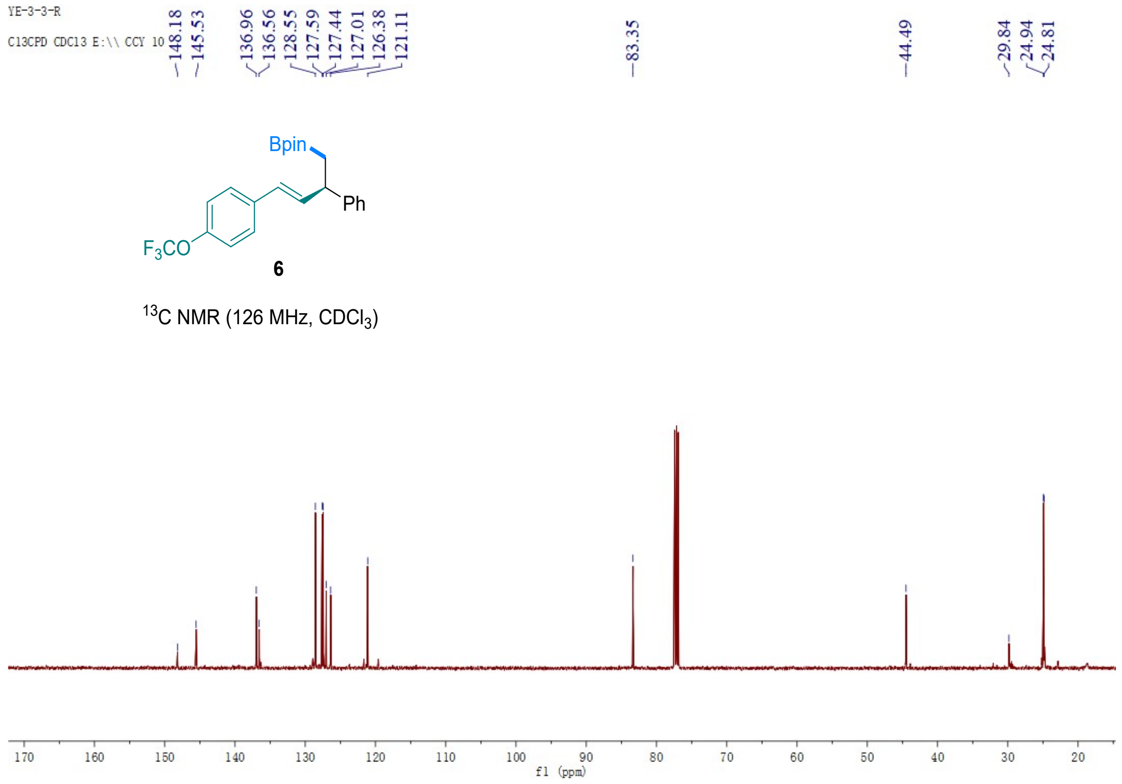
YE-3-3-R
C13CPD CDCl3 E:\\ CCY 10
148.18
145.53
136.96
136.56
128.55
127.59
127.44
127.01
126.38
121.11
83.35
44.49
29.84
24.94
24.81
Bpin
Ph
F_3CO
6
^{13}C NMR (126 MHz, $CDCl_3$)
f1 (ppm)

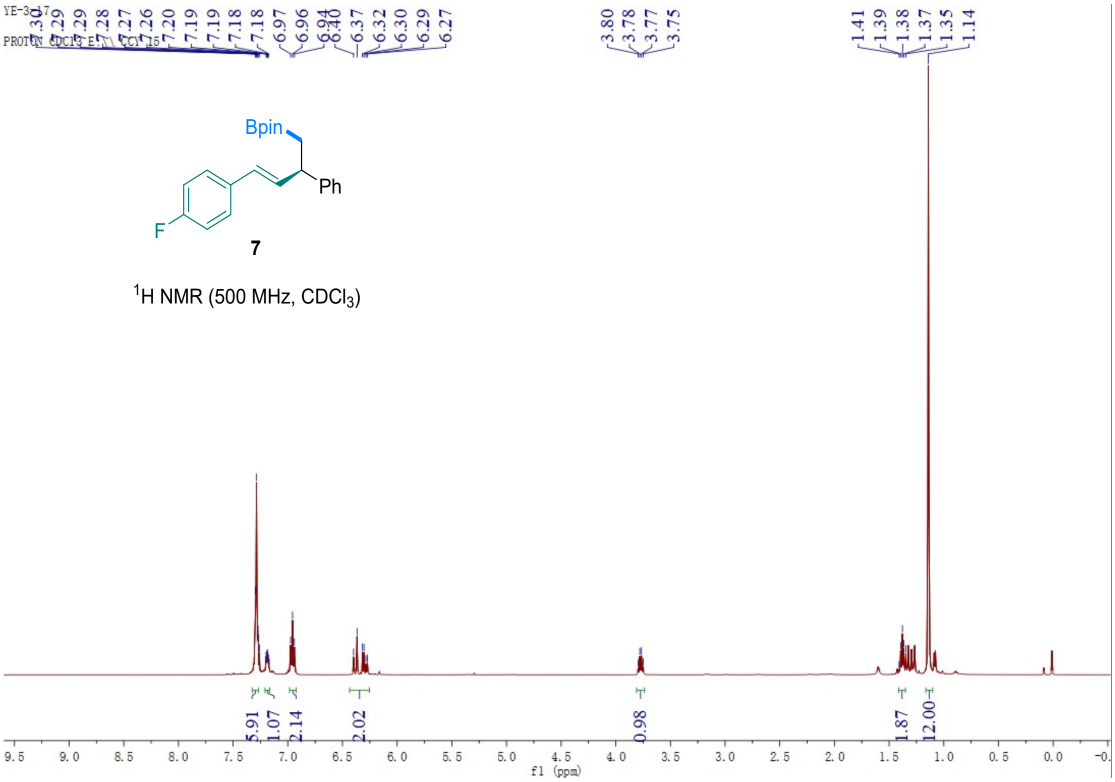
YE-3-17
PROTON CDCl3 E:\\ CCY 15
7.31 7.29 7.29 7.28 7.27 7.27 7.26 7.20 7.19 7.19 7.18 7.18 6.97 6.96 6.94 6.40 6.37 6.32 6.30 6.29 6.27
3.80 3.78 3.77 3.75
1.41 1.39 1.38 1.37 1.35 1.14
Bpin
Ph
F
7
1H NMR (500 MHz, CDCl3)
5.91 1.07 2.14 2.02 0.98 1.87 12.00
f1 (ppm)

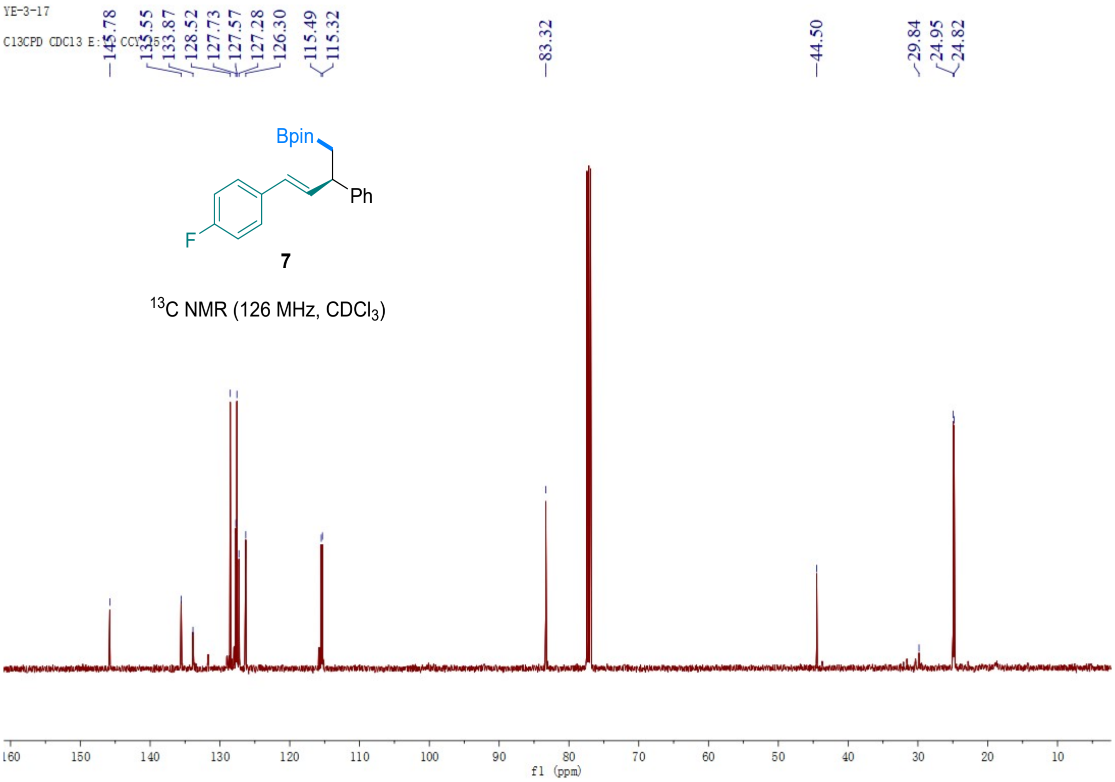
YE-3-17
C13CPD CDCl3 E:\\ CCY 15
145.78 135.55 133.87 128.52 127.73 127.57 127.28 126.30 115.49 115.32
83.32
44.50
29.84 24.95 24.82
Bpin
Ph
F
7
13C NMR (126 MHz, CDCl3)
f1 (ppm)

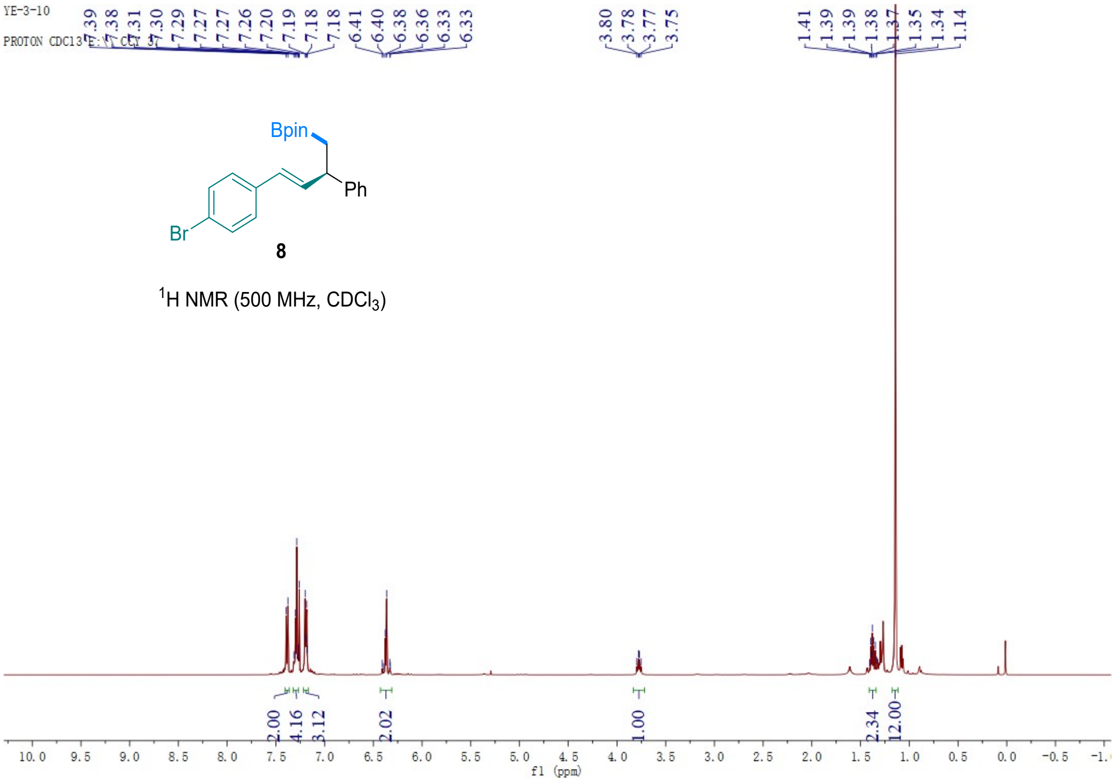

^{1}H NMR (500 MHz, $CDCl_3$)

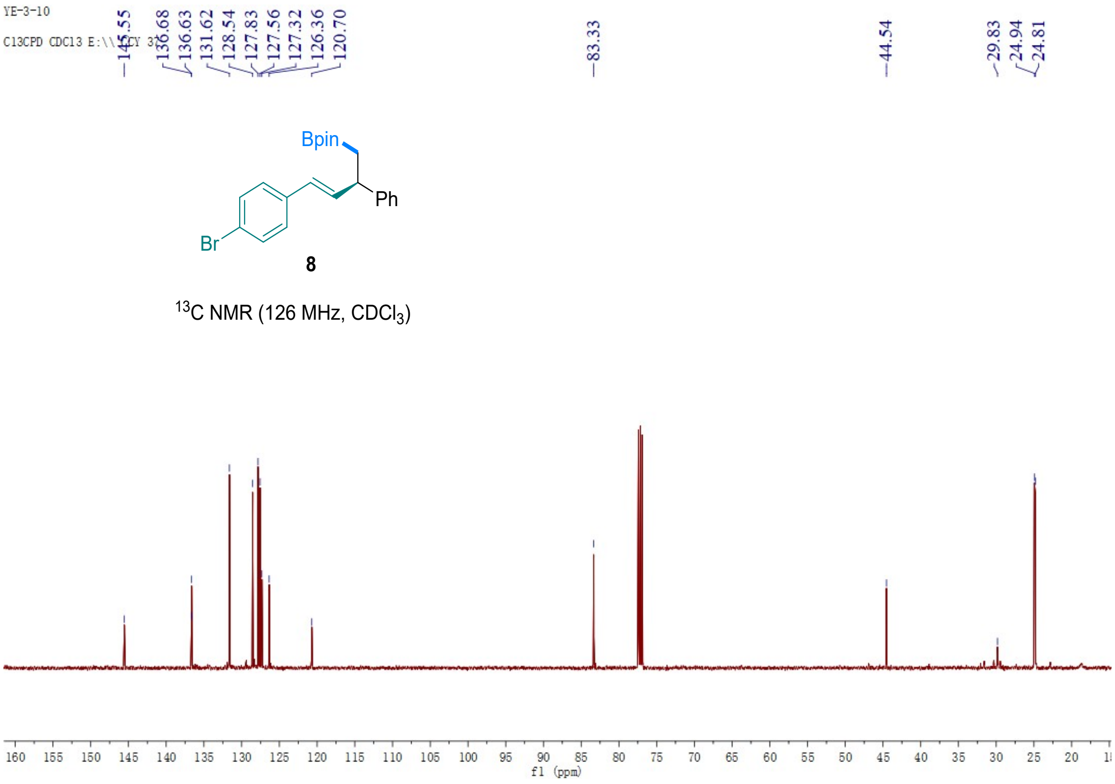

^{13}C NMR (126 MHz, $CDCl_3$)

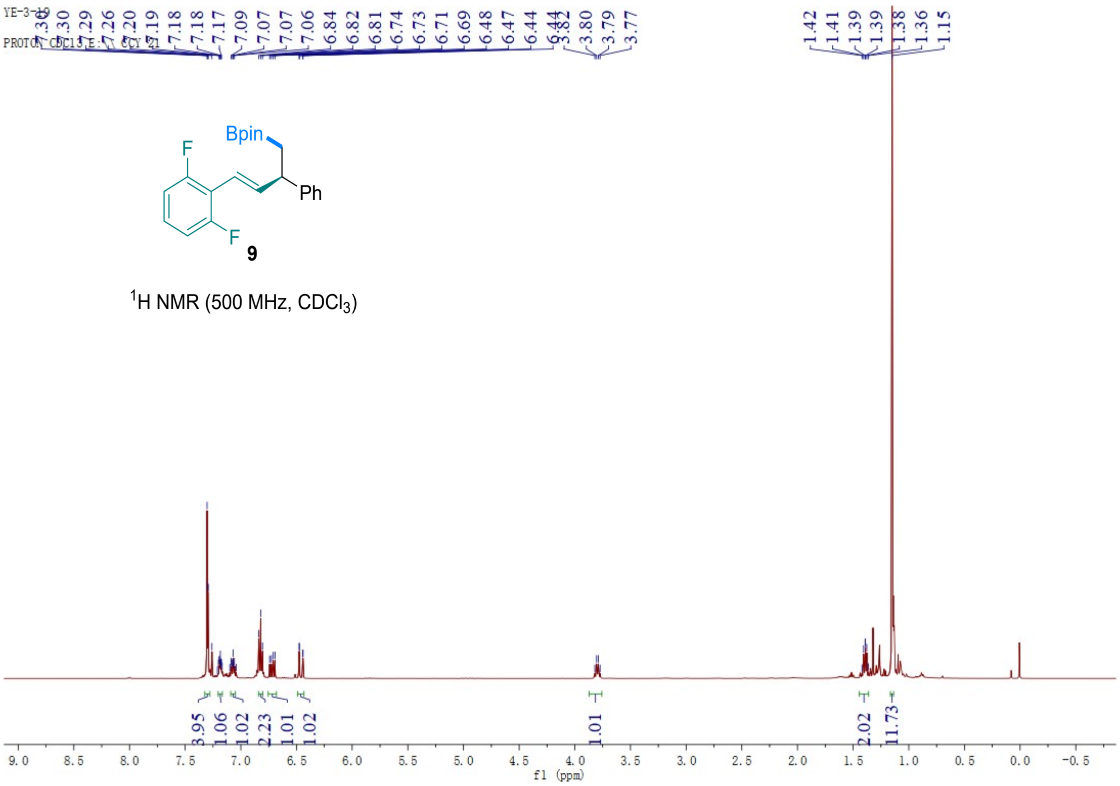

^{1}H NMR (500 MHz, $CDCl_3$)

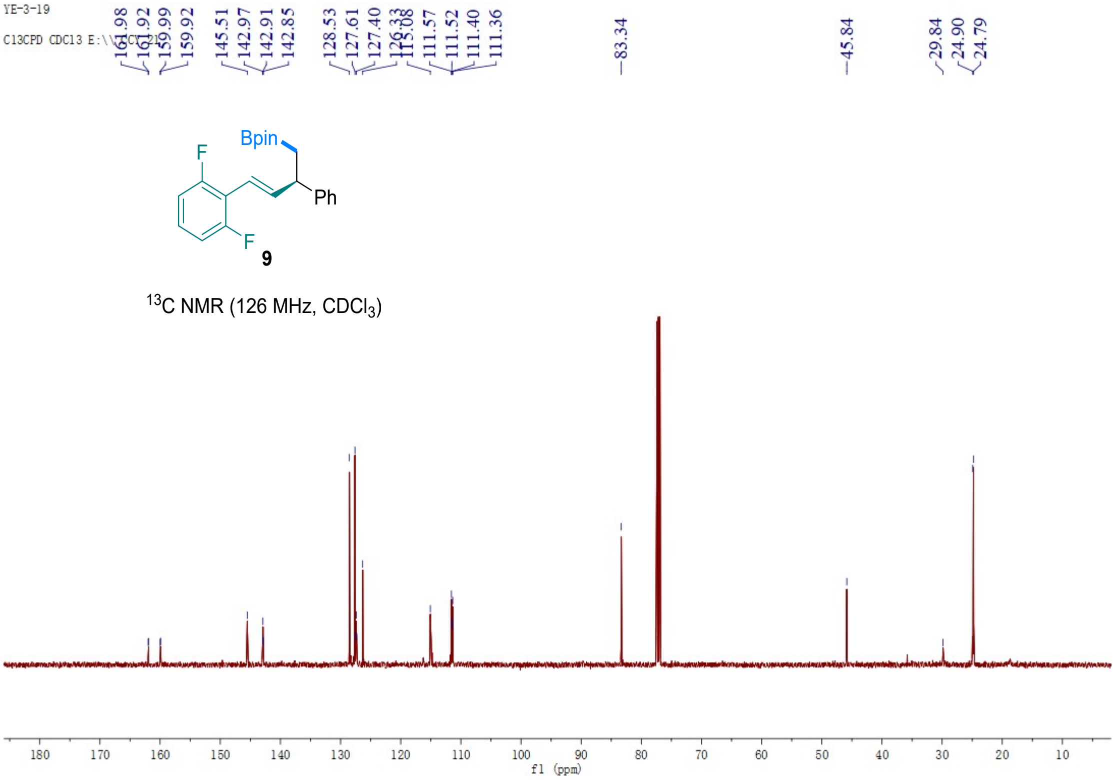

^{13}C NMR (126 MHz, $CDCl_3$)

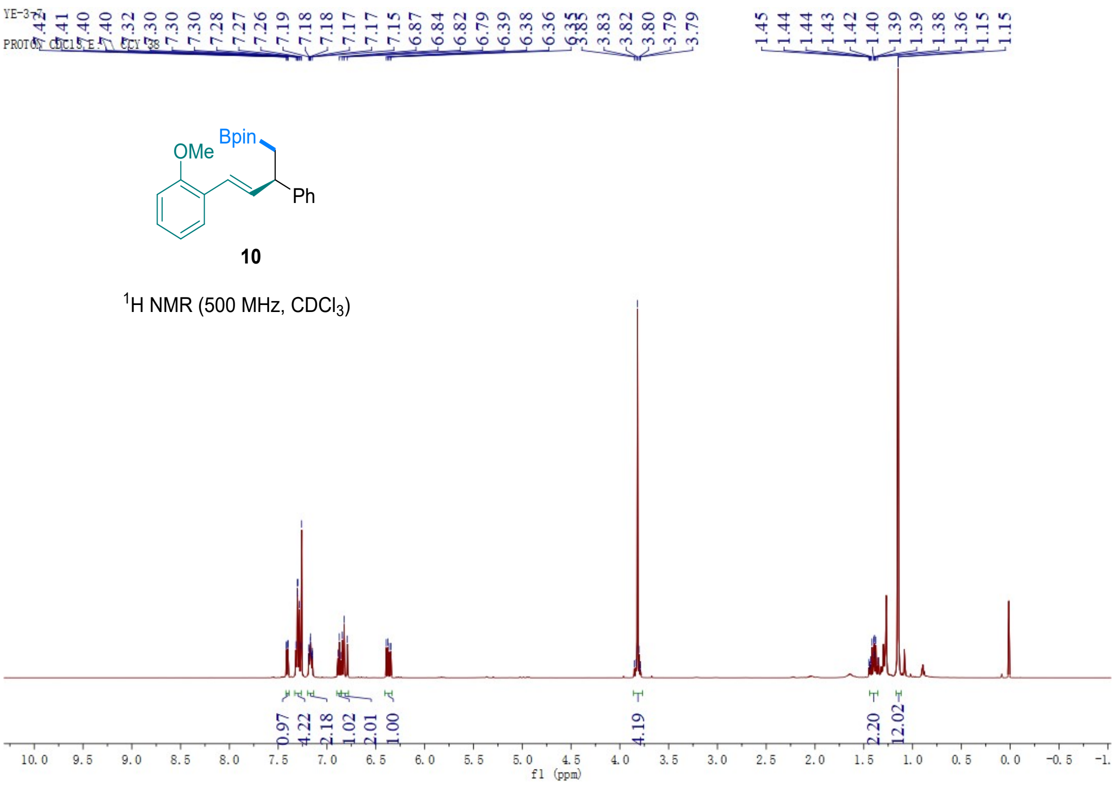
YE-3-7
7.42 7.41 7.40 7.40 7.32 7.30 7.30 7.30 7.28 7.27 7.26 7.19 7.18 7.18 7.17 7.17 7.15 6.87 6.84 6.82 6.79 6.39 6.38 6.36 6.35 3.85 3.83 3.82 3.80 3.79 3.79
1.45 1.44 1.44 1.43 1.42 1.40 1.39 1.39 1.38 1.36 1.15 1.15
OMe
Bpin
Ph
10
1H NMR (500 MHz, CDCl3)
0.97 4.22 2.18 1.02 2.01 1.00 4.19 2.20 12.02
f1 (ppm)

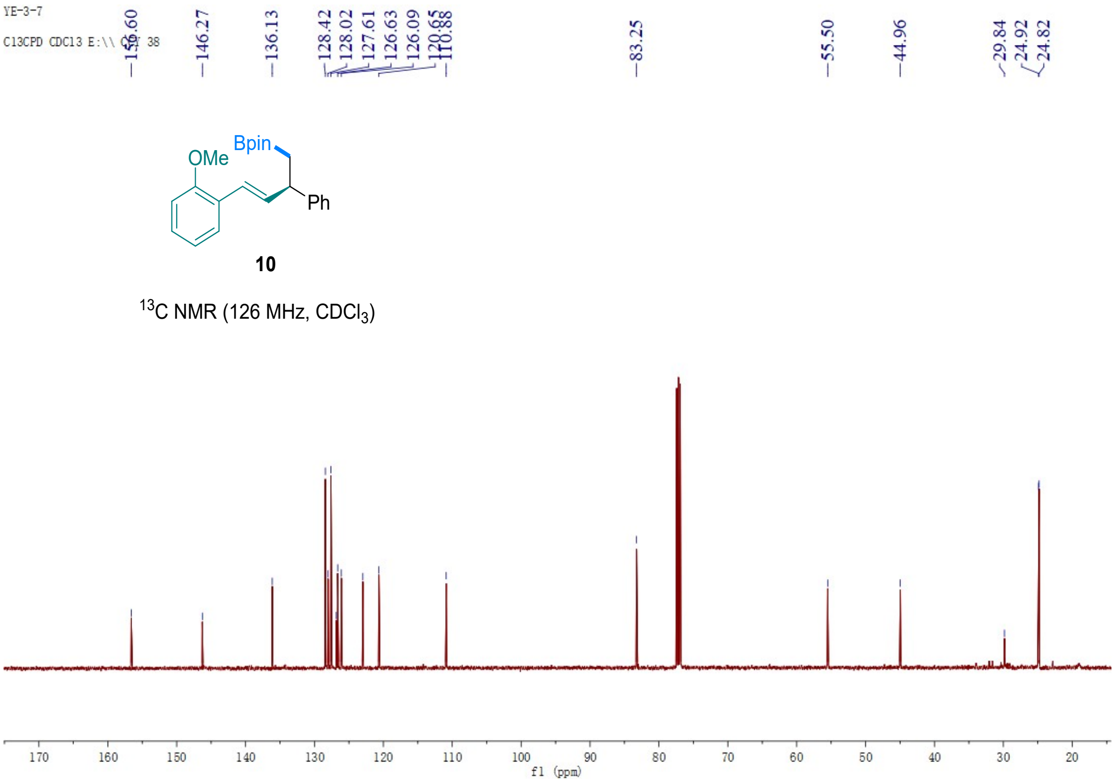
YE-3-7
146.27 136.13 128.42 128.02 127.61 126.63 126.09 110.88 83.25 55.50 44.96 29.84 24.92 24.82
OMe
Bpin
Ph
10
13C NMR (126 MHz, CDCl3)
f1 (ppm)

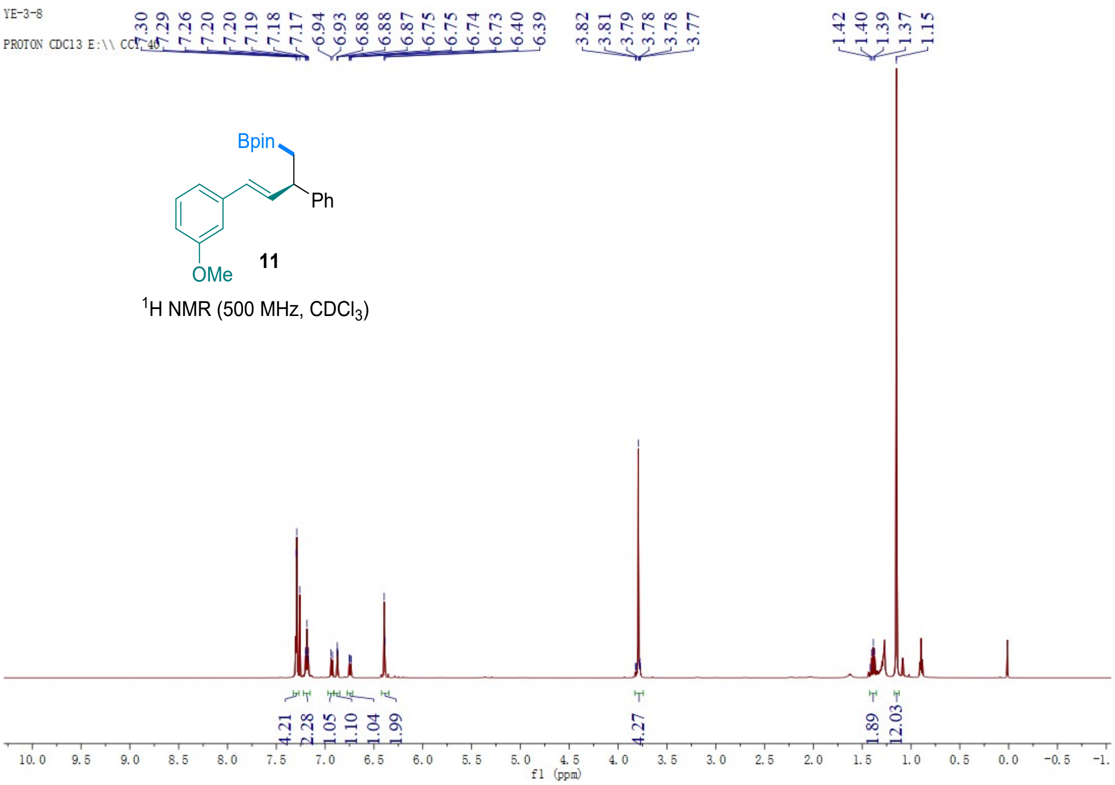
YE-3-8
PROTON CDCl3 E:\\ CC\ 40
Bpin
Ph
OMe
11
4.21
2.28
1.05
1.10
1.04
1.99
4.27
1.89
12.03
f1 (ppm)

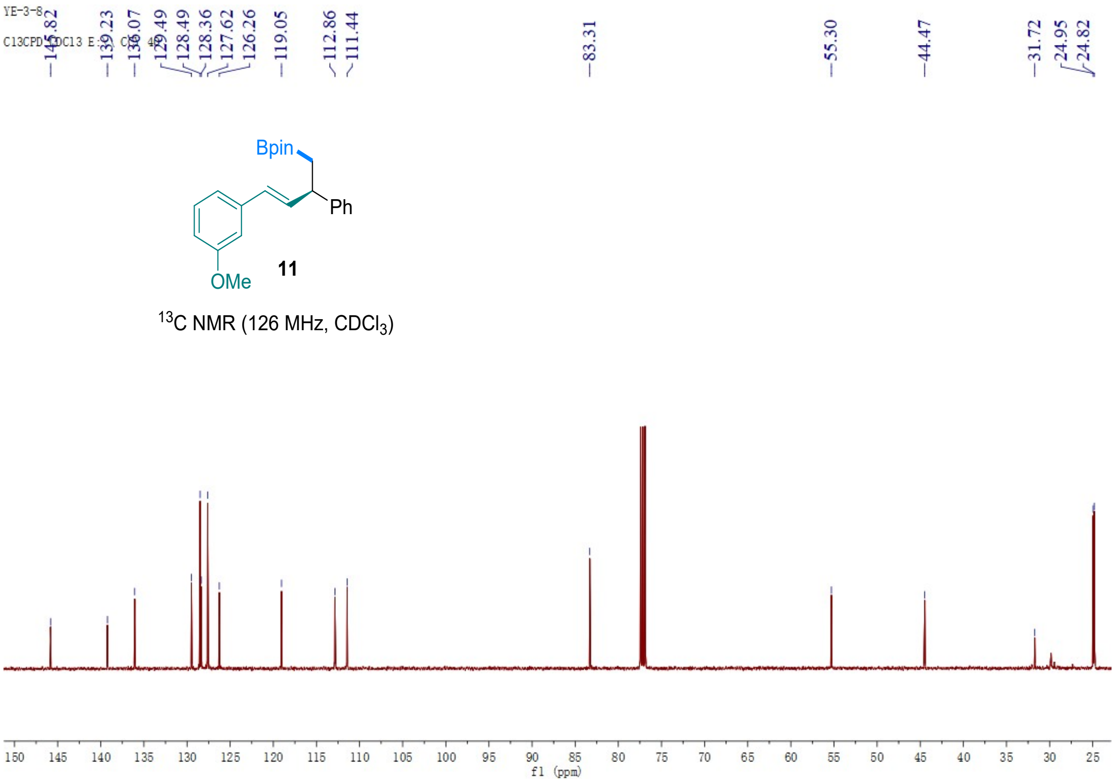
YE-3-8
C13CPD CDCl3
Bpin
Ph
OMe
11
f1 (ppm)

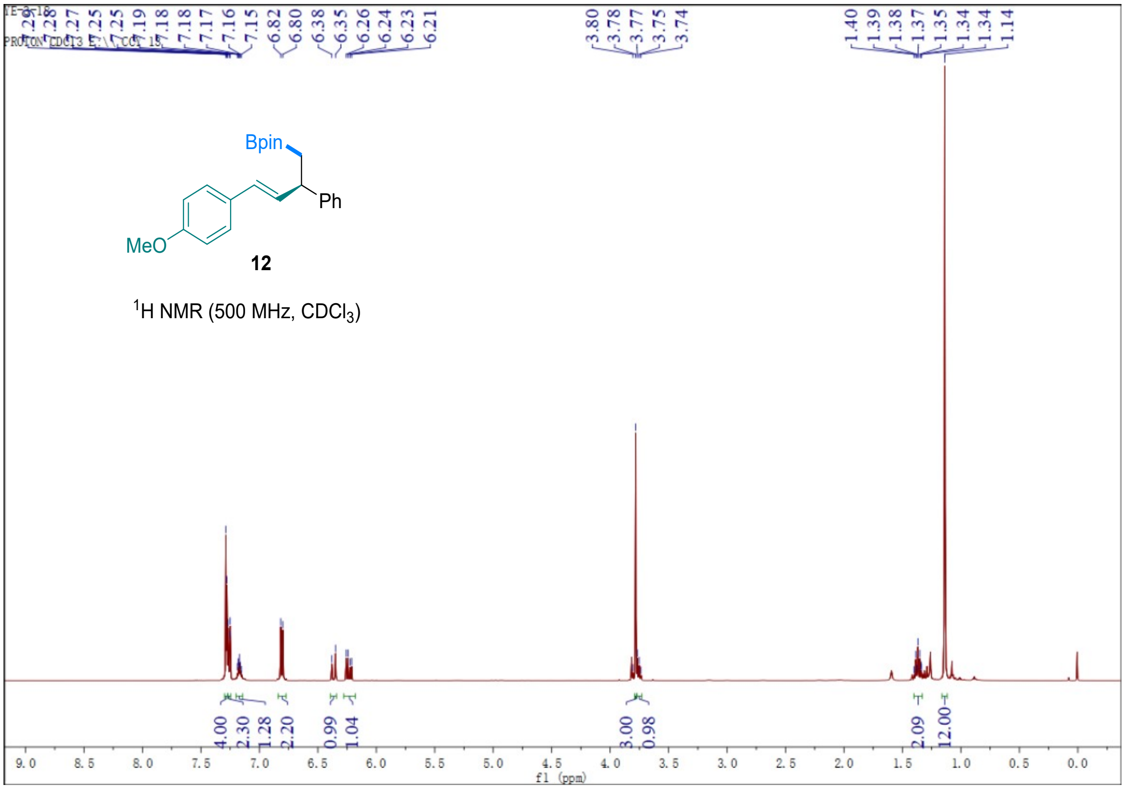
YE-3-18
PROTON CDCl3 E:\\ CCY 15
7.28 7.27 7.25 7.25 7.19 7.18 7.17 7.16 7.15 6.82 6.80 6.38 6.35 6.26 6.24 6.23 6.21
3.80 3.78 3.77 3.75 3.74
1.40 1.39 1.38 1.37 1.35 1.34 1.34 1.14
Bpin
Ph
MeO
12
^{1}H NMR (500 MHz, $CDCl_3$)
4.00 2.30 1.28 2.20 0.99 1.04 3.00 0.98 2.09 12.00
f1 (ppm)

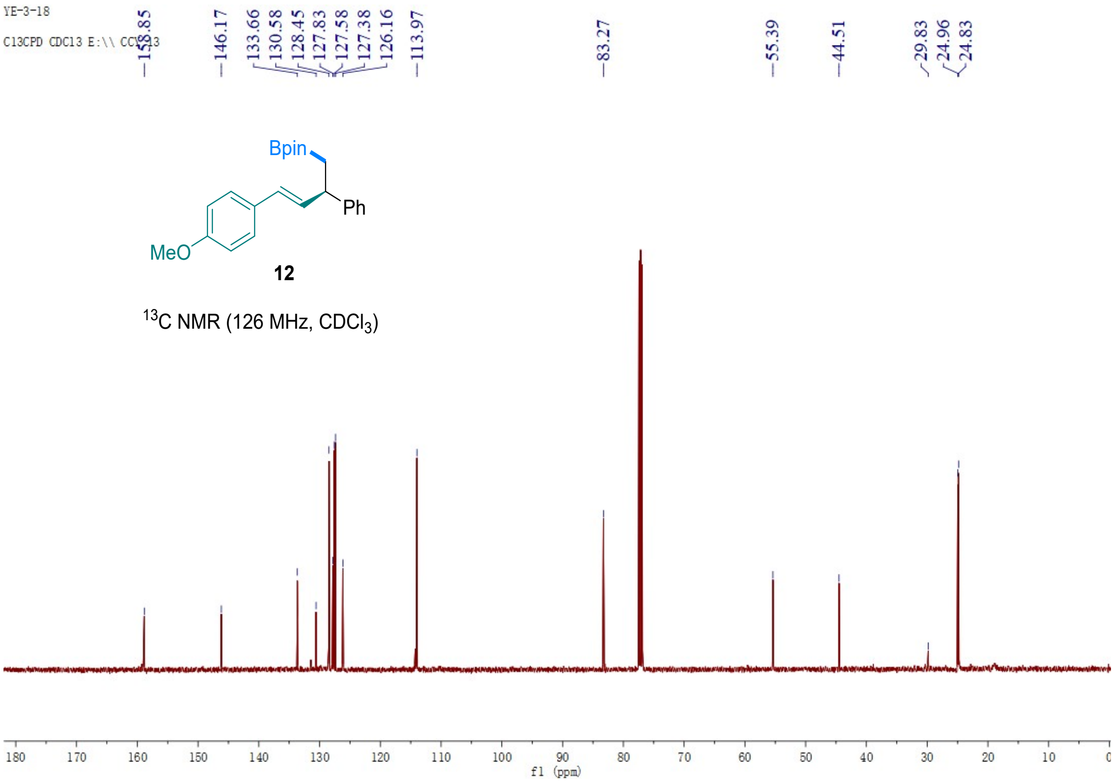
YE-3-18
C13CPD CDCl3 E:\\ CCY 13
158.85 146.17 133.66 130.58 128.45 127.83 127.58 127.38 126.16 113.97 83.27 55.39 44.51 29.83 24.96 24.83
Bpin
Ph
MeO
12
^{13}C NMR (126 MHz, $CDCl_3$)
f1 (ppm)

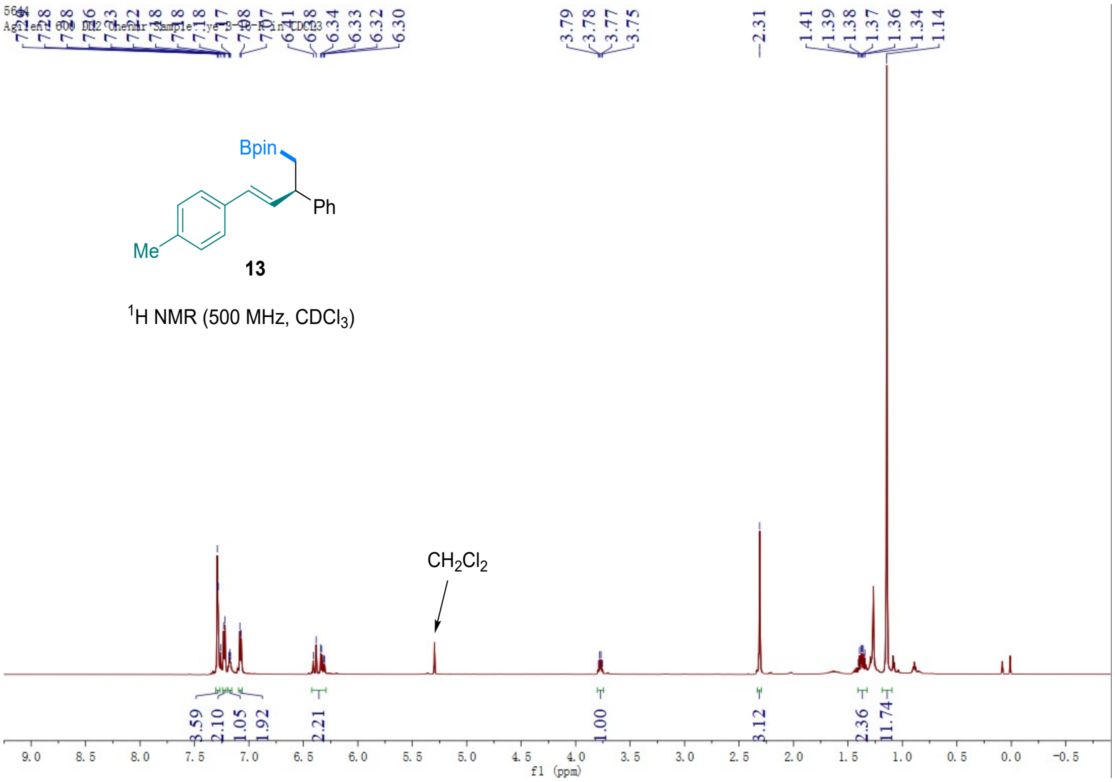

7.28 7.28 7.26 7.23 7.22 7.18 7.18 7.18 7.17 7.08 7.07 6.41 6.38 6.34 6.33 6.32 6.30
3.79 3.78 3.77 3.75
2.31
1.41 1.39 1.38 1.37 1.36 1.34 1.14
Bpin
Ph
Me
13
^{1}H NMR (500 MHz, $CDCl_3$)
CH_2Cl_2
3.59 2.10 1.05 1.92 2.21 1.00 3.12 2.36 11.74
f1 (ppm)

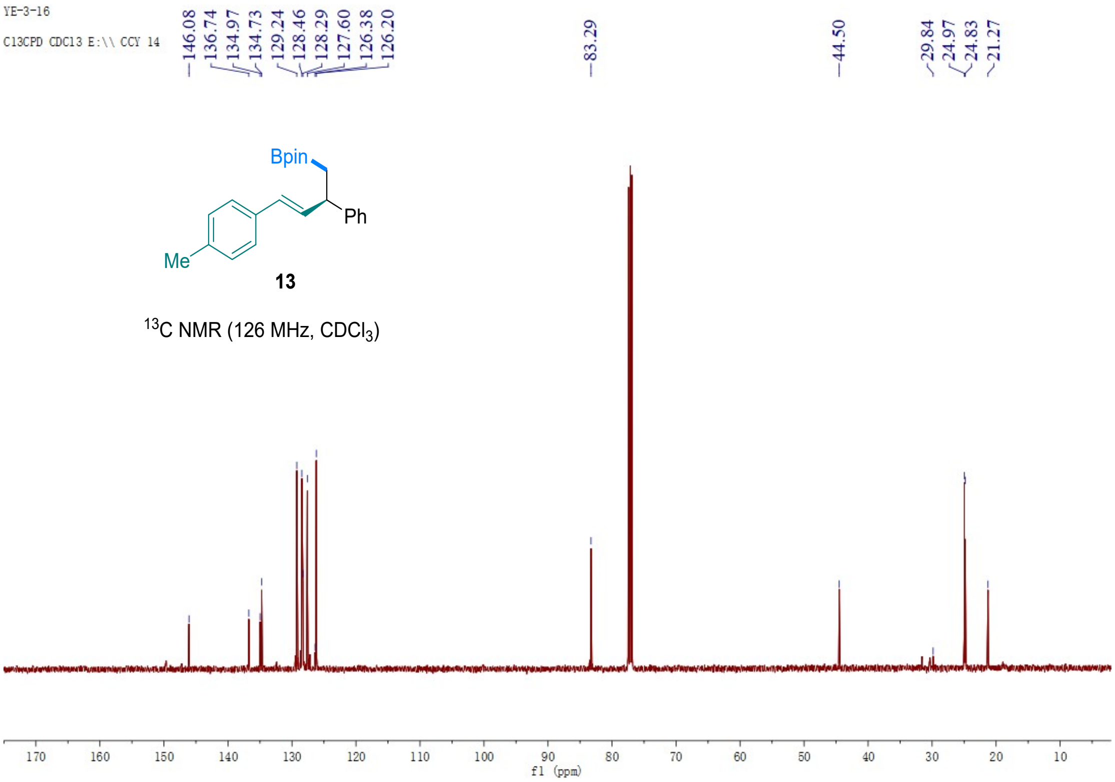

YE-3-16
C13CPD CDCl3 E:\\ CCY 14
146.08 136.74 134.97 134.73 129.24 128.46 128.29 127.60 126.38 126.20
83.29
44.50
29.84 24.97 24.83 21.27
Bpin
Ph
Me
13
^{13}C NMR (126 MHz, $CDCl_3$)
f1 (ppm)

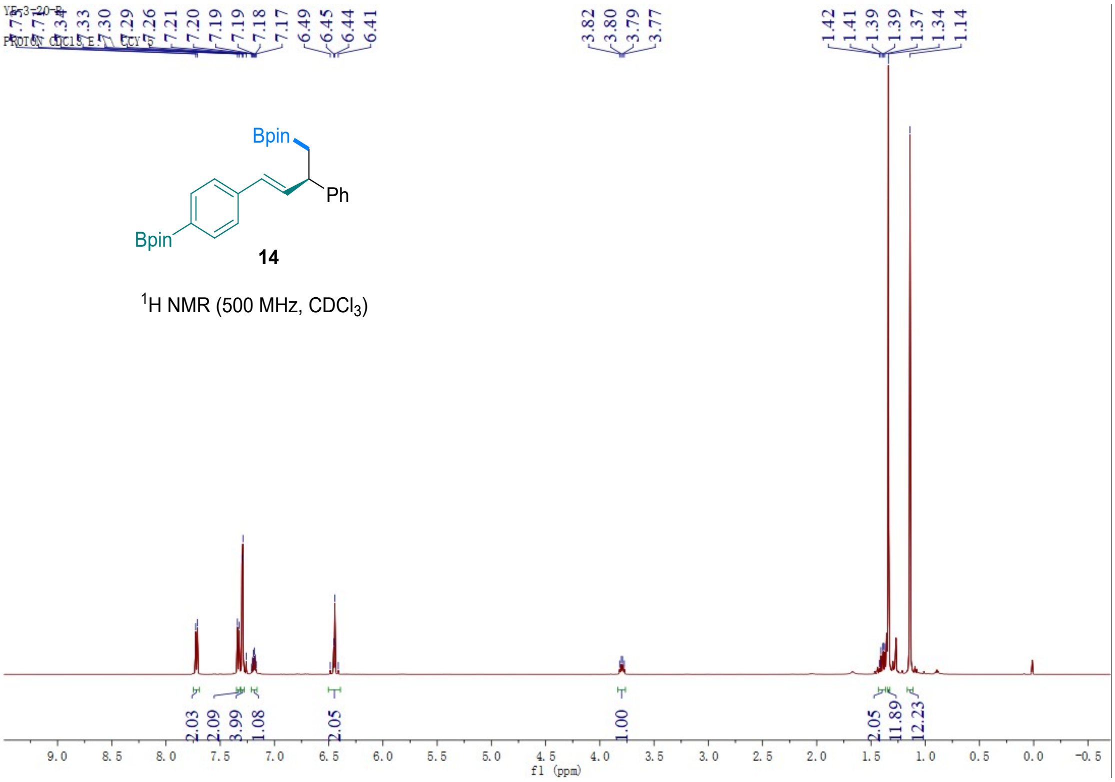

14

^{1}H NMR (500 MHz, $CDCl_3$)

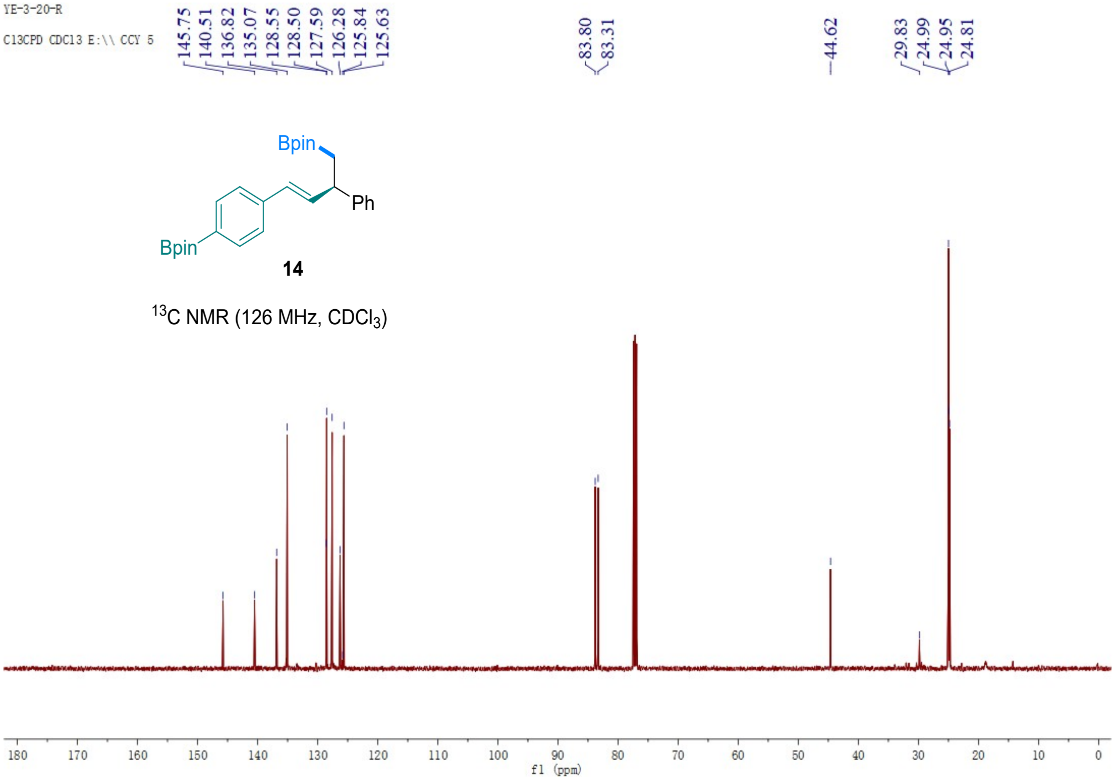

14

^{13}C NMR (126 MHz, $CDCl_3$)

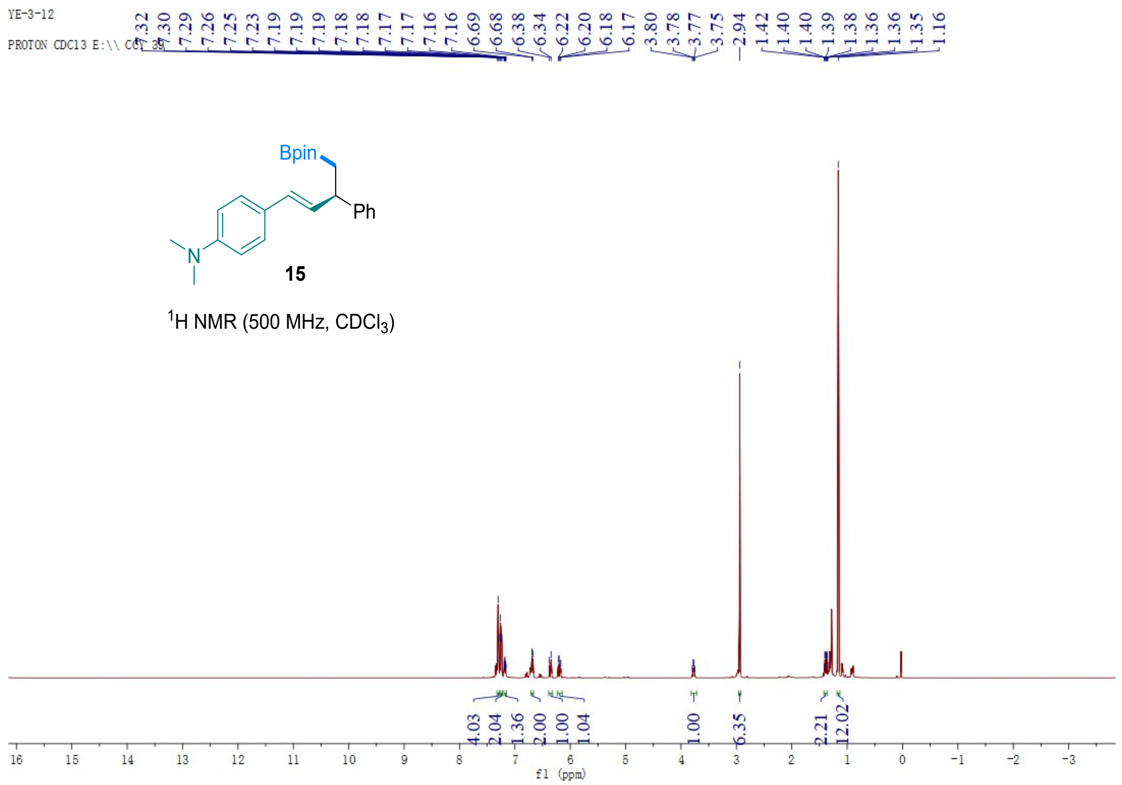
YE-3-12
PROTON CDCl3 E:\\ CCY 39
7.32 7.30 7.29 7.26 7.25 7.23 7.19 7.19 7.19 7.18 7.18 7.17 7.17 7.16 7.16 6.69 6.68 6.38 6.34 6.22 6.20 6.18 6.17 3.80 3.78 3.77 3.75 2.94 1.42 1.40 1.40 1.39 1.38 1.36 1.35 1.16
Bpin
Ph
N
15
^{1}H NMR (500 MHz, CDCl$_3$)
4.03 2.04 1.36 2.00 1.00 1.04 1.00 6.35 2.21 12.02
16 15 14 13 12 11 10 9 8 7 6 5 4 3 2 1 0 -1 -2 -3
f1 (ppm)

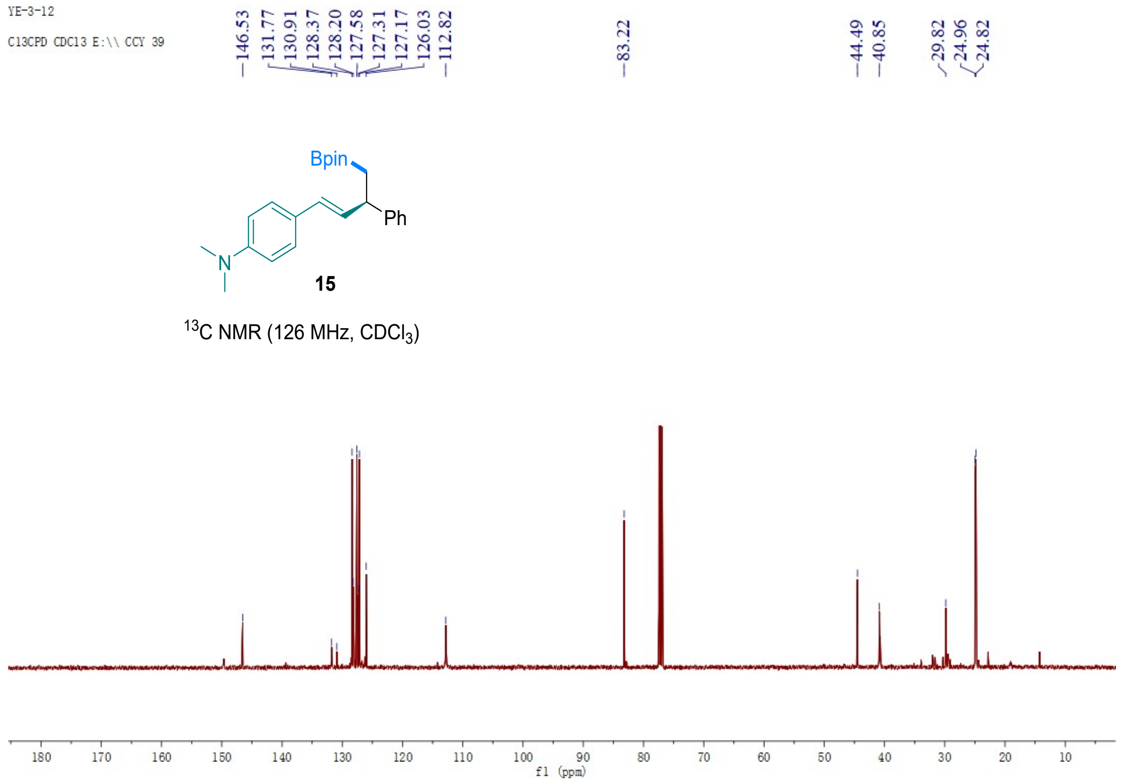
YE-3-12
C13CPD CDCl3 E:\\ CCY 39
146.53 131.77 130.91 128.37 128.20 127.58 127.31 127.17 126.03 112.82 83.22 44.49 40.85 29.82 24.96 24.82
Bpin
Ph
N
15
^{13}C NMR (126 MHz, CDCl$_3$)
180 170 160 150 140 130 120 110 100 90 80 70 60 50 40 30 20 10
f1 (ppm)

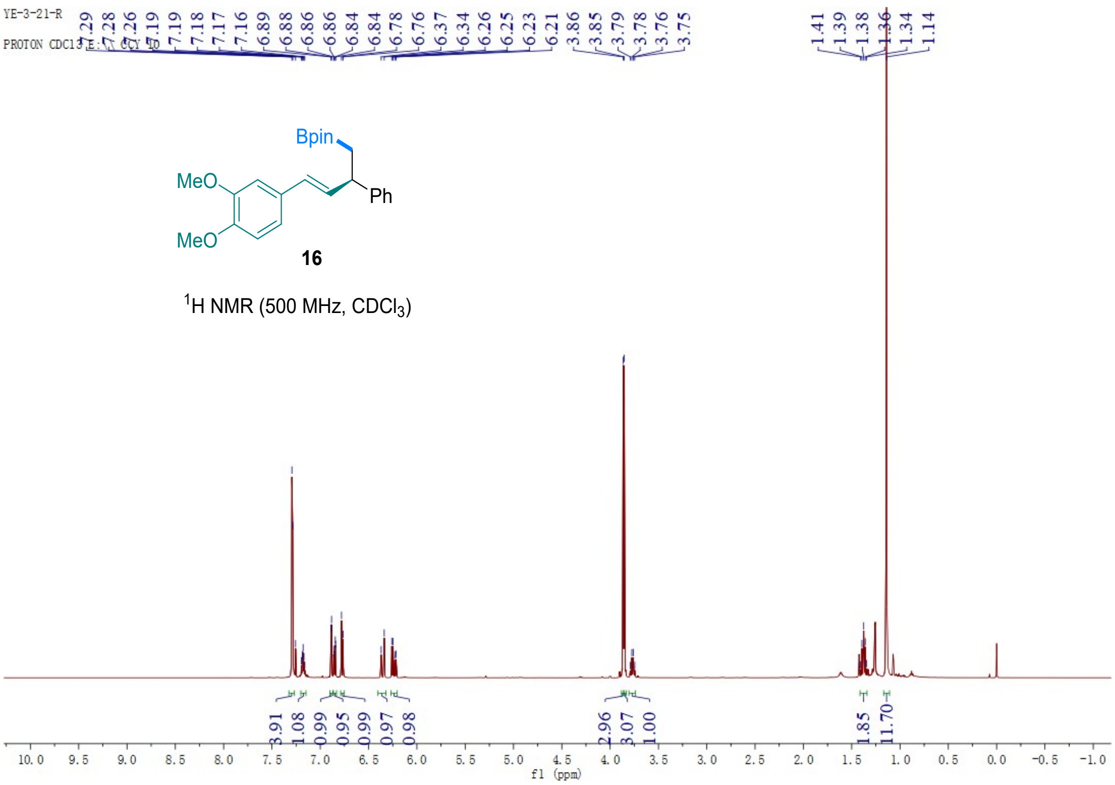
YE-3-21-R
PROTON CDCl3 E:\\ CCY 10
7.29 7.28 7.26 7.19 7.19 7.18 7.17 7.16 6.89 6.88 6.86 6.86 6.84 6.84 6.78 6.76 6.37 6.34 6.26 6.25 6.23 6.21 3.86 3.85 3.79 3.78 3.76 3.75
1.41 1.39 1.38 1.36 1.34 1.14
Bpin
MeO
MeO
Ph
16
1H NMR (500 MHz, CDCl3)
3.91 1.08 0.99 0.95 0.99 0.97 0.98 2.96 3.07 1.00 1.85 11.70
f1 (ppm)

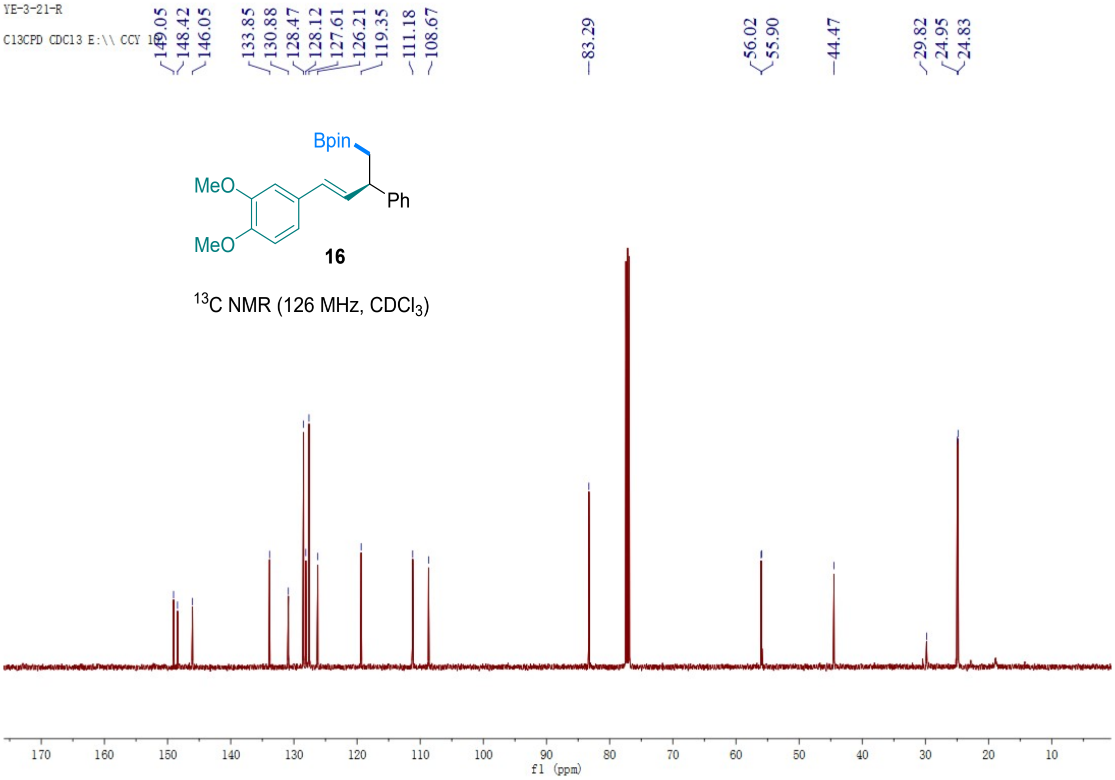
YE-3-21-R
C13CPD CDCl3 E:\\ CCY 10
149.05 148.42 146.05 133.85 130.88 128.47 128.12 127.61 126.21 119.35 111.18 108.67
83.29
56.02 55.90
44.47
29.82 24.95 24.83
Bpin
MeO
MeO
Ph
16
13C NMR (126 MHz, CDCl3)
f1 (ppm)

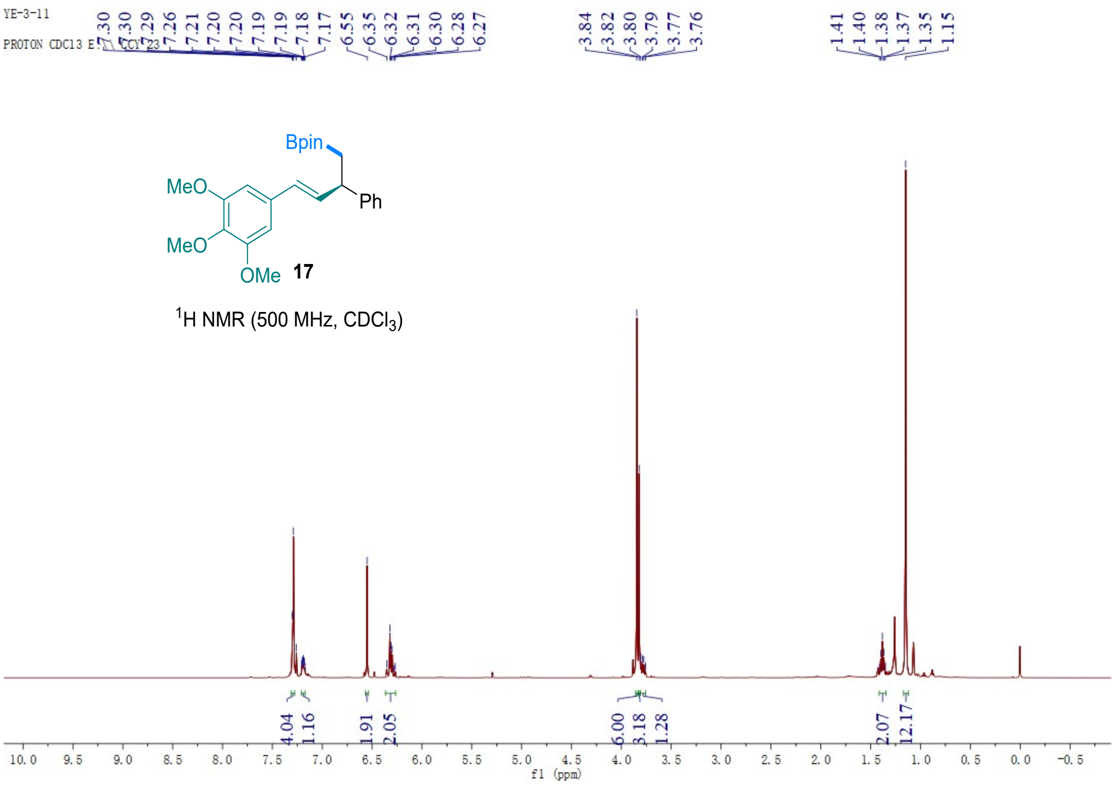
YE-3-11
7.30 7.30 7.29 7.26 7.21 7.20 7.20 7.19 7.19 7.18 7.17 6.55 6.35 6.32 6.31 6.30 6.28 6.27
3.84 3.82 3.80 3.79 3.77 3.76
1.41 1.40 1.38 1.37 1.35 1.15
Bpin
MeO
MeO
OMe
Ph
17
^{1}H NMR (500 MHz, $CDCl_3$)
4.04 1.16 1.91 2.05 6.00 3.18 1.28 2.07 12.17
f1 (ppm)

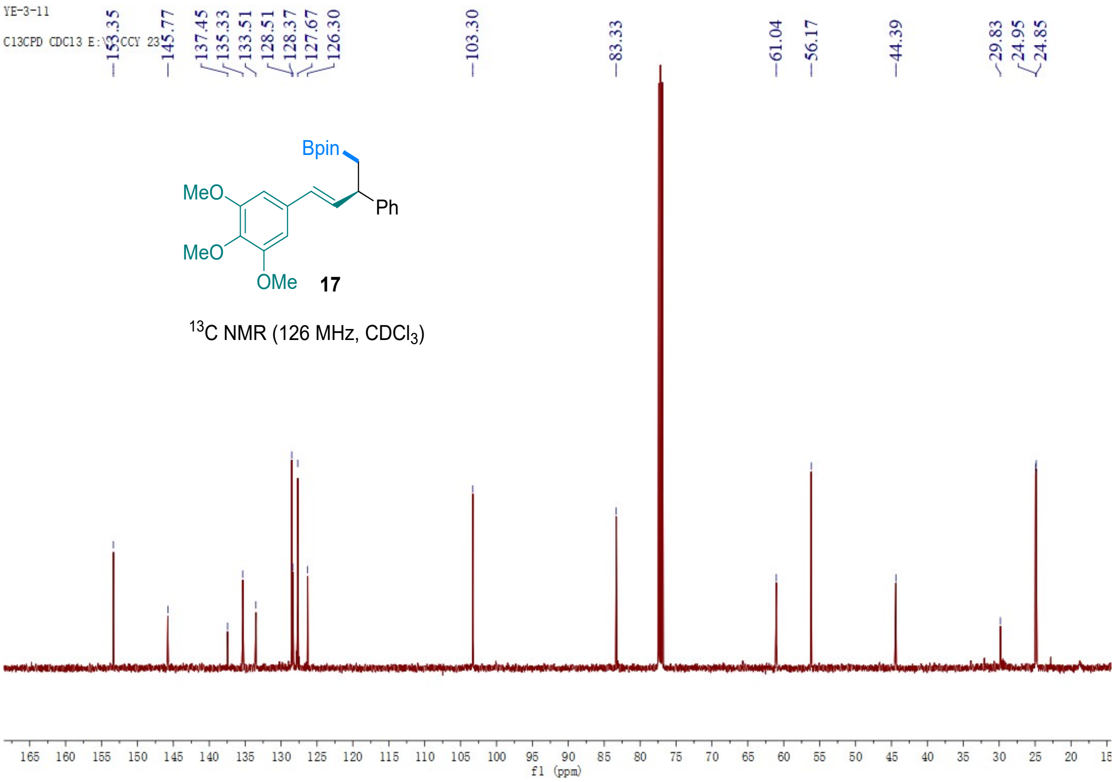
YE-3-11
153.35 145.77 137.45 135.33 133.51 128.51 128.37 127.67 126.30 103.30 83.33 61.04 56.17 44.39 29.83 24.95 24.85
Bpin
MeO
MeO
OMe
Ph
17
^{13}C NMR (126 MHz, $CDCl_3$)
f1 (ppm)

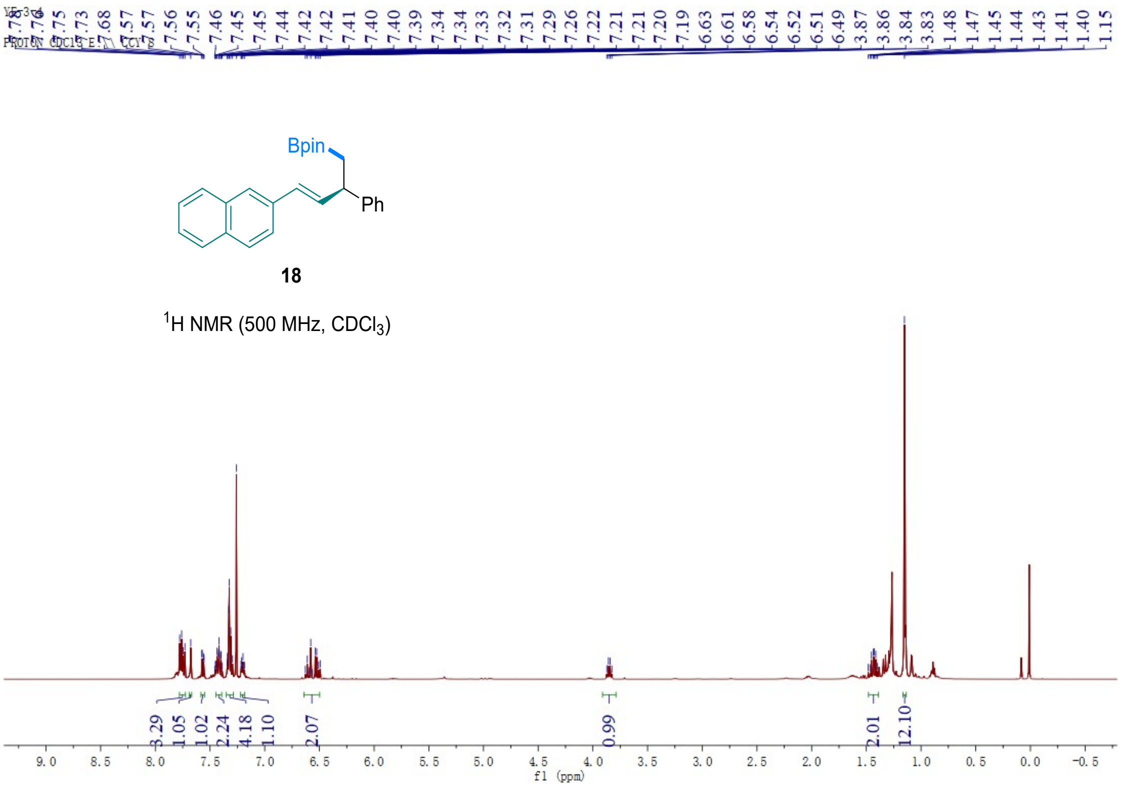

18

^{1}H NMR (500 MHz, $CDCl_3$)

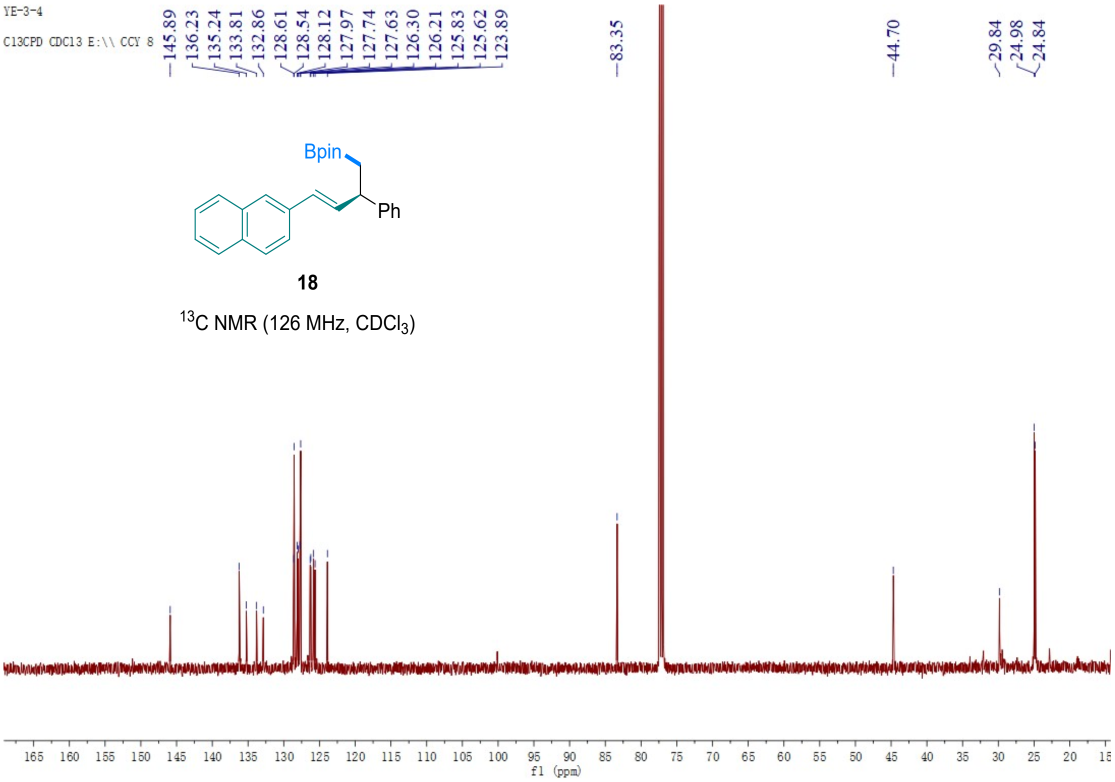

18

^{13}C NMR (126 MHz, $CDCl_3$)

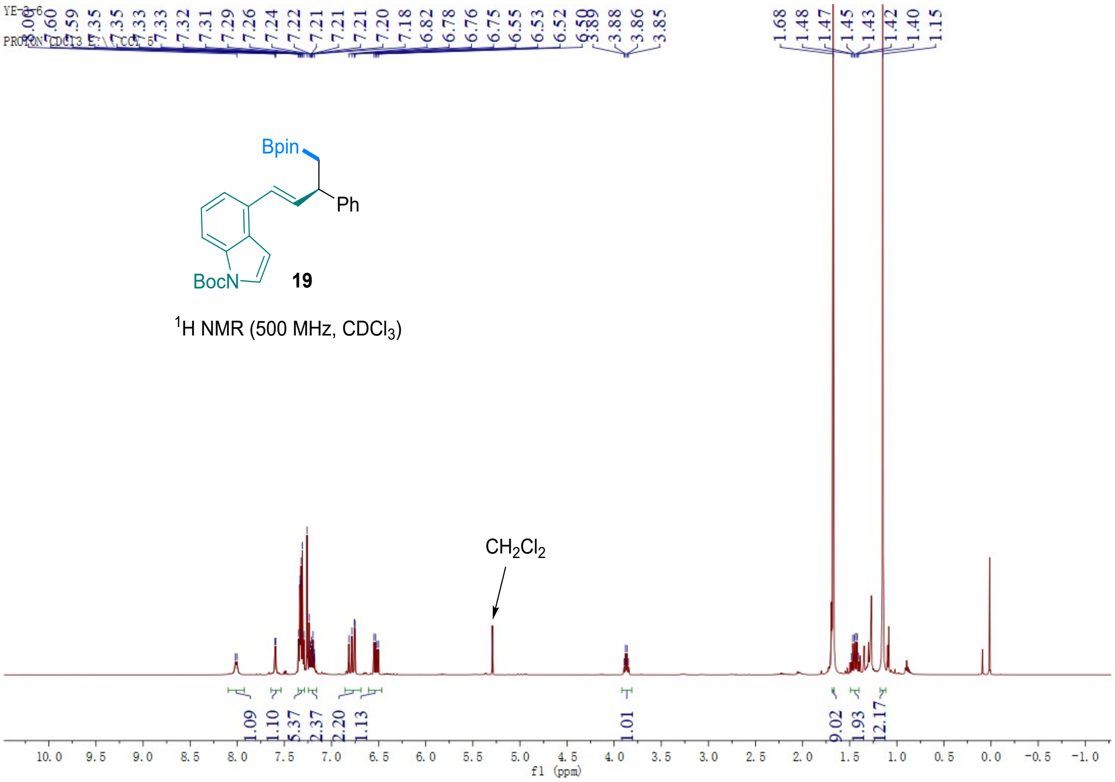
Bpin
Ph
BocN
19
1H NMR (500 MHz, CDCl3)
CH2Cl2
1.68
1.48
1.47
1.45
1.43
1.42
1.40
1.15
3.88
3.86
3.85
1.09
1.10
5.37
2.37
2.20
1.13
1.01
9.02
1.93
12.17
f1 (ppm)

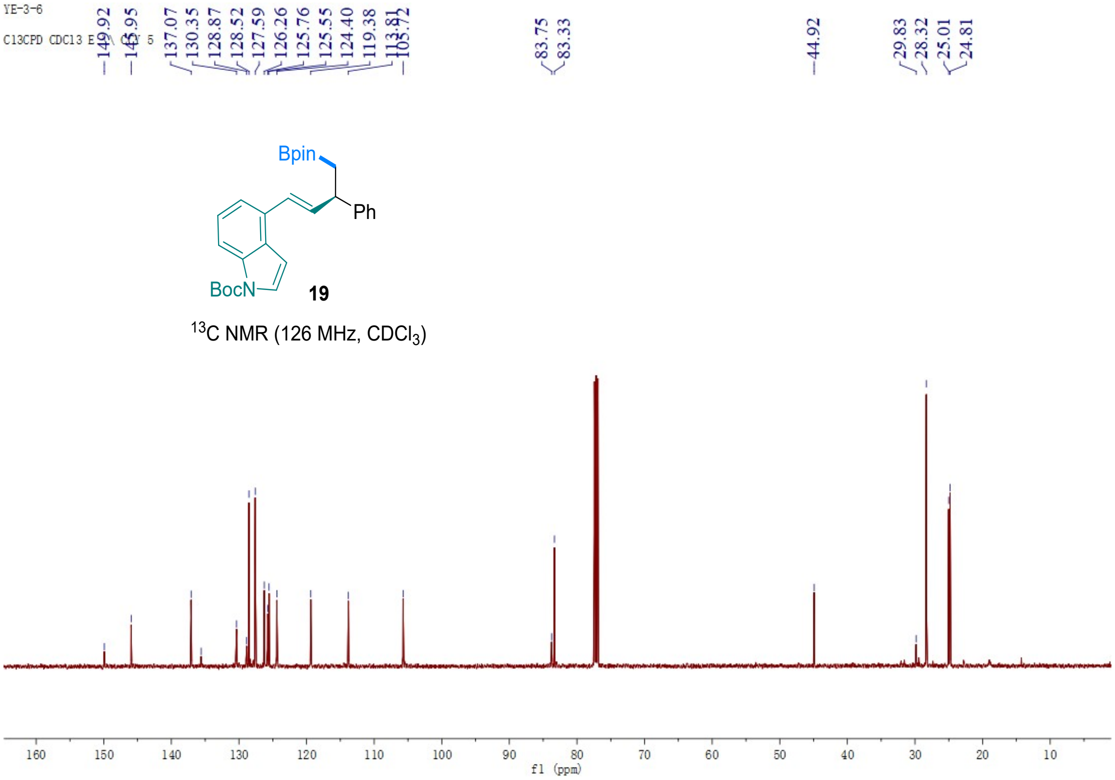
YE-3-6
Bpin
Ph
BocN
19
13C NMR (126 MHz, CDCl3)
149.92
137.07
130.35
128.87
128.52
127.59
126.26
125.76
125.55
124.40
119.38
105.72
83.75
83.33
44.92
29.83
28.32
25.01
24.81
f1 (ppm)

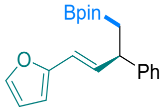

20

^{1}H NMR (500 MHz, $CDCl_3$)

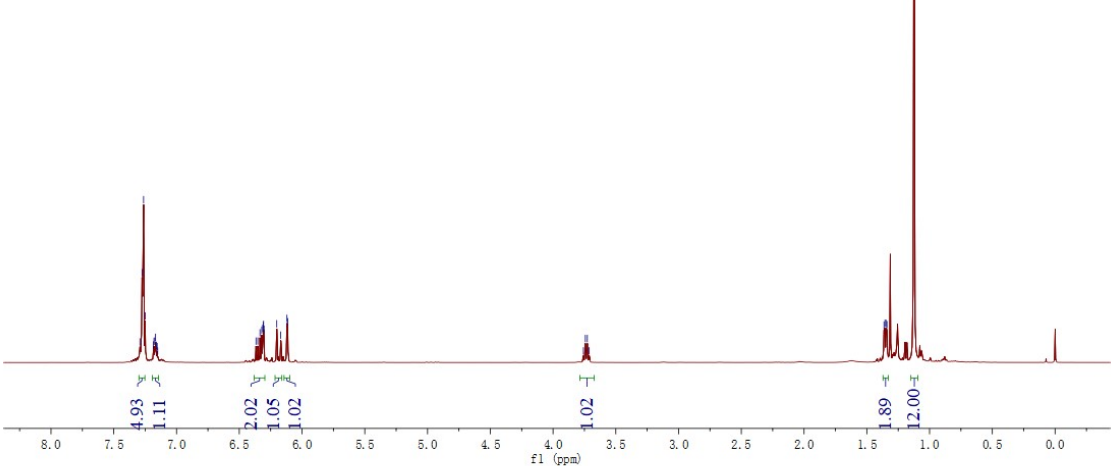

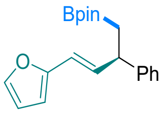

20

^{13}C NMR (126 MHz, $CDCl_3$)

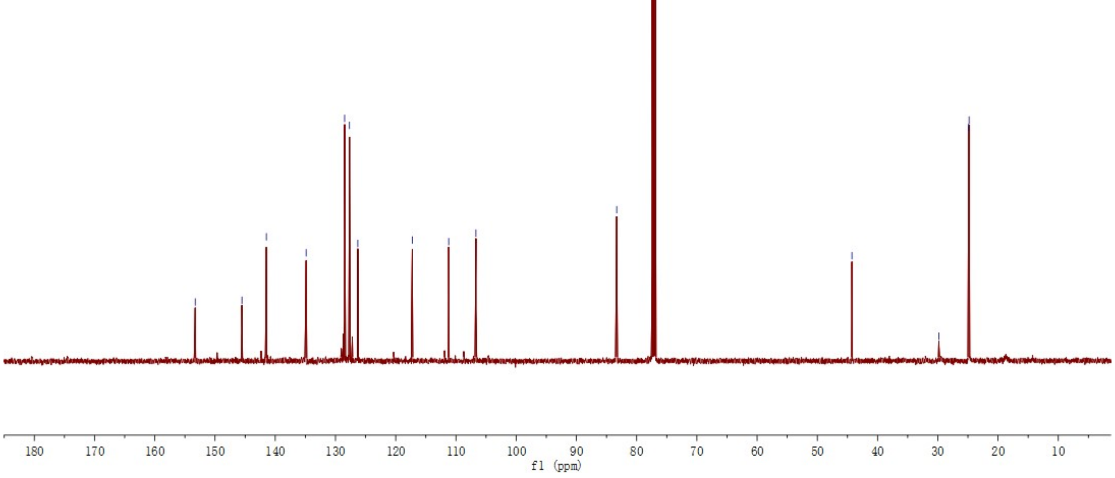

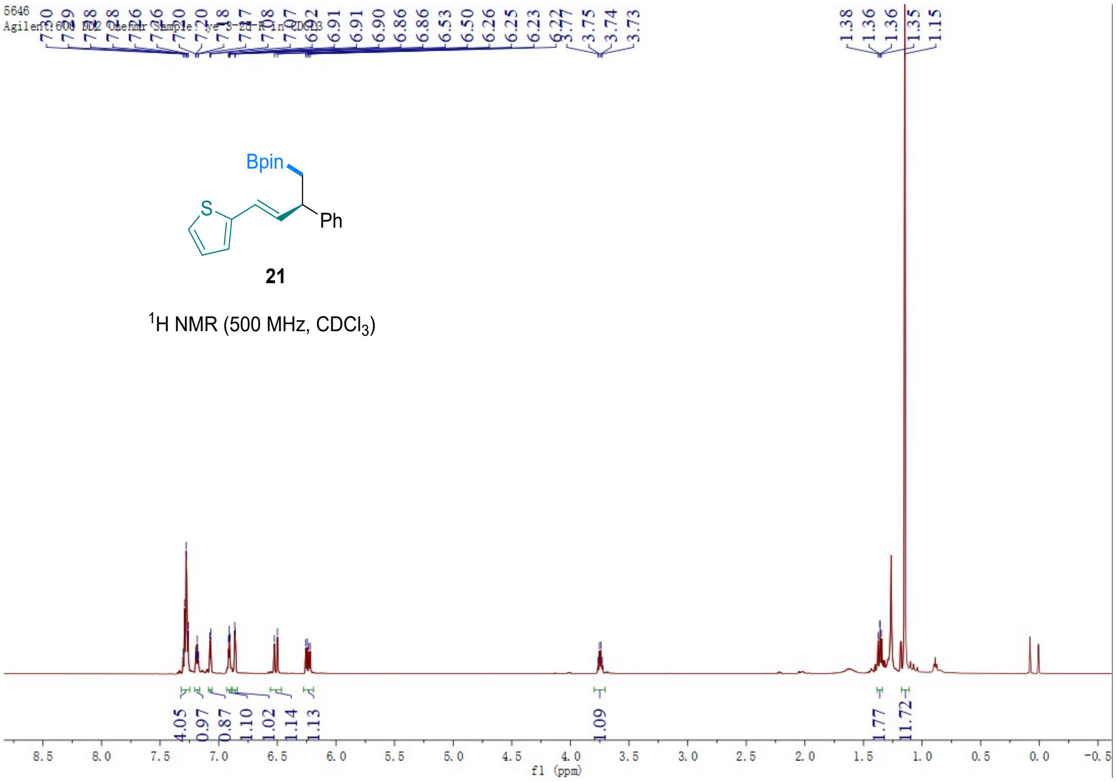
5646
7.30
7.29
7.28
7.28
7.26
7.26
7.20
7.20
7.18
7.17
7.18
7.17
6.92
6.91
6.91
6.90
6.86
6.86
6.53
6.50
6.26
6.25
6.23
6.22
3.77
3.75
3.74
3.73
1.38
1.36
1.36
1.35
1.15
Bpin
S
Ph
21
1H NMR (500 MHz, CDCl3)
4.05
0.97
0.87
1.10
1.02
1.14
1.13
1.09
1.77
11.72
f1 (ppm)

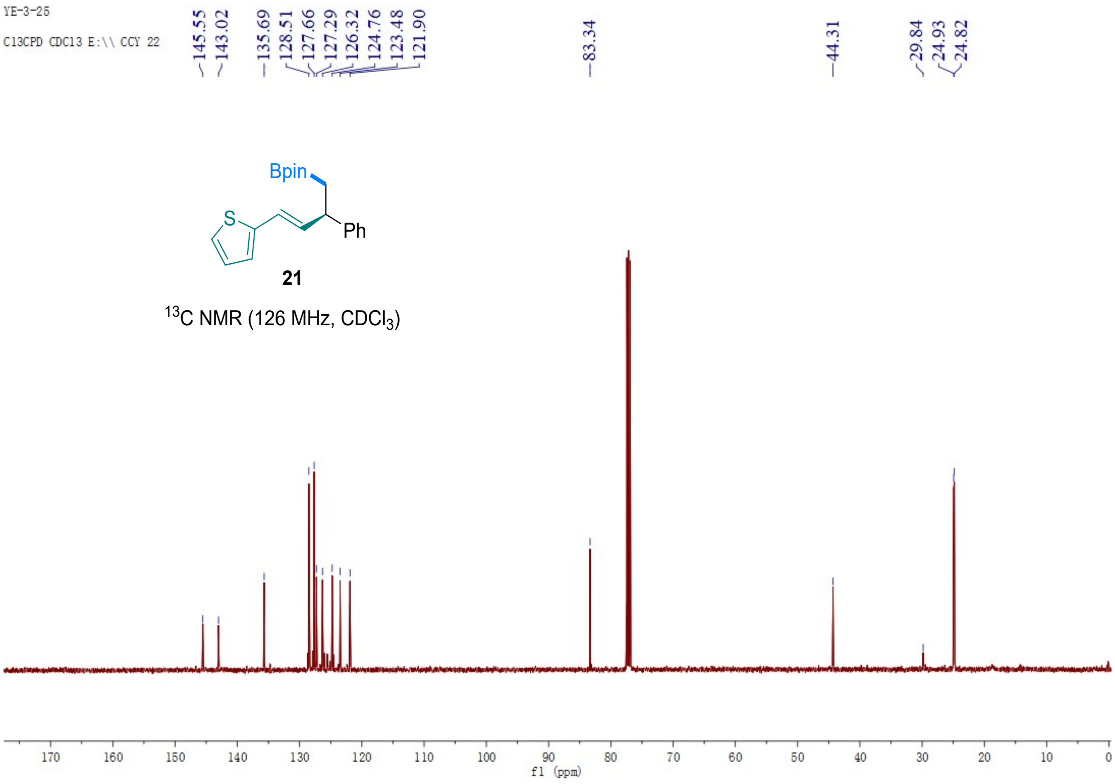
YE-3-25
C13CPD CDCl3 E:\\ CCY 22
145.55
143.02
135.69
128.51
127.66
127.29
126.32
124.76
123.48
121.90
83.34
44.31
29.84
24.93
24.82
Bpin
S
Ph
21
13C NMR (126 MHz, CDCl3)
f1 (ppm)

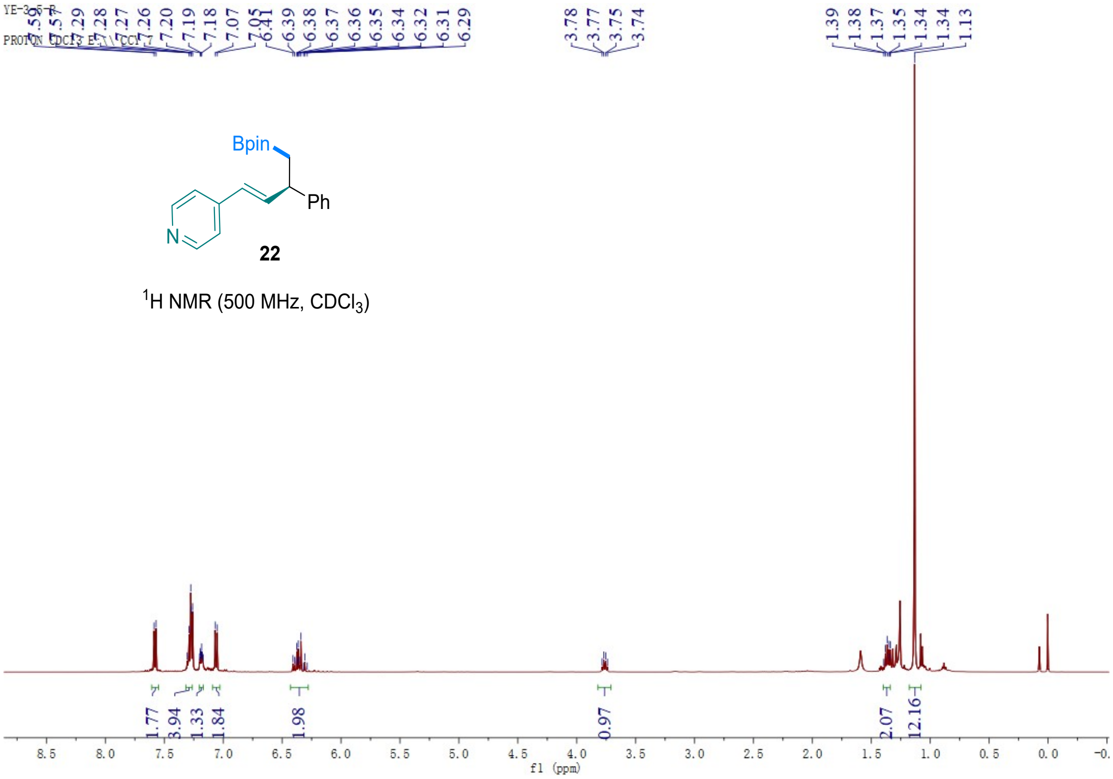

^{1}H NMR (500 MHz, $CDCl_3$)

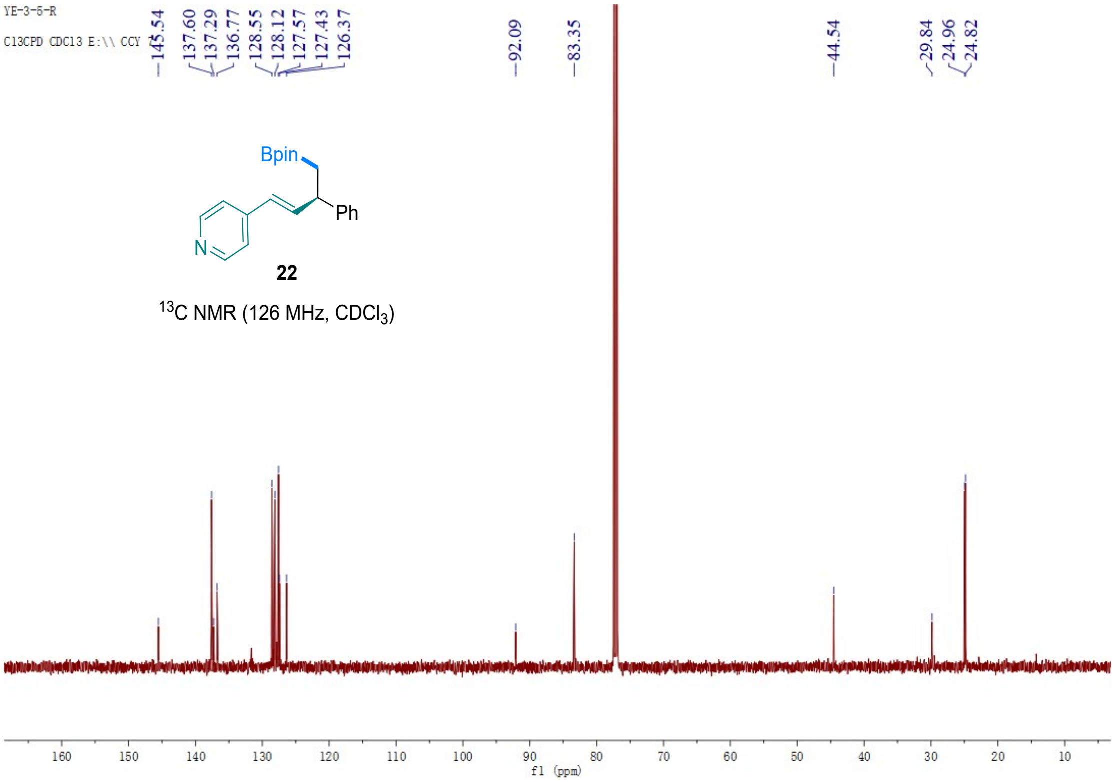

^{13}C NMR (126 MHz, $CDCl_3$)

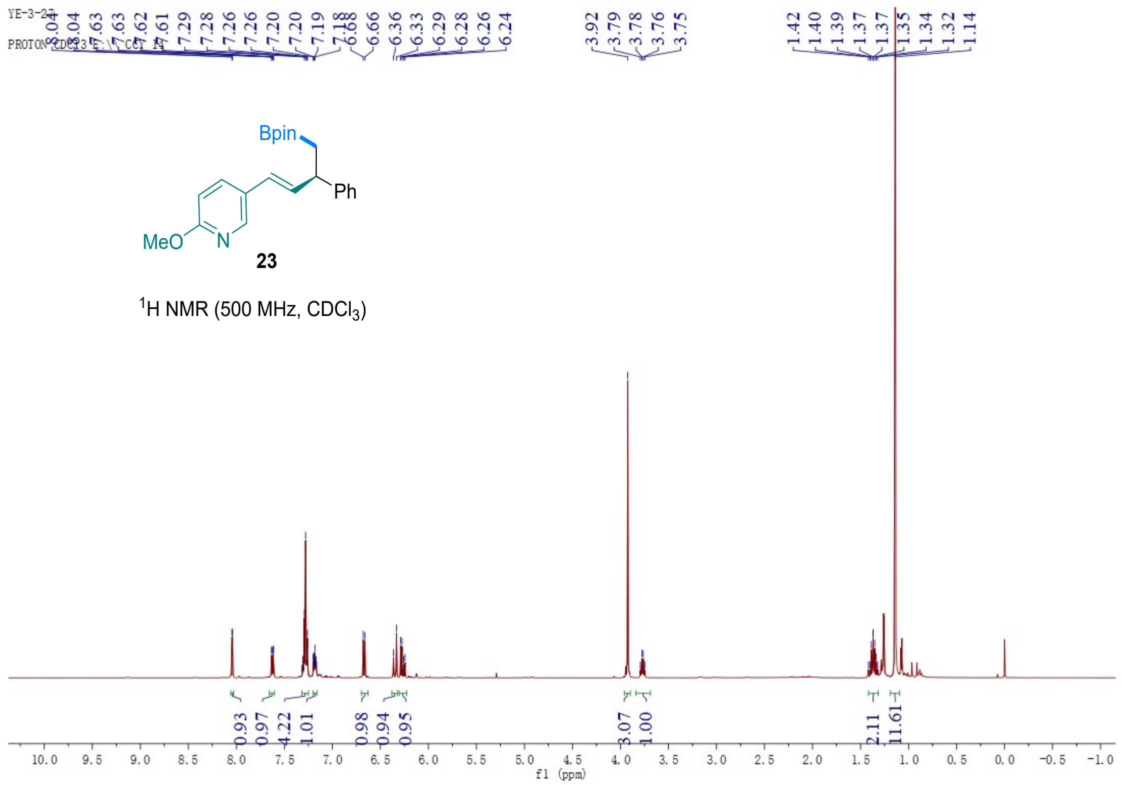
YE-3-27
PROTON CDCl3 E:\\ CCY 14
Bpin
Ph
MeO
N
23
1H NMR (500 MHz, CDCl3)
f1 (ppm)

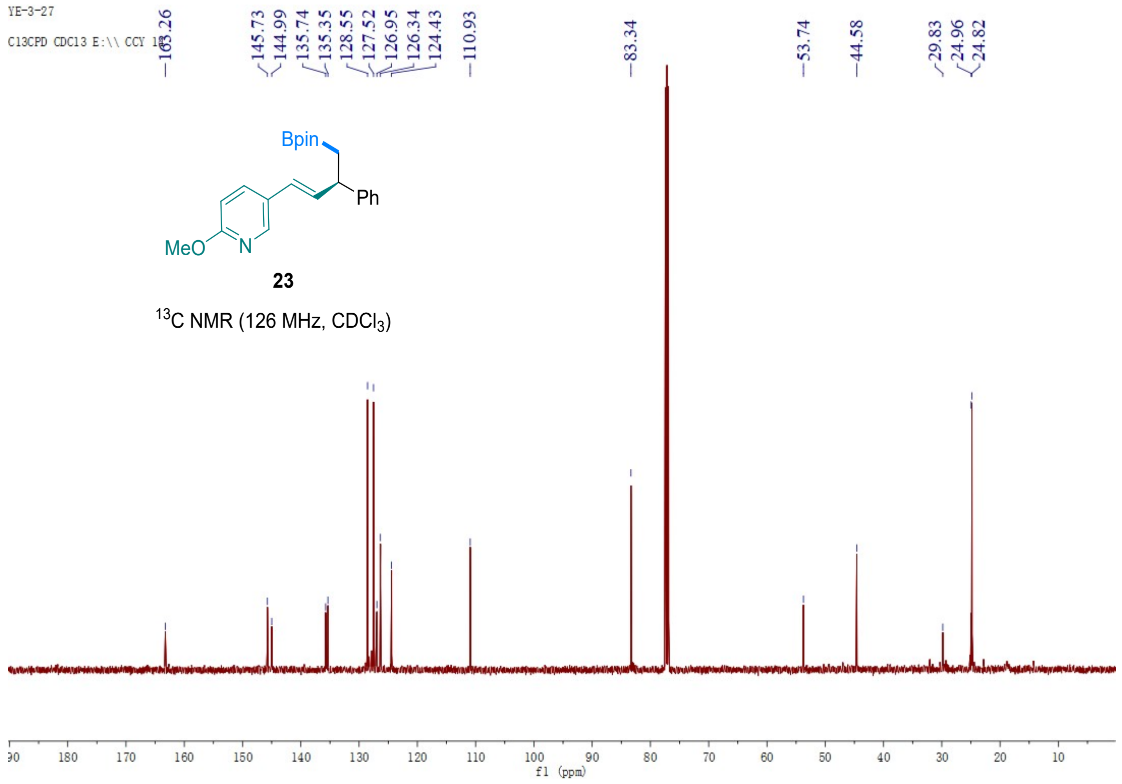
YE-3-27
C13CPD CDCl3 E:\\ CCY 14
Bpin
Ph
MeO
N
23
13C NMR (126 MHz, CDCl3)
f1 (ppm)

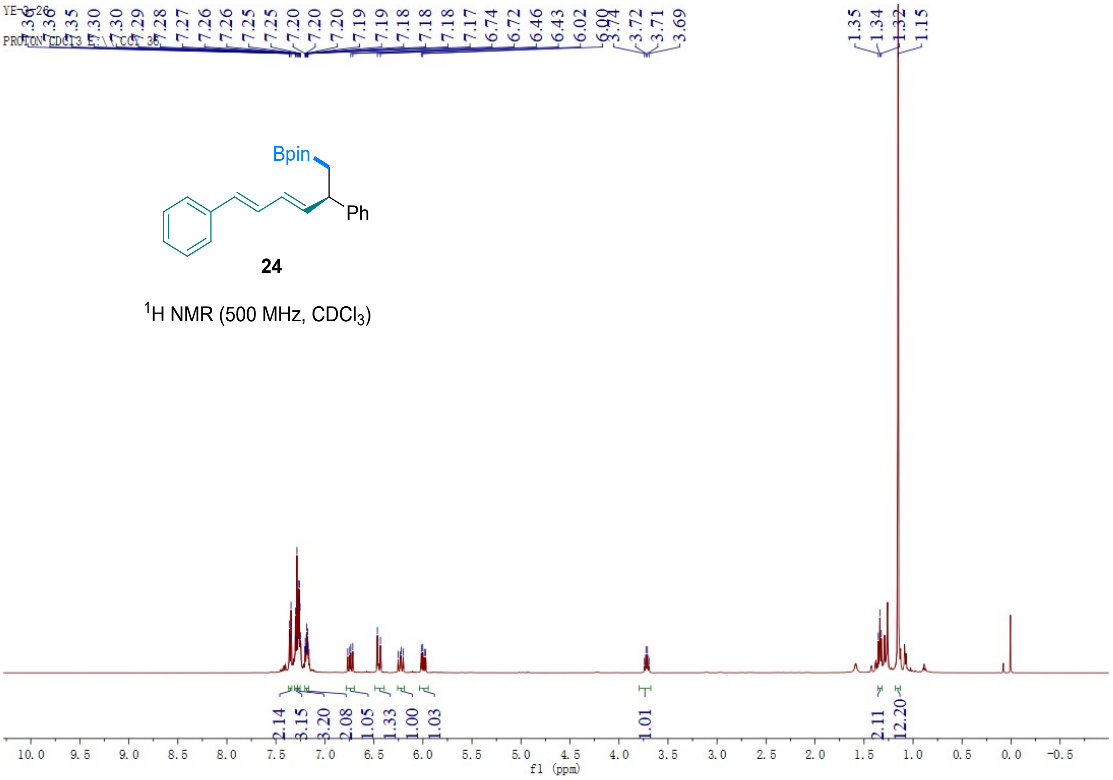

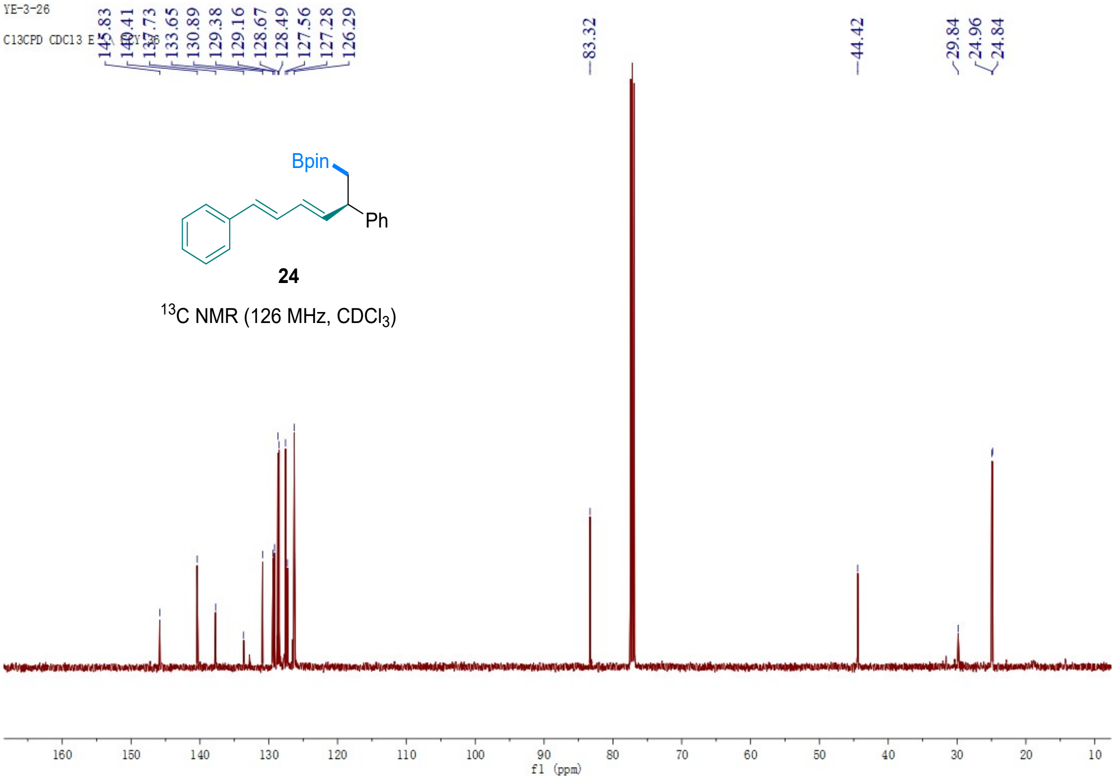

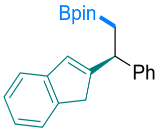

25

^{1}H NMR (500 MHz, $CDCl_3$)

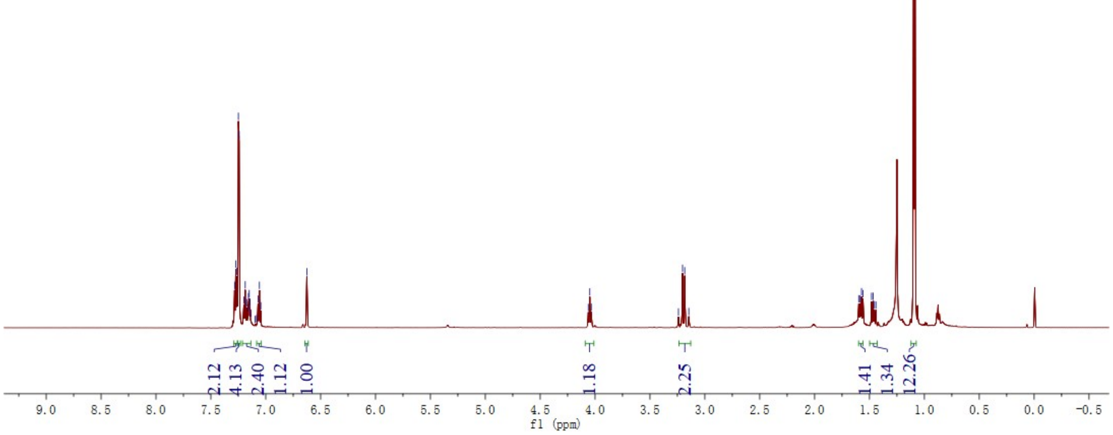

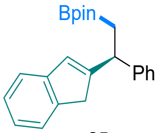

25

^{13}C NMR (126 MHz, $CDCl_3$)

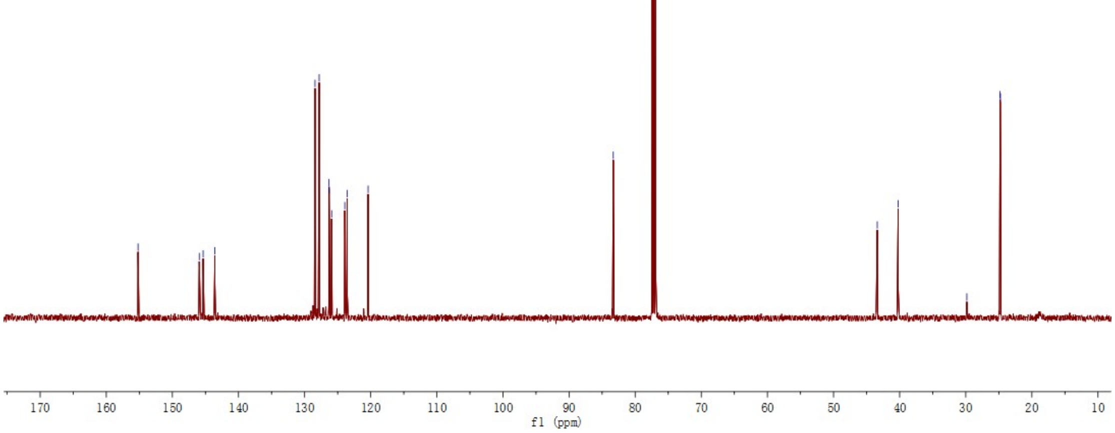

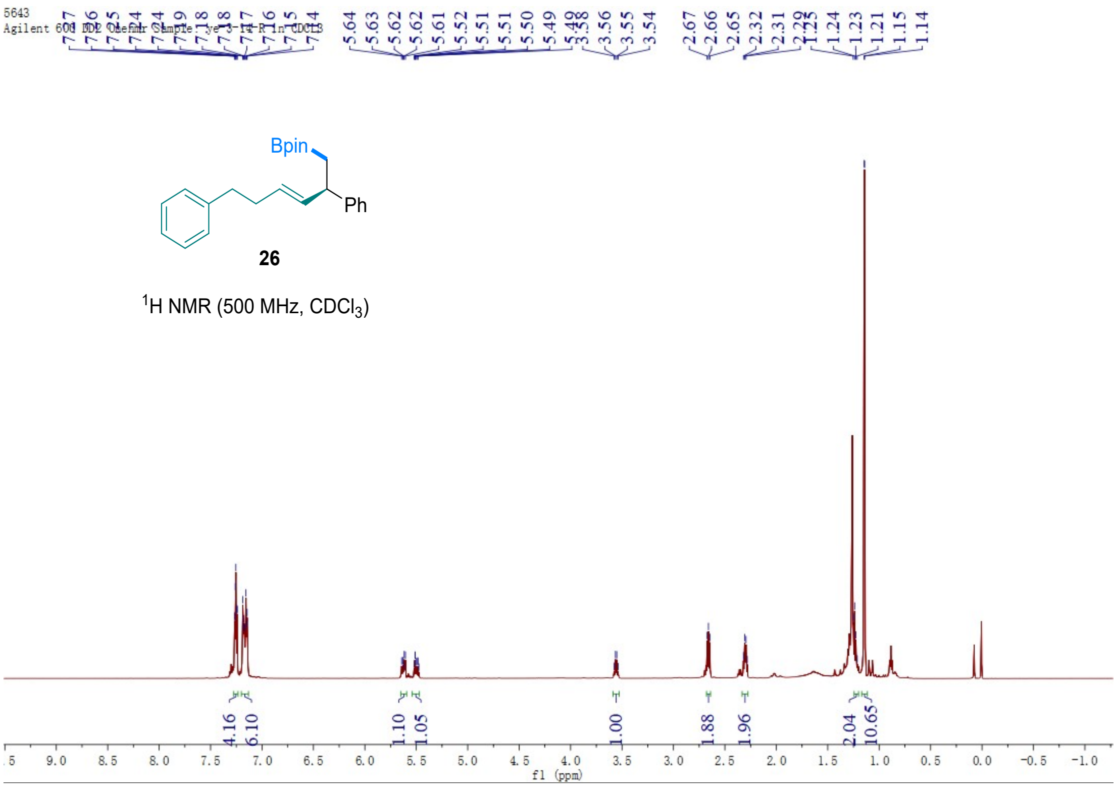

26

^{1}H NMR (500 MHz, $CDCl_3$)

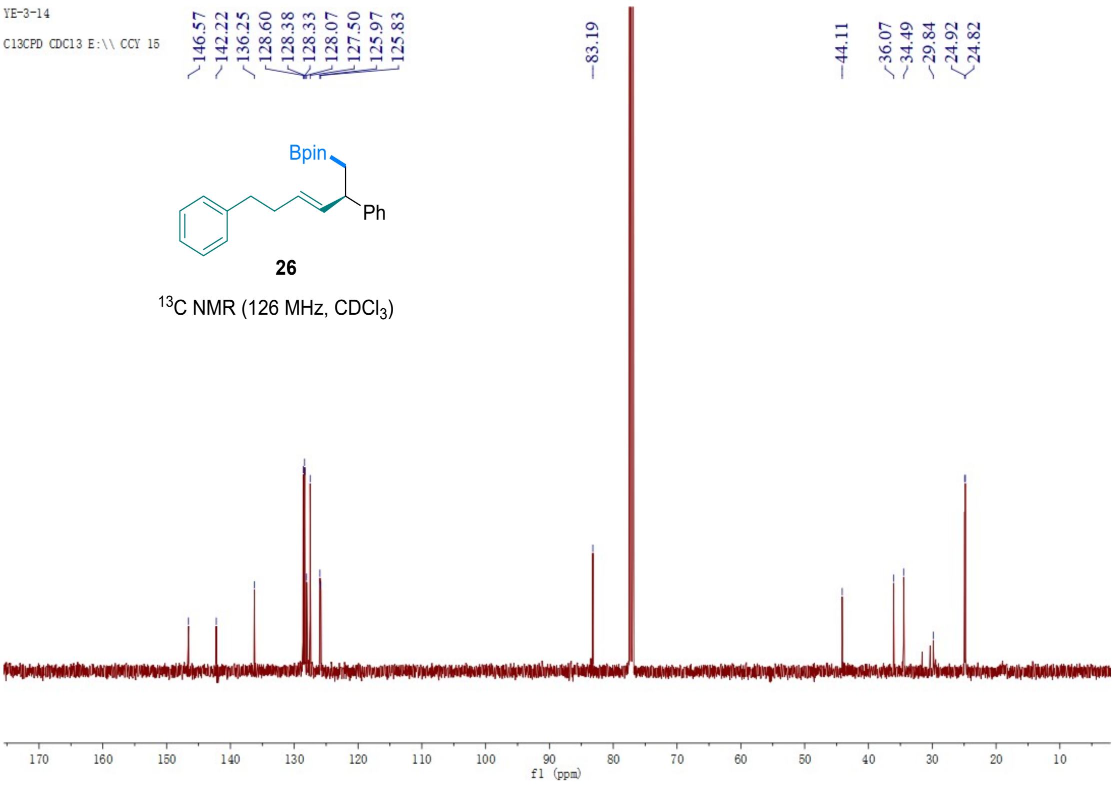

26

^{13}C NMR (126 MHz, $CDCl_3$)

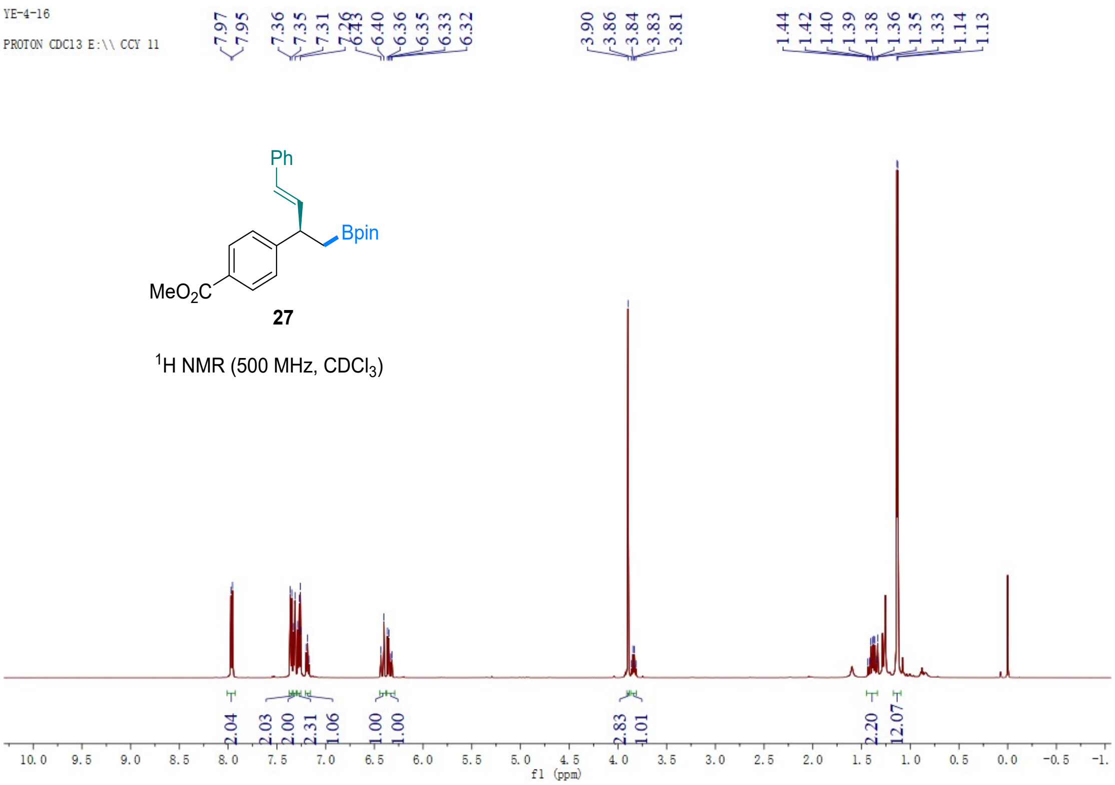
YE-4-16
PROTON CDCl3 E:\\ CCY 11
7.97
7.95
7.36
7.35
7.31
7.26
6.43
6.40
6.36
6.35
6.33
6.32
3.90
3.86
3.84
3.83
3.81
1.44
1.42
1.40
1.39
1.38
1.36
1.35
1.33
1.14
1.13
Ph
Bpin
MeO2C
27
1H NMR (500 MHz, CDCl3)
2.04
2.03
2.00
2.31
1.06
1.00
1.00
2.83
1.01
2.20
12.07
f1 (ppm)

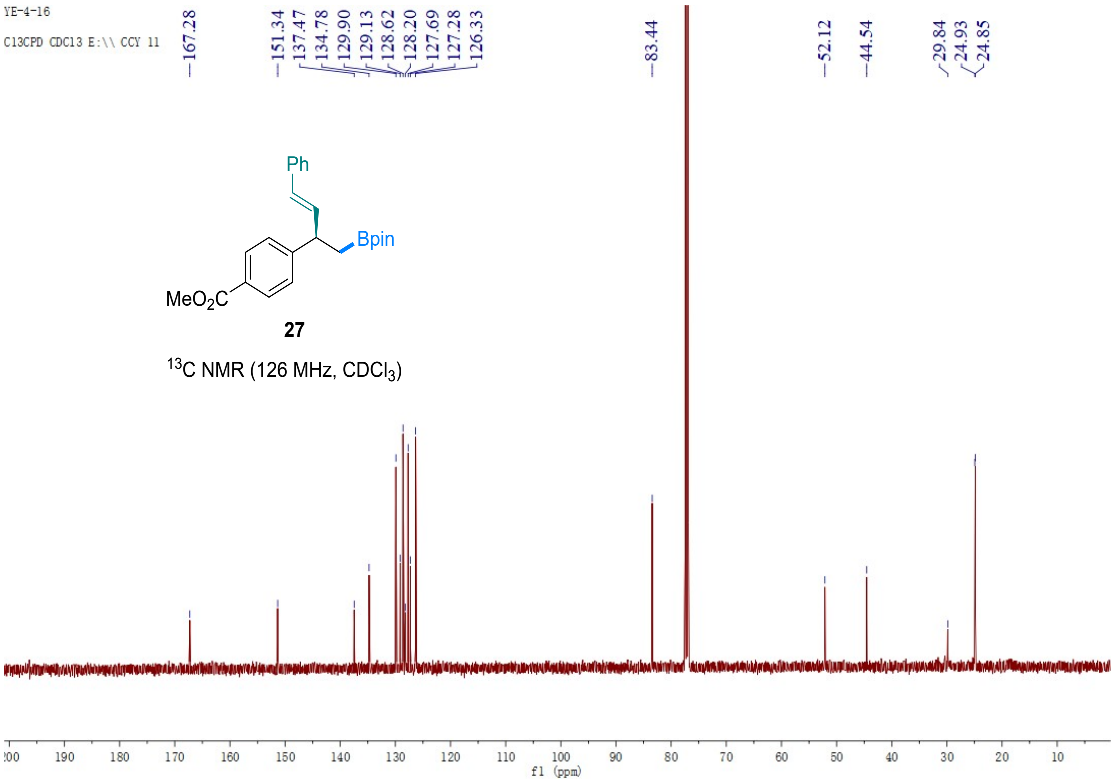
YE-4-16
C13CPD CDCl3 E:\\ CCY 11
167.28
151.34
137.47
134.78
129.90
129.13
128.62
128.20
127.69
127.28
126.33
83.44
52.12
44.54
29.84
24.93
24.85
Ph
Bpin
MeO2C
27
13C NMR (126 MHz, CDCl3)
f1 (ppm)

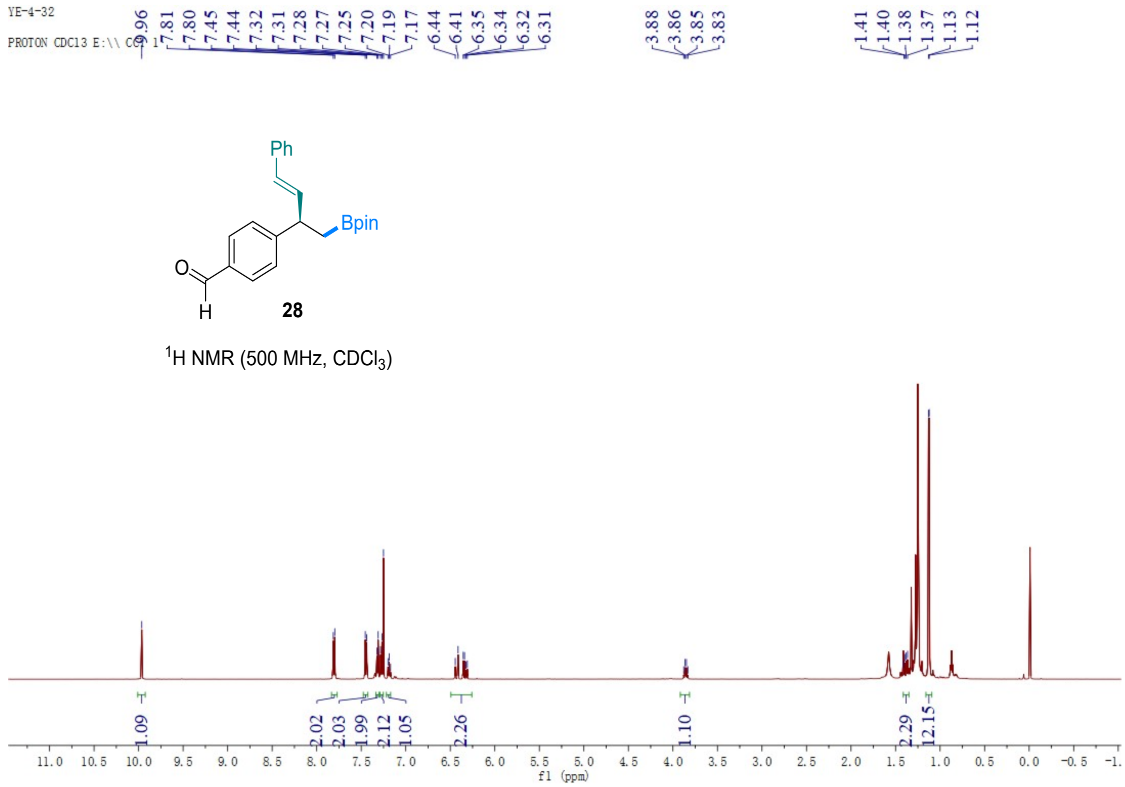

^{1}H NMR (500 MHz, $CDCl_3$)

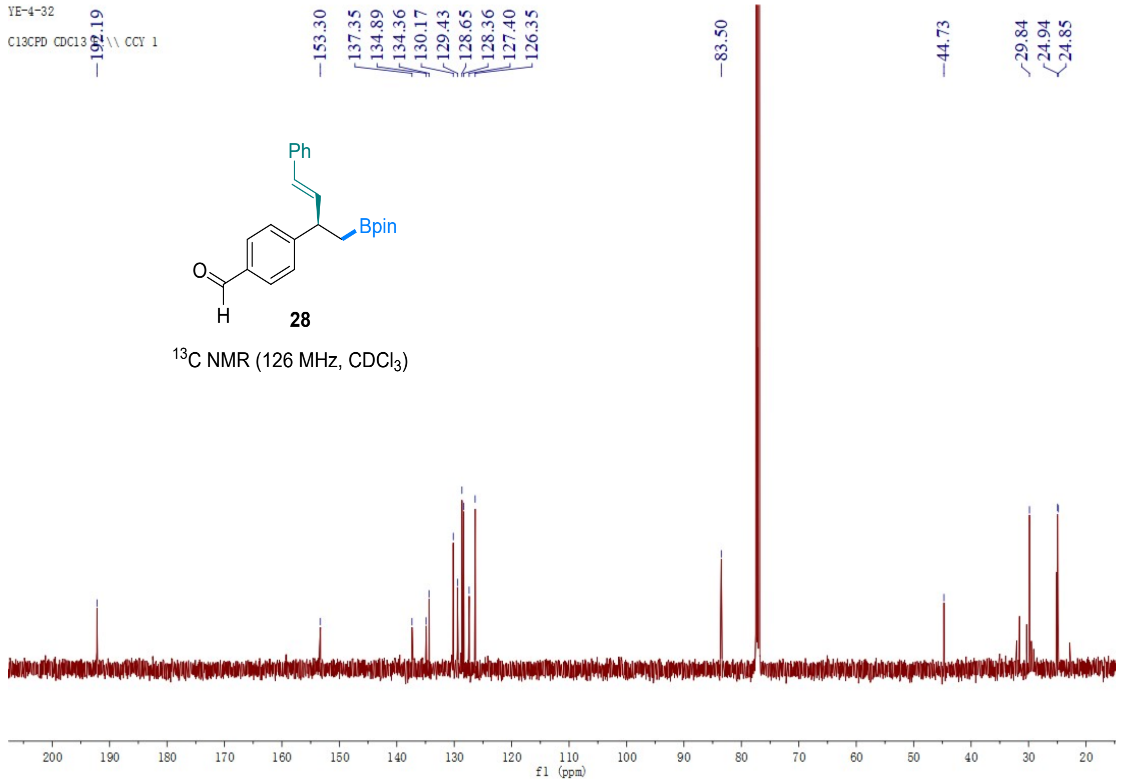

^{13}C NMR (126 MHz, $CDCl_3$)

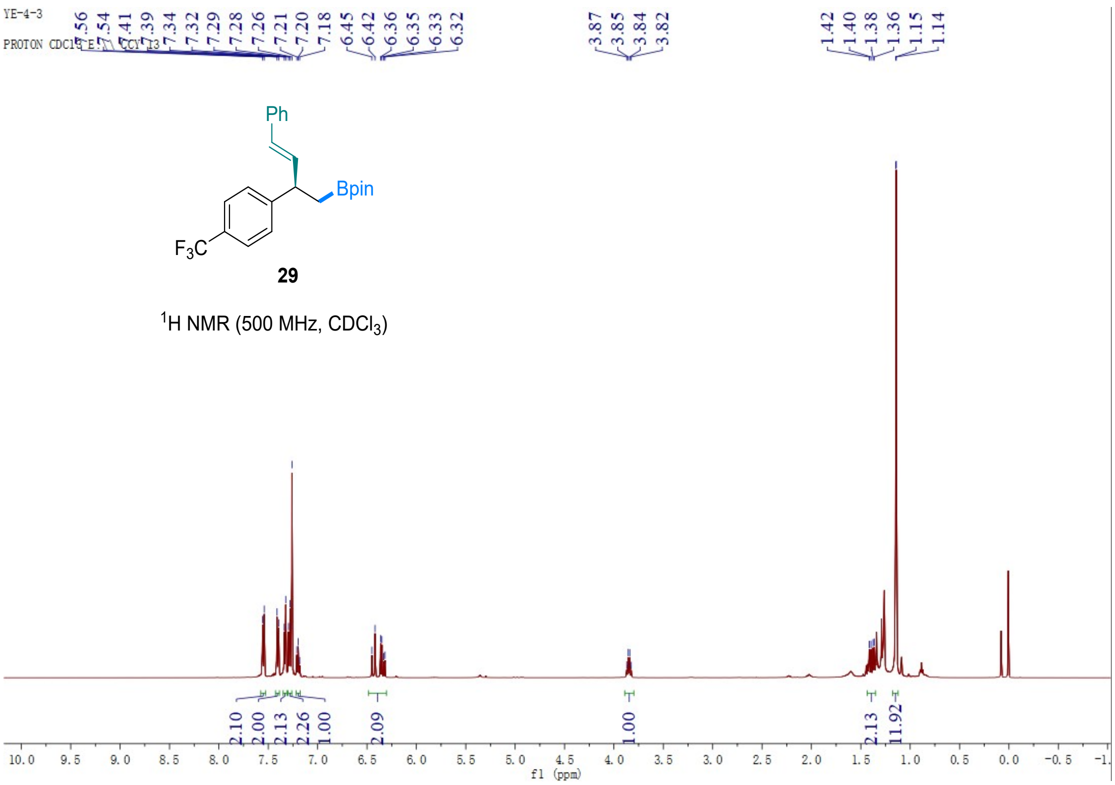
YE-4-3
PROTON CDCl3 E:\\ CCY 13
7.56 7.54 7.41 7.39 7.34 7.32 7.29 7.28 7.26 7.21 7.20 7.18 6.45 6.42 6.36 6.35 6.33 6.32
3.87 3.85 3.84 3.82
1.42 1.40 1.38 1.36 1.15 1.14
Ph
Bpin
F3C
29
1H NMR (500 MHz, CDCl3)
2.10 2.00 2.13 2.26 1.00 2.09 1.00 2.13 11.92
f1 (ppm)

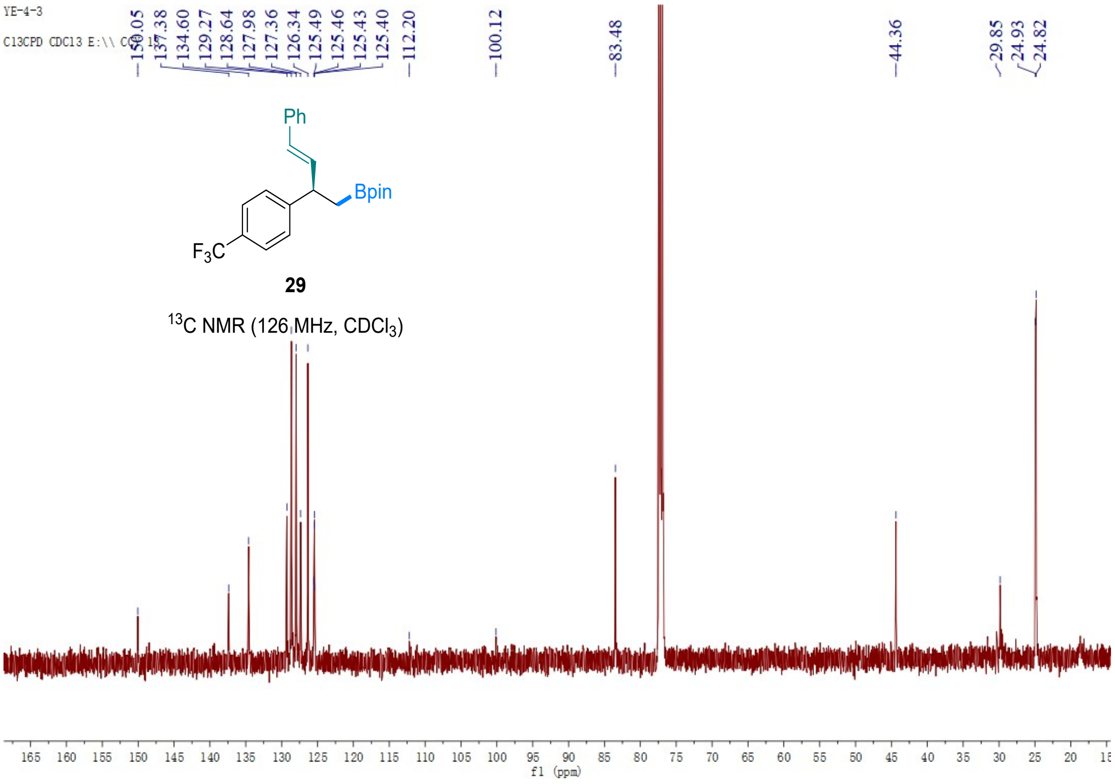
YE-4-3
C13CPD CDCl3 E:\\ CCY 13
150.05 137.38 134.60 129.27 128.64 127.98 127.36 126.34 125.49 125.46 125.43 125.40 112.20
100.12
83.48
44.36
29.85 24.93 24.82
Ph
Bpin
F3C
29
13C NMR (126 MHz, CDCl3)
f1 (ppm)

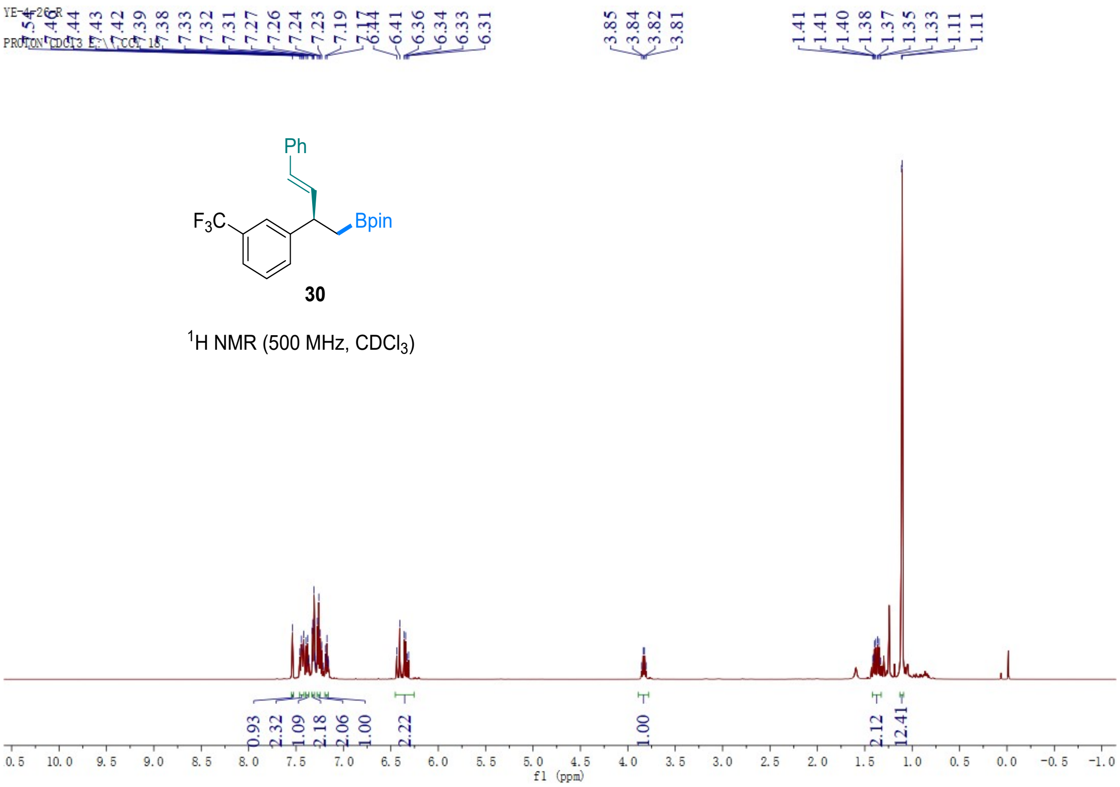
YE-4-26-R
PROTON CDCl3 E:\\ CCY 18
7.44
7.43
7.42
7.39
7.38
7.33
7.32
7.31
7.27
7.26
7.24
7.23
7.19
6.41
6.36
6.34
6.33
6.31
3.85
3.84
3.82
3.81
1.41
1.41
1.40
1.38
1.37
1.35
1.33
1.11
1.11
Ph
F_3C
Bpin
30
^{1}H NMR (500 MHz, CDCl$_3$)
0.93
2.32
1.09
2.18
2.06
1.00
2.22
1.00
2.12
12.41
f1 (ppm)

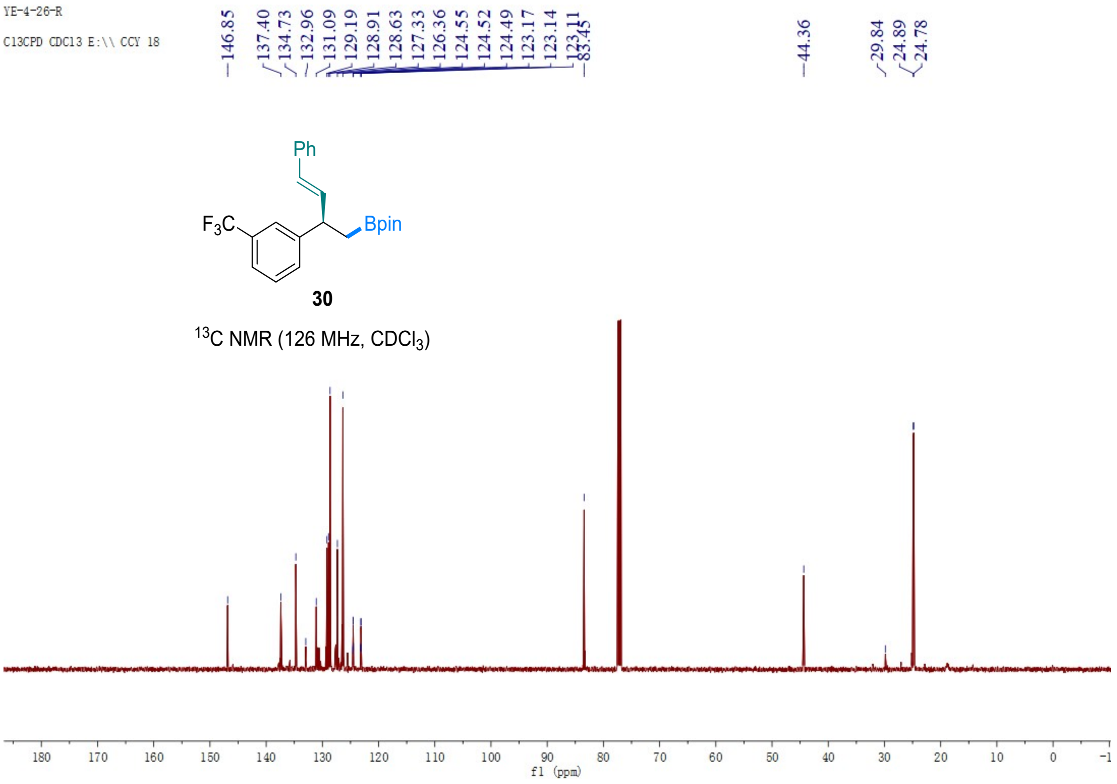
YE-4-26-R
C13CPD CDCl3 E:\\ CCY 18
146.85
137.40
134.73
132.96
131.09
129.19
128.91
128.63
127.33
126.36
124.55
124.52
124.49
123.17
123.14
123.11
83.45
44.36
29.84
24.89
24.78
Ph
F_3C
Bpin
30
^{13}C NMR (126 MHz, CDCl$_3$)
f1 (ppm)

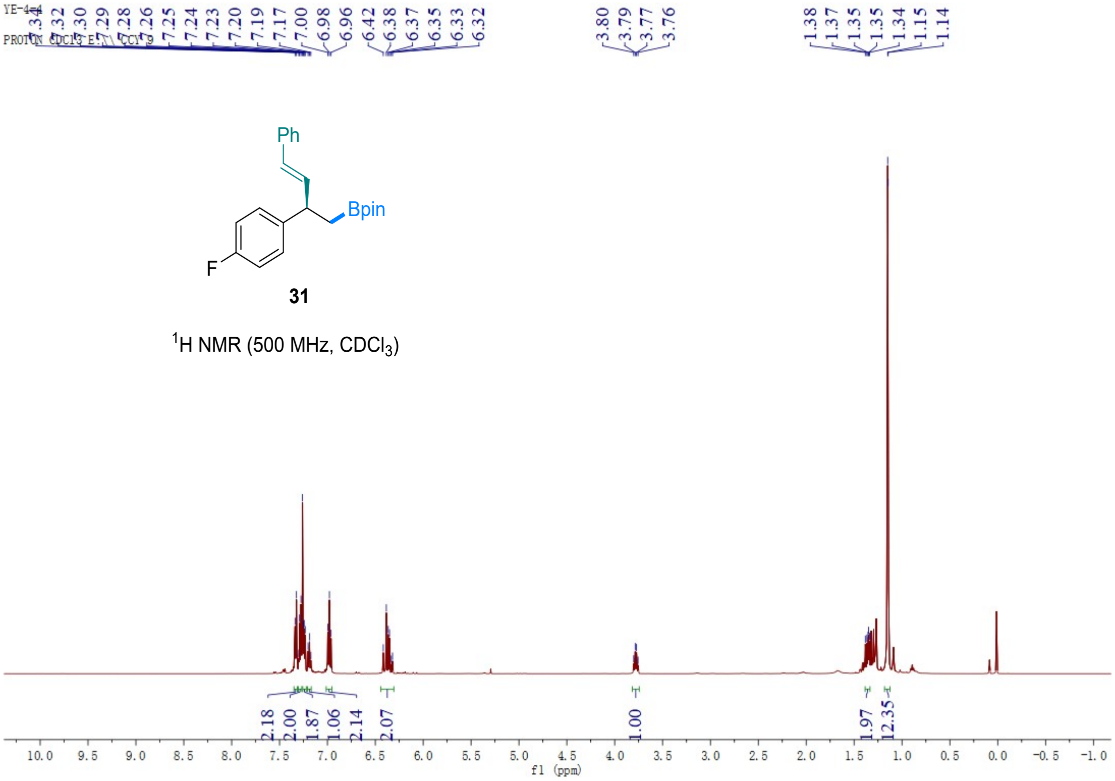
YE-4-4
PROTON CDCl3 E:\\ CCY 9
7.34 7.32 7.30 7.29 7.28 7.26 7.25 7.24 7.23 7.20 7.19 7.17 7.00 6.98 6.96 6.42 6.38 6.37 6.35 6.33 6.32
3.80 3.79 3.77 3.76
1.38 1.37 1.35 1.35 1.34 1.15 1.14
Ph
Bpin
F
31
^{1}H NMR (500 MHz, $CDCl_3$)
2.18 2.00 1.87 1.06 2.14 2.07 1.00 1.97 12.35
10.0 9.5 9.0 8.5 8.0 7.5 7.0 6.5 6.0 5.5 5.0 4.5 4.0 3.5 3.0 2.5 2.0 1.5 1.0 0.5 0.0 -0.5 -1.0
f1 (ppm)

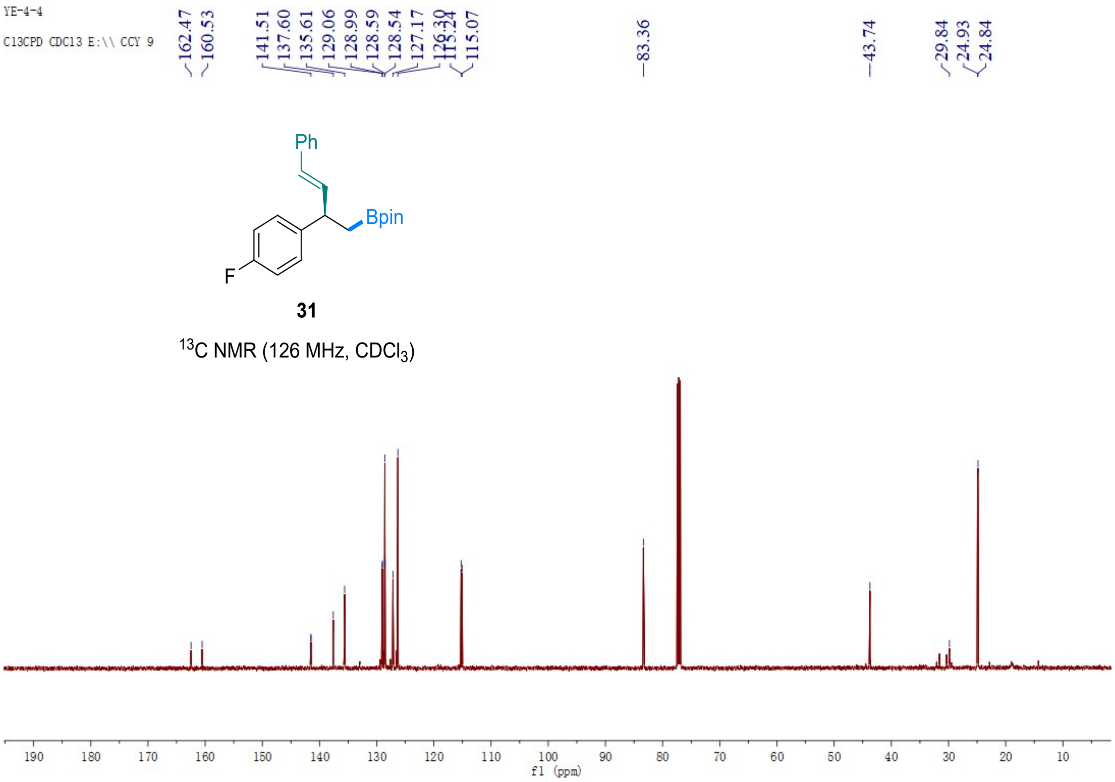
YE-4-4
C13CPD CDCl3 E:\\ CCY 9
162.47 160.53
141.51 137.60 135.61 129.06 128.99 128.59 128.54 127.17 126.30 115.24 115.07
83.36
43.74
29.84 24.93 24.84
Ph
Bpin
F
31
^{13}C NMR (126 MHz, $CDCl_3$)
190 180 170 160 150 140 130 120 110 100 90 80 70 60 50 40 30 20 10
f1 (ppm)

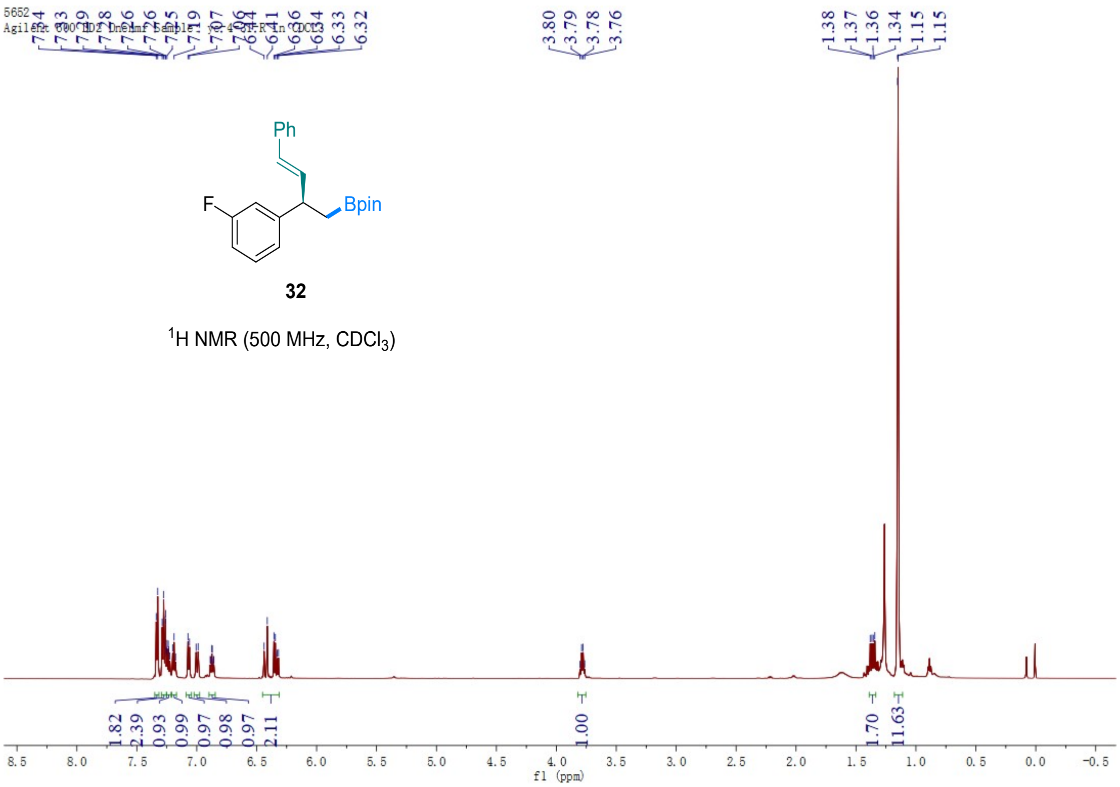

3.80
3.79
3.78
3.76
1.38
1.37
1.36
1.34
1.15
1.15
Ph
F
Bpin
32
1H NMR (500 MHz, CDCl3)
1.82
2.39
0.93
0.99
0.97
0.98
0.97
2.11
1.00
1.70
11.63
f1 (ppm)

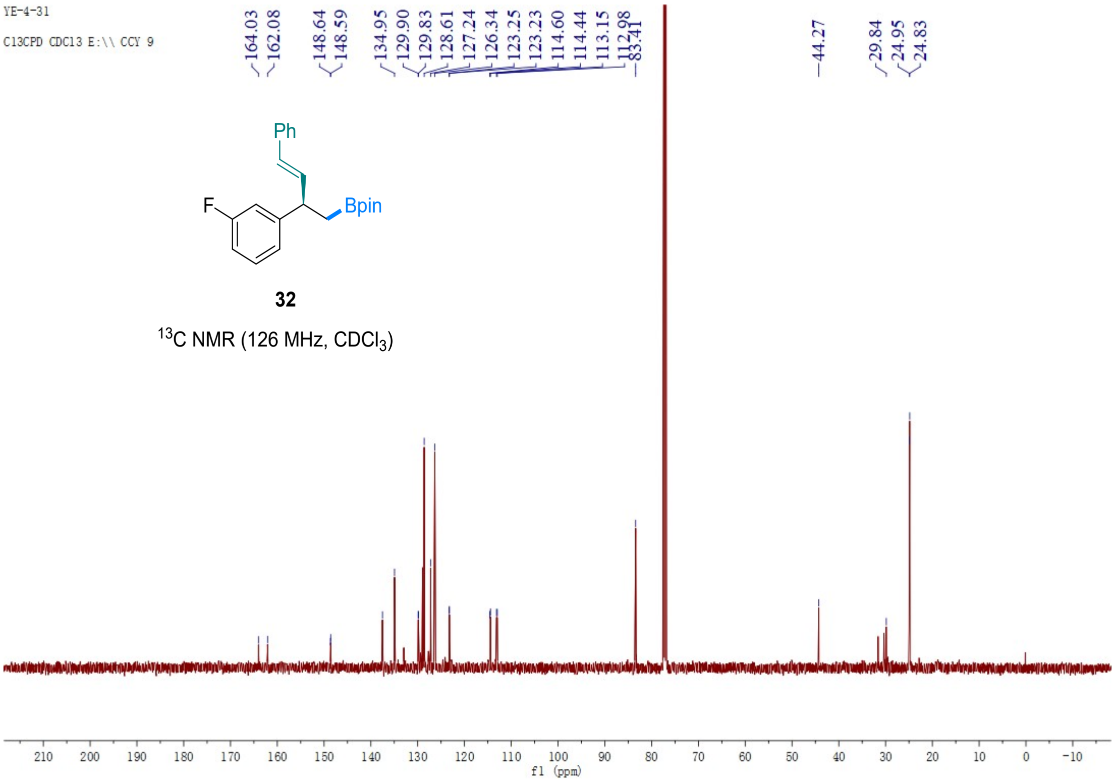

YE-4-31
C13CPD CDCl3 E:\\ CCY 9
164.03
162.08
148.64
148.59
134.95
129.90
129.83
128.61
127.24
126.34
123.25
123.23
114.60
114.44
113.15
112.98
83.41
44.27
29.84
24.95
24.83
Ph
F
Bpin
32
13C NMR (126 MHz, CDCl3)
f1 (ppm)

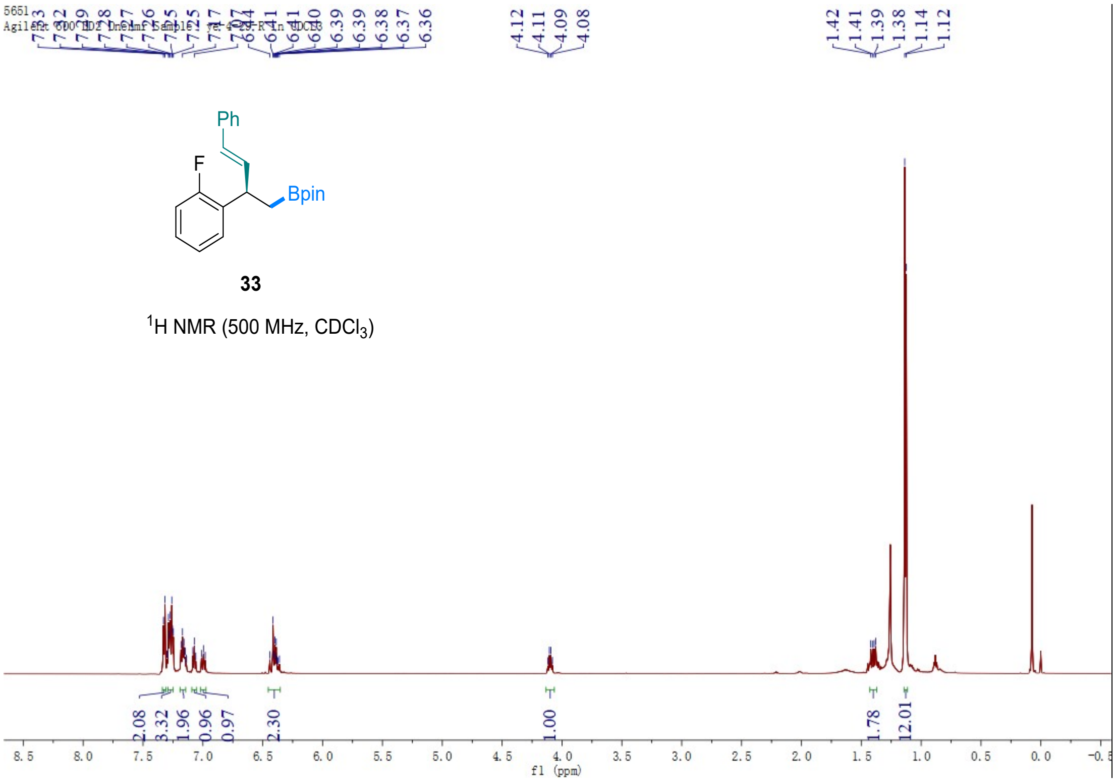

33

^{1}H NMR (500 MHz, $CDCl_3$)

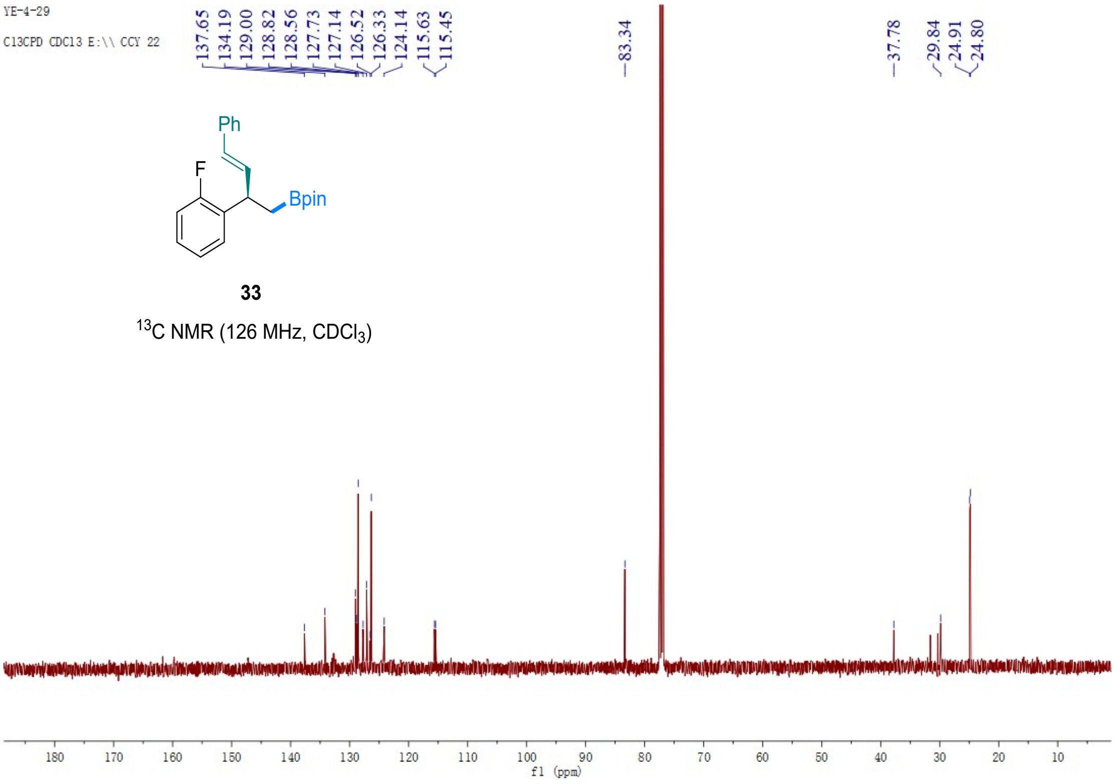

33

^{13}C NMR (126 MHz, $CDCl_3$)

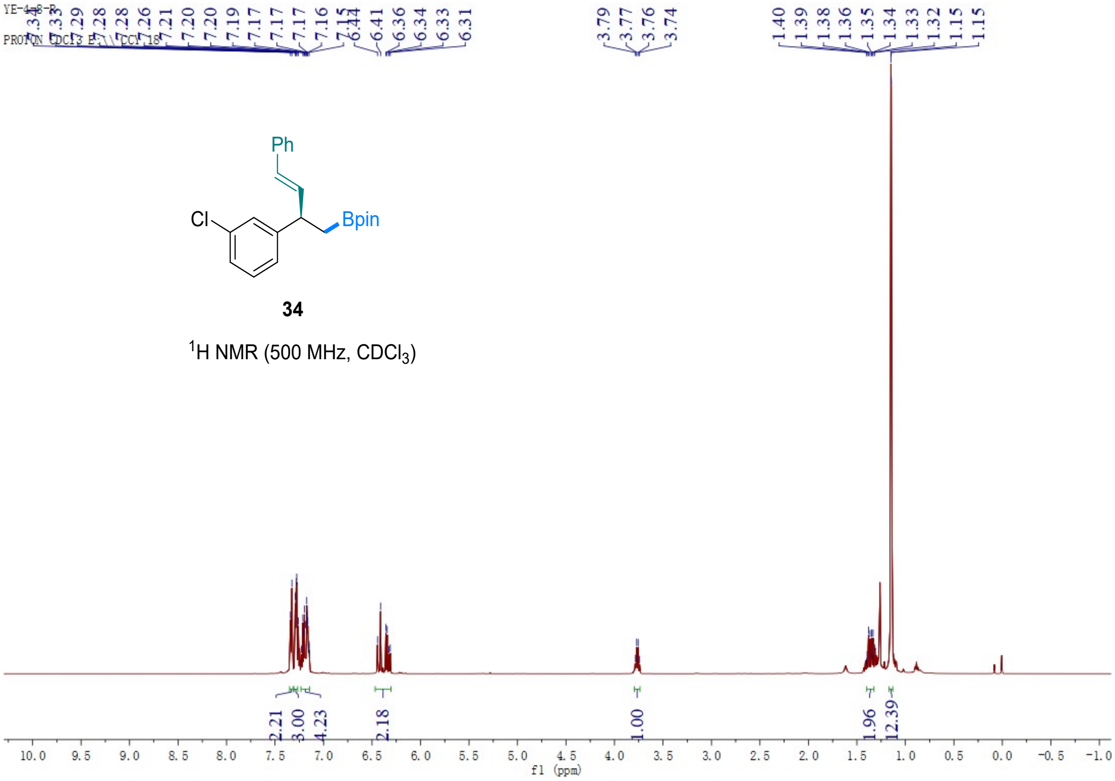

34

^{1}H NMR (500 MHz, $CDCl_3$)

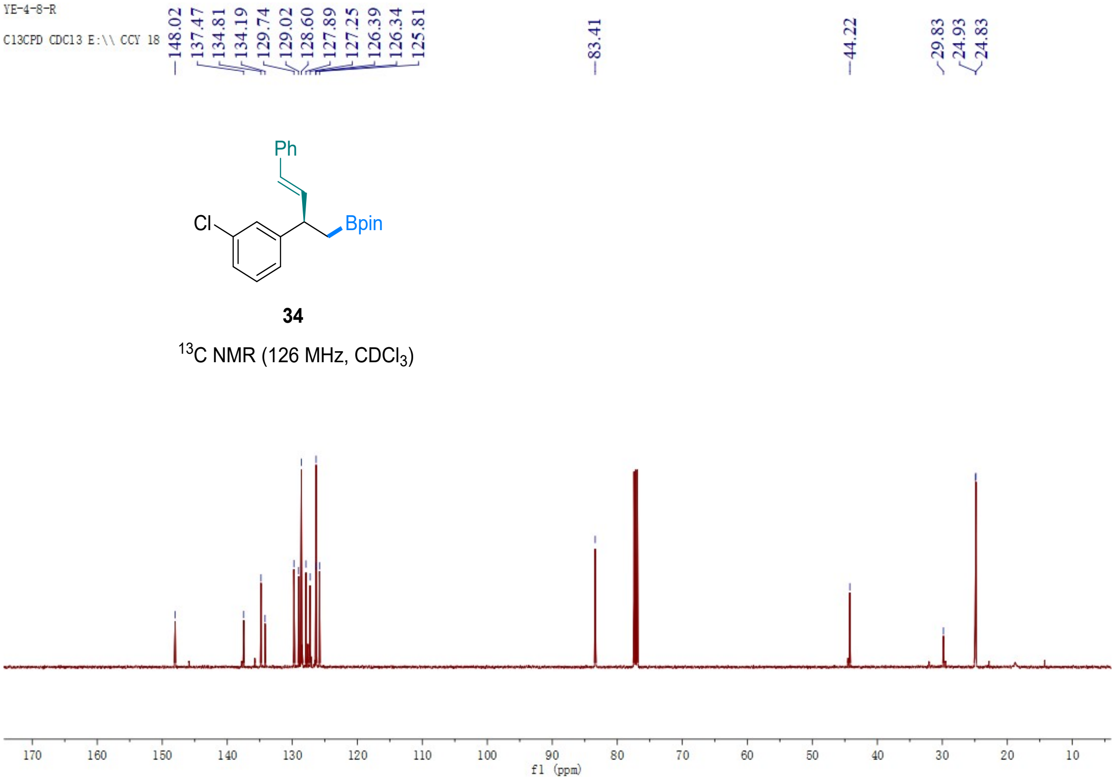

34

^{13}C NMR (126 MHz, $CDCl_3$)

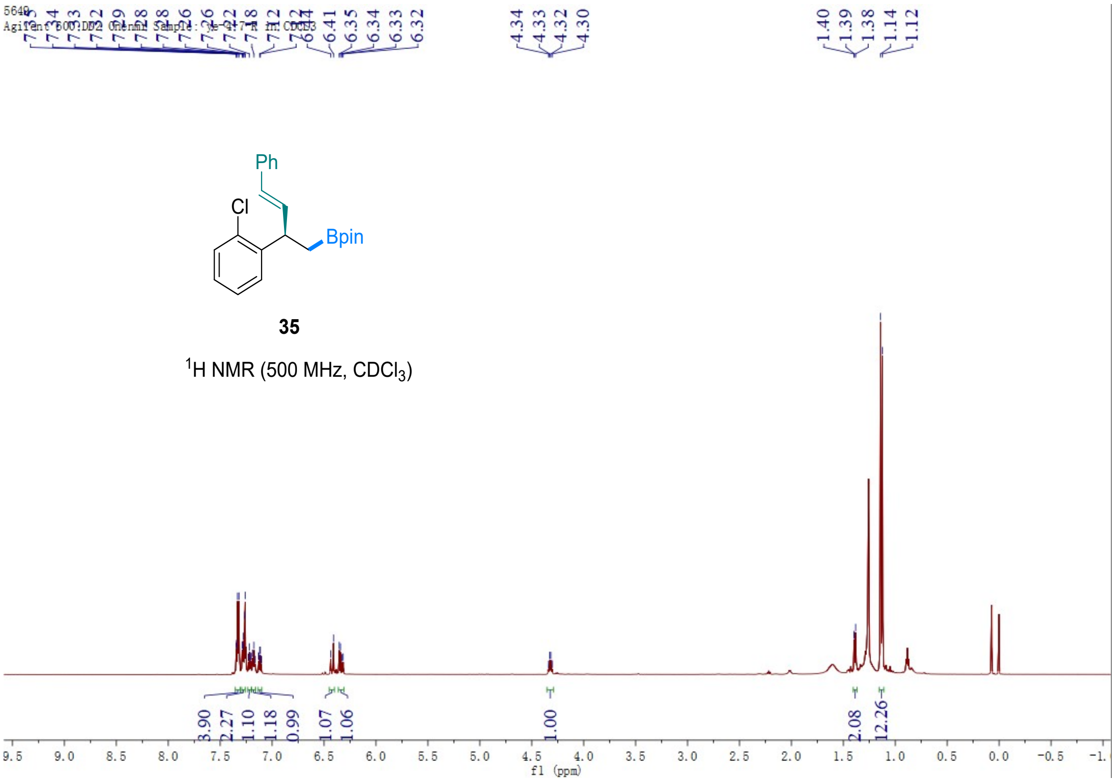

35

^{1}H NMR (500 MHz, $CDCl_3$)

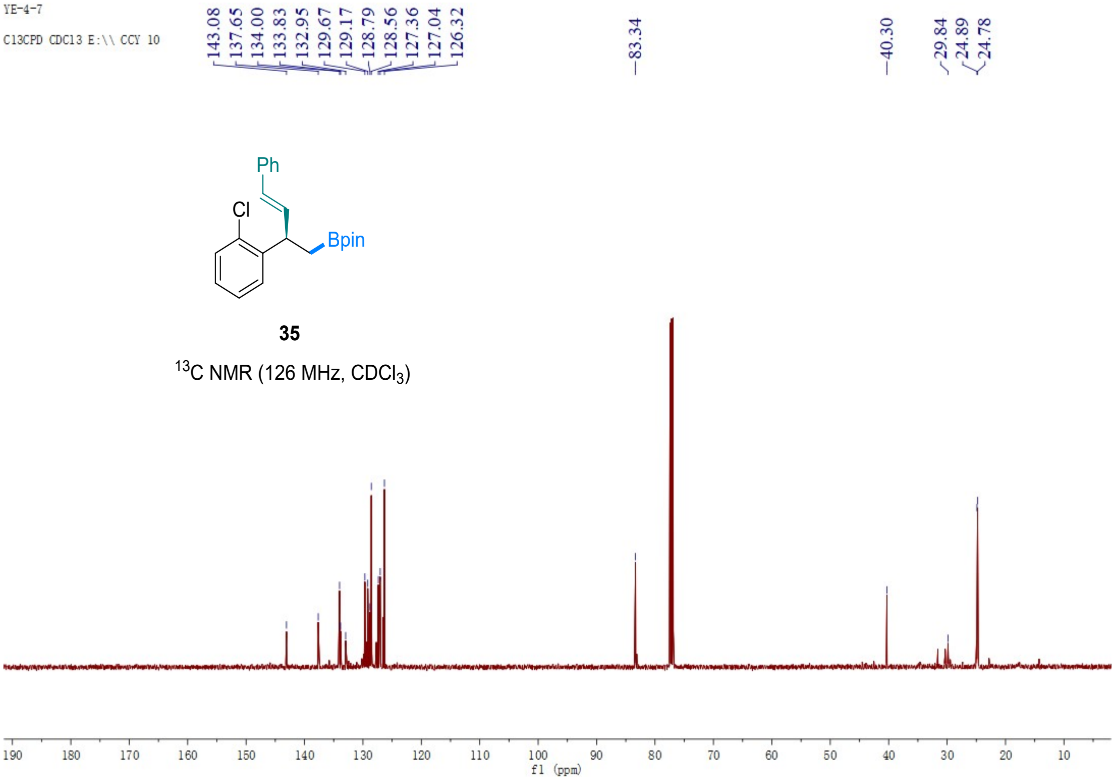

35

^{13}C NMR (126 MHz, $CDCl_3$)

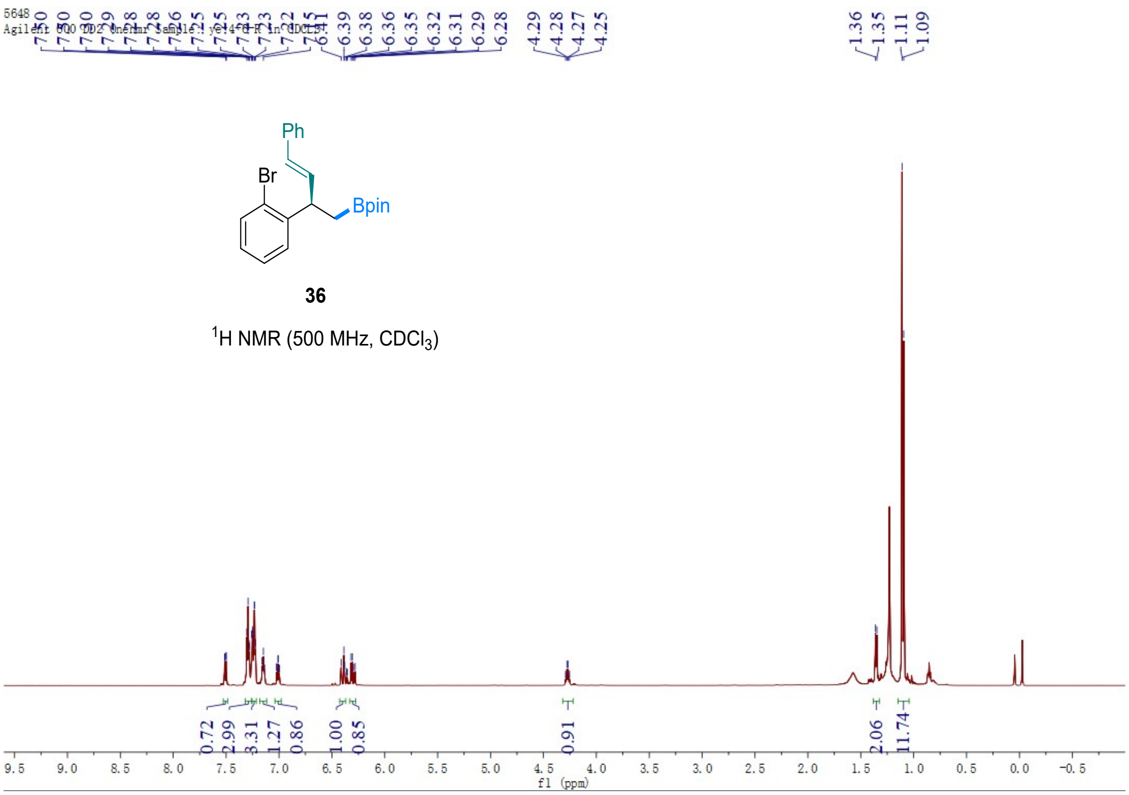
5648
7.50
7.50
7.30
7.29
7.28
7.28
7.26
7.25
7.25
7.23
7.23
7.22
7.15
6.41
6.39
6.38
6.36
6.35
6.32
6.31
6.29
6.28
4.29
4.28
4.27
4.25
1.36
1.35
1.11
1.09
Ph
Br
Bpin
36
1H NMR (500 MHz, CDCl3)
0.72
2.99
3.31
1.27
0.86
1.00
0.85
0.91
2.06
11.74
9.5 9.0 8.5 8.0 7.5 7.0 6.5 6.0 5.5 5.0 4.5 4.0 3.5 3.0 2.5 2.0 1.5 1.0 0.5 0.0 -0.5
f1 (ppm)

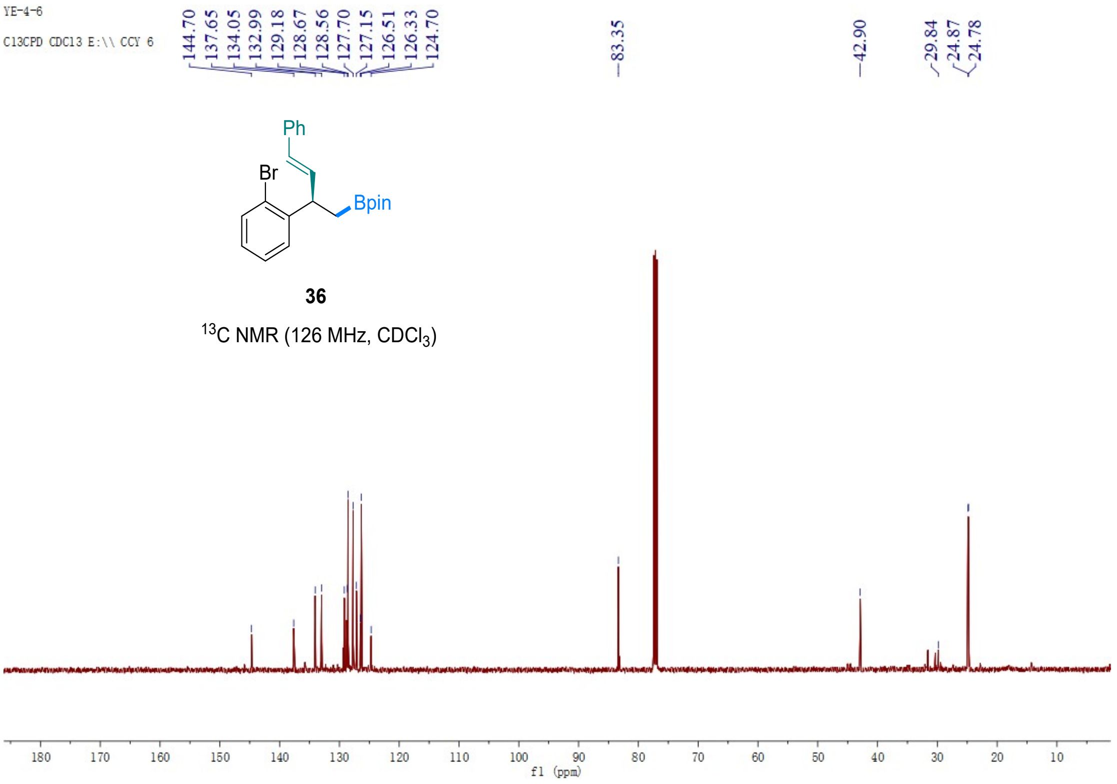
YE-4-6
C13CPD CDCl3 E:\\ CCY 6
144.70
137.65
134.05
132.99
129.18
128.67
128.56
127.70
127.15
126.51
126.33
124.70
83.35
42.90
29.84
24.87
24.78
Ph
Br
Bpin
36
13C NMR (126 MHz, CDCl3)
180 170 160 150 140 130 120 110 100 90 80 70 60 50 40 30 20 10
f1 (ppm)

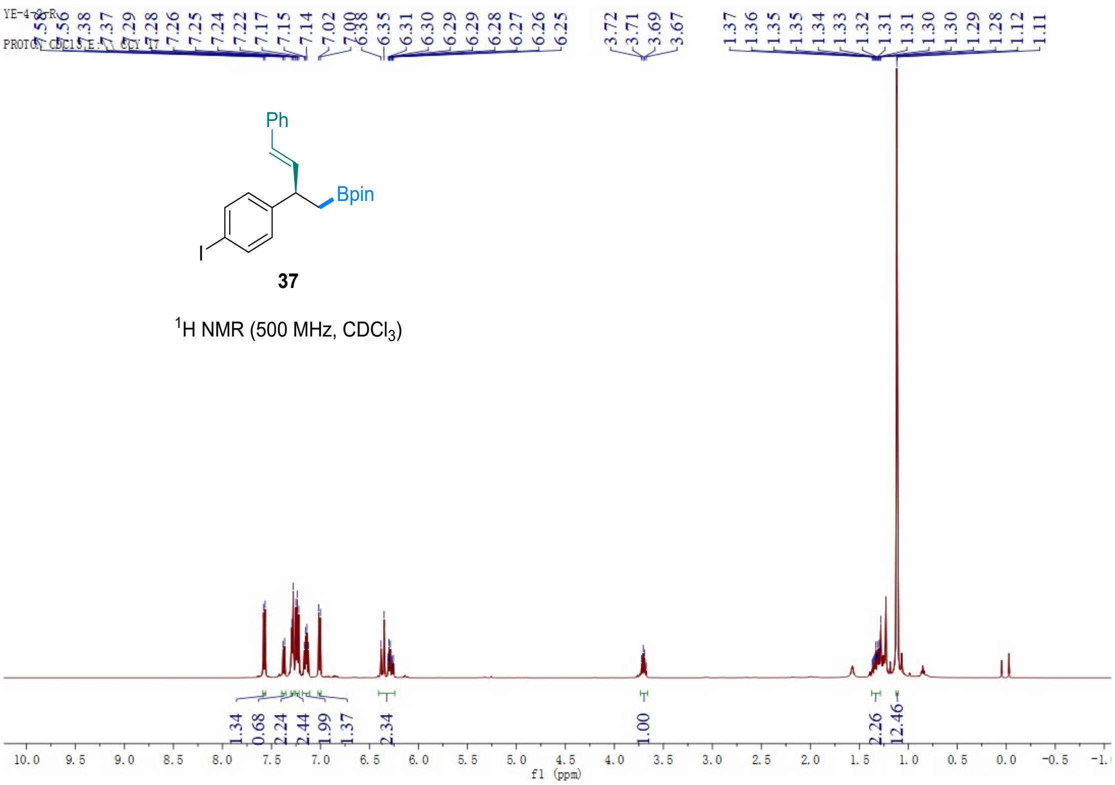

37

^{1}H NMR (500 MHz, $CDCl_3$)

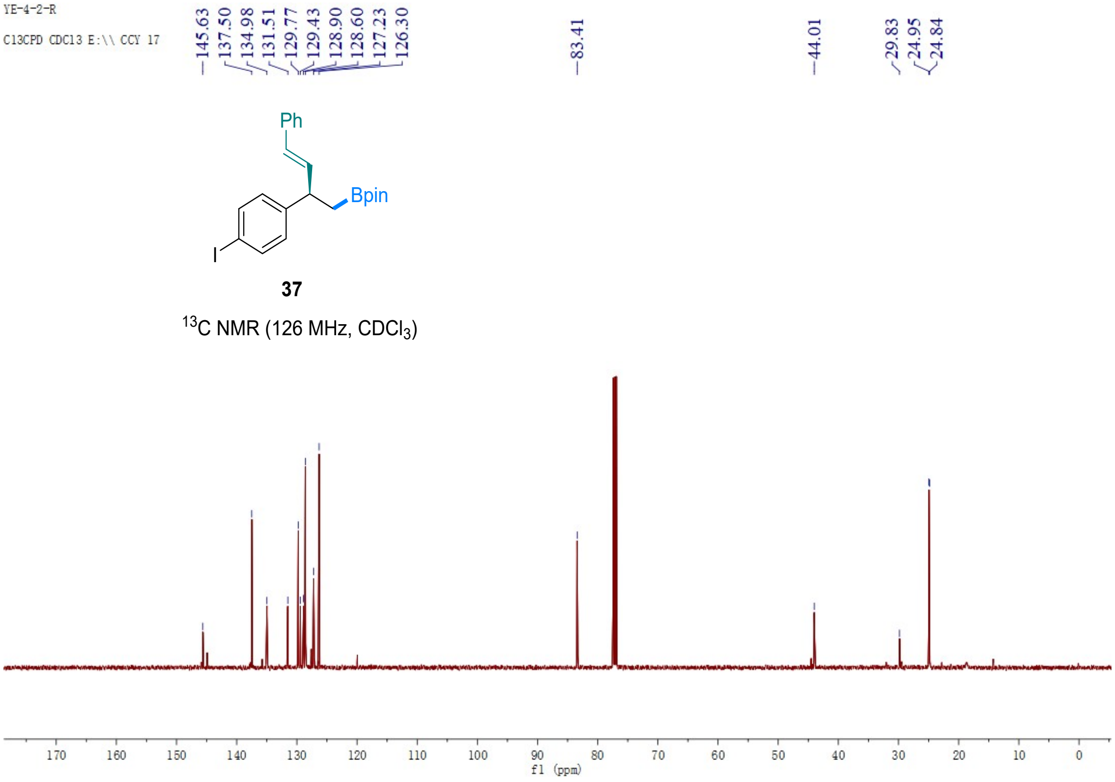

37

^{13}C NMR (126 MHz, $CDCl_3$)

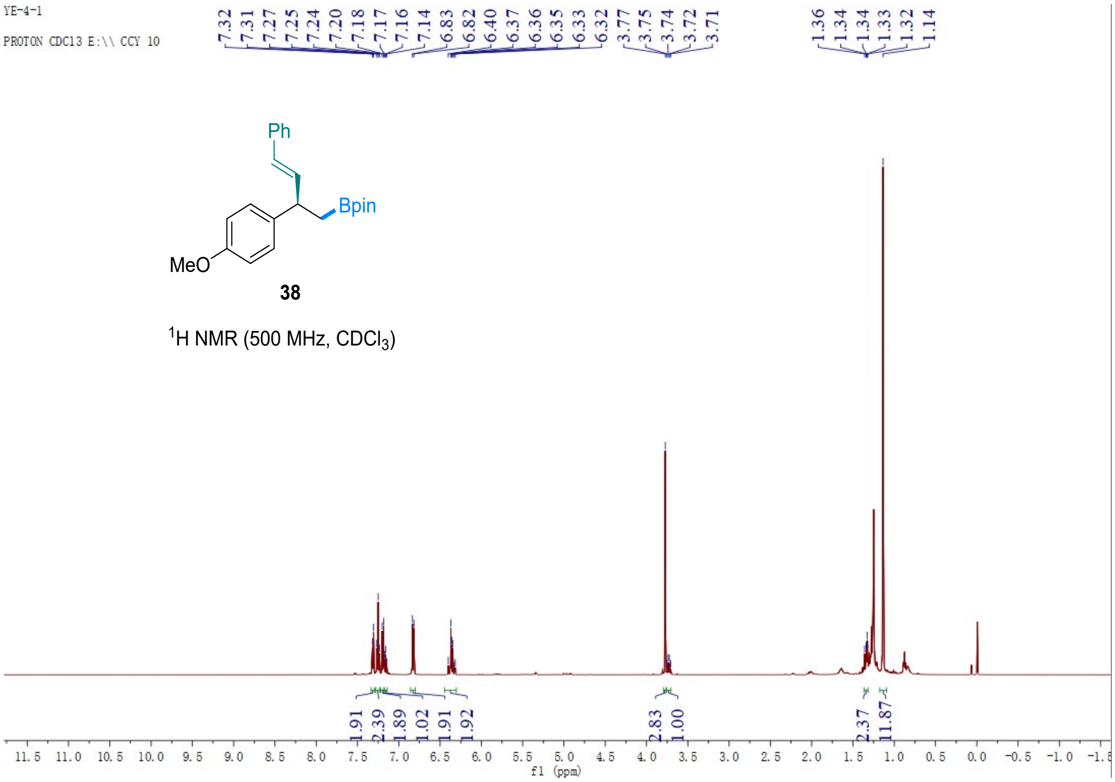
YE-4-1
PROTON CDCl3 E:\\ CCY 10
7.32
7.31
7.27
7.25
7.24
7.20
7.18
7.17
7.16
7.14
6.83
6.82
6.40
6.37
6.36
6.35
6.33
6.32
3.77
3.75
3.74
3.72
3.71
1.36
1.34
1.34
1.33
1.32
1.14
Ph
Bpin
MeO
38
1H NMR (500 MHz, CDCl3)
1.91
2.39
1.89
1.02
1.91
1.92
2.83
1.00
2.37
11.87
f1 (ppm)

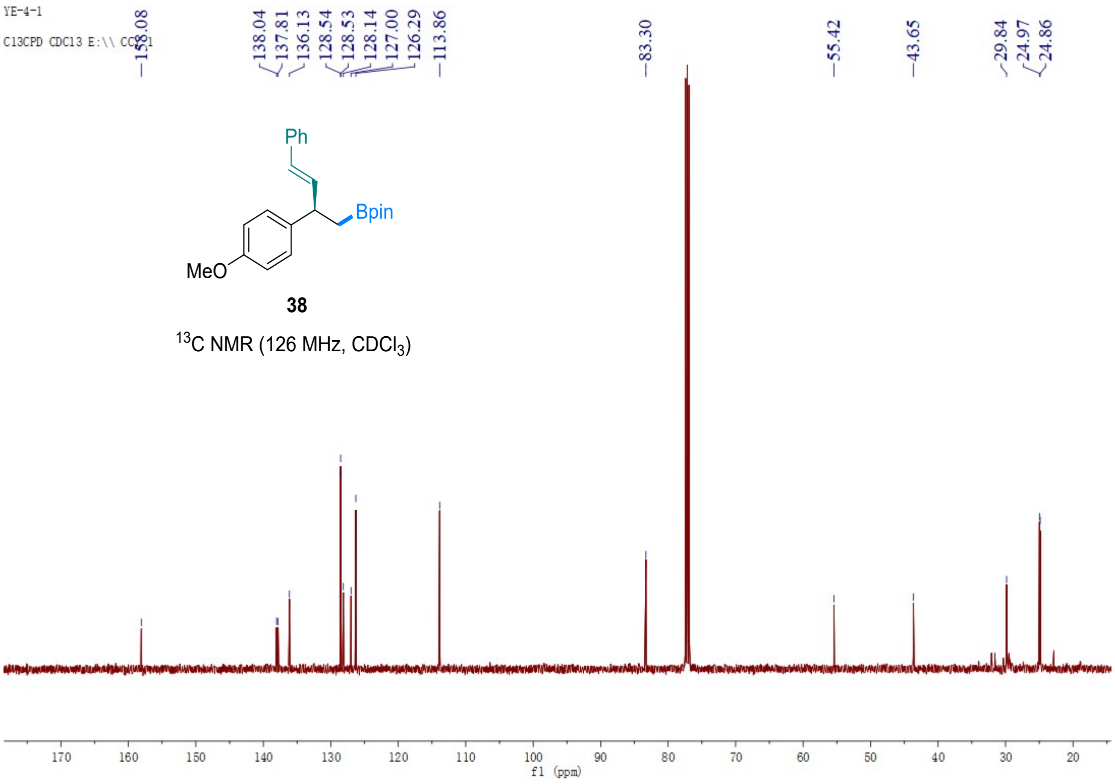
YE-4-1
C13CPD CDCl3 E:\\ CCY 11
158.08
138.04
137.81
136.13
128.54
128.53
128.14
127.00
126.29
113.86
83.30
55.42
43.65
29.84
24.97
24.86
Ph
Bpin
MeO
38
13C NMR (126 MHz, CDCl3)
f1 (ppm)

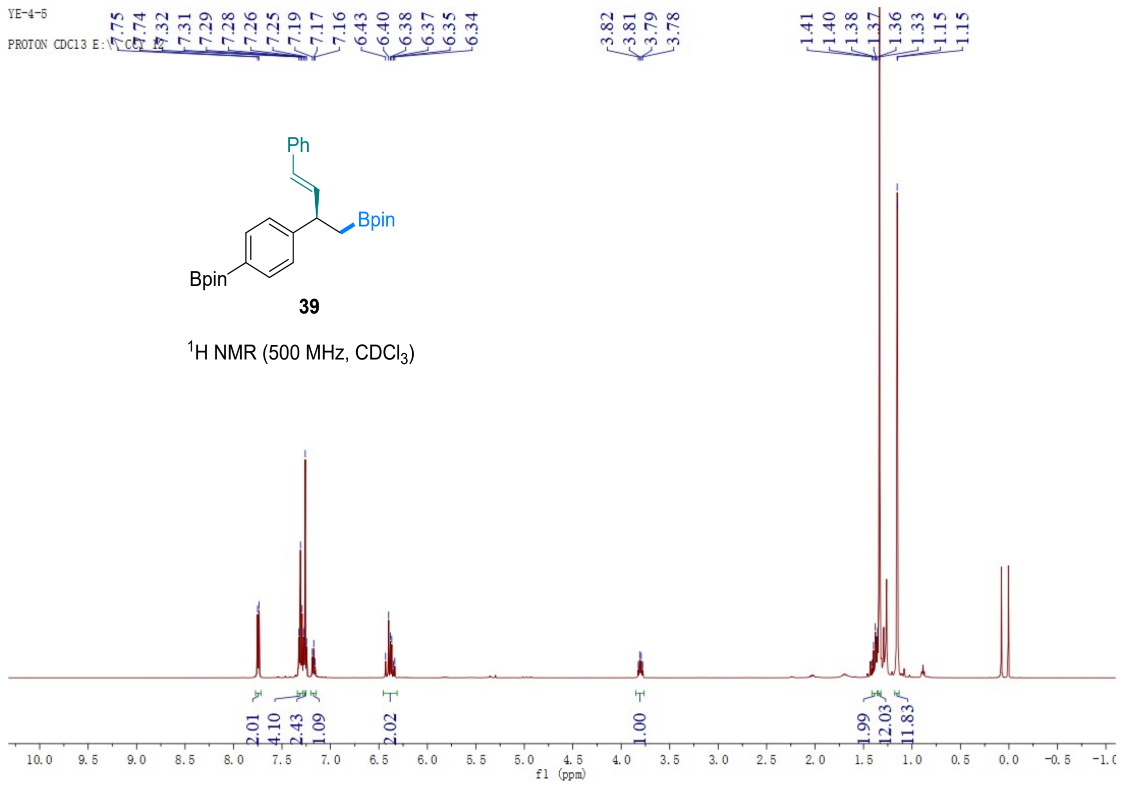
YE-4-5
PROTON CDCl3 E:\\ CCY 12
7.75
7.74
7.32
7.31
7.29
7.28
7.26
7.25
7.19
7.17
7.16
6.43
6.40
6.38
6.37
6.35
6.34
3.82
3.81
3.79
3.78
1.41
1.40
1.38
1.37
1.36
1.33
1.15
1.15
Ph
Bpin
Bpin
39
1H NMR (500 MHz, CDCl3)
2.01
4.10
2.43
1.09
2.02
1.00
1.99
12.03
11.83
f1 (ppm)

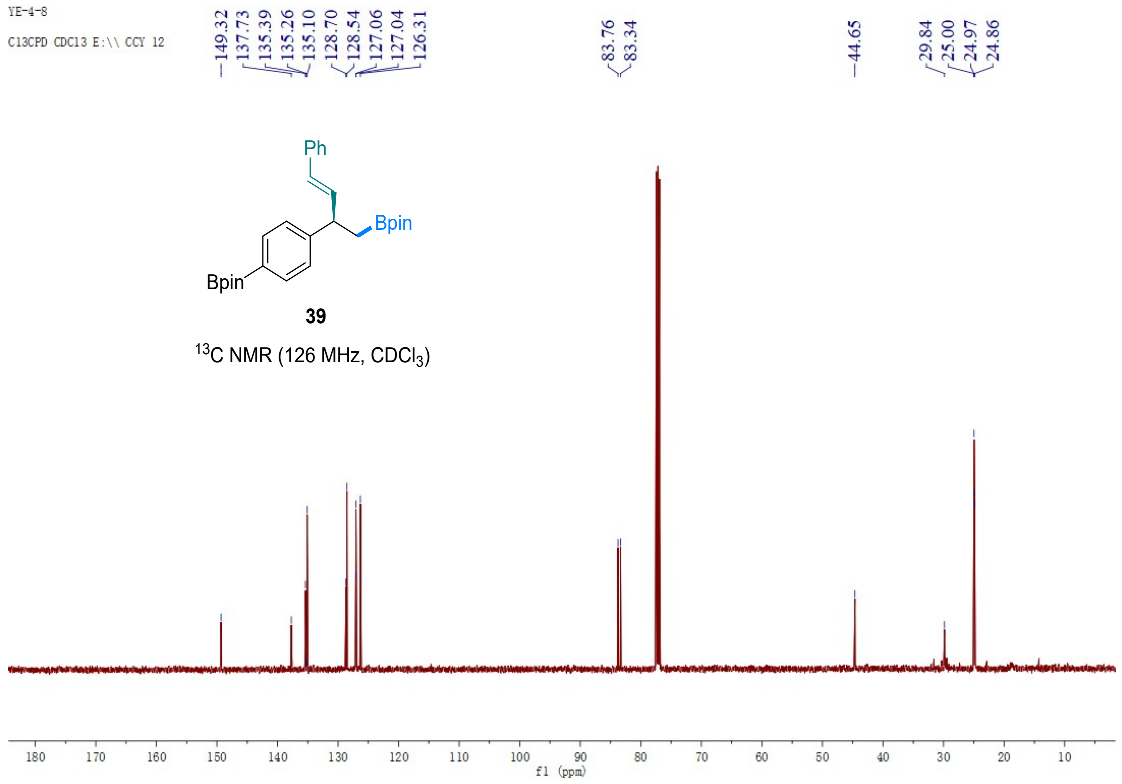
YE-4-8
C13CPD CDCl3 E:\\ CCY 12
149.32
137.73
135.39
135.26
135.10
128.70
128.54
127.06
127.04
126.31
83.76
83.34
44.65
29.84
25.00
24.97
24.86
Ph
Bpin
Bpin
39
13C NMR (126 MHz, CDCl3)
f1 (ppm)

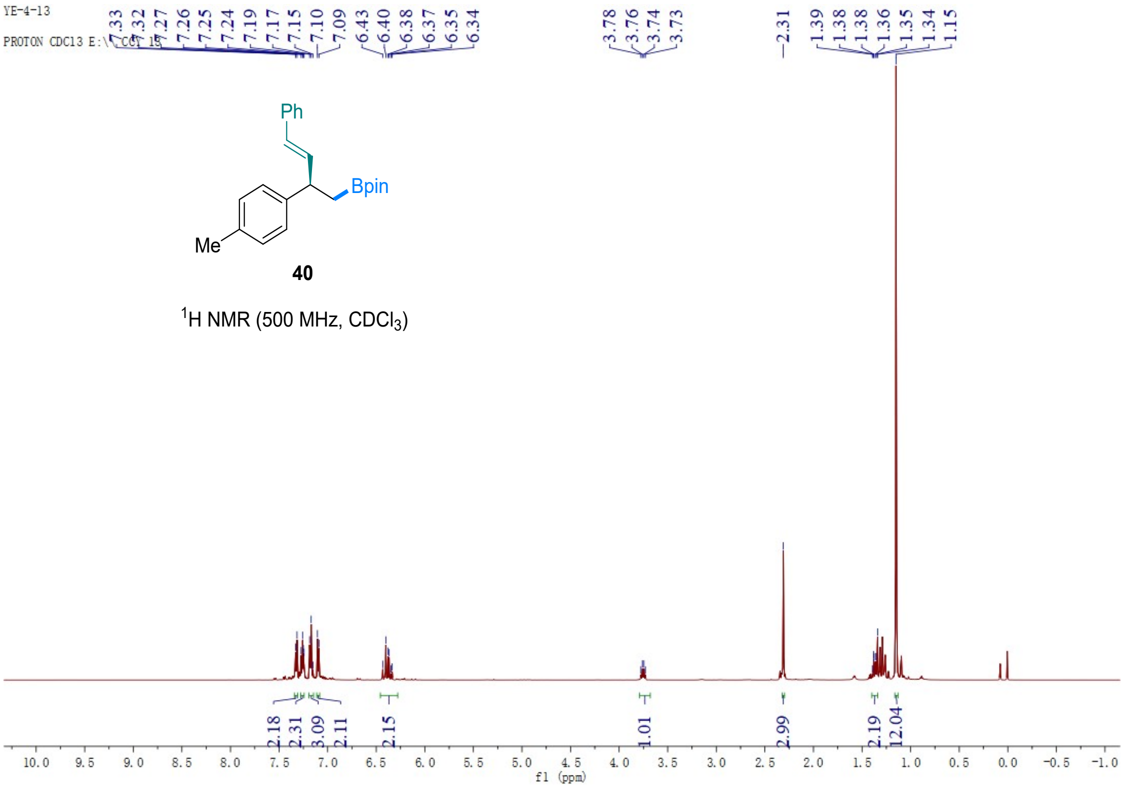

^{1}H NMR (500 MHz, $CDCl_3$)

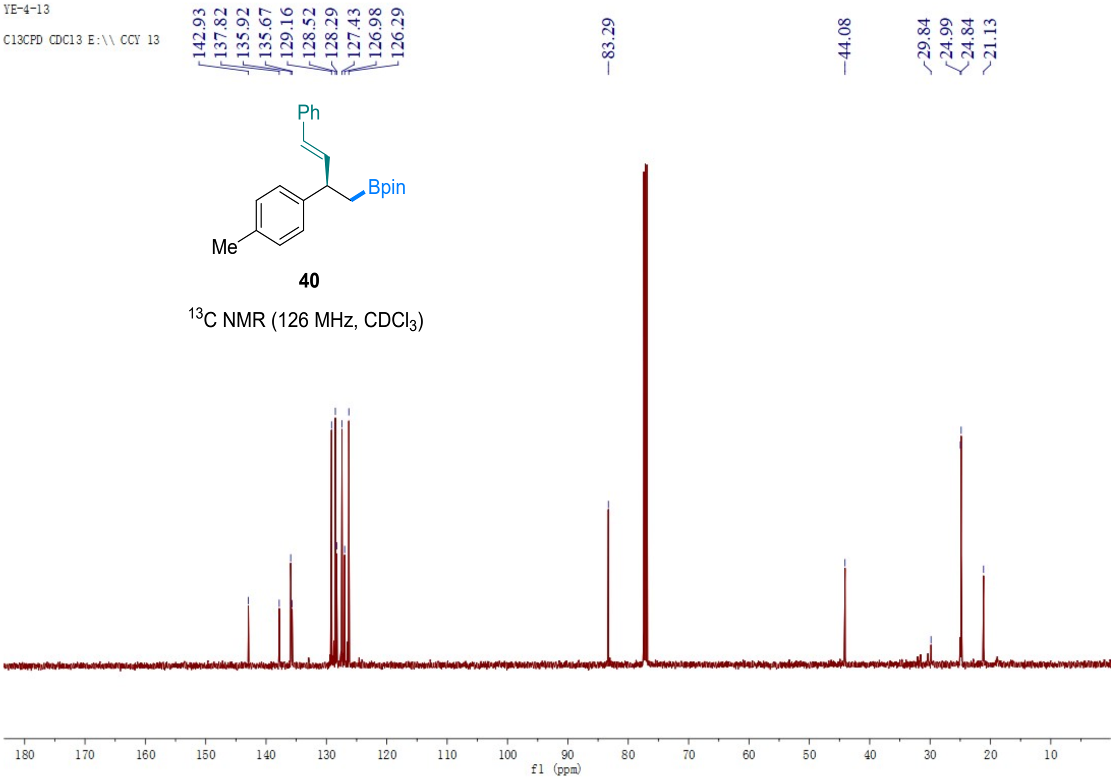

^{13}C NMR (126 MHz, $CDCl_3$)

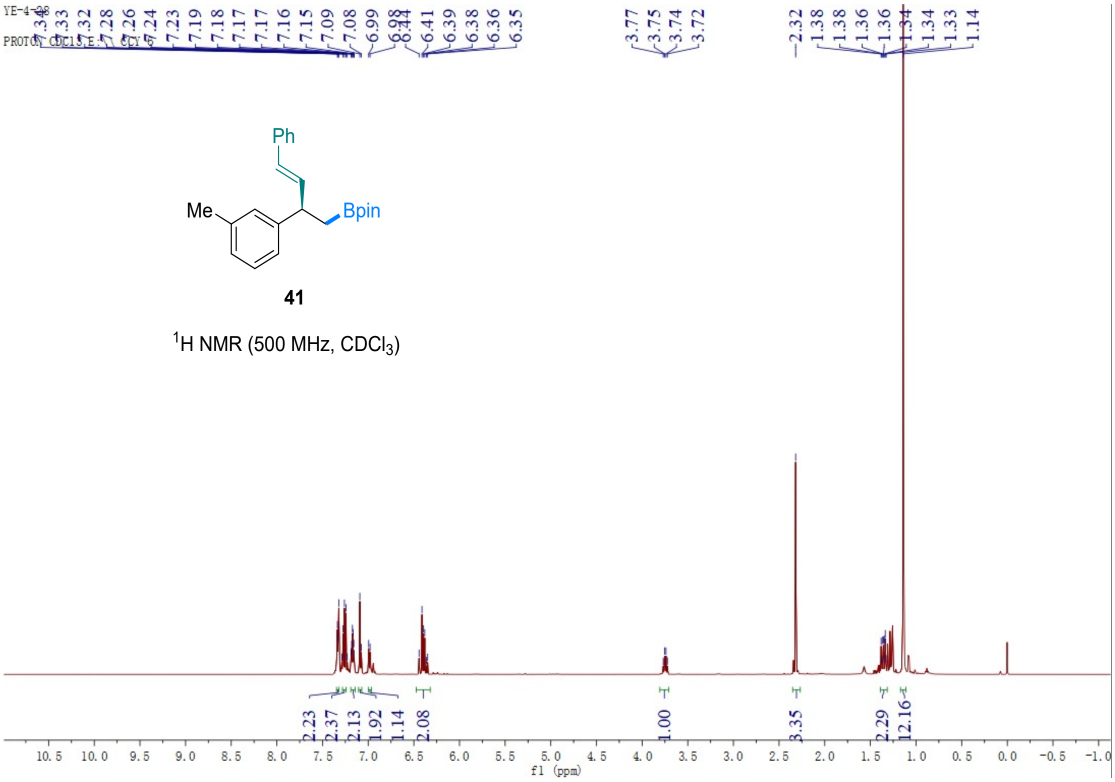

41

^{1}H NMR (500 MHz, $CDCl_3$)

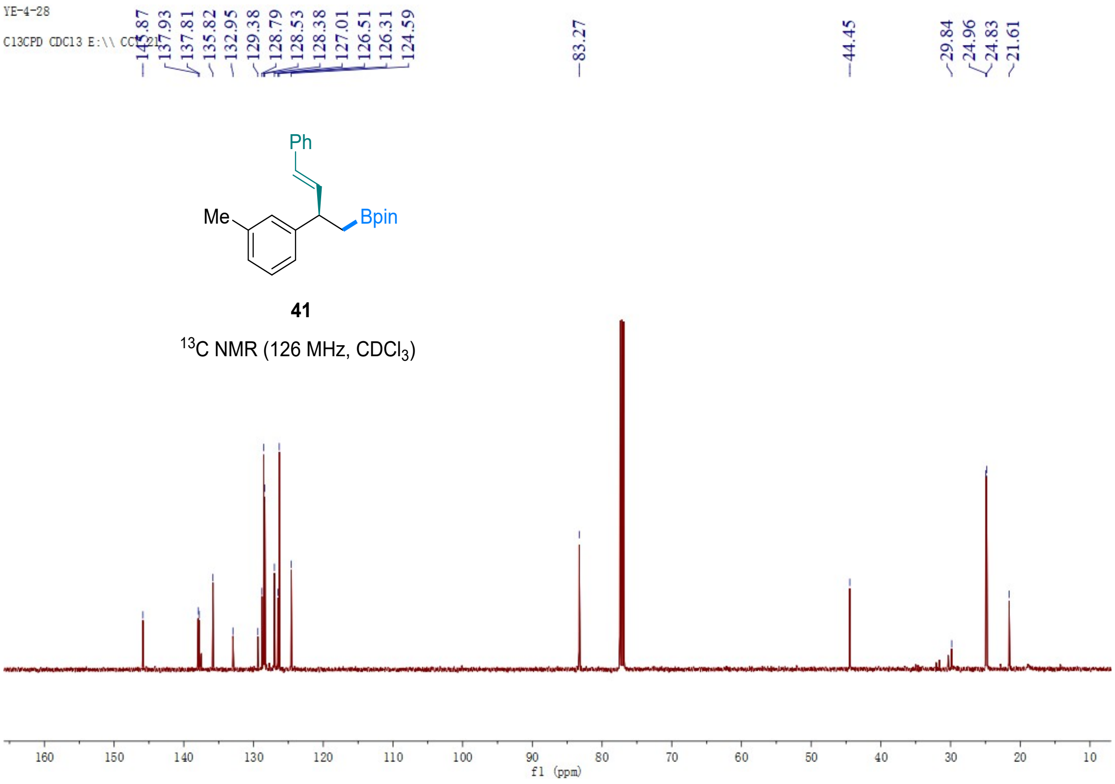

41

^{13}C NMR (126 MHz, $CDCl_3$)

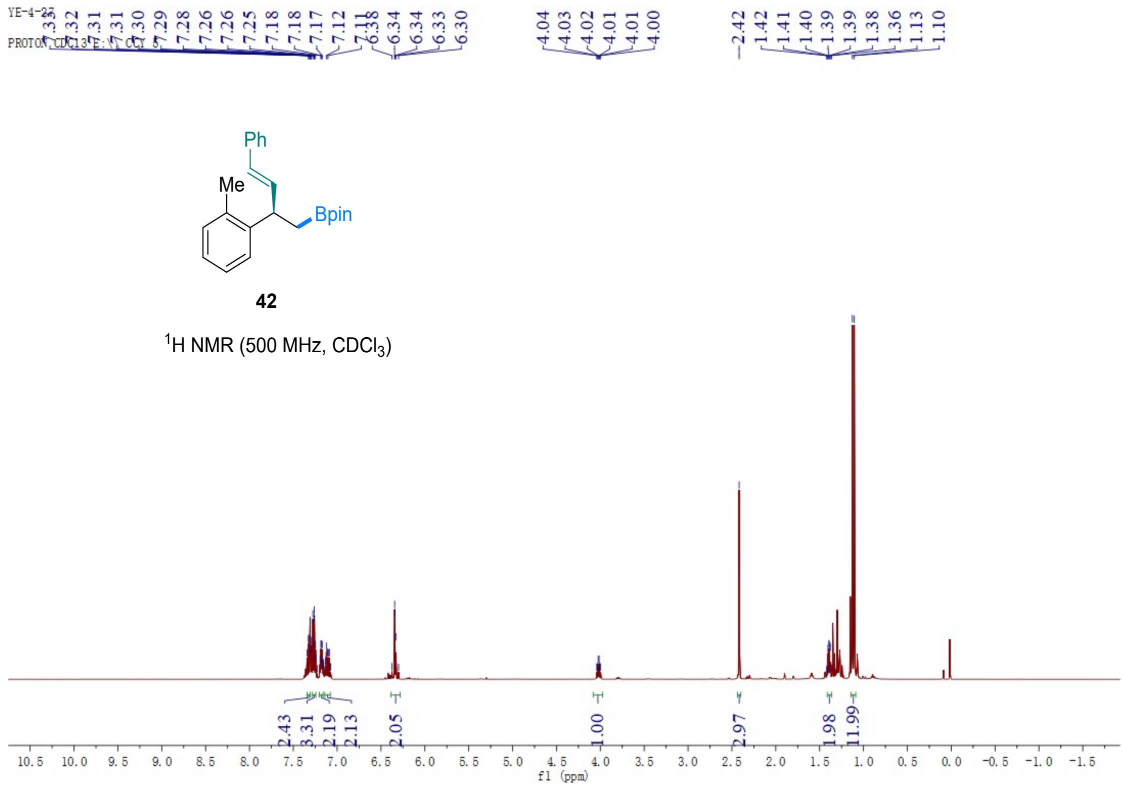

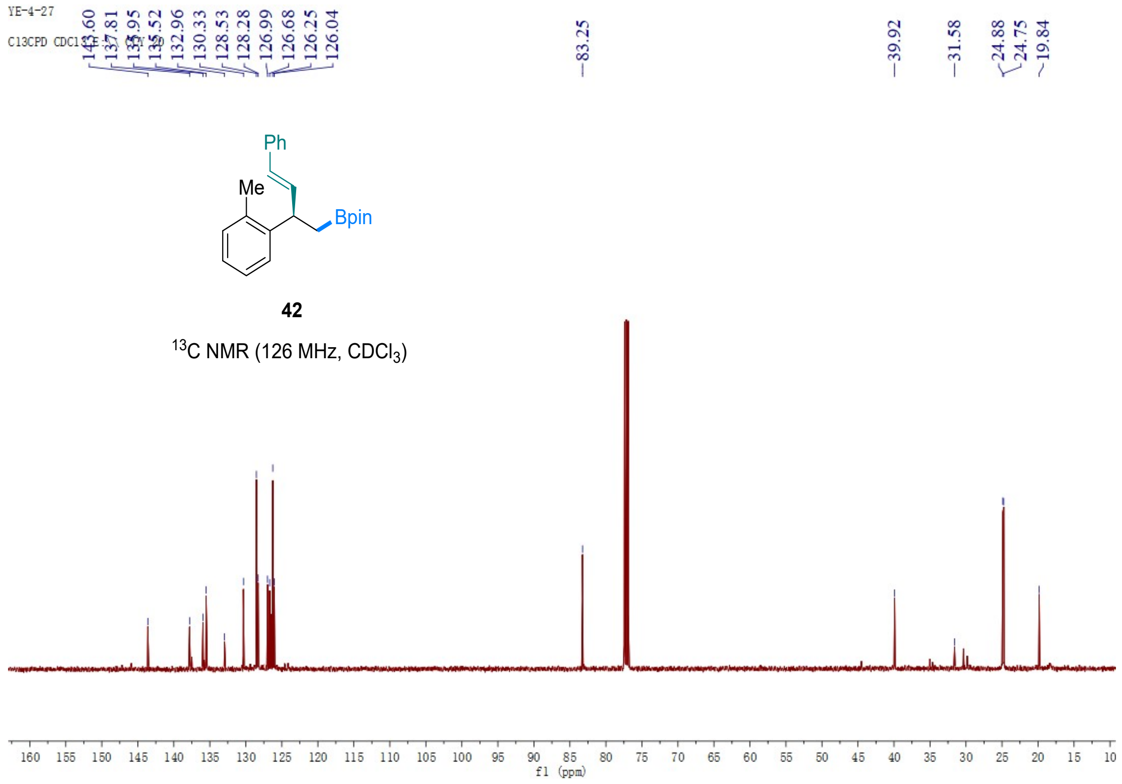

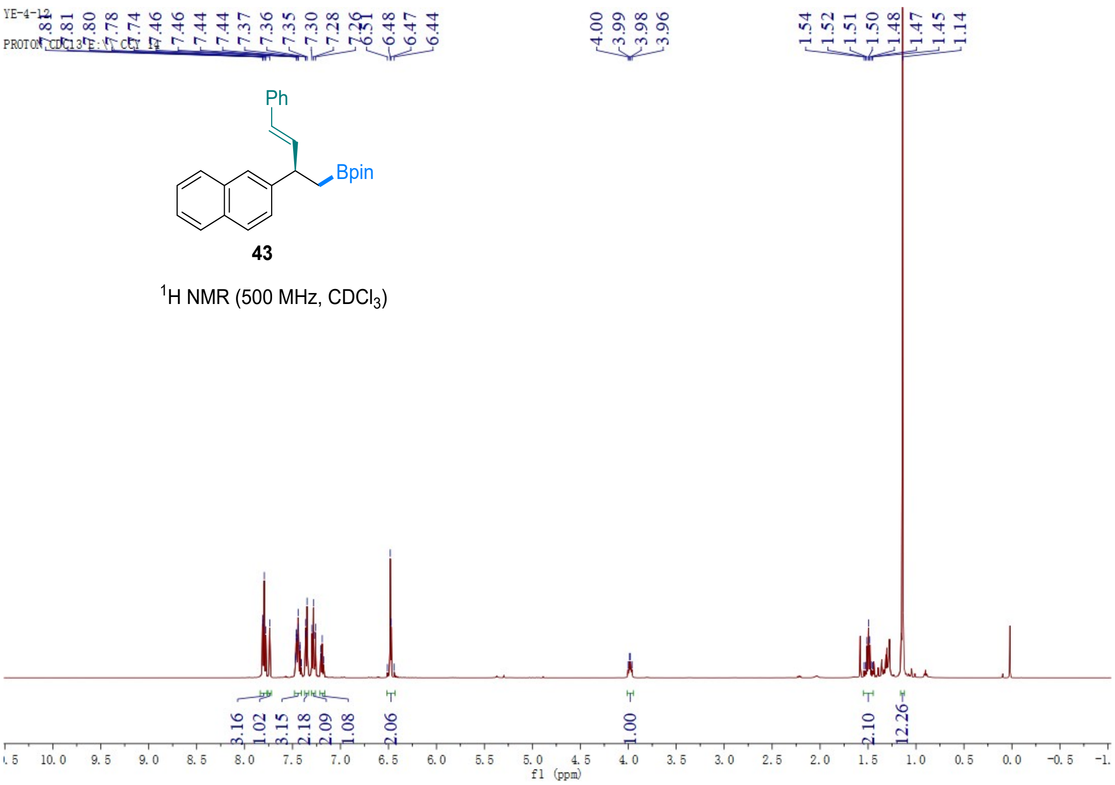

^{1}H NMR (500 MHz, $CDCl_3$)

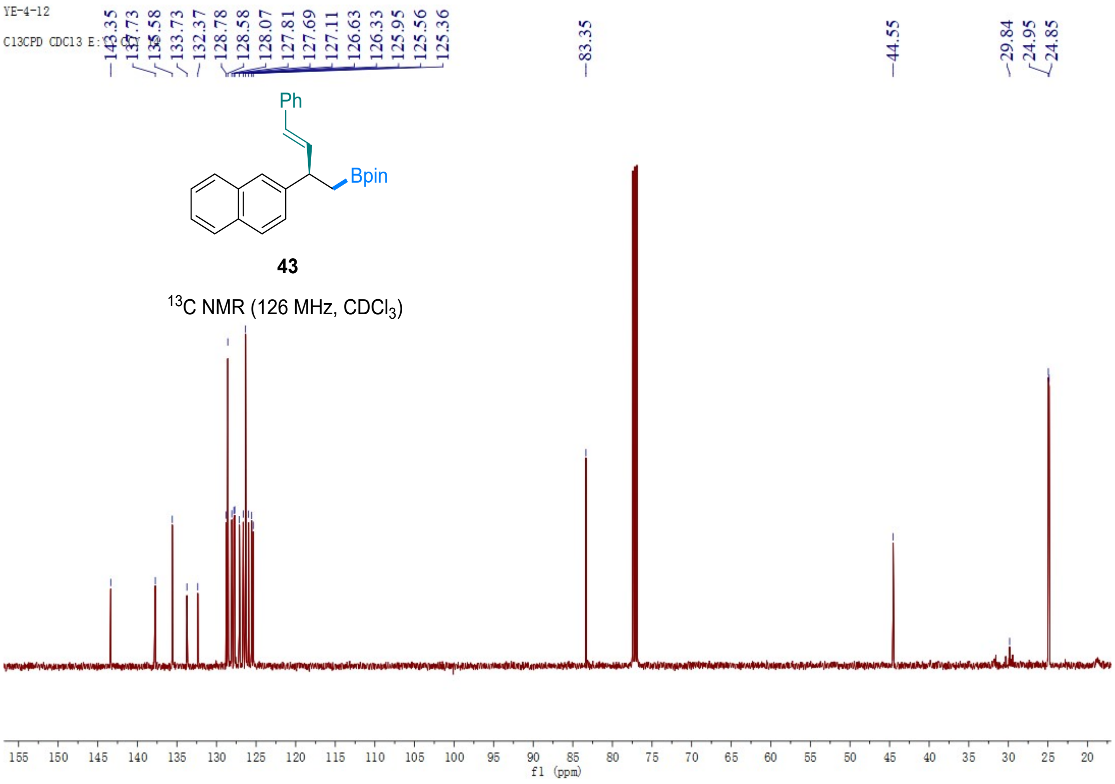

^{13}C NMR (126 MHz, $CDCl_3$)

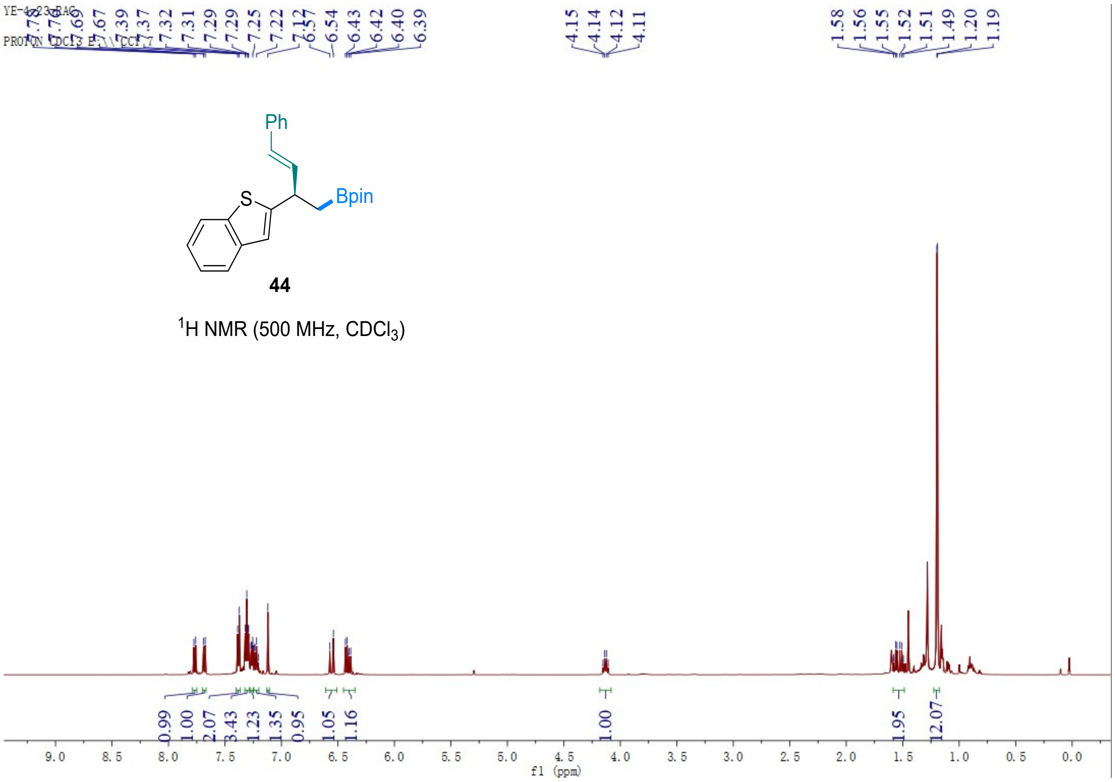
Ph
Bpin
S
44
1H NMR (500 MHz, CDCl3)
4.15
4.14
4.12
4.11
1.58
1.56
1.55
1.52
1.51
1.49
1.20
1.19
0.99
1.00
2.07
3.43
1.23
1.35
0.95
1.05
1.16
1.00
1.95
12.07
f1 (ppm)

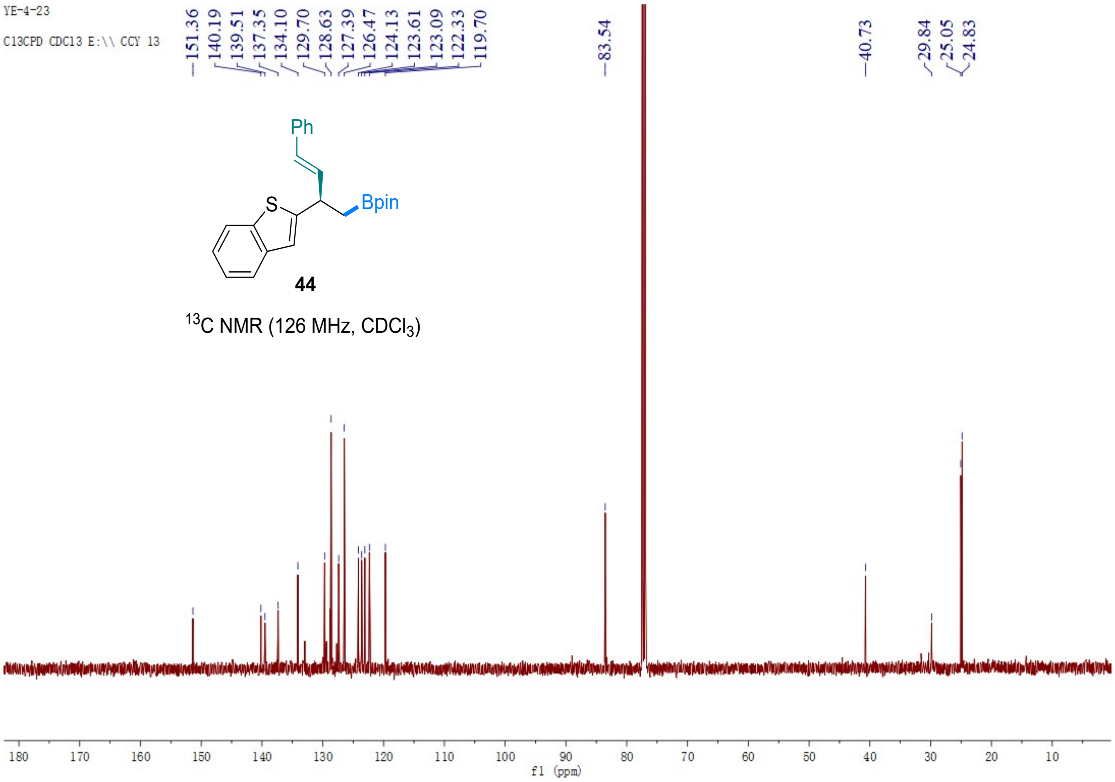
YE-4-23
C13CPD CDCl3 E:\\ CCY 13
151.36
140.19
139.51
137.35
134.10
129.70
128.63
127.39
126.47
124.13
123.61
123.09
122.33
119.70
83.54
40.73
29.84
25.05
24.83
Ph
Bpin
S
44
13C NMR (126 MHz, CDCl3)
f1 (ppm)

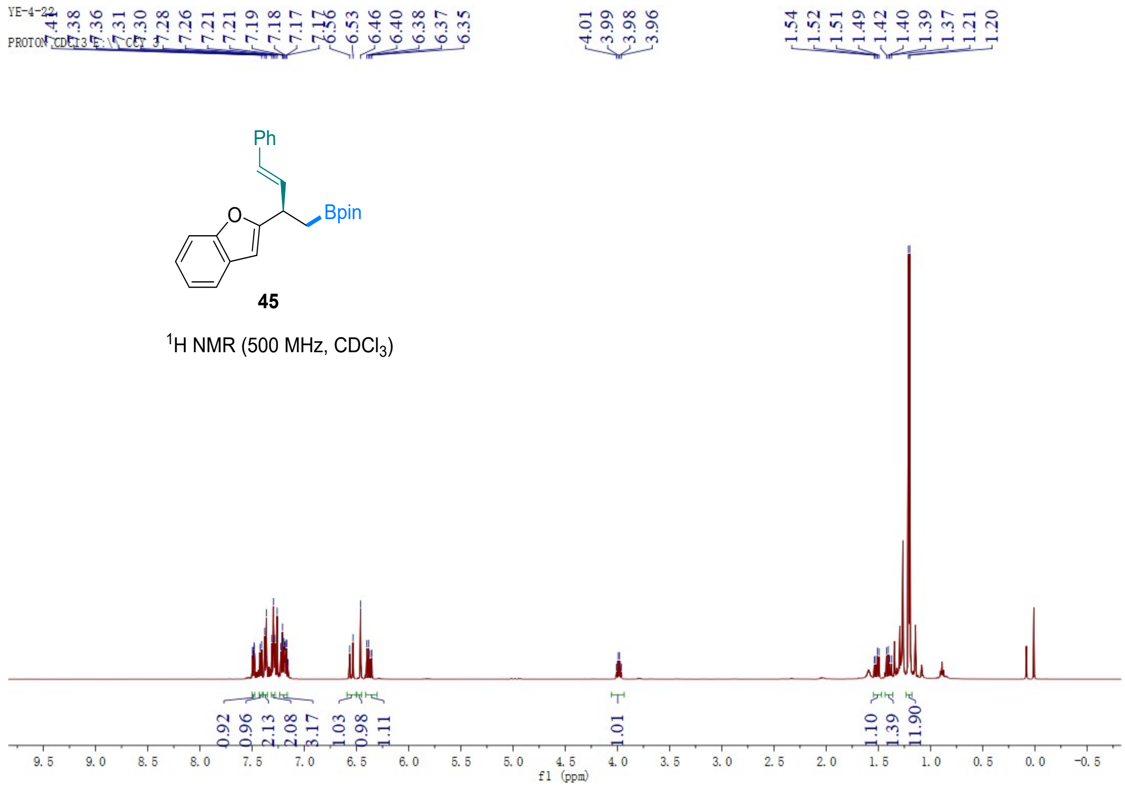
YE-4-22
7.41 7.38 7.36 7.31 7.30 7.28 7.26 7.21 7.21 7.19 7.18 7.17 7.17 6.56 6.53 6.46 6.40 6.38 6.37 6.35
4.01 3.99 3.98 3.96
1.54 1.52 1.51 1.49 1.42 1.40 1.39 1.37 1.21 1.20
Ph
O
Bpin
45
^{1}H NMR (500 MHz, $CDCl_3$)
0.92 0.96 2.13 2.08 3.17 1.03 0.98 1.11 1.01 1.10 1.39 11.90
f1 (ppm)

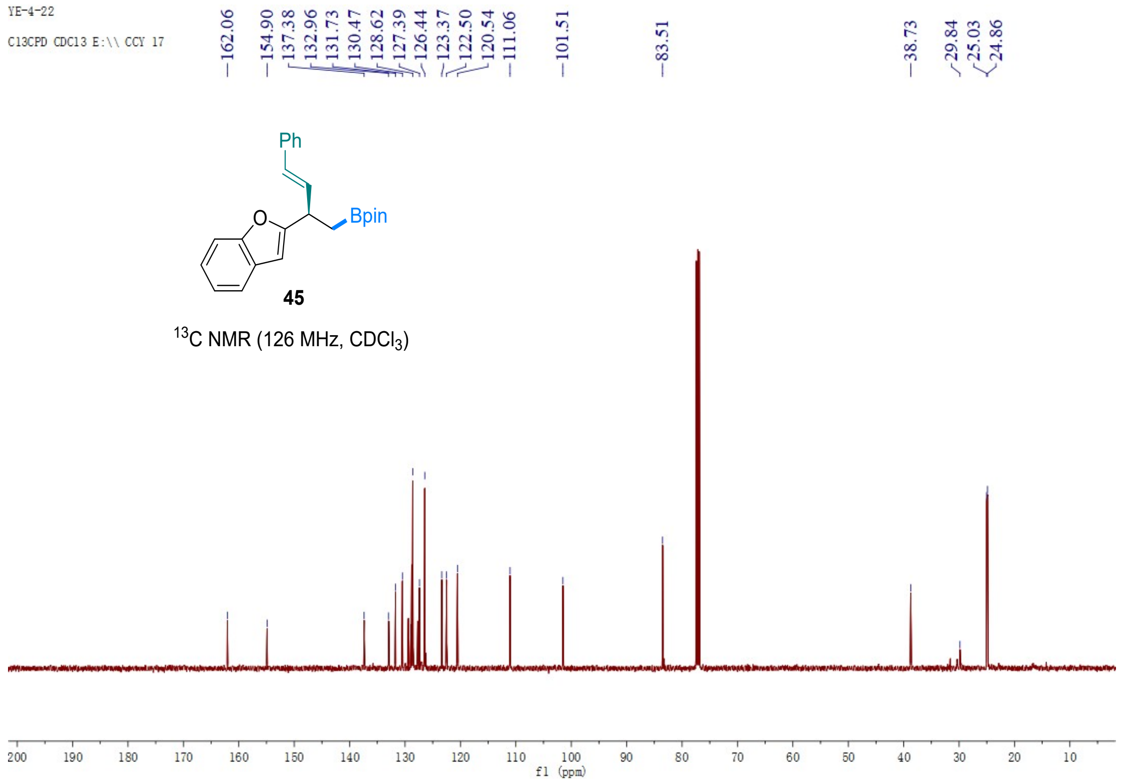
YE-4-22
C13CPD CDCl3 E:\\ CCY 17
162.06 154.90 137.38 132.96 131.73 130.47 128.62 127.39 126.44 123.37 122.50 120.54 111.06 101.51 83.51 38.73 29.84 25.03 24.86
Ph
O
Bpin
45
^{13}C NMR (126 MHz, $CDCl_3$)
f1 (ppm)

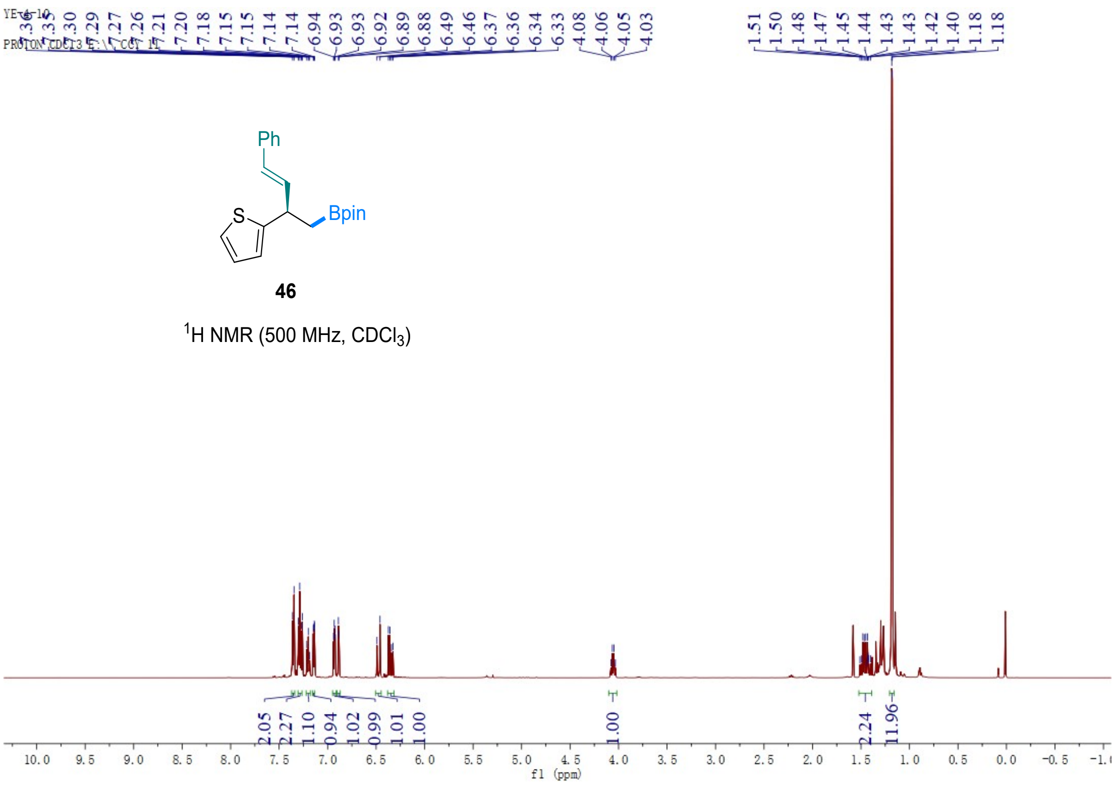

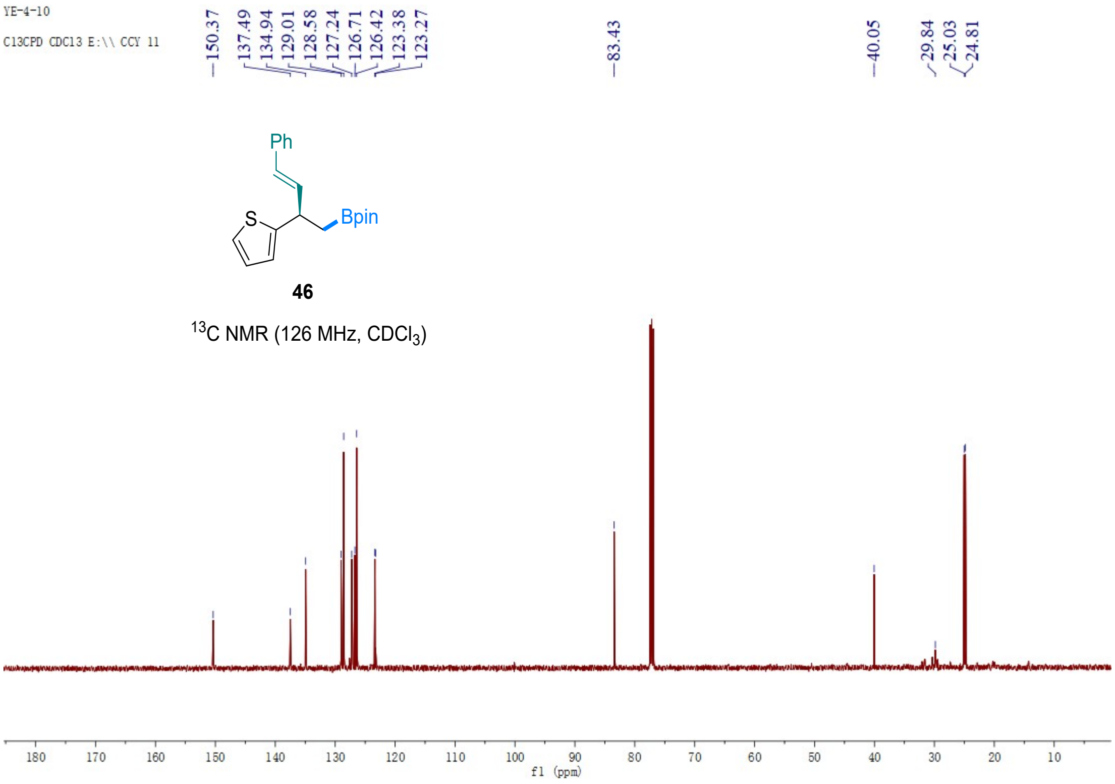

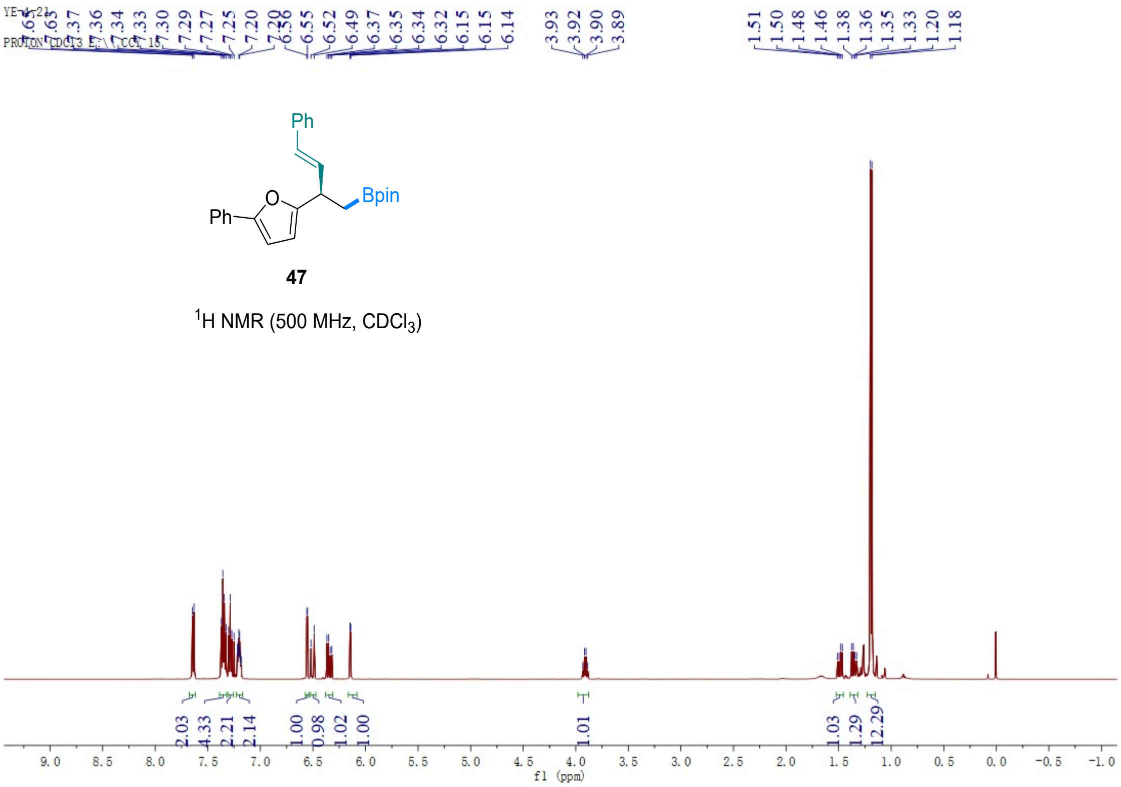
Ph
Bpin
Ph
O
47
¹H NMR (500 MHz, CDCl₃)
fl (ppm)

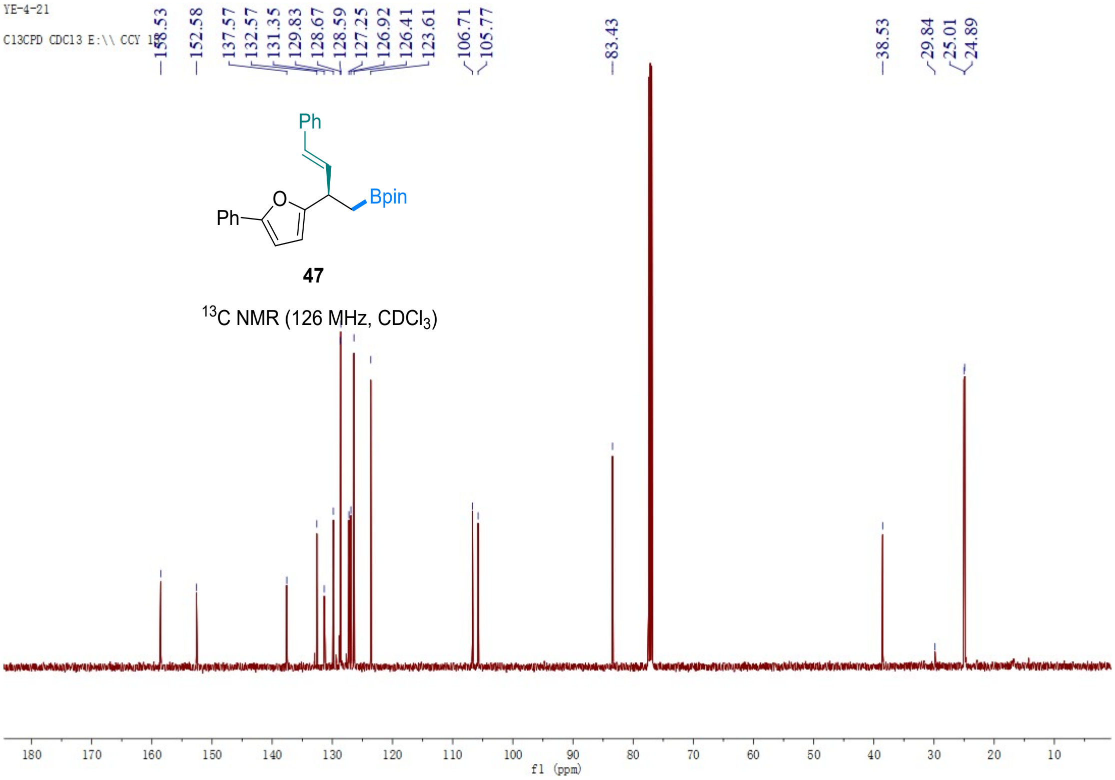
Ph
Bpin
Ph
O
47
¹³C NMR (126 MHz, CDCl₃)
fl (ppm)

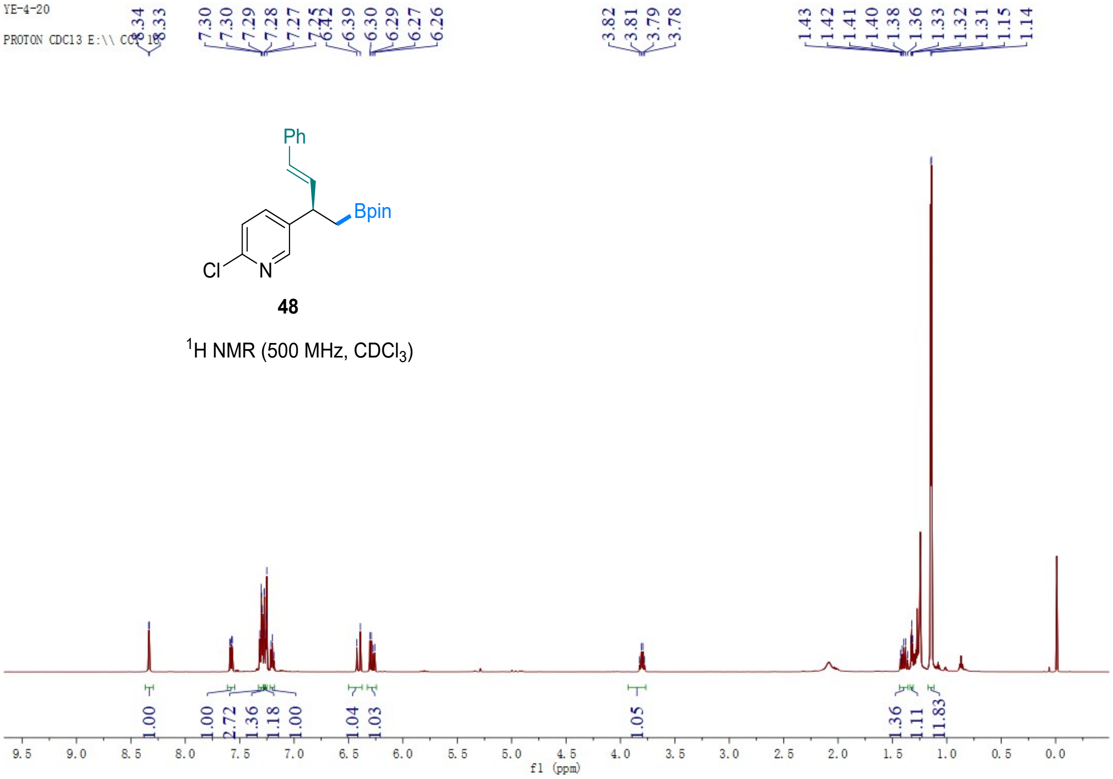
YE-4-20
PROTON CDCl3 E:\\ CCY 16
8.34
8.33
7.30
7.30
7.29
7.28
7.27
7.25
6.42
6.39
6.30
6.29
6.27
6.26
3.82
3.81
3.79
3.78
1.43
1.42
1.41
1.40
1.38
1.36
1.33
1.32
1.31
1.15
1.14
Ph
Bpin
Cl
N
48
^{1}H NMR (500 MHz, CDCl$_3$)
1.00
1.00
2.72
1.36
1.18
1.00
1.04
1.03
1.05
1.36
1.11
11.83
f1 (ppm)

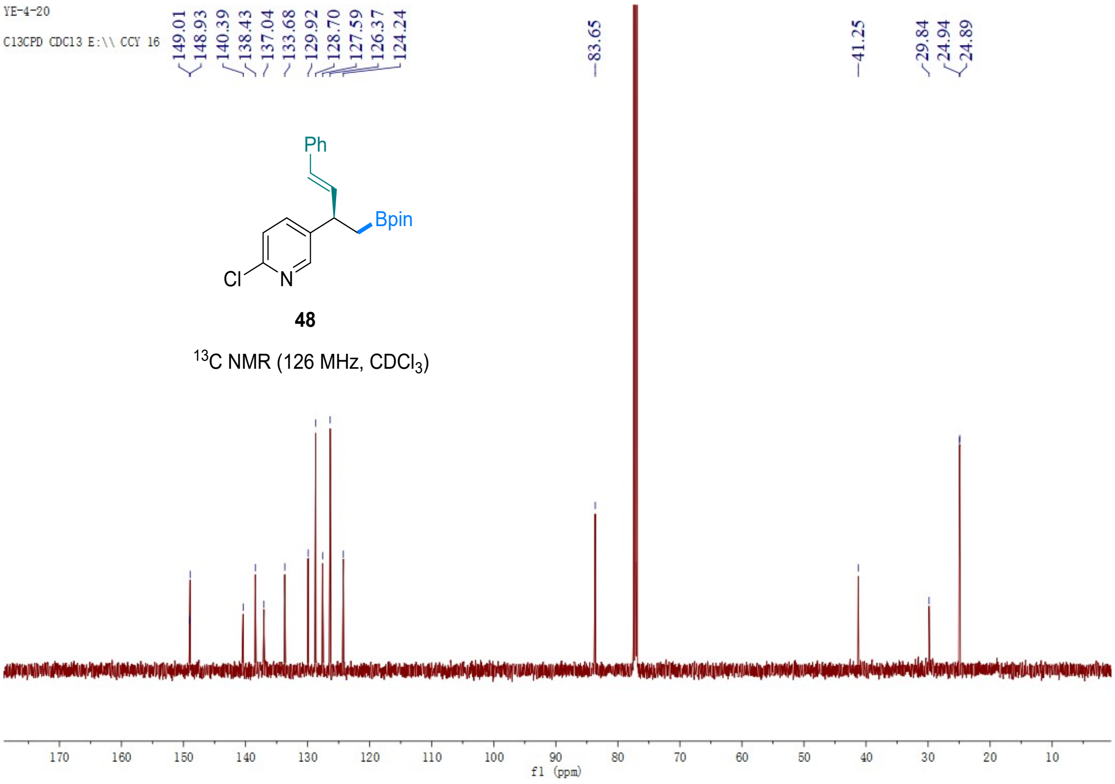
YE-4-20
C13CPD CDCl3 E:\\ CCY 16
149.01
148.93
140.39
138.43
137.04
133.68
129.92
128.70
127.59
126.37
124.24
83.65
41.25
29.84
24.94
24.89
Ph
Bpin
Cl
N
48
^{13}C NMR (126 MHz, CDCl$_3$)
f1 (ppm)

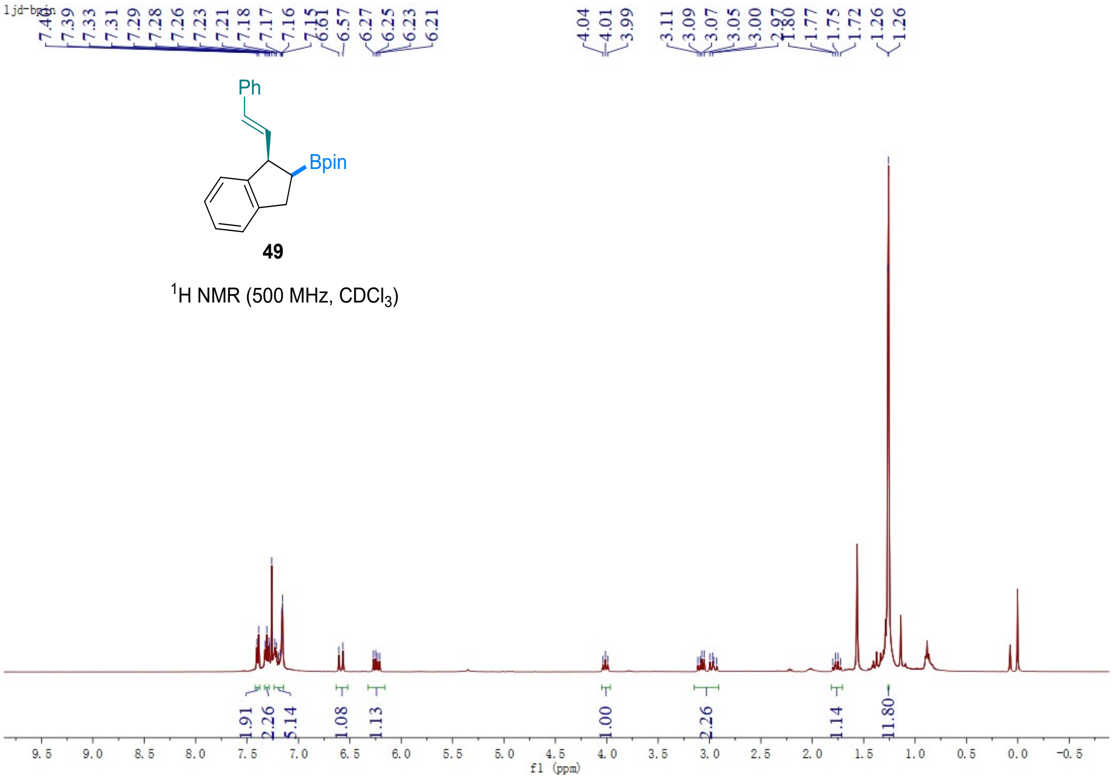
1jd-bpin
7.40 7.39 7.33 7.31 7.29 7.28 7.26 7.23 7.21 7.18 7.17 7.16 7.15 6.61 6.57 6.27 6.25 6.23 6.21
4.04 4.01 3.99
3.11 3.09 3.07 3.05 3.00 2.97 1.80 1.77 1.75 1.72 1.26 1.26
Ph
Bpin
49
^{1}H NMR (500 MHz, CDCl$_3$)
1.91 2.26 5.14 1.08 1.13 1.00 2.26 1.14 11.80
9.5 9.0 8.5 8.0 7.5 7.0 6.5 6.0 5.5 5.0 4.5 4.0 3.5 3.0 2.5 2.0 1.5 1.0 0.5 0.0 -0.5
f1 (ppm)

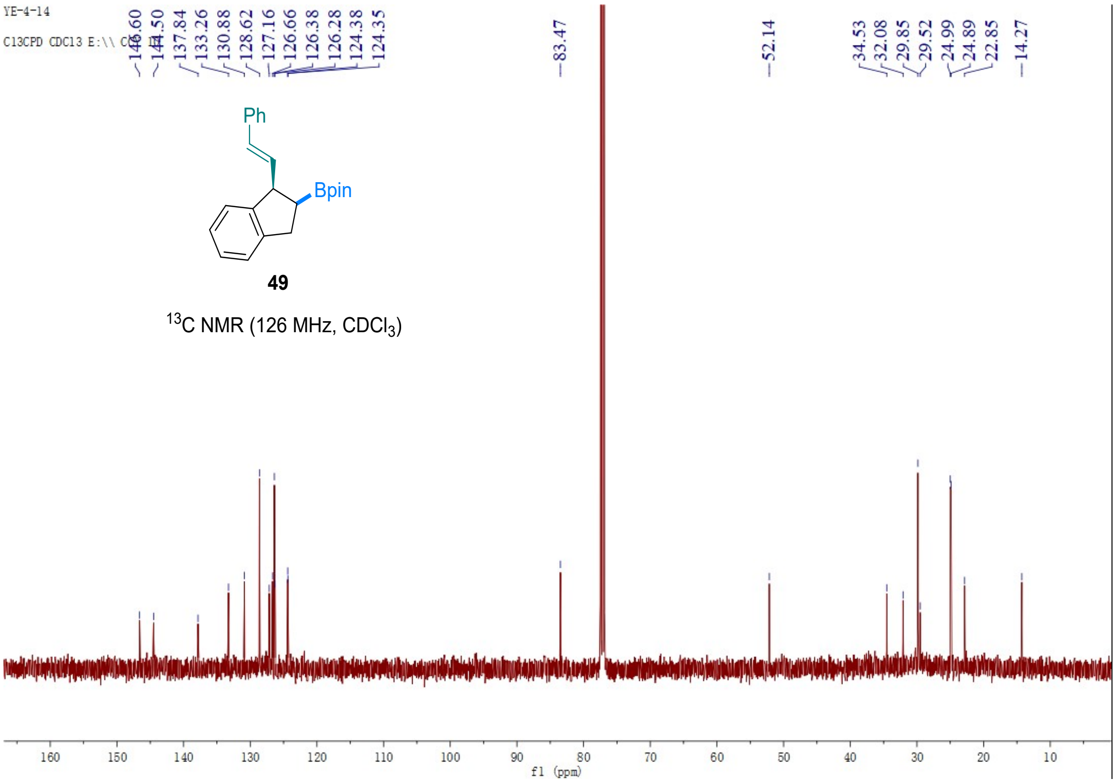
YE-4-14
C13CPD CDCl3 E:\\ CDCl3
146.60 144.50 137.84 133.26 130.88 128.62 127.16 126.66 126.38 126.28 124.38 124.35
83.47
52.14
34.53 32.08 29.85 29.52 24.99 24.89 22.85 14.27
Ph
Bpin
49
^{13}C NMR (126 MHz, CDCl$_3$)
160 150 140 130 120 110 100 90 80 70 60 50 40 30 20 10
f1 (ppm)

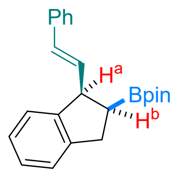

49

COSY NMR (500 MHz, $CDCl_3$)

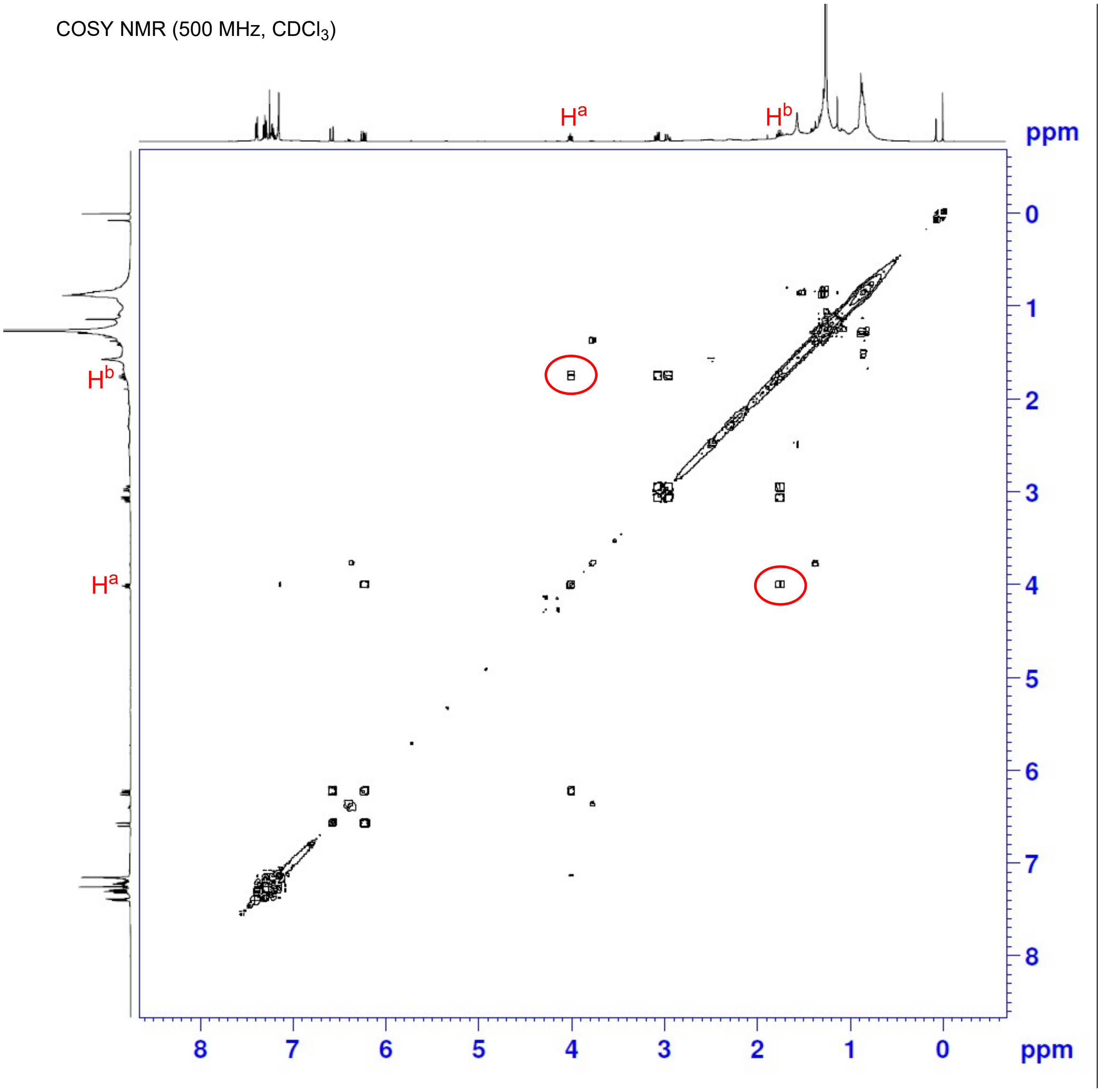

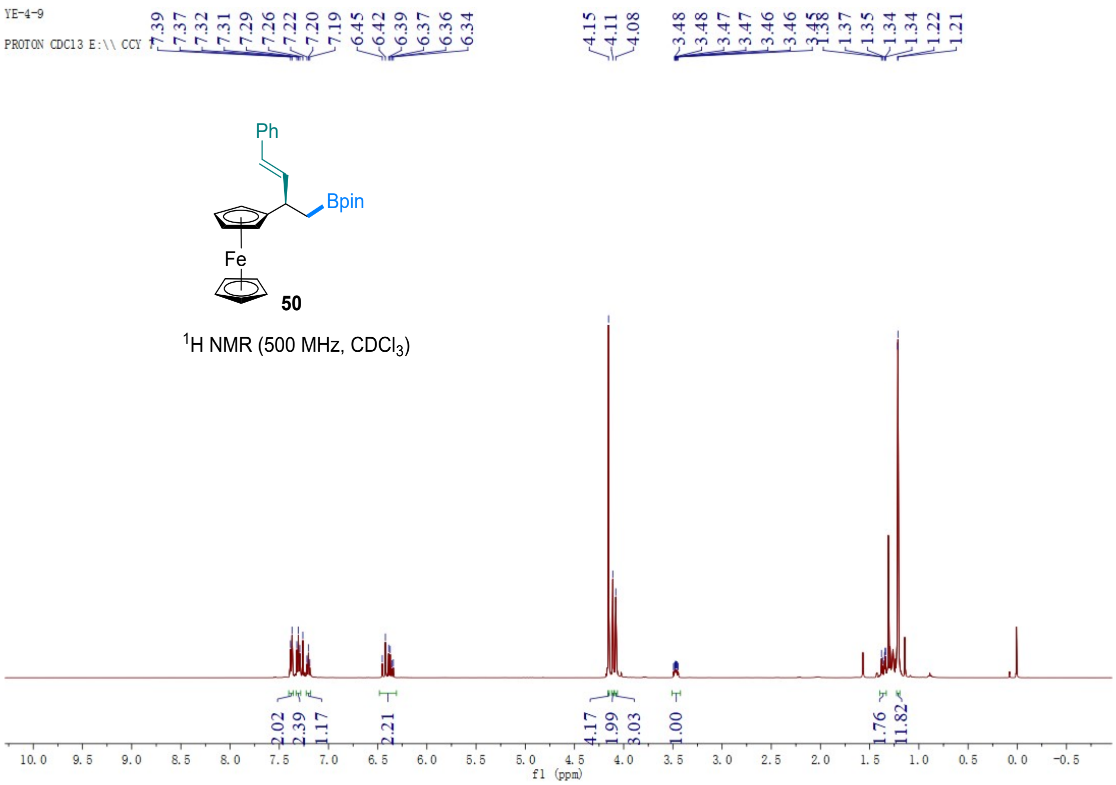

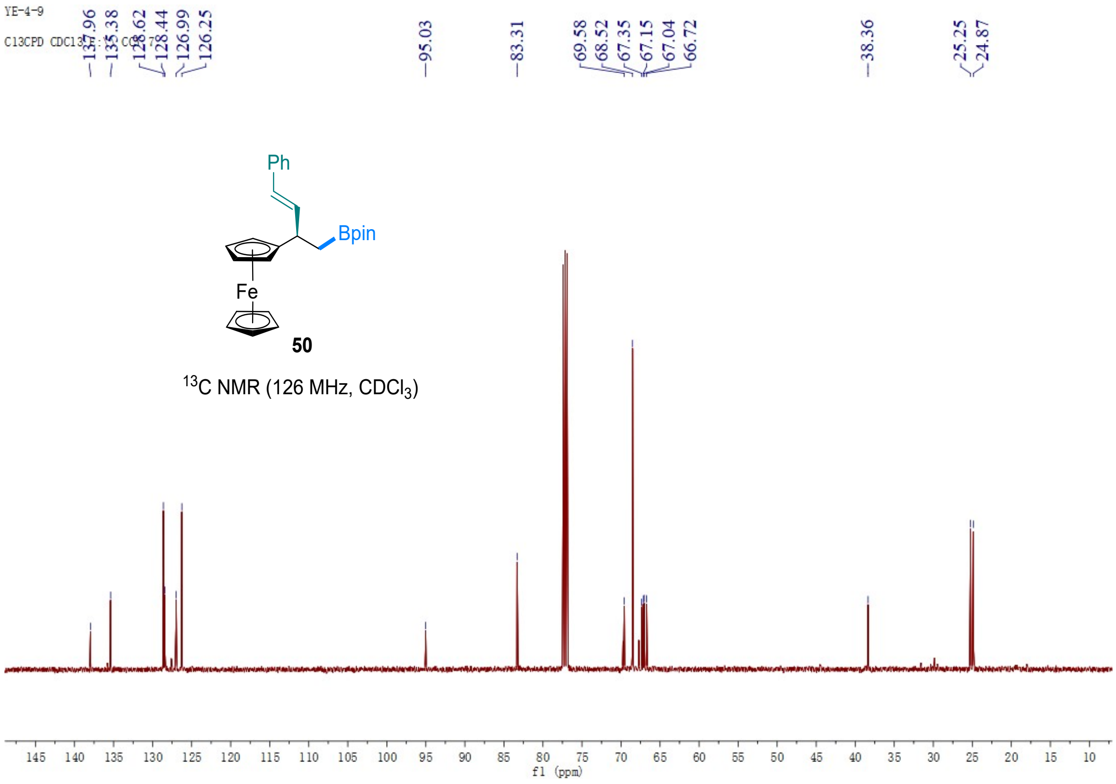

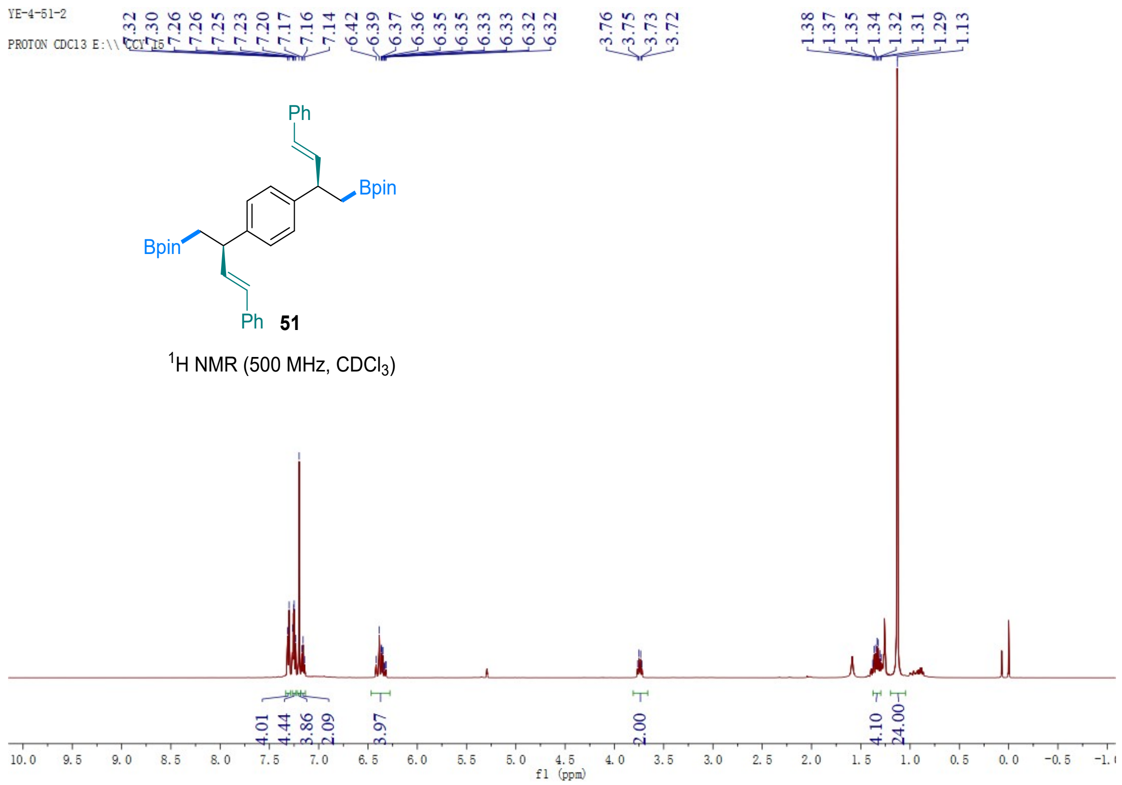
YE-4-51-2
PROTON CDCl3
7.32 7.30 7.26 7.26 7.25 7.23 7.20 7.17 7.16 7.14 6.42 6.39 6.37 6.36 6.35 6.35 6.33 6.33 6.32 6.32
3.76 3.75 3.73 3.72
1.38 1.37 1.35 1.34 1.32 1.31 1.29 1.13
Ph
Bpin
Bpin
Ph
51
1H NMR (500 MHz, CDCl3)
4.01 4.44 3.86 2.09 3.97 2.00 4.10 24.00
f1 (ppm)

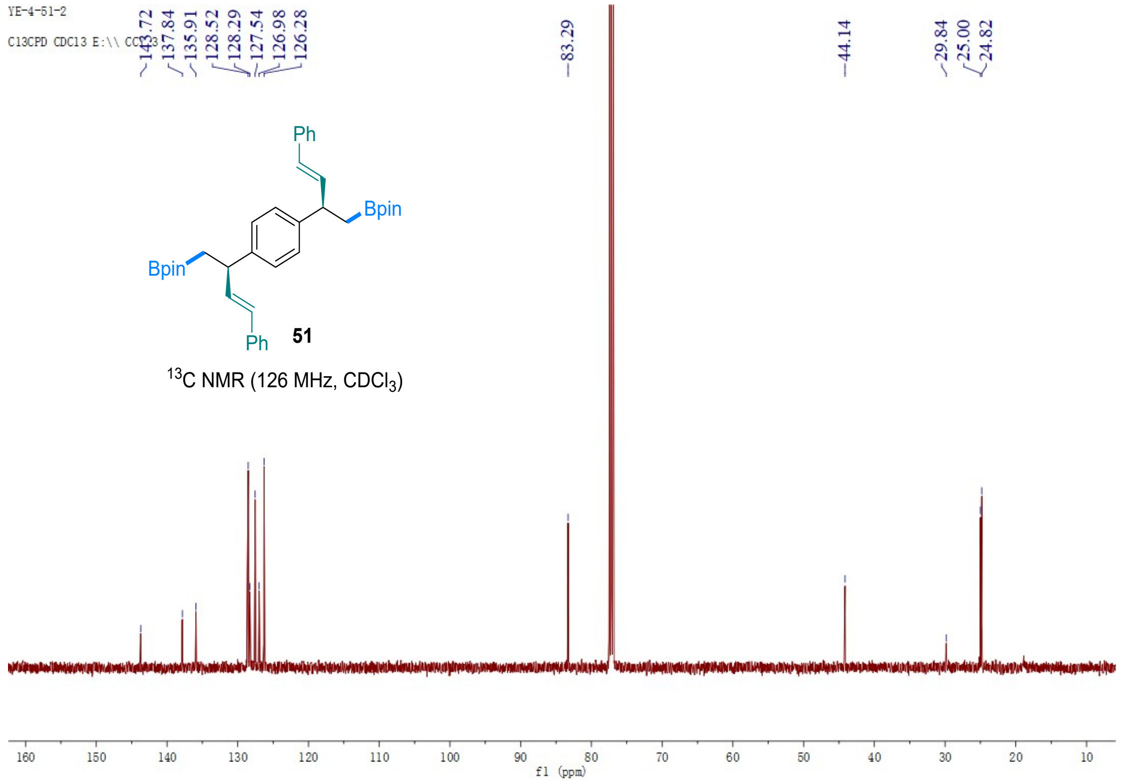
YE-4-51-2
C13CPD CDCl3
143.72 137.84 135.91 128.52 128.29 127.54 126.98 126.28
83.29
44.14
29.84 25.00 24.82
Ph
Bpin
Bpin
Ph
51
13C NMR (126 MHz, CDCl3)
f1 (ppm)

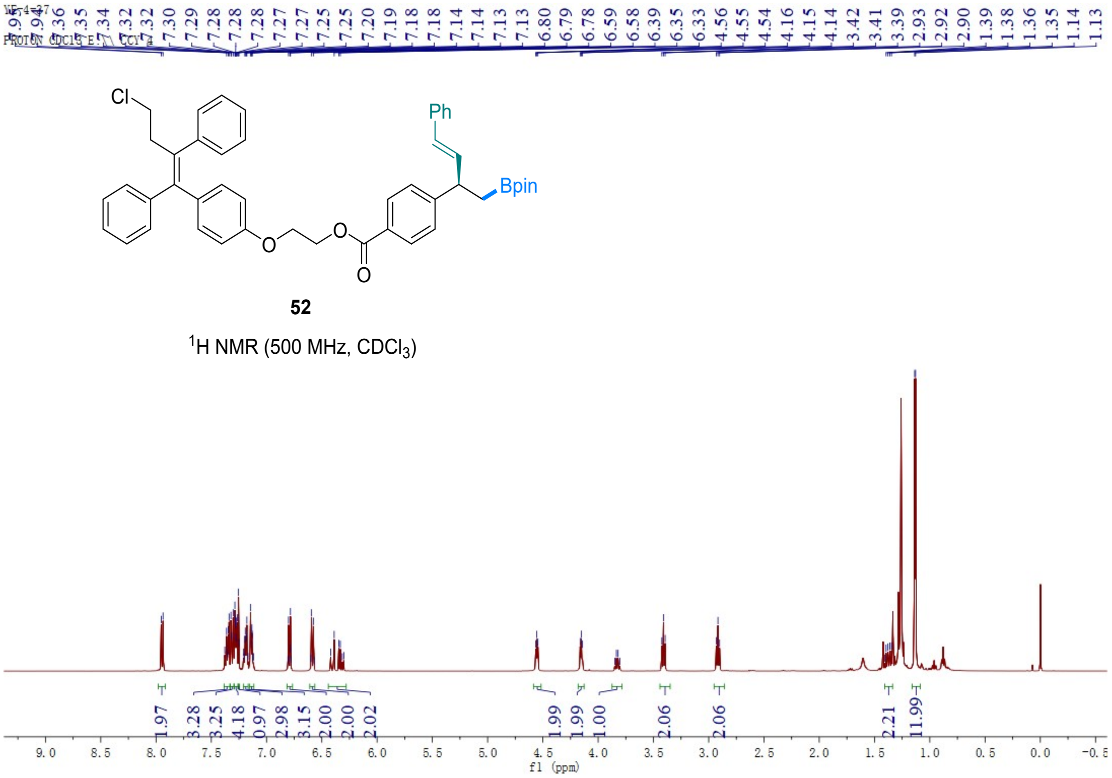

52

^{1}H NMR (500 MHz, $CDCl_3$)

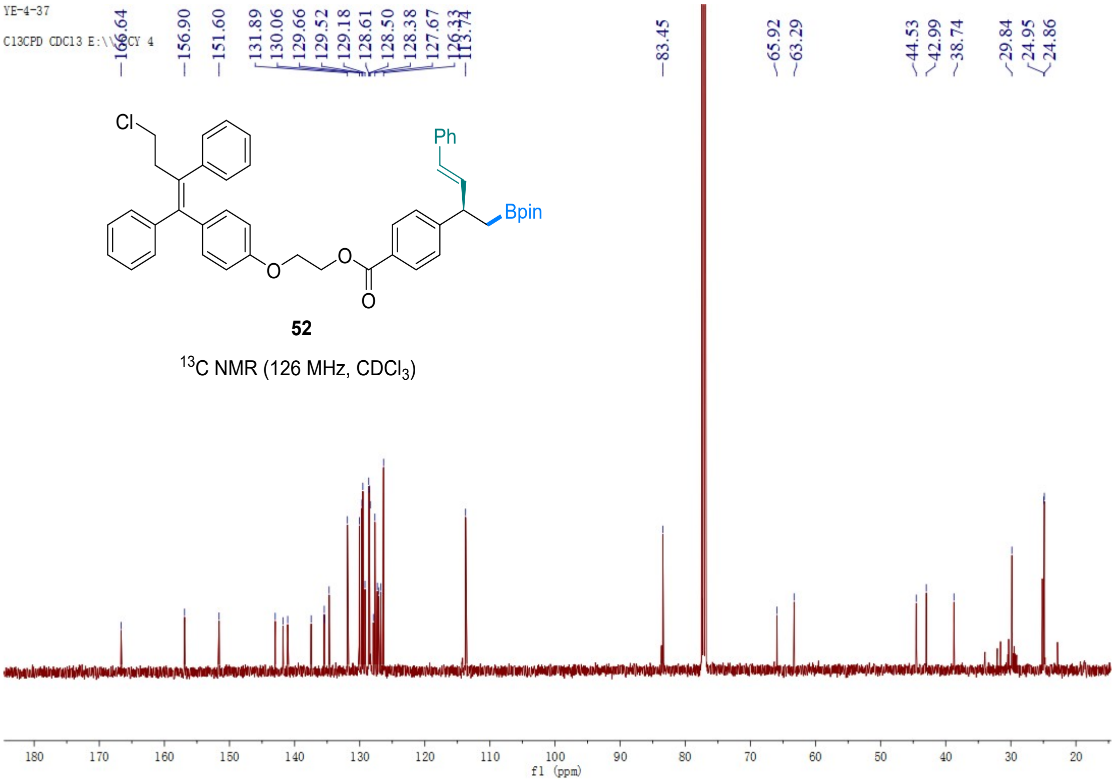

52

^{13}C NMR (126 MHz, $CDCl_3$)

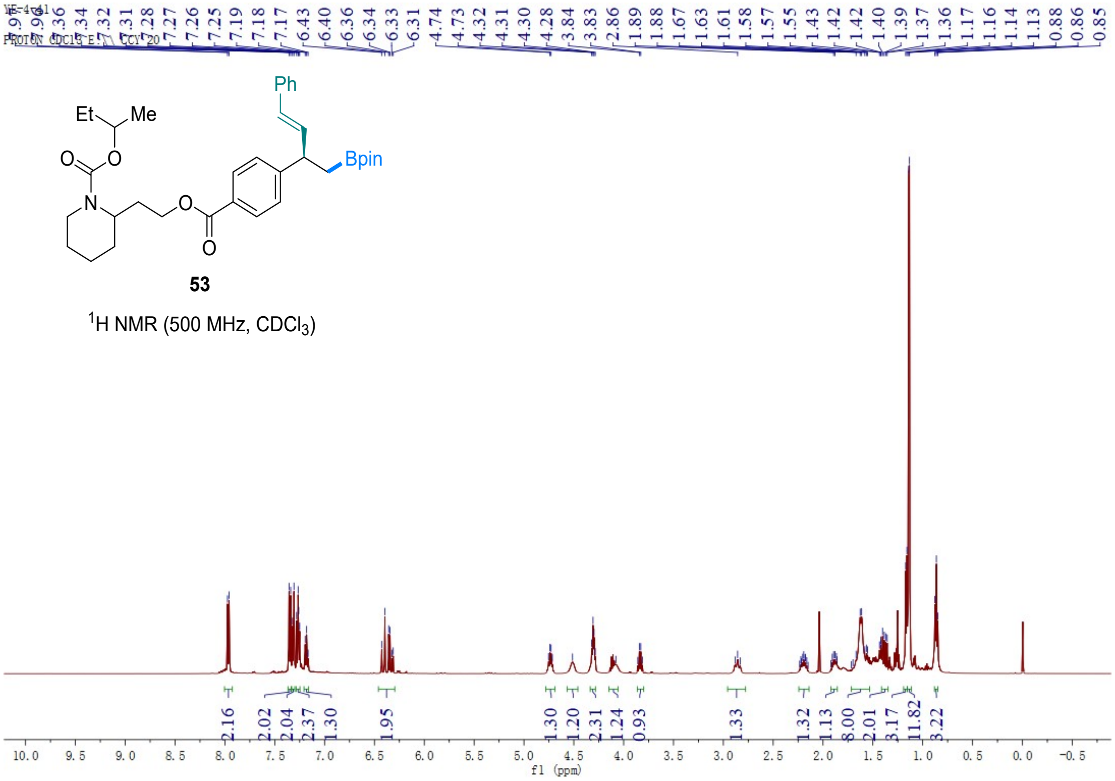

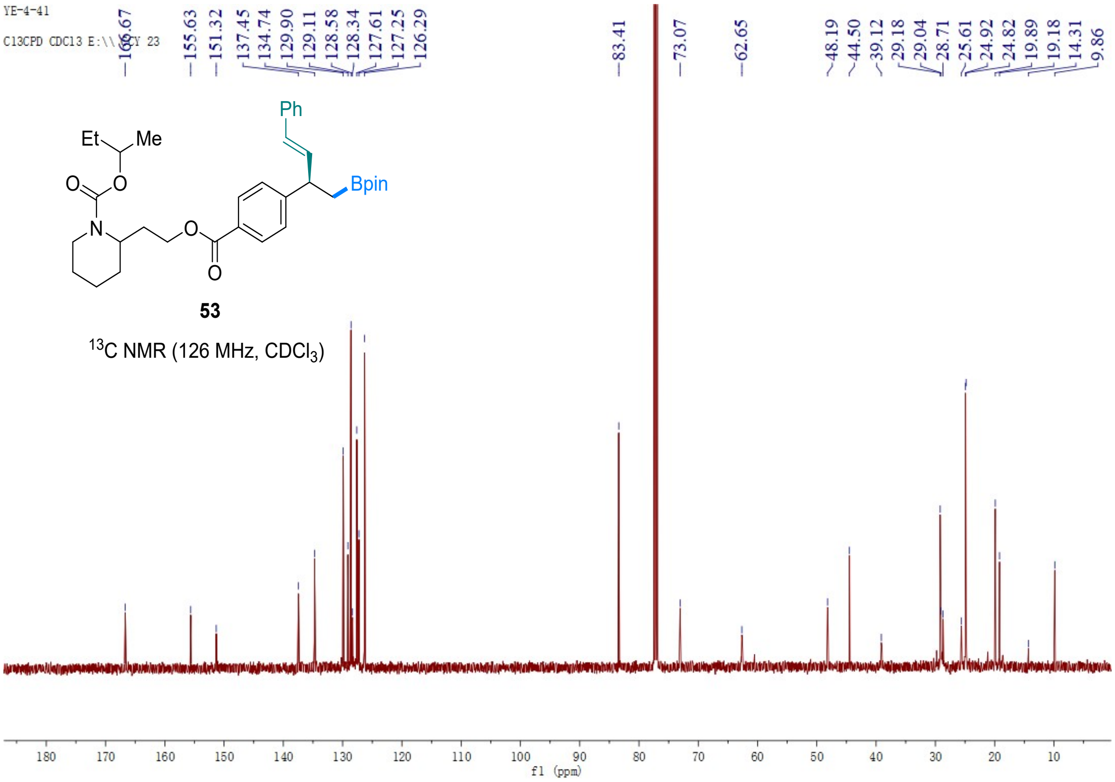

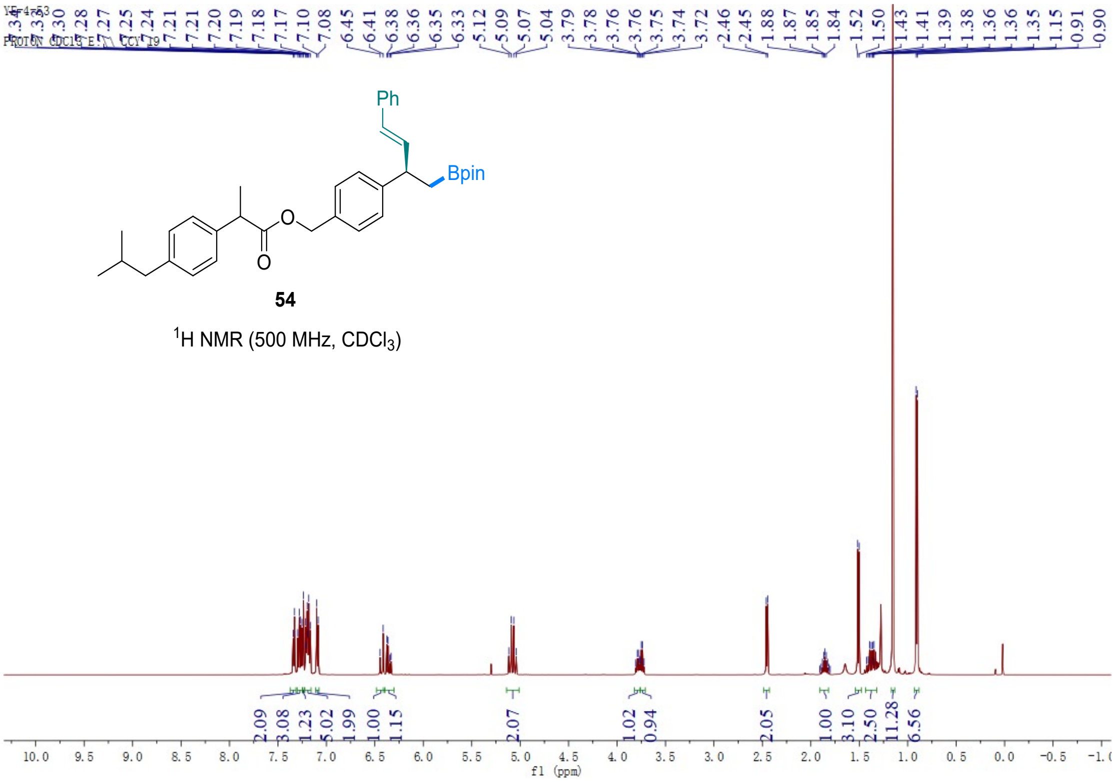
54
¹H NMR (500 MHz, CDCl₃)
f1 (ppm)

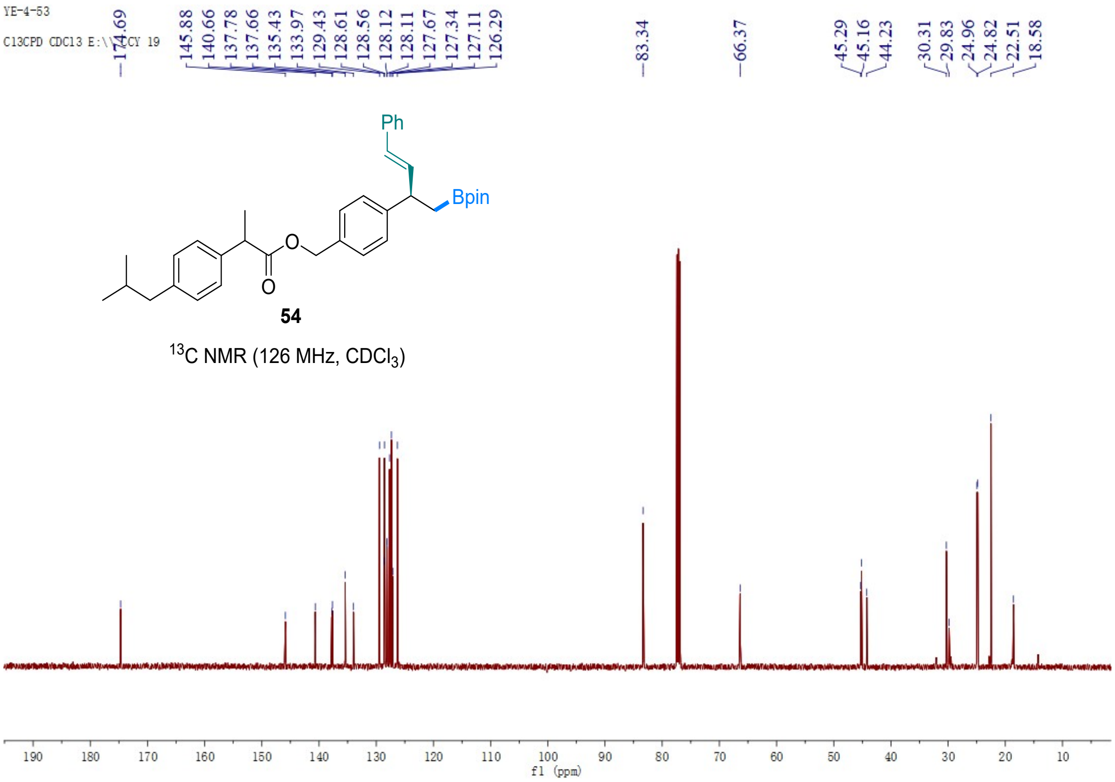
54
¹³C NMR (126 MHz, CDCl₃)
f1 (ppm)

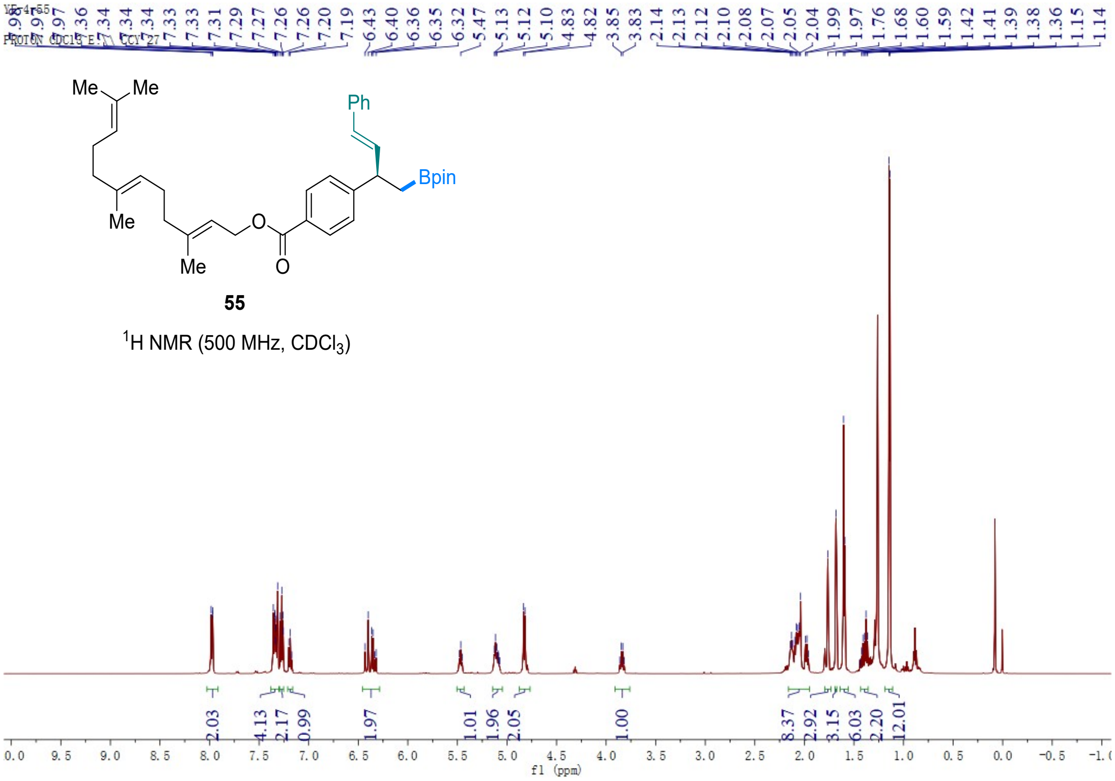
YE-4-55
PROTON CDCl3 E:\\ CCY 27
Me
Me
Ph
Bpin
Me
O
O
Me
55
1H NMR (500 MHz, CDCl3)
f1 (ppm)

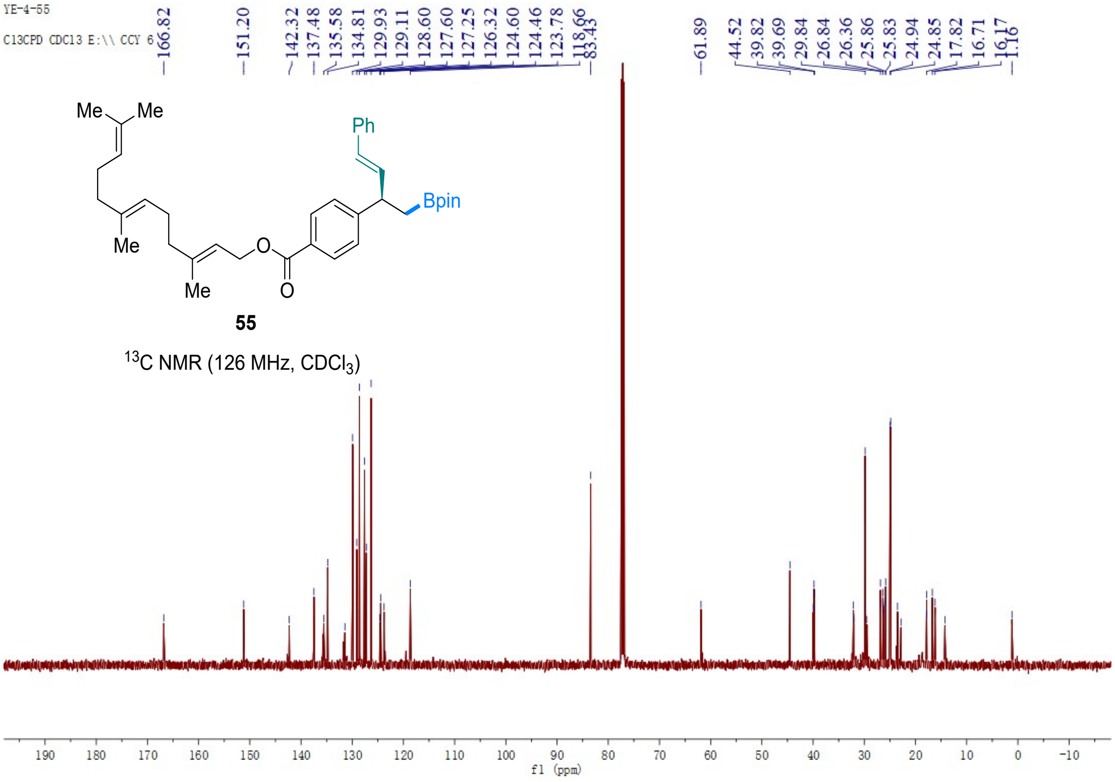
YE-4-55
C13CPD CDCl3 E:\\ CCY 6
Me
Me
Ph
Bpin
Me
O
O
Me
55
13C NMR (126 MHz, CDCl3)
f1 (ppm)

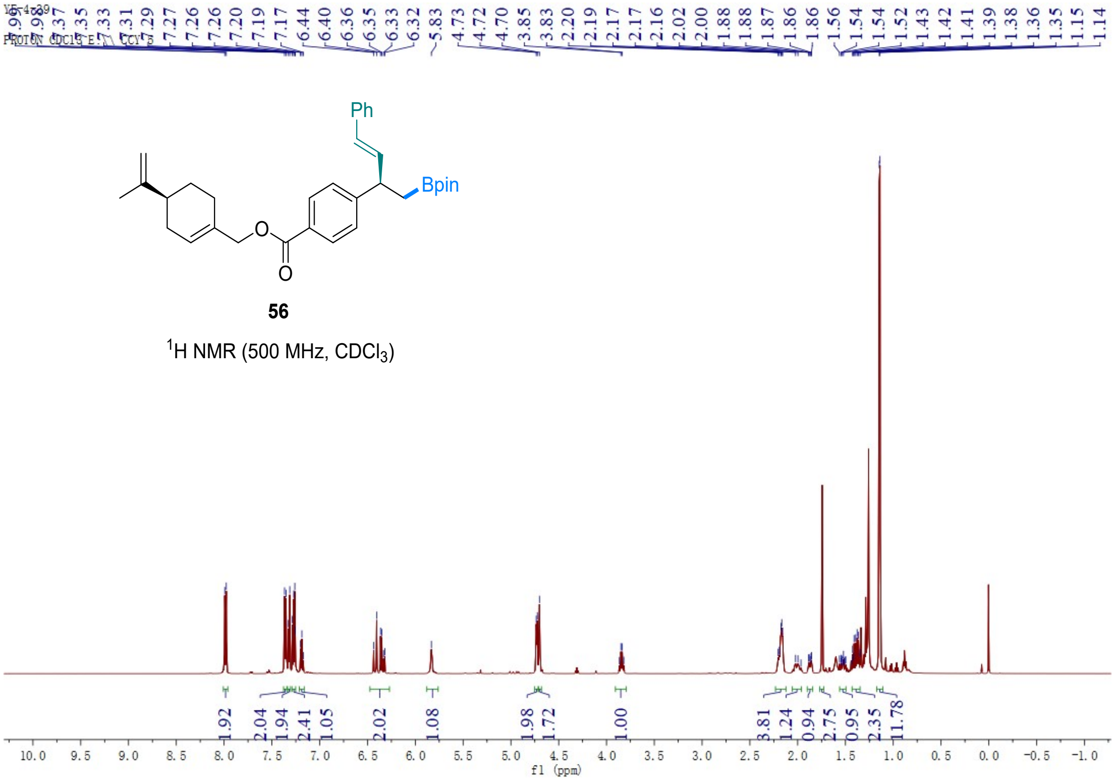

56

^{1}H NMR (500 MHz, $CDCl_3$)

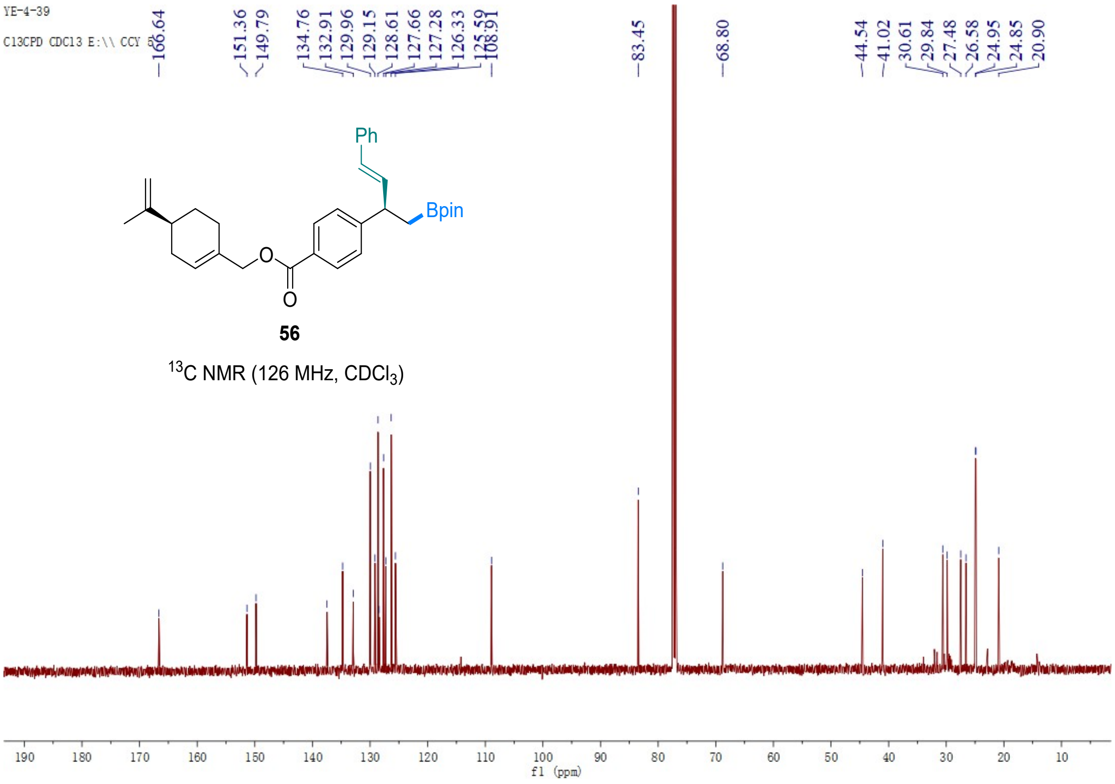

56

^{13}C NMR (126 MHz, $CDCl_3$)

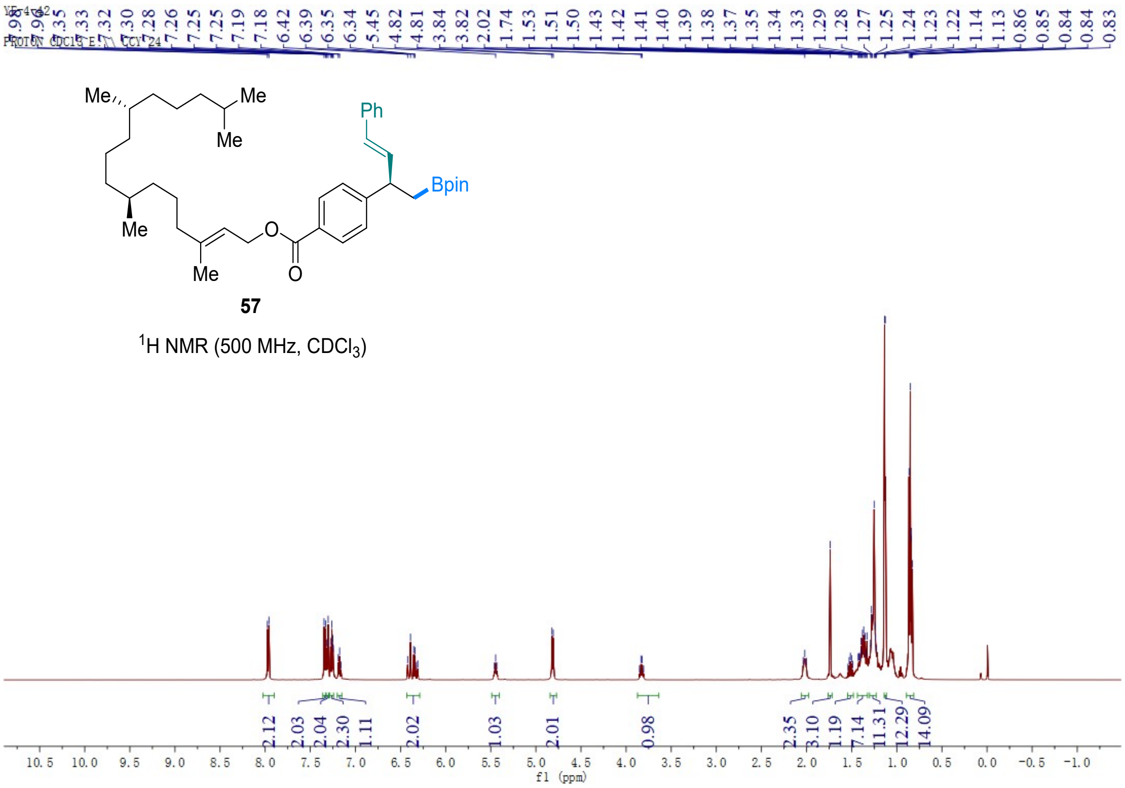

57

^1H NMR (500 MHz, $CDCl_3$)

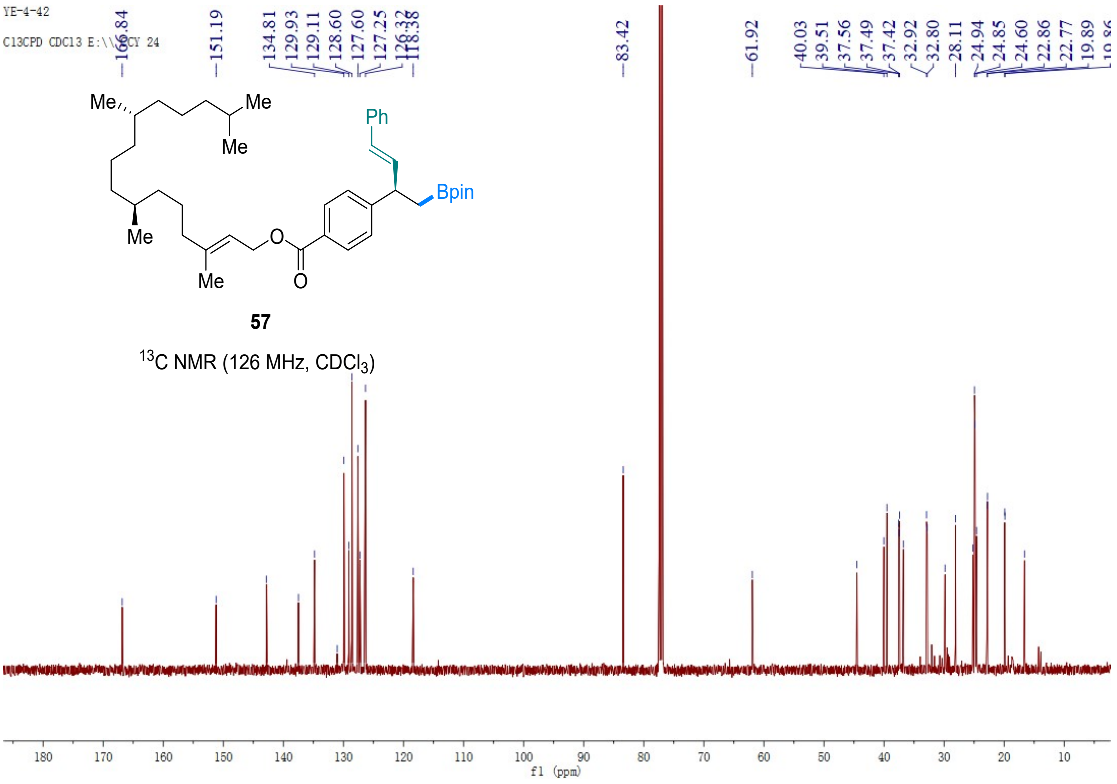

57

^{13}C NMR (126 MHz, $CDCl_3$)

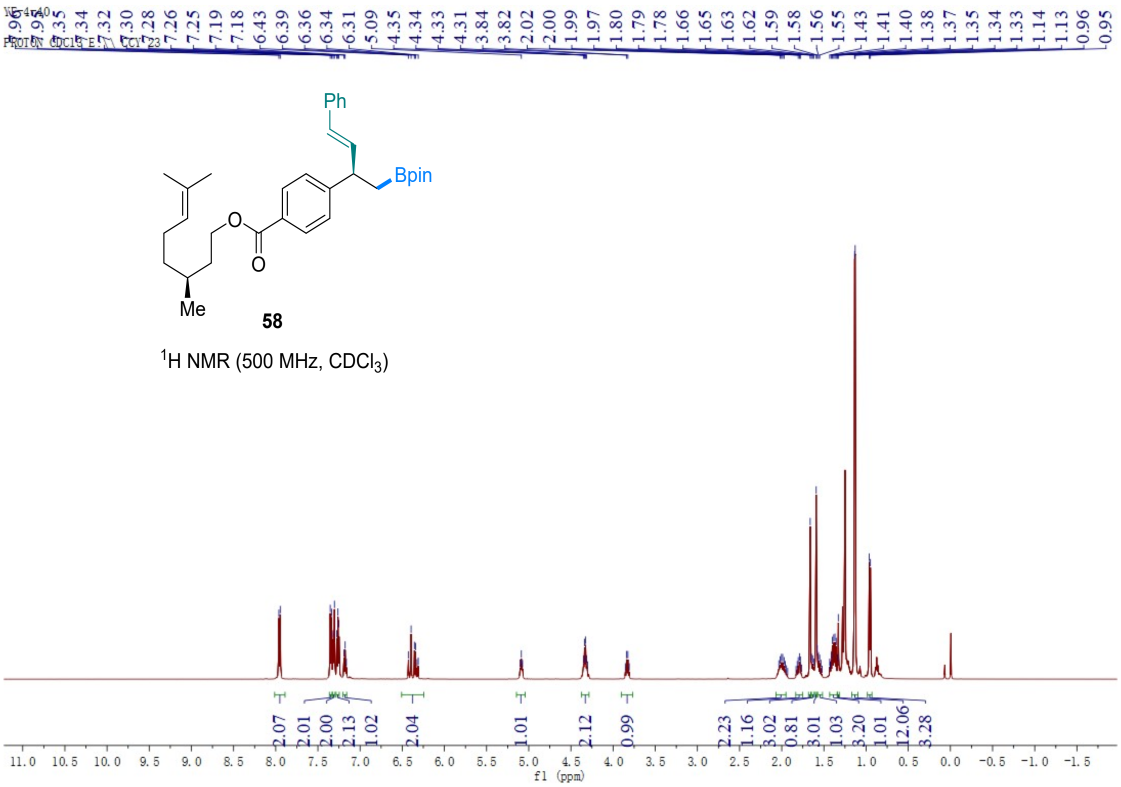
YE-4-40
PROTON CDCl3 E:\\ CCY 23
7.91 7.89 7.35 7.34 7.32 7.30 7.28 7.26 7.25 7.19 7.18 6.43 6.39 6.36 6.34 6.31 5.09 4.35 4.34 4.33 4.31 3.84 3.82 2.02 2.00 1.99 1.97 1.80 1.79 1.78 1.66 1.65 1.63 1.62 1.59 1.58 1.56 1.55 1.43 1.41 1.40 1.38 1.37 1.35 1.34 1.33 1.14 1.13 0.96 0.95
Ph
Bpin
O
O
Me
58
^{1}H NMR (500 MHz, $CDCl_3$)
2.07 2.01 2.00 2.13 1.02 2.04 1.01 2.12 0.99 2.23 1.16 3.02 0.81 3.01 1.03 3.20 1.01 12.06 3.28
f1 (ppm)

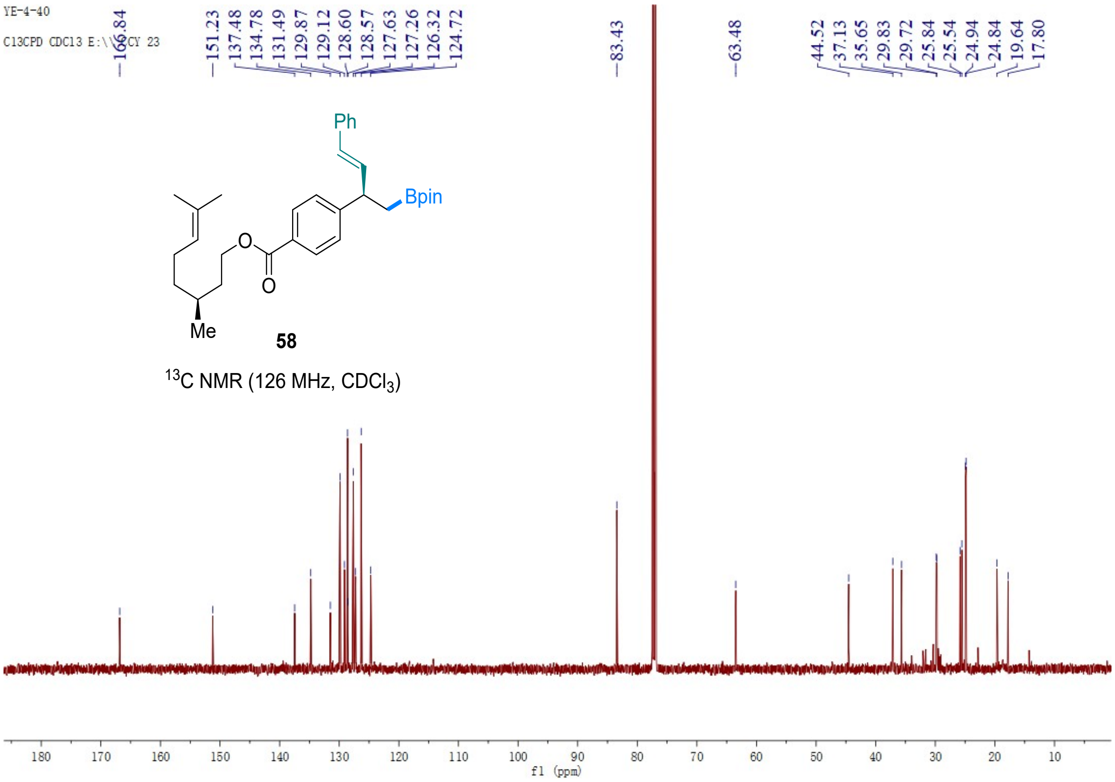
YE-4-40
C13CPD CDCl3 E:\\ CCY 23
166.84
151.23 137.48 134.78 131.49 129.87 129.12 128.60 128.57 127.63 127.26 126.32 124.72
83.43
63.48
44.52 37.13 35.65 29.83 29.72 25.84 25.54 24.94 24.84 19.64 17.80
Ph
Bpin
O
O
Me
58
^{13}C NMR (126 MHz, $CDCl_3$)
f1 (ppm)

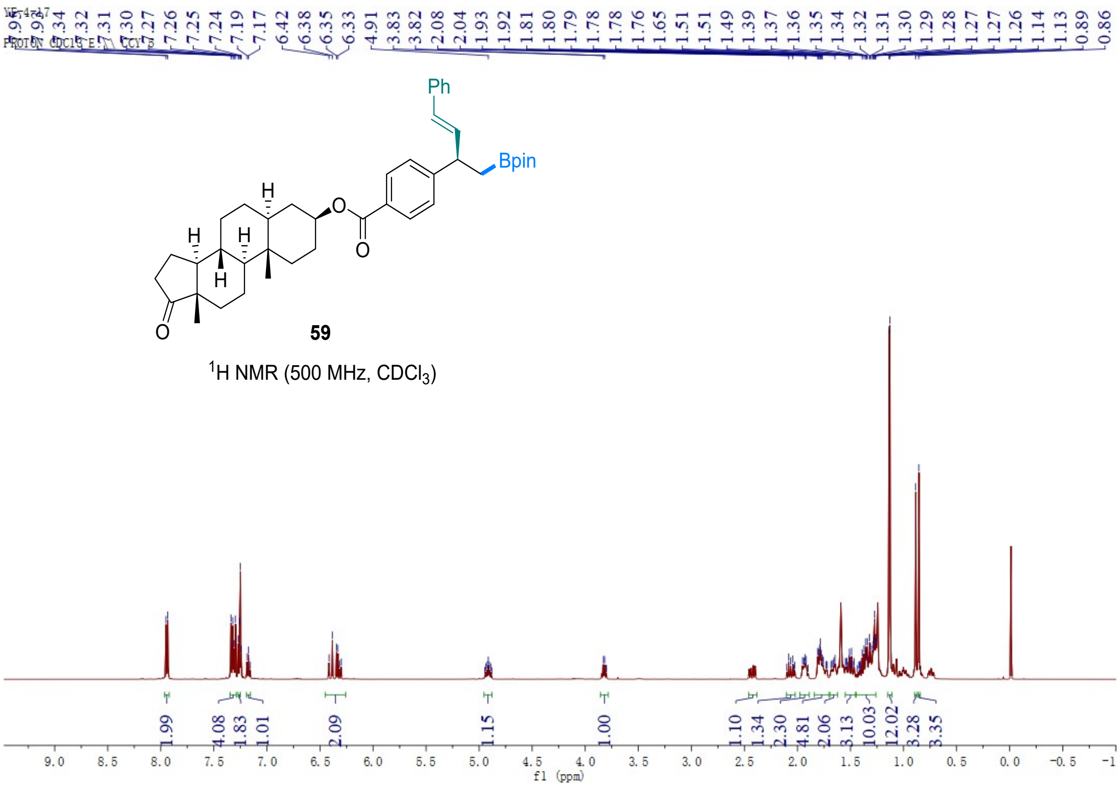

59

^{1}H NMR (500 MHz, $CDCl_3$)

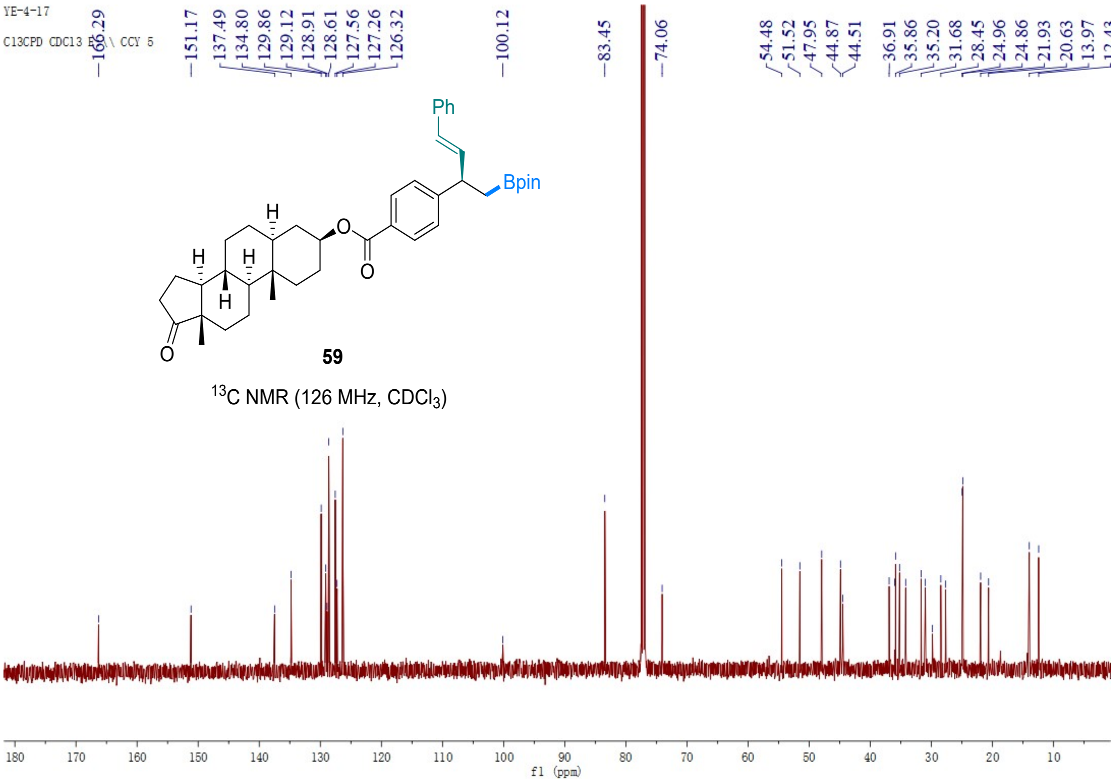

59

^{13}C NMR (126 MHz, $CDCl_3$)

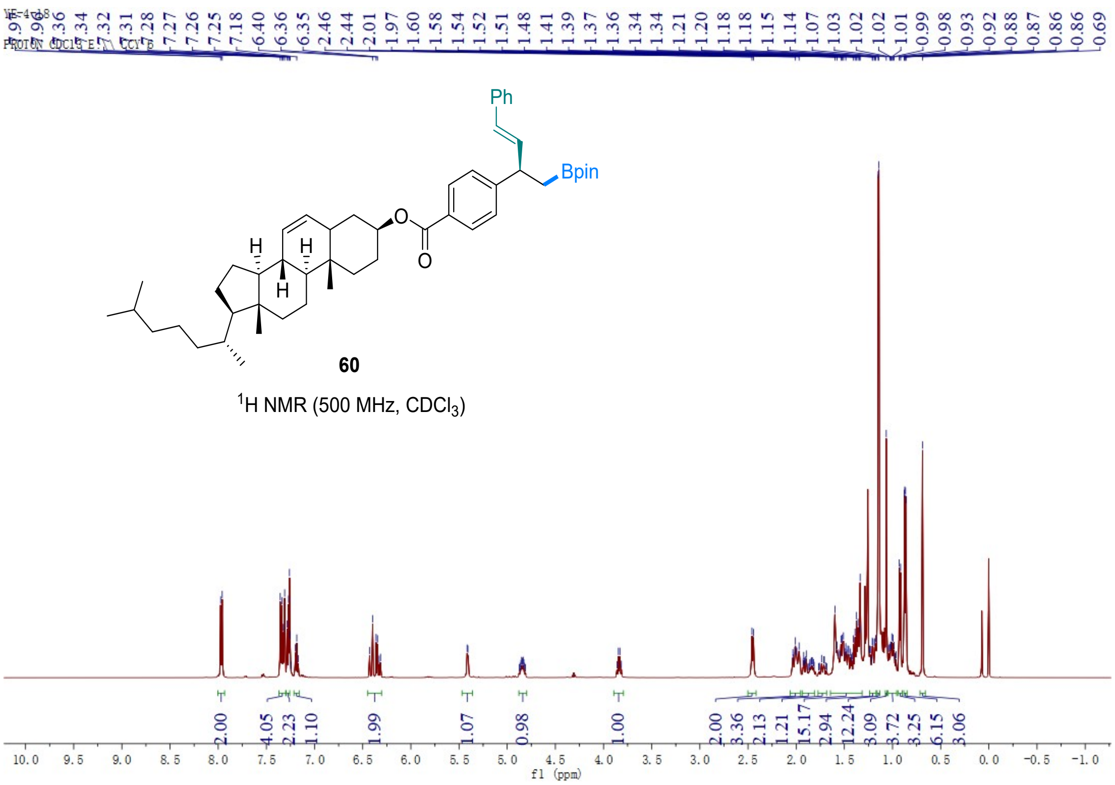

Ph
Bpin
H
H
H
O
O
60
¹H NMR (500 MHz, CDCl₃)
f1 (ppm)

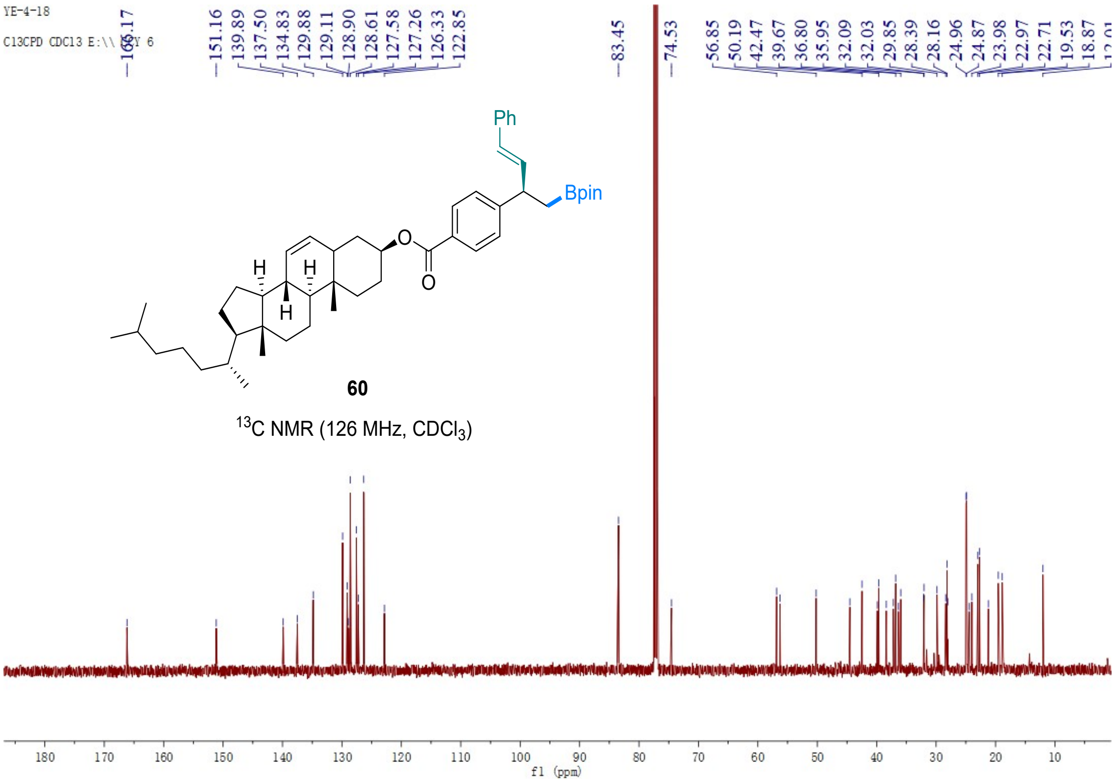

Ph
Bpin
H
H
H
O
O
60
¹³C NMR (126 MHz, CDCl₃)
f1 (ppm)

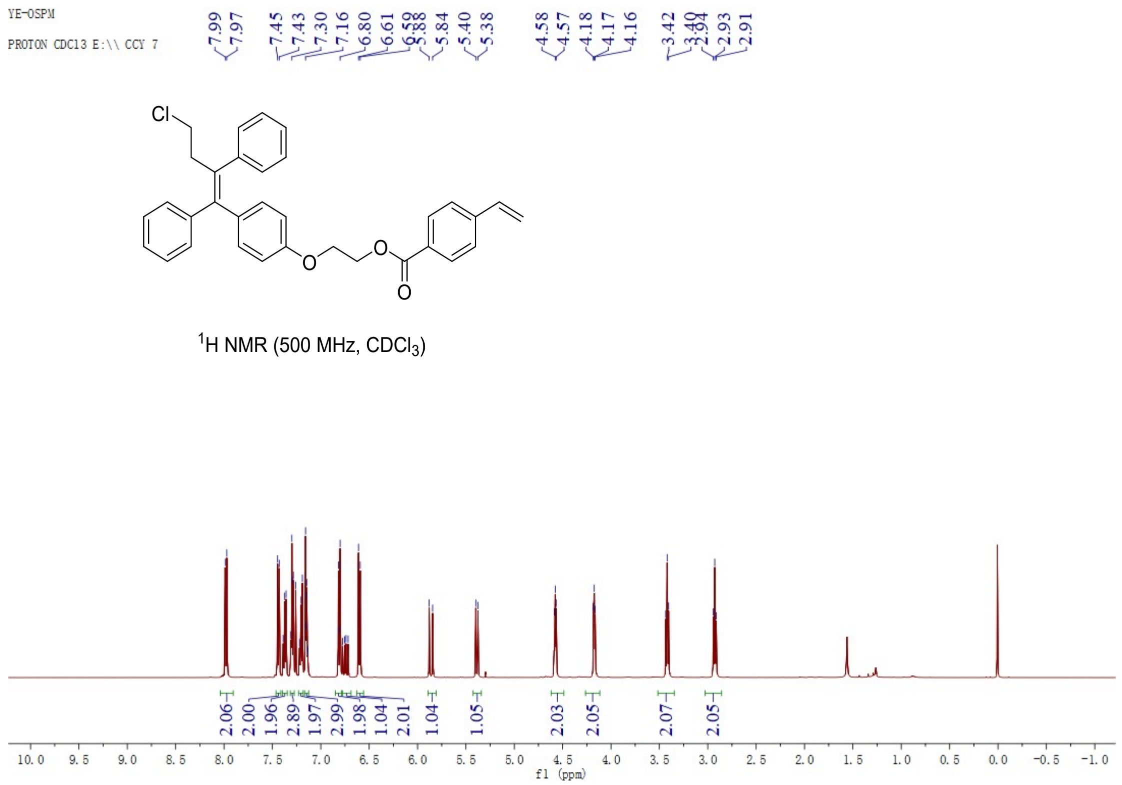

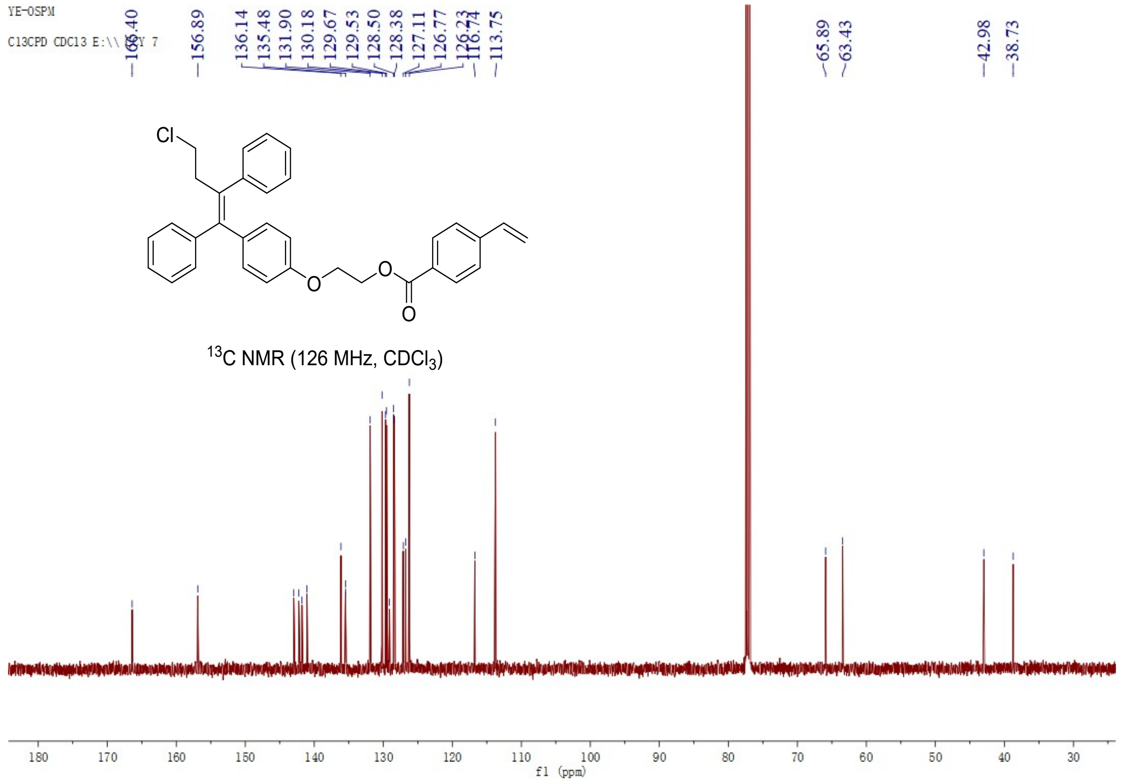

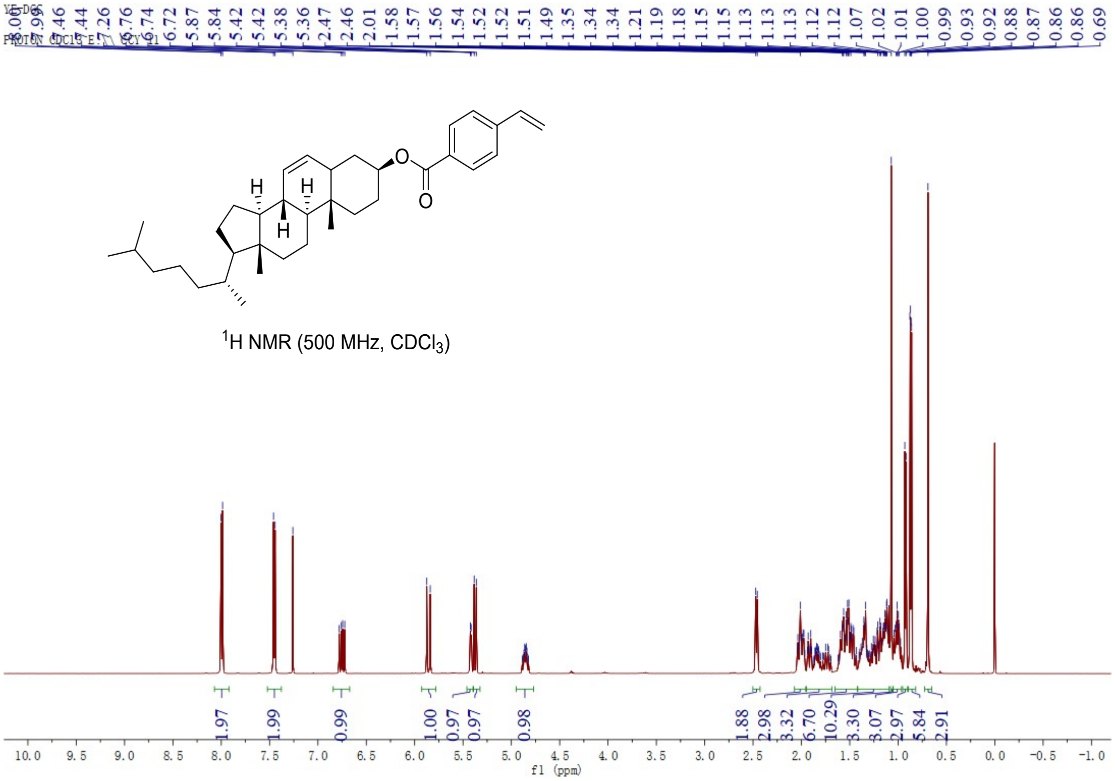

YE-DGC
1H NMR (500 MHz, CDCl3)
f1 (ppm)

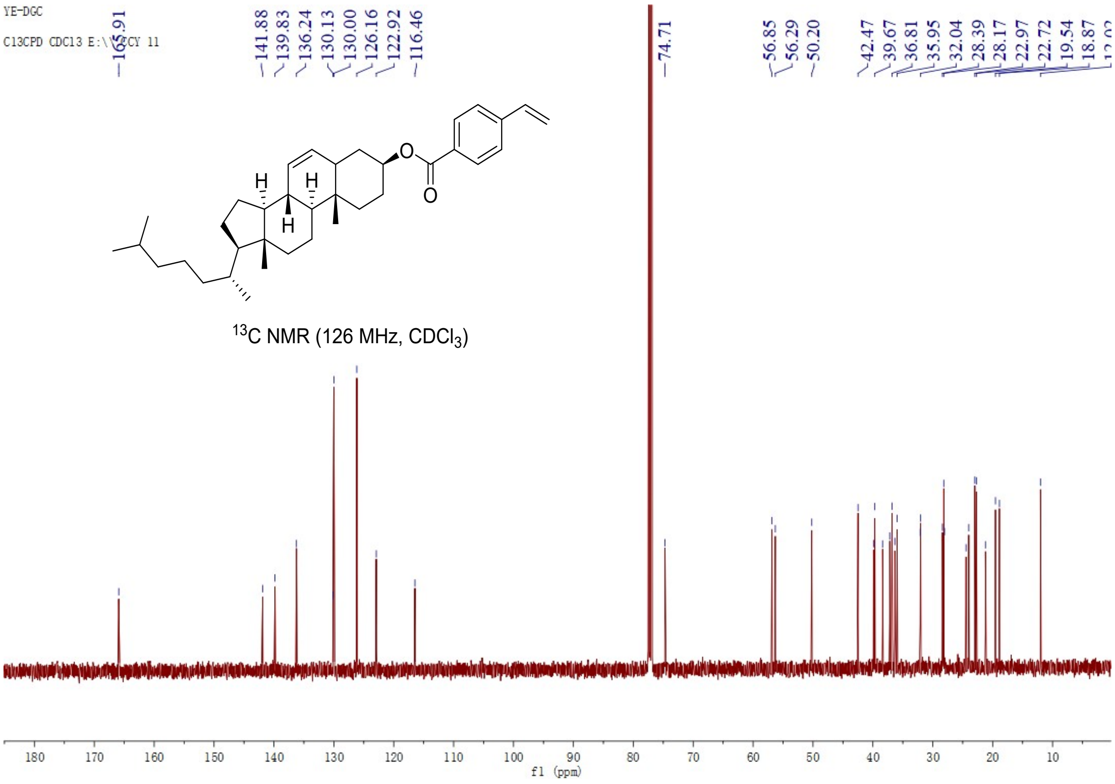

YE-DGC
C13CPD CDCl3
13C NMR (126 MHz, CDCl3)
f1 (ppm)

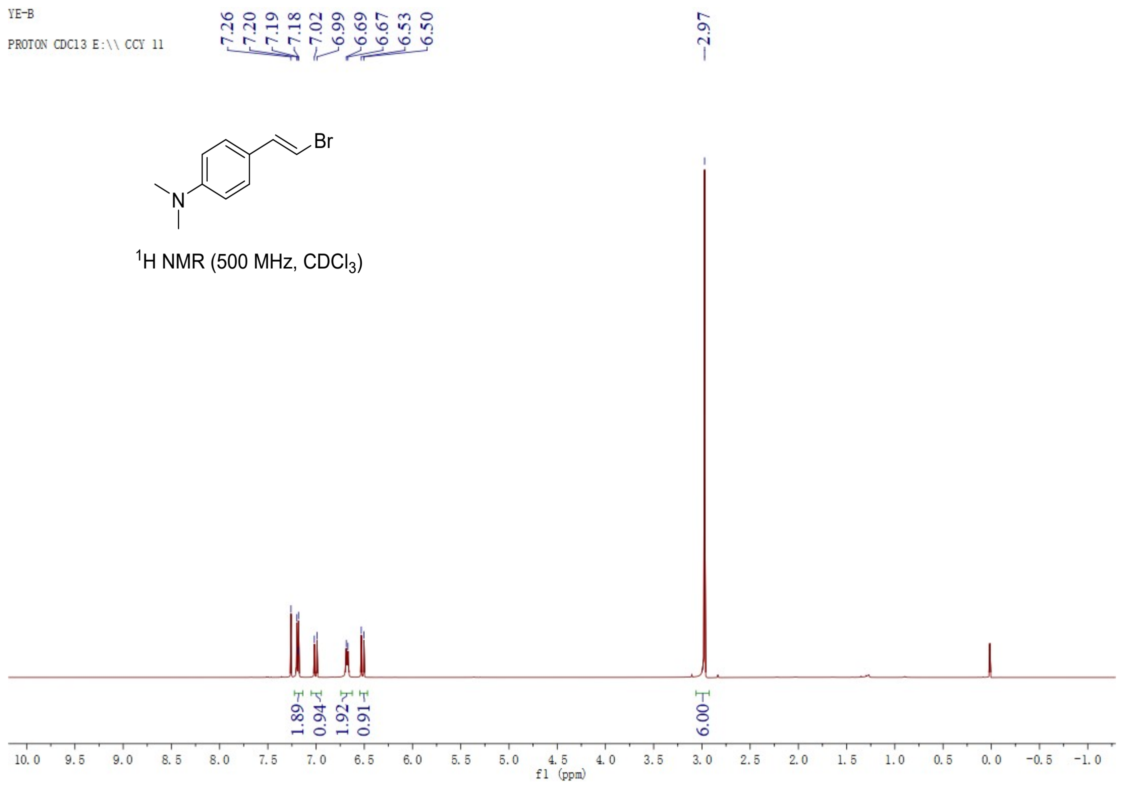
YE-B
PROTON CDCl3 E:\\ CCY 11
7.26
7.20
7.19
7.18
7.02
6.99
6.69
6.67
6.53
6.50
2.97
Br
N
1H NMR (500 MHz, CDCl3)
1.89
0.94
1.92
0.91
6.00
f1 (ppm)

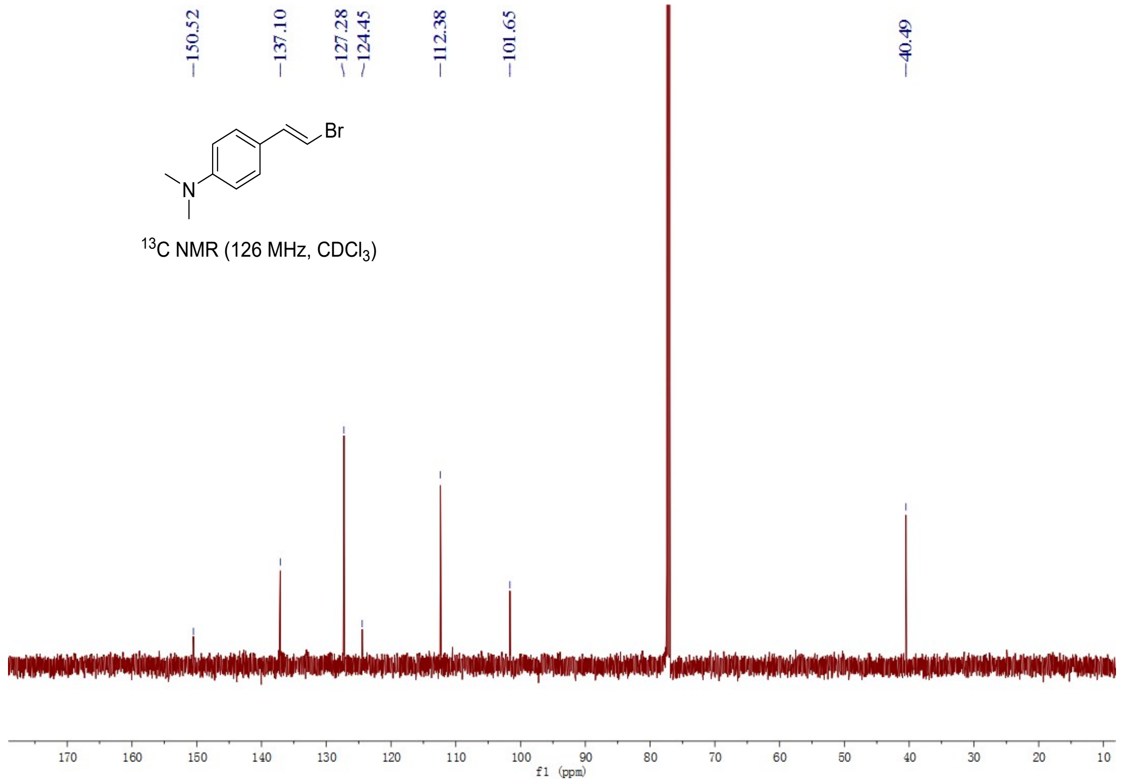
150.52
137.10
127.28
124.45
112.38
101.65
40.49
Br
N
13C NMR (126 MHz, CDCl3)
f1 (ppm)

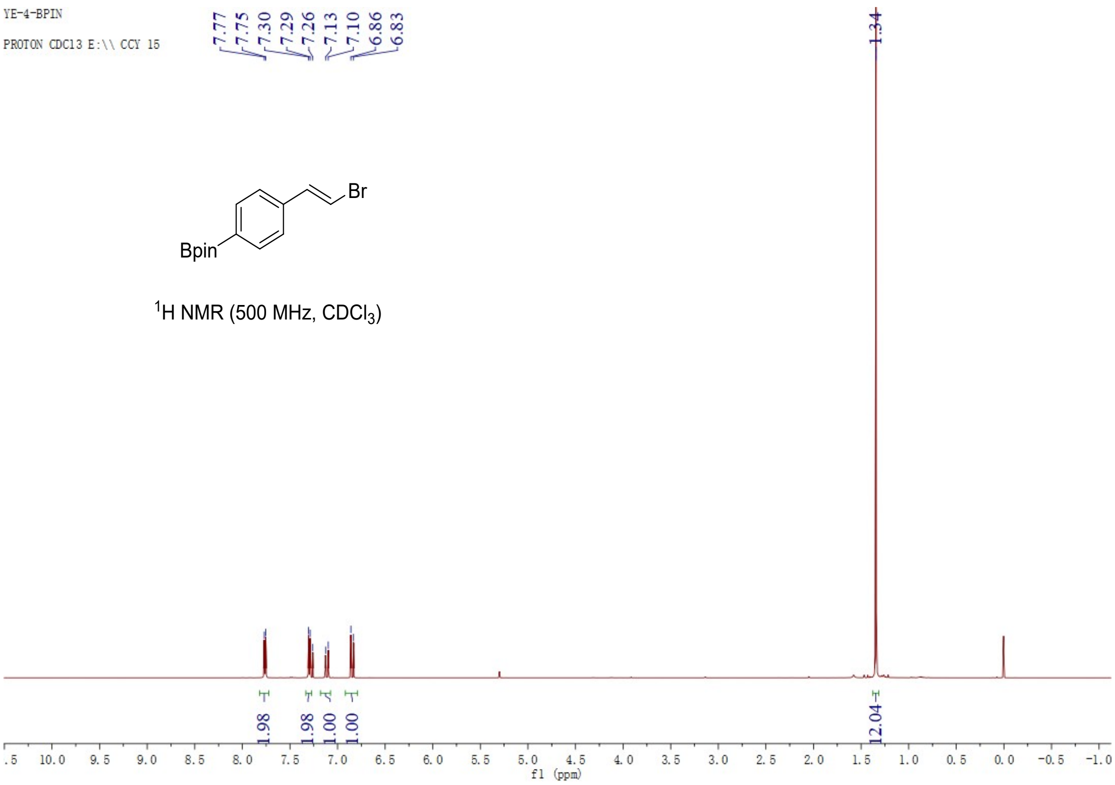
YE-4-BPIN
PROTON CDCl3 E:\\ CCY 15
7.77
7.75
7.30
7.29
7.26
7.13
7.10
6.86
6.83
1.34
Br
Bpin
1H NMR (500 MHz, CDCl3)
1.98
1.98
1.00
1.00
12.04
f1 (ppm)

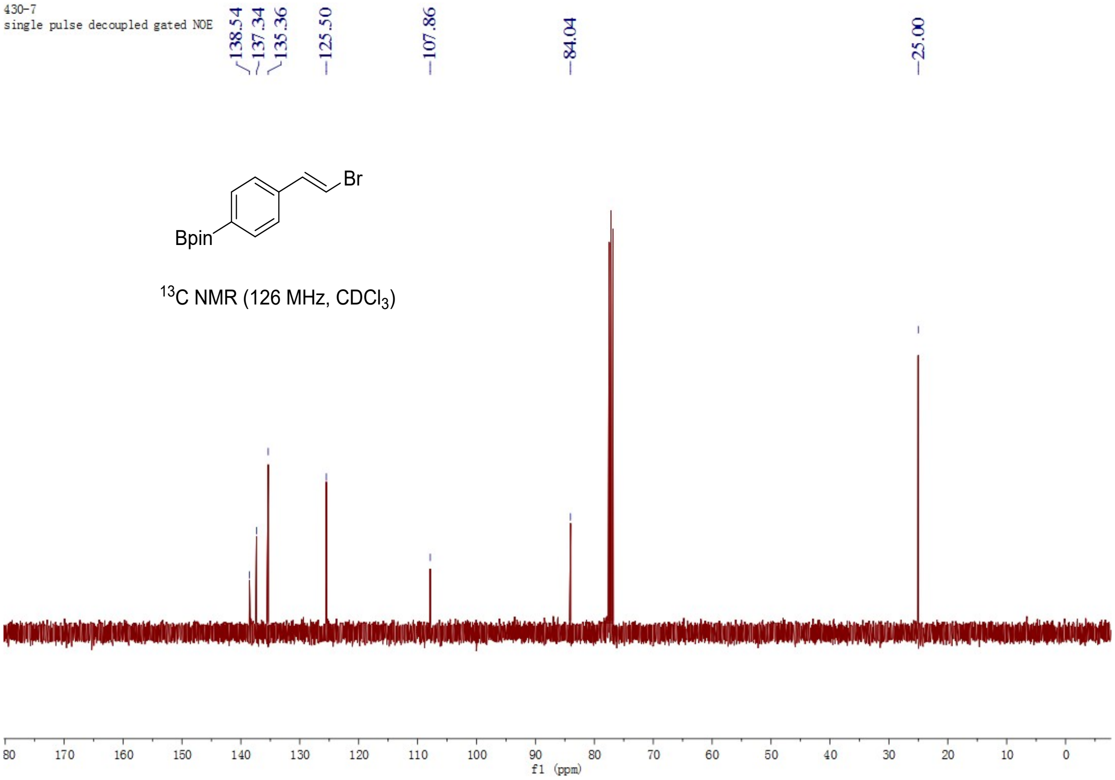
430-7
single pulse decoupled gated NOE
138.54
137.34
135.36
125.50
107.86
84.04
25.00
Br
Bpin
13C NMR (126 MHz, CDCl3)
f1 (ppm)

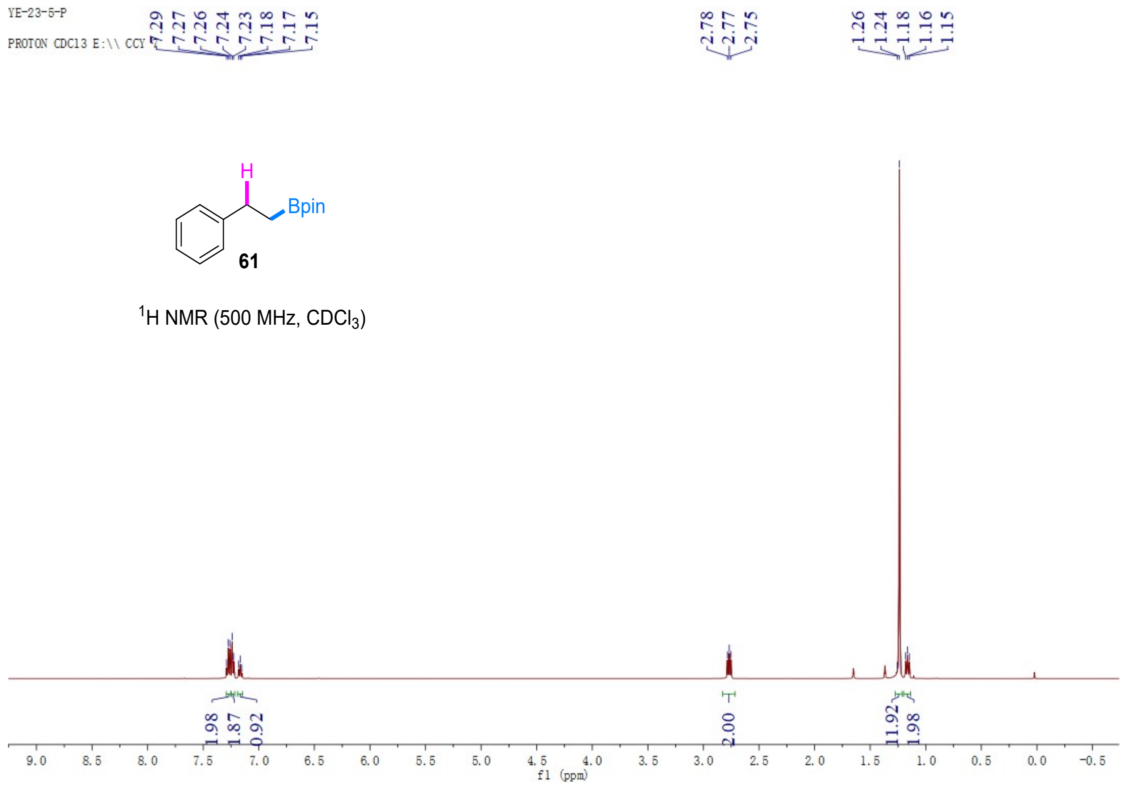

^{1}H NMR (500 MHz, $CDCl_3$)

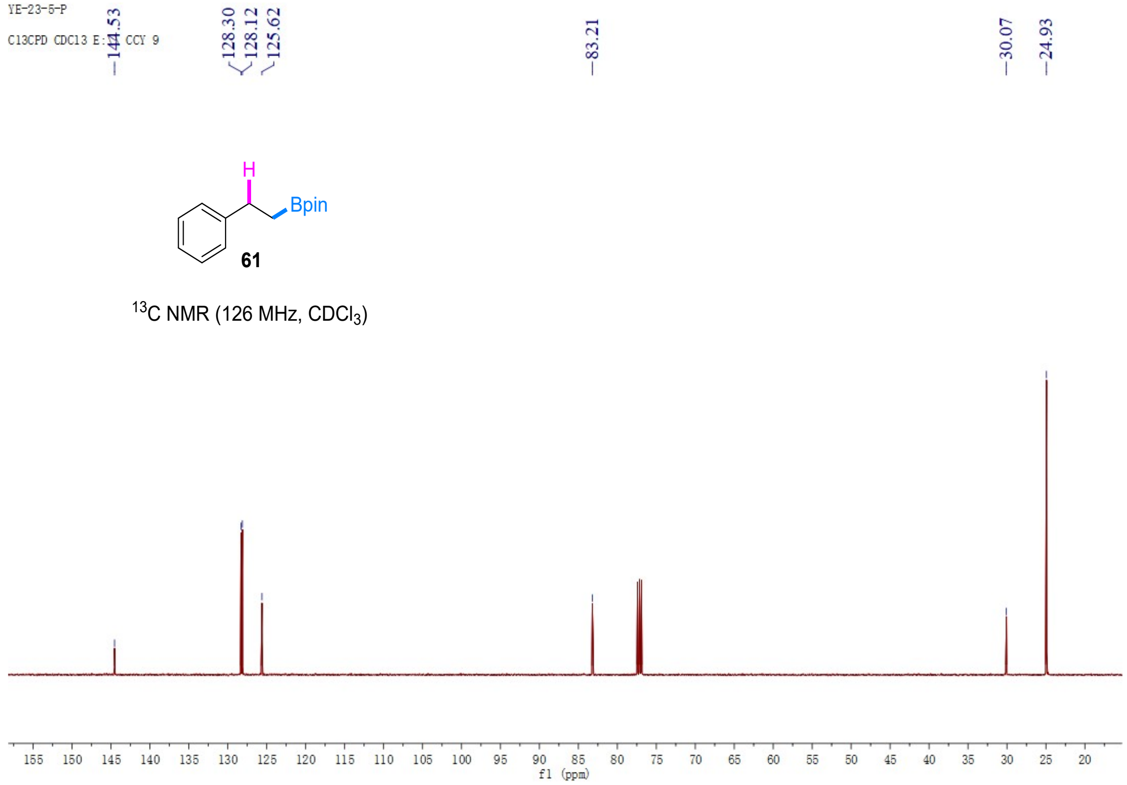

^{13}C NMR (126 MHz, $CDCl_3$)

IX. Spectral Data (HPLC Trace)

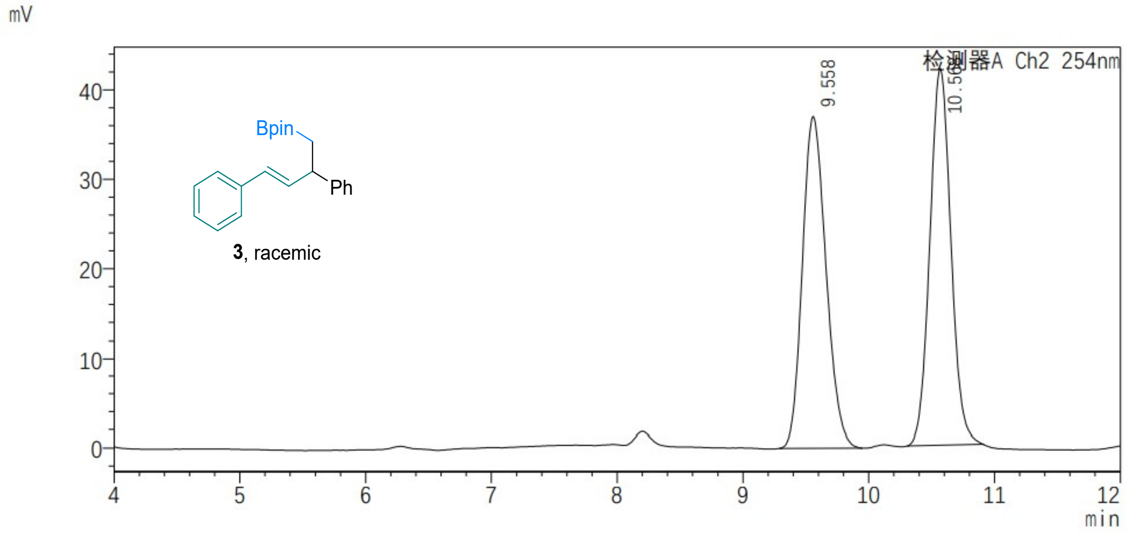

PDA Ch2 254nm

Peak#	Resolution Time	Area	Height	Area %	Height %
1	9. 558	481997	37031	49. 613	46. 810
2	10. 568	489511	42078	50. 387	53. 190
Total		971507	79108	100. 000	100. 000

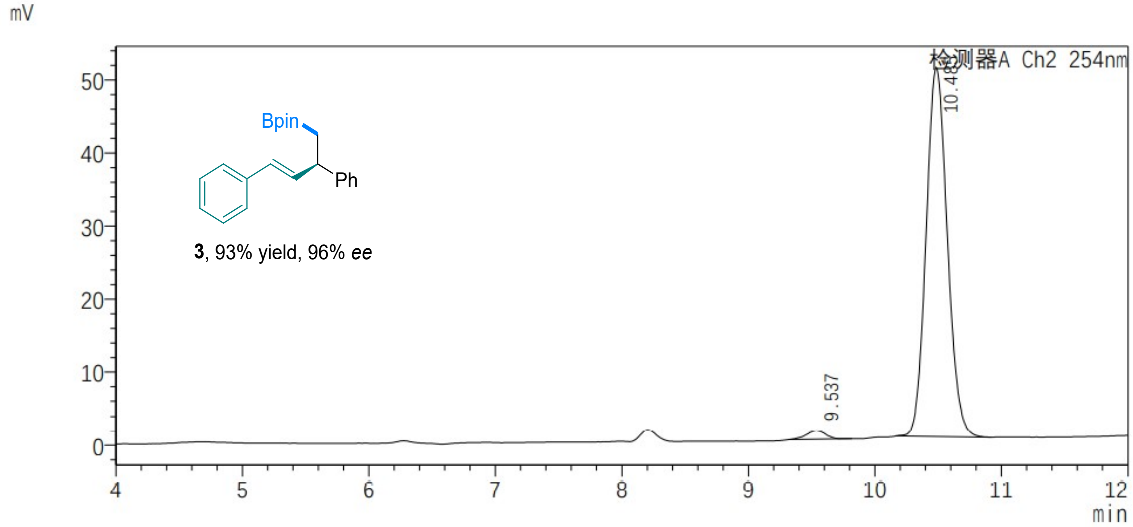

PDA Ch2 254nm

Peak#	Resolution Time	Area	Height	Area %	Height %
1	9. 537	13046	1212	2. 166	2. 343
2	10. 485	589222	50519	97. 834	97. 657
Total		602268	51731	100. 000	100. 000

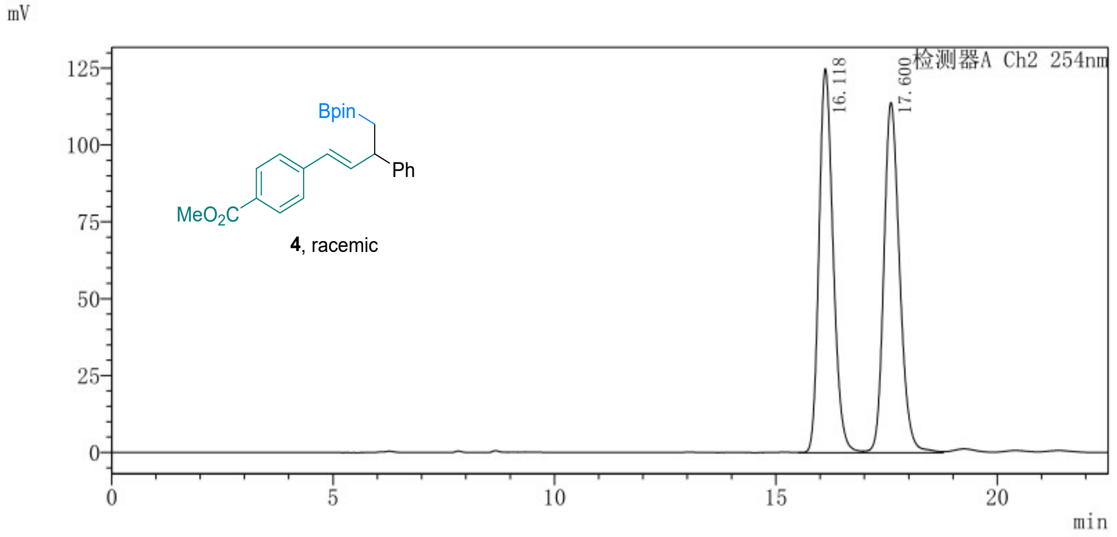

PDA Ch2 254nm

Peak#	Resolution Time	Area	Height	Area %	Height %
1	16. 118	2766788	124875	49. 474	52. 290
2	17. 600	2825630	113935	50. 526	47. 710
Total		5592418	238810	100. 000	100. 000

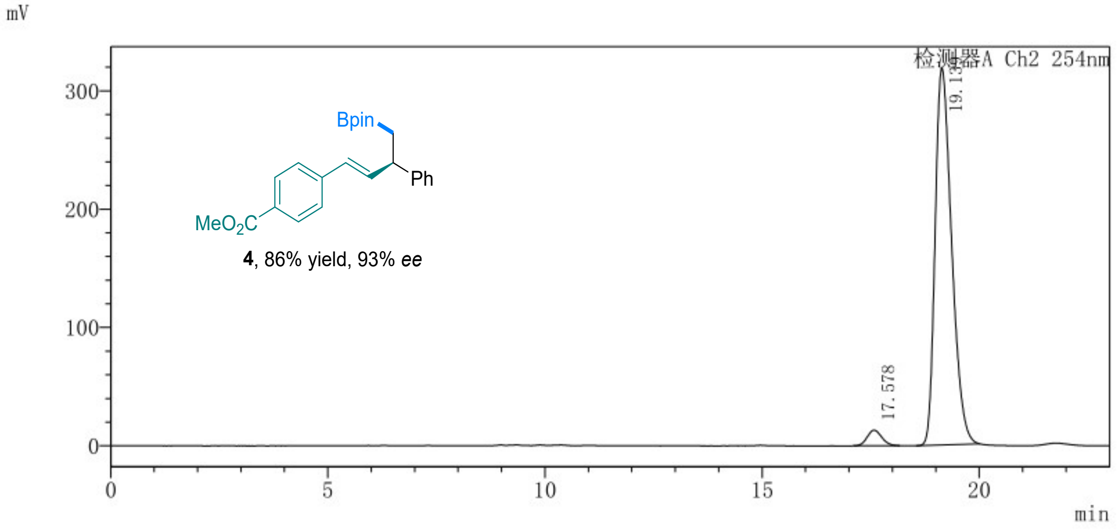

PDA Ch2 254nm

Peak#	Resolution Time	Area	Height	Area %	Height %
1	17. 578	308137	13176	3. 528	3. 969
2	19. 139	8425084	318769	96. 472	96. 031
Total		8733221	331945	100. 000	100. 000

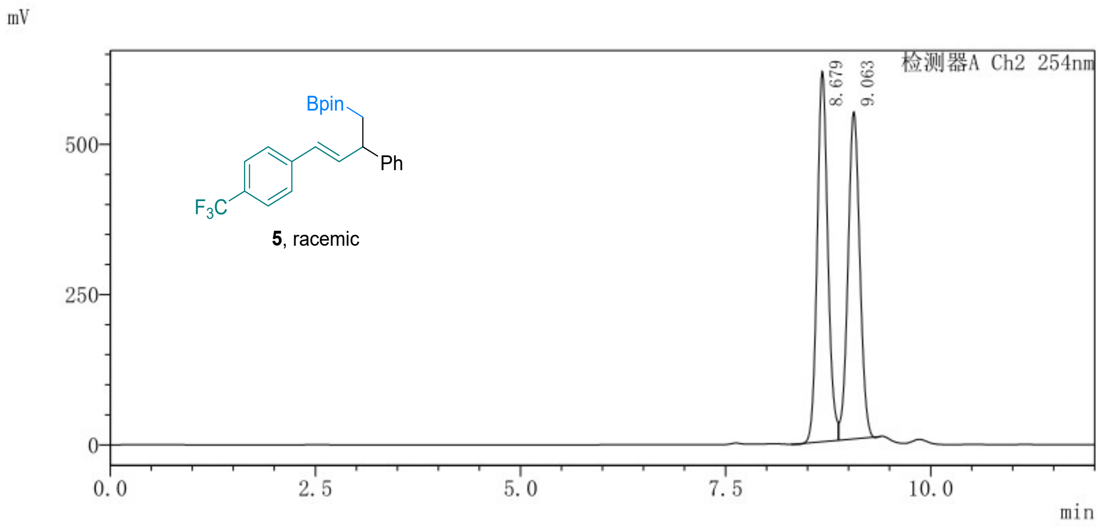

PDA Ch2 254nm

Peak#	Resolution Time	Area	Height	Area %	Height %
1	8.679	5560992	616379	50.478	53.096
2	9.063	5455769	544501	49.522	46.904
Total		11016761	1160880	100.000	100.000

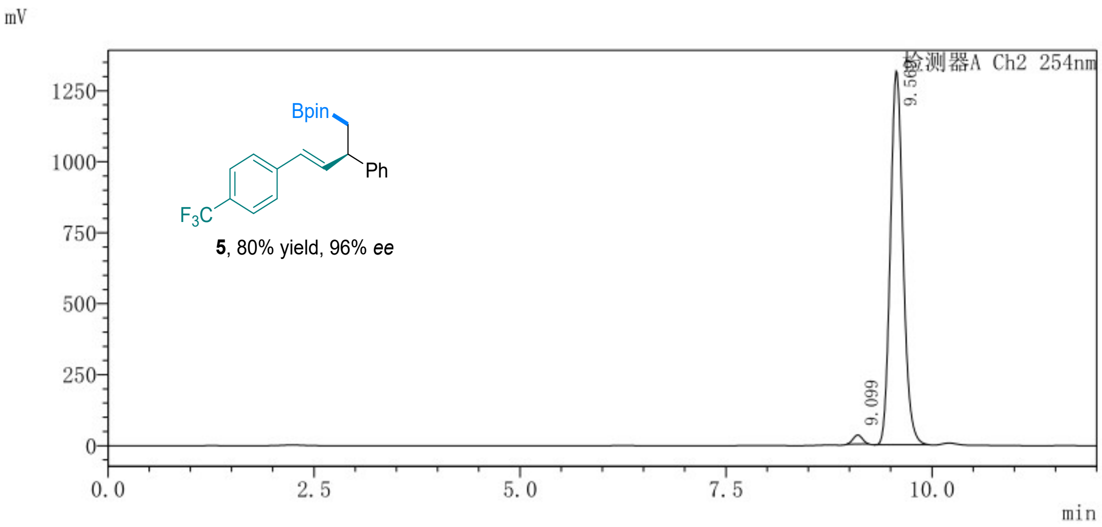

PDA Ch2 254nm

Peak#	Resolution Time	Area	Height	Area %	Height %
1	9.099	251800	31980	1.767	2.373
2	9.569	13996979	1315628	98.233	97.627
Total		14248779	1347608	100.000	100.000

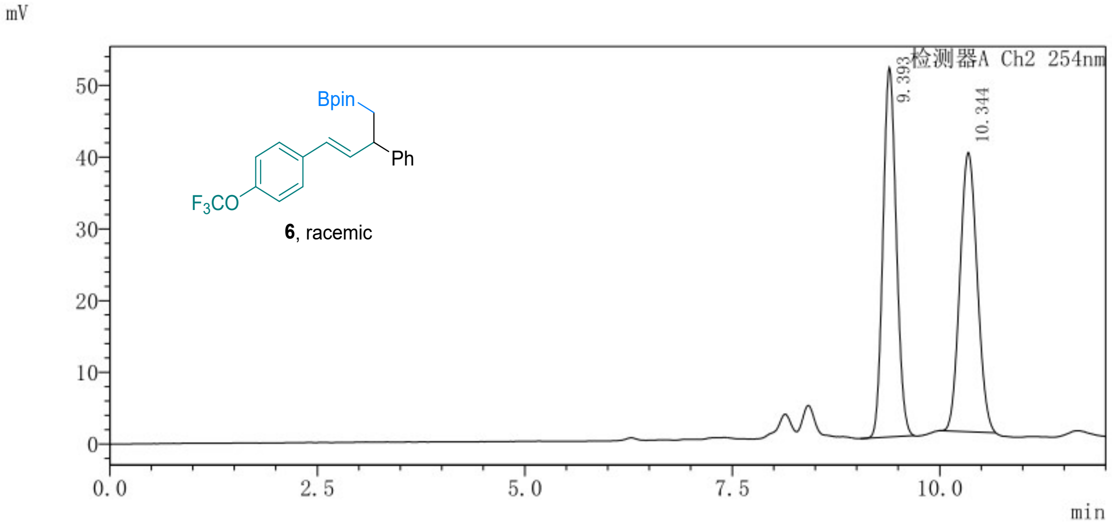

PDA Ch2 254nm

Peak#	Resolution Time	Area	Height	Area %	Height %
1	9.393	574449	51489	50.434	56.948
2	10.344	564555	38926	49.566	43.052
Total		1139004	90415	100.000	100.000

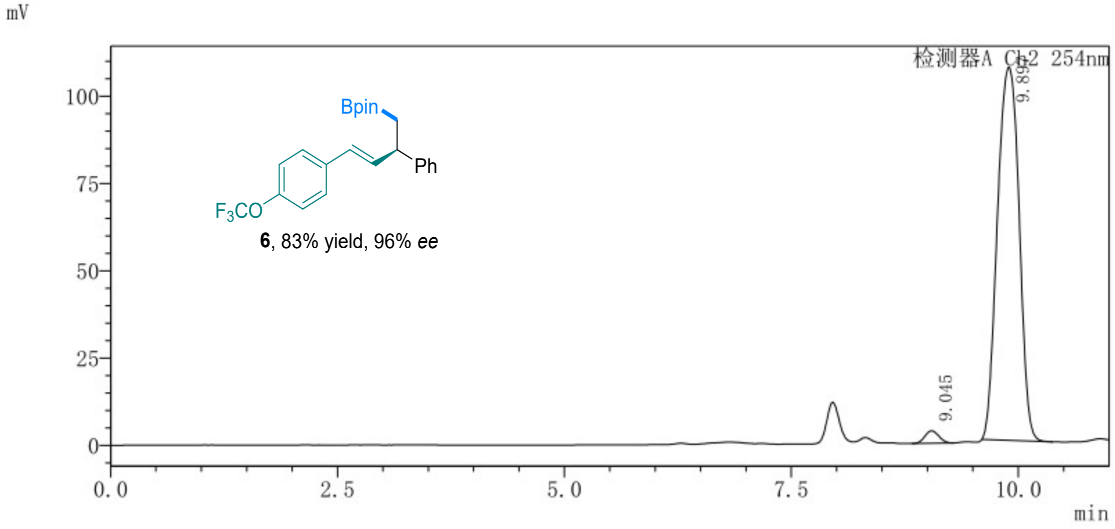

PDA Ch2 254nm

Peak#	Resolution Time	Area	Height	Area %	Height %
1	9.045	38261	3585	2.110	3.246
2	9.897	1775355	106863	97.890	96.754
Total		1813617	110448	100.000	100.000

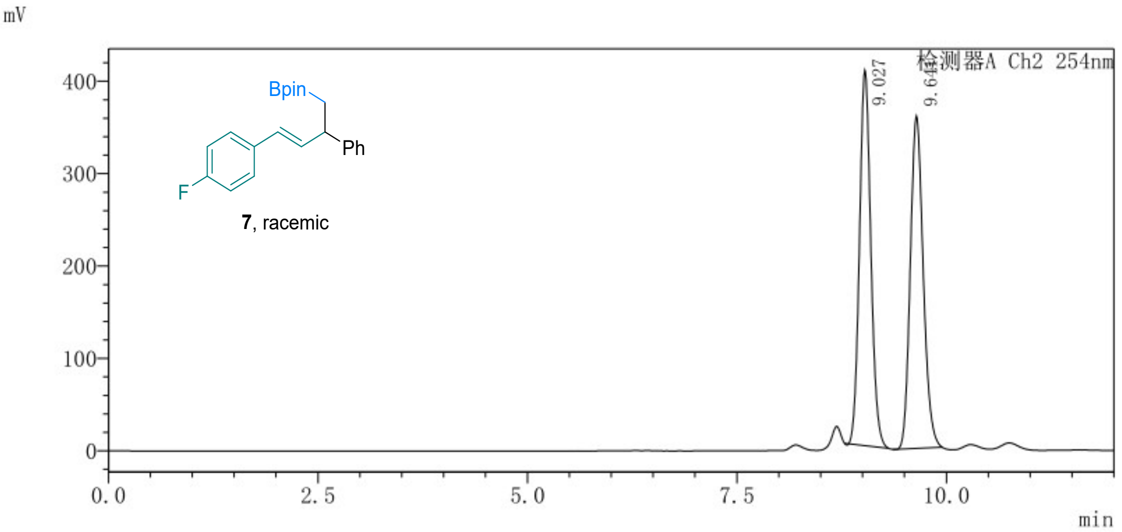

PDA Ch2 254nm

Peak#	Rasolution Time	Area	Height	Area %	Height %
1	9.027	3831563	406364	49.941	53.036
2	9.641	3840640	359840	50.059	46.964
Total		7672203	766204	100.000	100.000

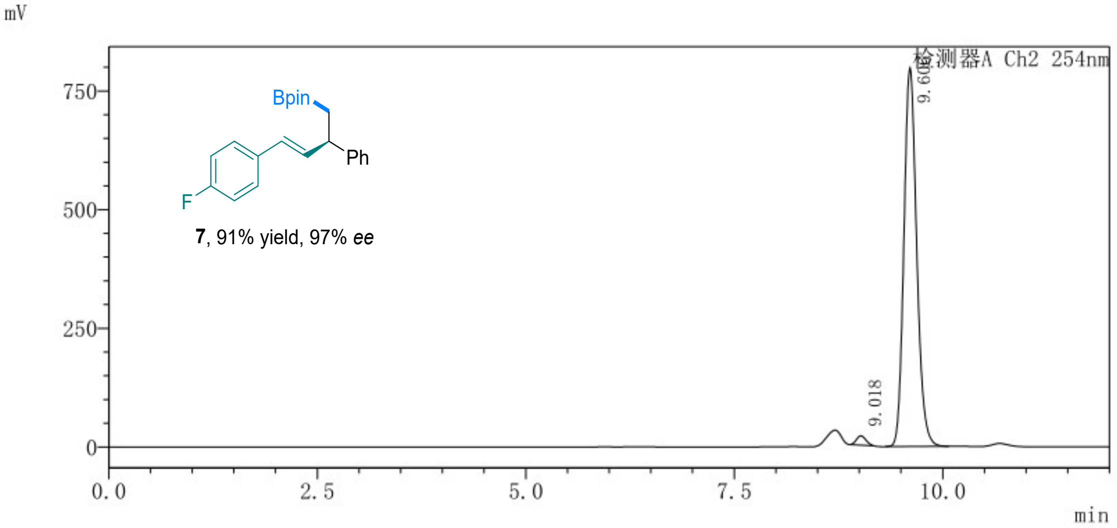

PDA Ch2 254nm

Peak#	Resolution Time	Area	Height	Area %	Height %
1	9.018	152030	19409	1.773	2.375
2	9.606	8423013	797749	98.227	97.625
Total		8575043	817158	100.000	100.000

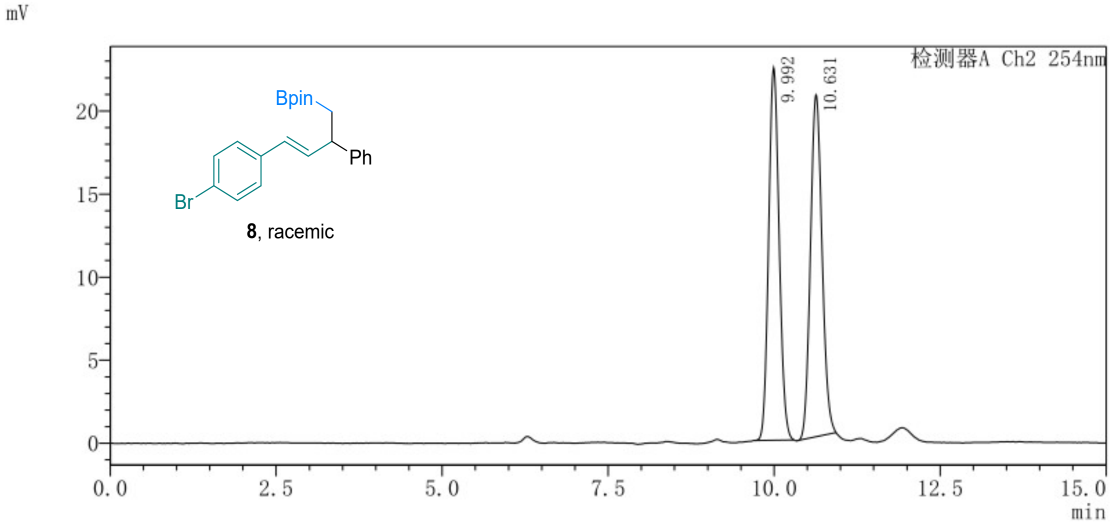

PDA Ch2 254nm

Peak#	Resolution Time	Area	Height	Area %	Height %
1	9.992	247185	22440	49.724	52.164
2	10.631	249925	20577	50.276	47.836
Total		497109	43017	100.000	100.000

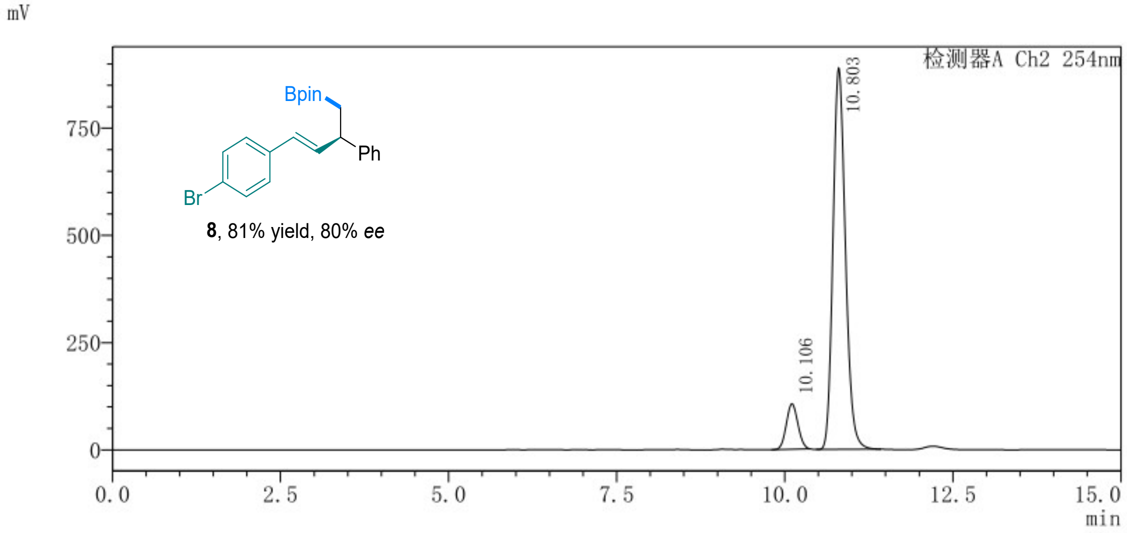

PDA Ch2 254nm

Peak#	Resolution Time	Area	Height	Area %	Height %
1	10.106	1276224	106071	10.010	10.657
2	10.803	11472949	889229	89.990	89.343
Total		12749173	995300	100.000	100.000

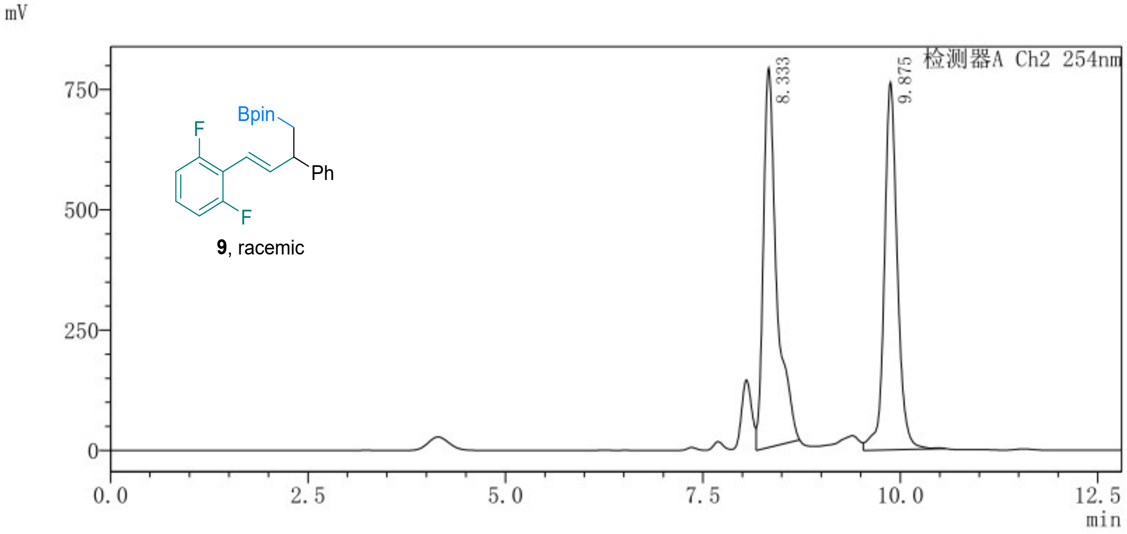

PDA Ch2 254nm

Peak#	Resolution Time	Area	Height	Area %	Height %
1	8. 333	9250442	788380	51. 380	50. 791
2	9. 875	8753443	763823	48. 620	49. 209
Total		18003885	1552203	100. 000	100. 000

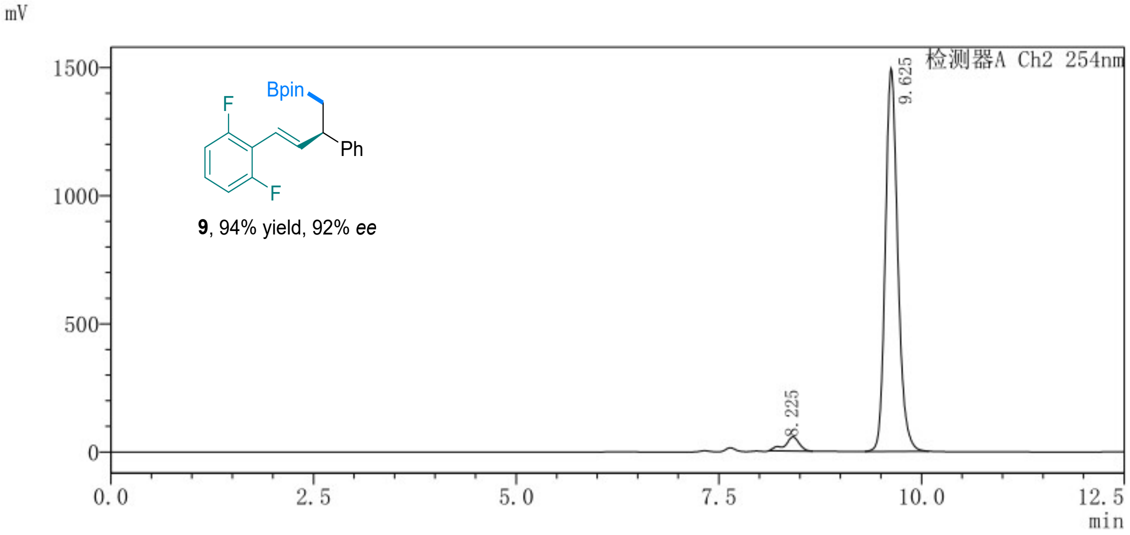

PDA Ch2 254nm

Peak#	Resolution Time	Area	Height	Area %	Height %
1	8. 225	687184	16825	4. 123	1. 114
2	9. 625	15981786	1493856	95. 877	98. 886
Total		16668971	1510682	100. 000	100. 000

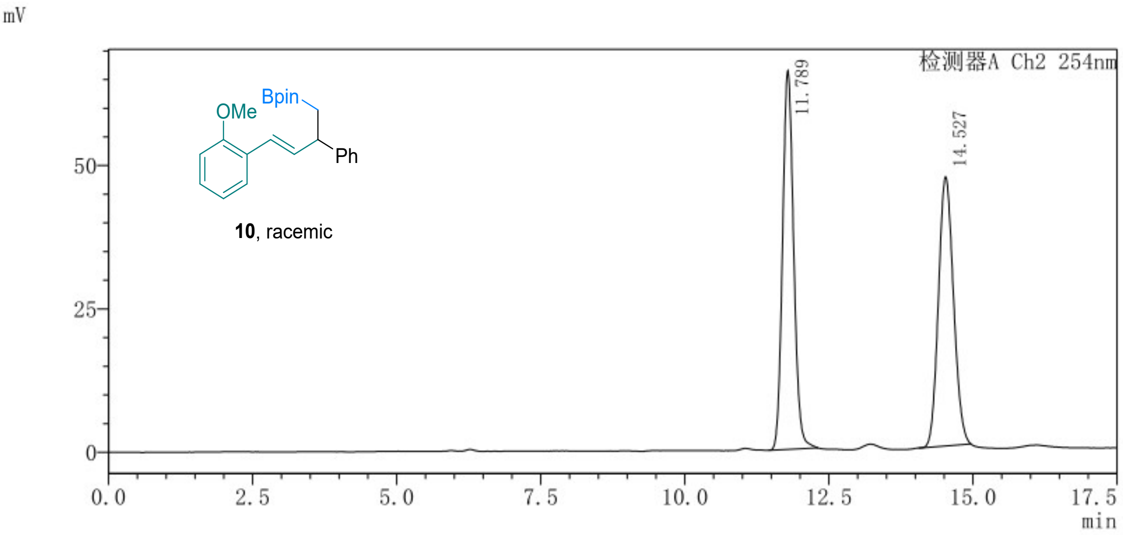

PDA Ch2 254nm

Peak#	Resolution Time	Area	Height	Area %	Height %
1	11.789	884377	66028	50.948	58.449
2	14.527	851457	46940	49.052	41.551
Total		1735835	112968	100.000	100.000

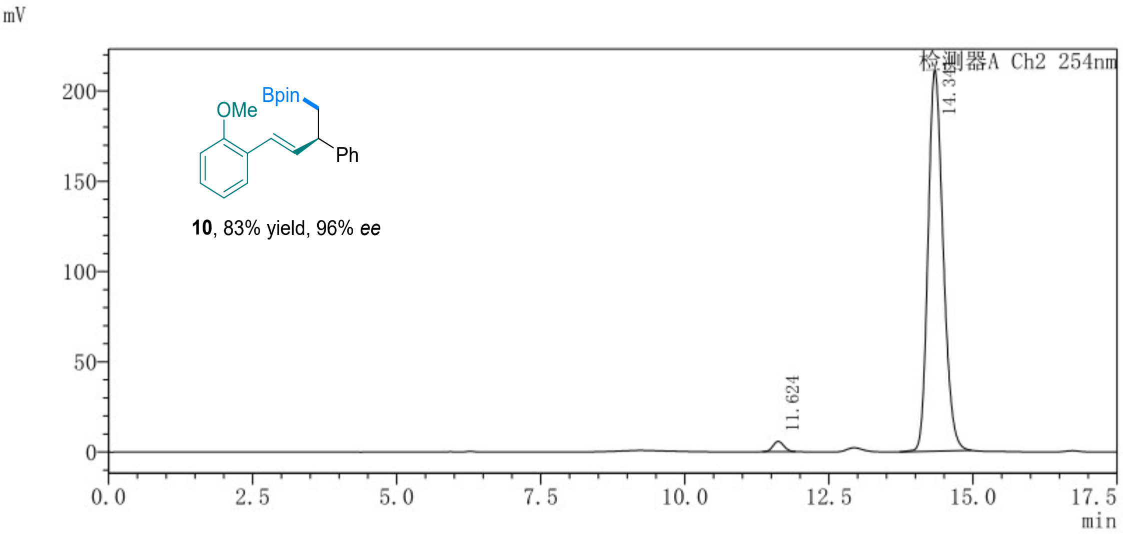

PDA Ch2 254nm

Peak#	Resolution Time	Area	Height	Area %	Height %
1	11.624	75978	5741	1.937	2.650
2	14.341	3846191	210901	98.063	97.350
Total		3922169	216641	100.000	100.000

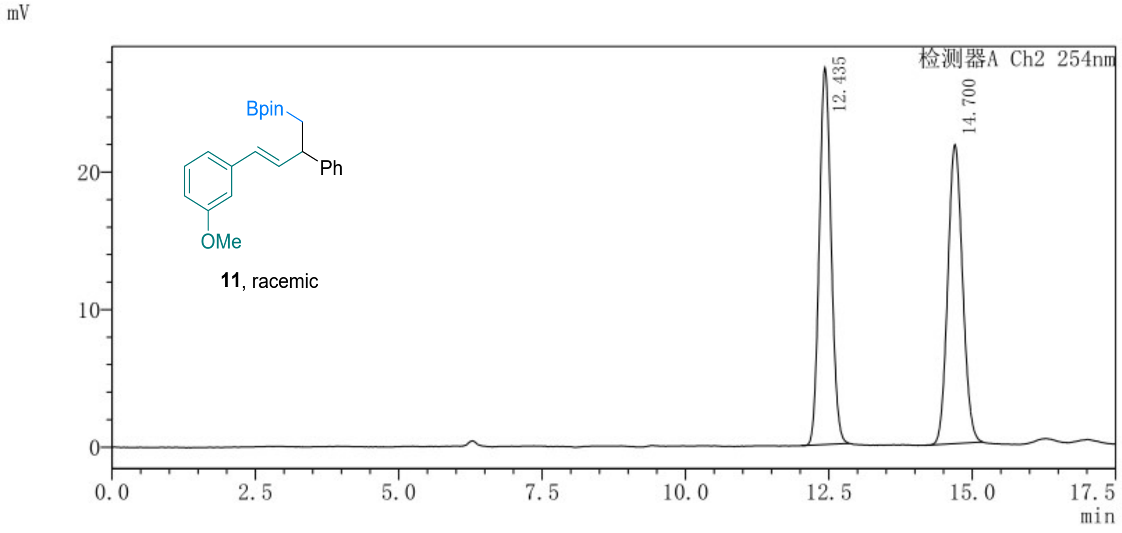

PDA Ch2 254nm

Peak#	Resolution Time	Area	Height	Area %	Height %
1	12. 435	392983	27406	50. 171	55. 751
2	14. 700	390303	21752	49. 829	44. 249
Total		783286	49158	100. 000	100. 000

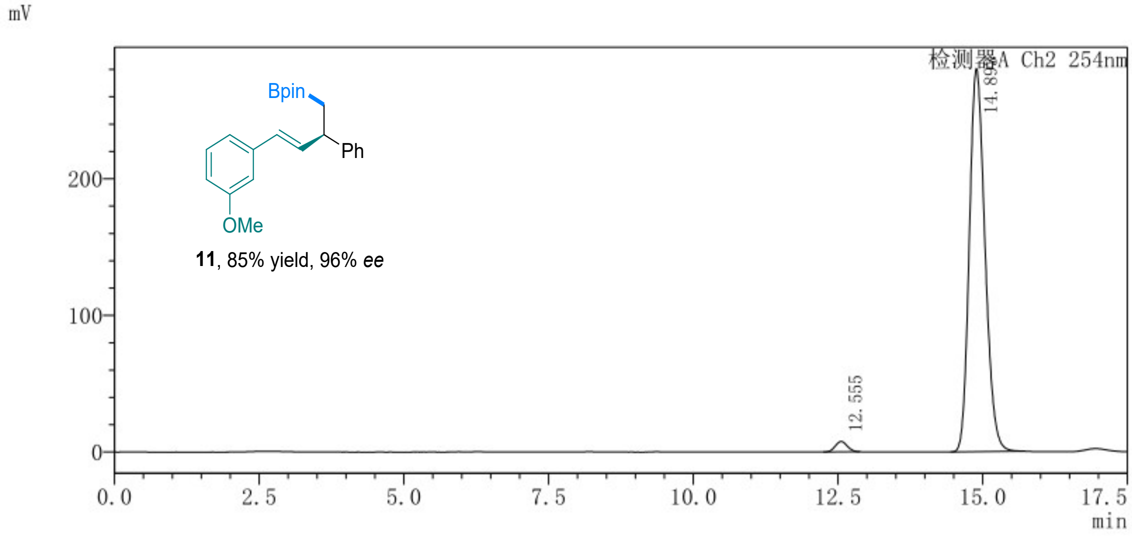

PDA Ch2 254nm

Peak#	Resolution Time	Area	Height	Area %	Height %
1	12. 555	111205	7656	2. 061	2. 658
2	14. 893	5283387	280350	97. 939	97. 342
Total		5394592	288006	100. 000	100. 000

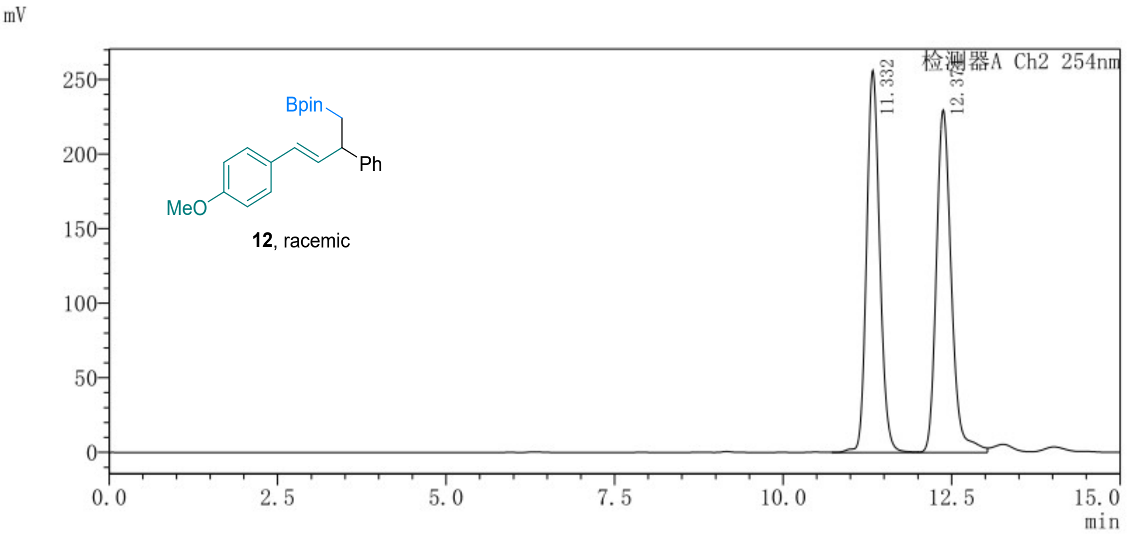

PDA Ch2 254nm

Peak#	Resolution Time	Area	Height	Area %	Height %
1	11. 332	3490542	256261	49. 473	52. 716
2	12. 374	3564931	229859	50. 527	47. 284
Total		7055473	486120	100. 000	100. 000

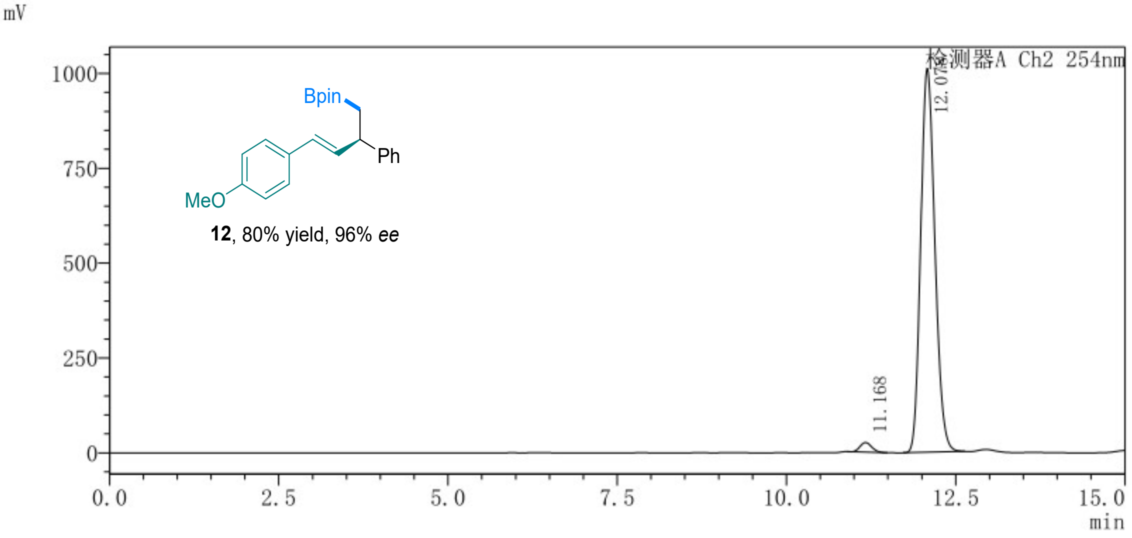

PDA Ch2 254nm

Peak#	Resolution Time	Area	Height	Area %	Height %
1	11. 168	304085	25271	2. 039	2. 438
2	12. 078	14611514	1011410	97. 961	97. 562
Total		14915599	1036681	100. 000	100. 000

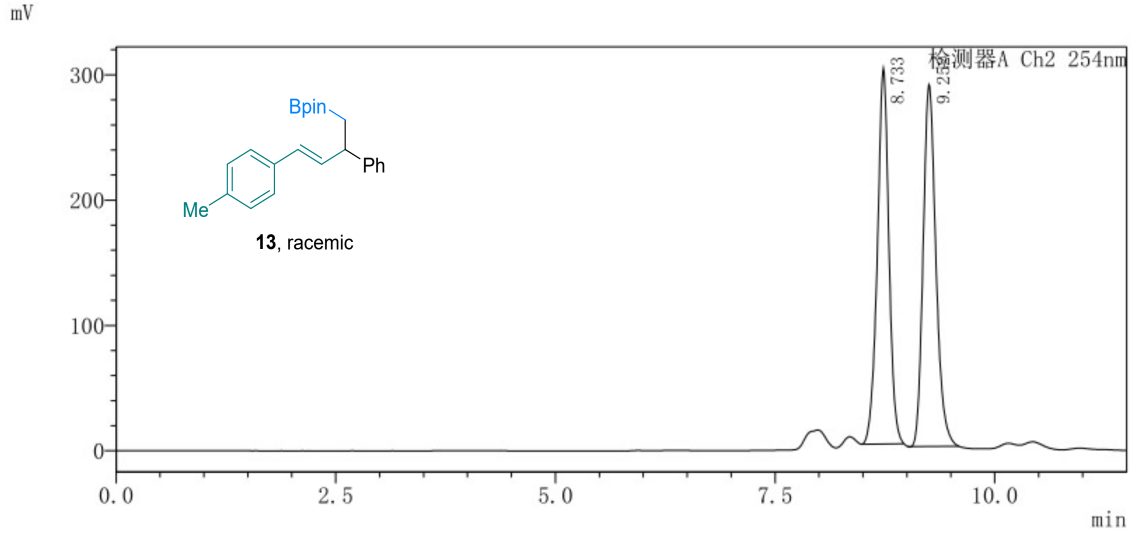

PDA Ch2 254nm

Peak#	Resolution Time	Area	Height	Area %	Height %
1	8. 733	2750707	299802	48. 507	50. 941
2	9. 252	2920043	288721	51. 493	49. 059
Total		5670750	588523	100. 000	100. 000

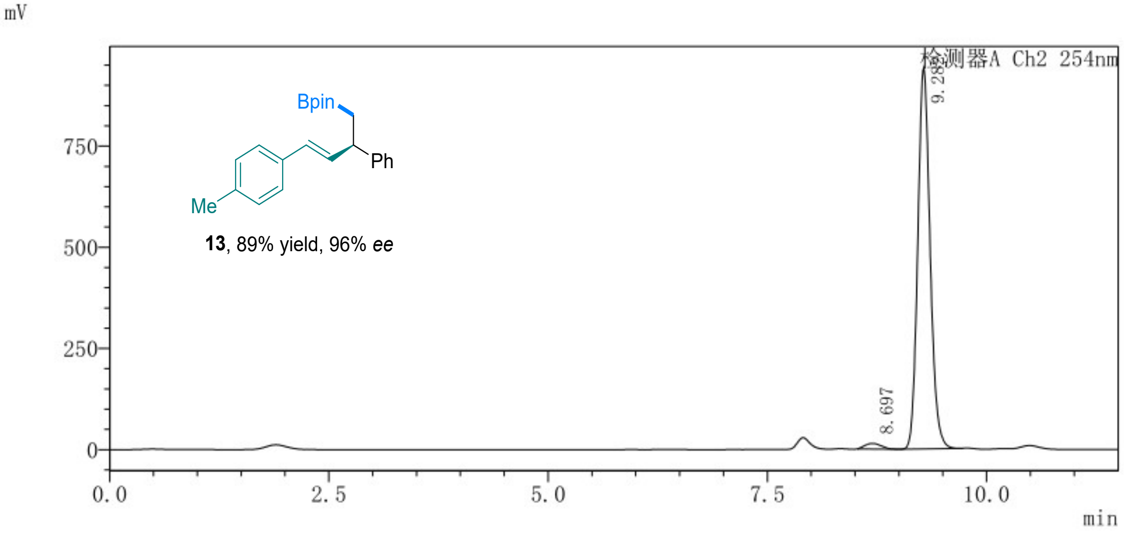

PDA Ch2 254nm

Peak#	Resolution Time	Area	Height	Area %	Height %
1	8. 697	175855	13493	1. 875	1. 413
2	9. 282	9200890	941632	98. 125	98. 587
Total		9376745	955124	100. 000	100. 000

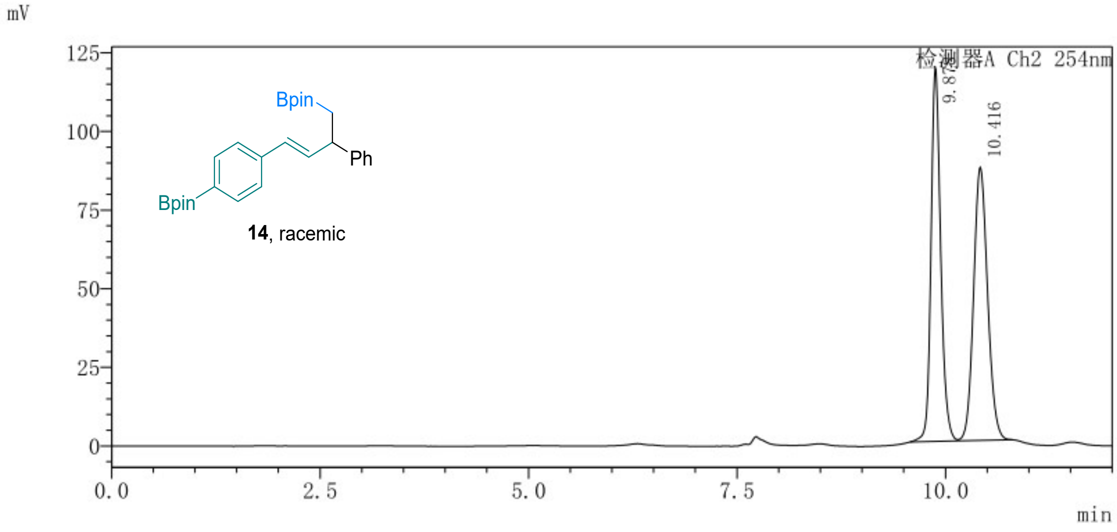

PDA Ch2 254nm

Peak#	Resolution Time	Area	Height	Area %	Height %
1	9.878	960158	118761	48.612	57.738
2	10.416	1014974	86929	51.388	42.262
Total		1975132	205690	100.000	100.000

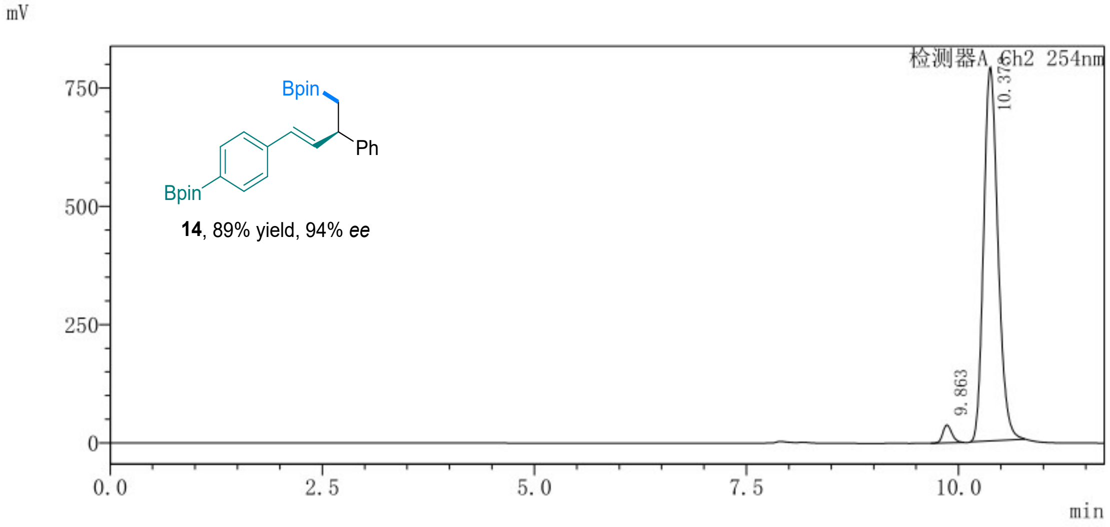

PDA Ch2 254nm

Peak#	Resolution Time	Area	Height	Area %	Height %
1	9.863	285648	37904	2.991	4.579
2	10.373	9263778	789887	97.009	95.421
Total		9549426	827792	100.000	100.000

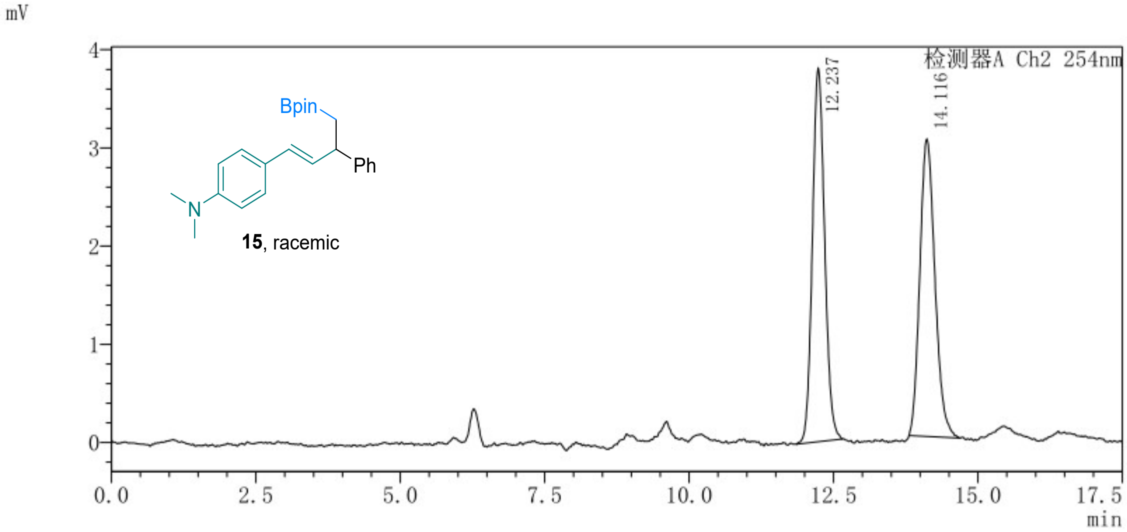

PDA Ch2 254nm

Peak#	Resolution Time	Area	Height	Area %	Height %
1	12.237	56098	3803	50.427	55.672
2	14.116	55147	3028	49.573	44.328
Total		111245	6831	100.000	100.000

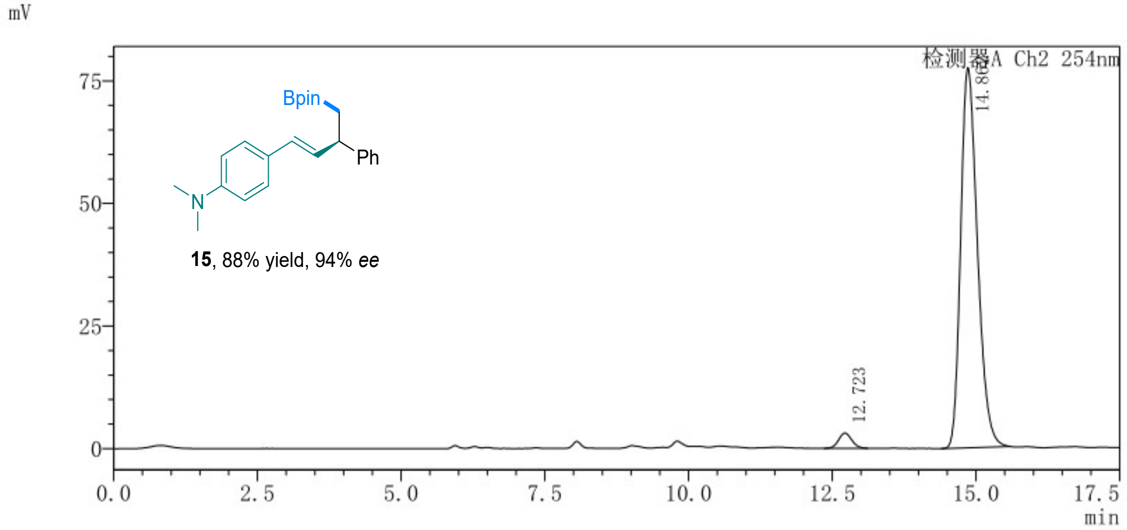

PDA Ch2 254nm

Peak#	Resolution Time	Area	Height	Area %	Height %
1	12.723	49967	3117	3.083	3.866
2	14.862	1570744	77518	96.917	96.134
Total		1620711	80635	100.000	100.000

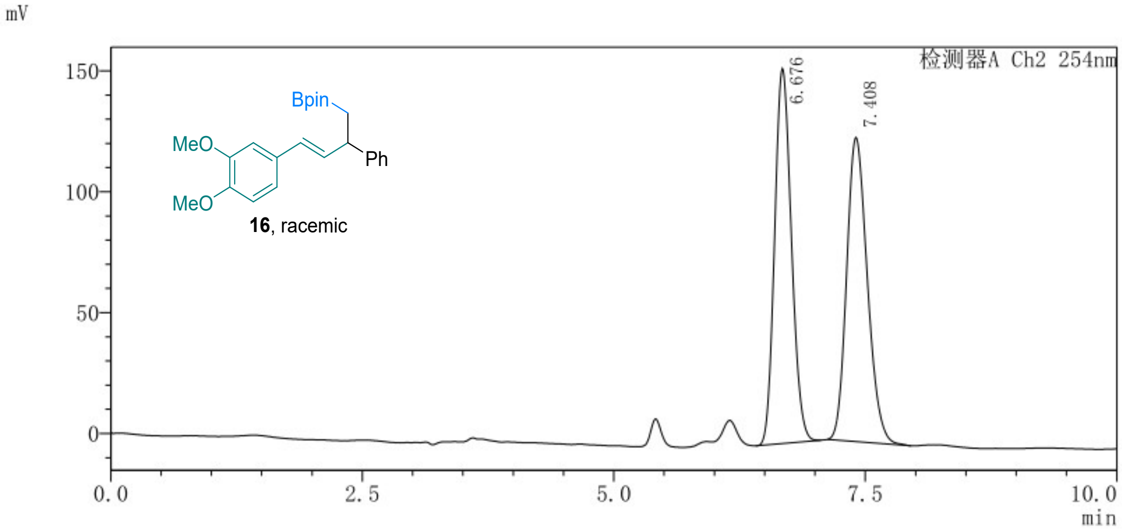

PDA Ch2 254nm

Peak#	Resolution Time	Area	Height	Area %	Height %
1	6.676	1798322	155116	49.974	55.214
2	7.408	1800164	125821	50.026	44.786
Total		3598487	280937	100.000	100.000

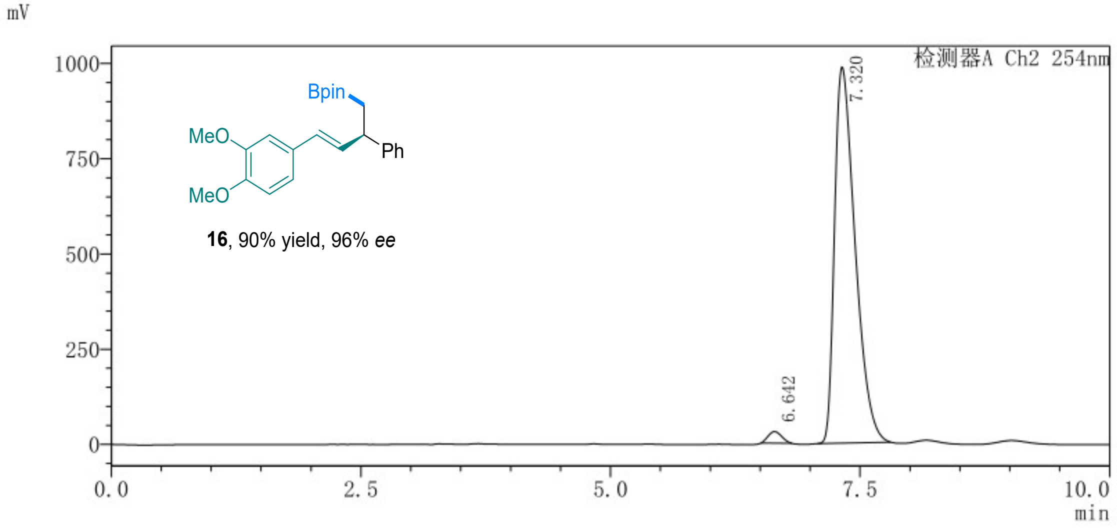

PDA Ch2 254nm

Peak#	Resolution Time	Area	Height	Area %	Height %
1	6.642	285660	30256	1.981	2.975
2	7.320	14134854	986892	98.019	97.025
Total		14420514	1017148	100.000	100.000

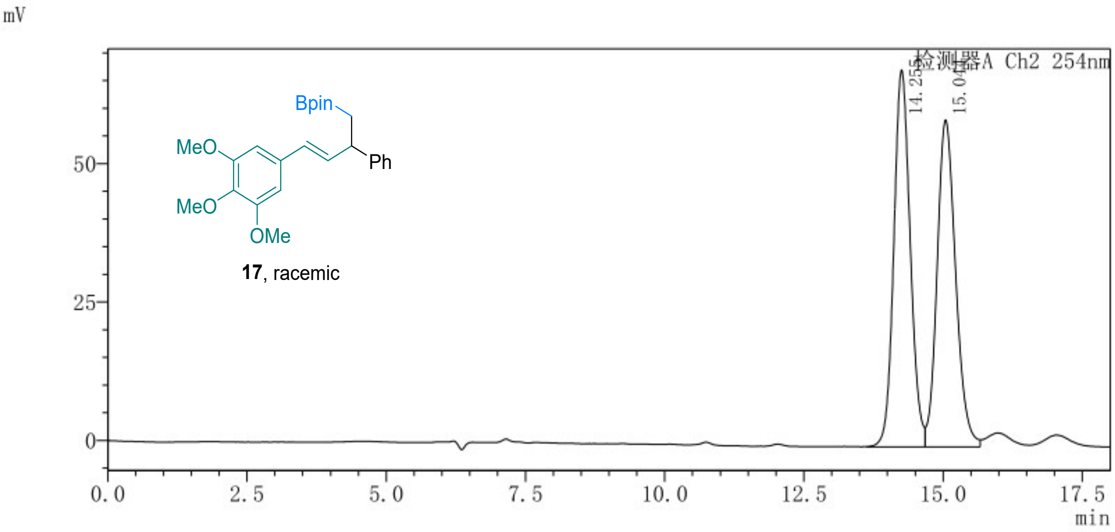

PDA Ch2 254nm

Peak#	Resolution Time	Area	Height	Area %	Height %
1	14.255	1392790	68070	50.393	53.541
2	15.044	1371086	59067	49.607	46.459
Total		2763876	127137	100.000	100.000

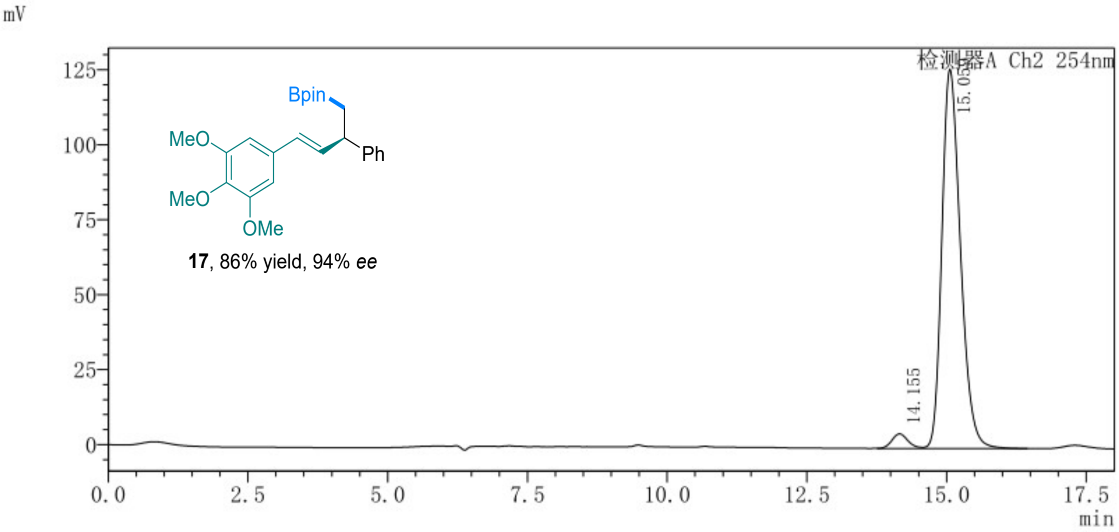

PDA Ch2 254nm

Peak#	Resolution Time	Area	Height	Area %	Height %
1	14.155	98899	4888	3.268	3.721
2	15.059	2927852	126475	96.732	96.279
Total		3026751	131364	100.000	100.000

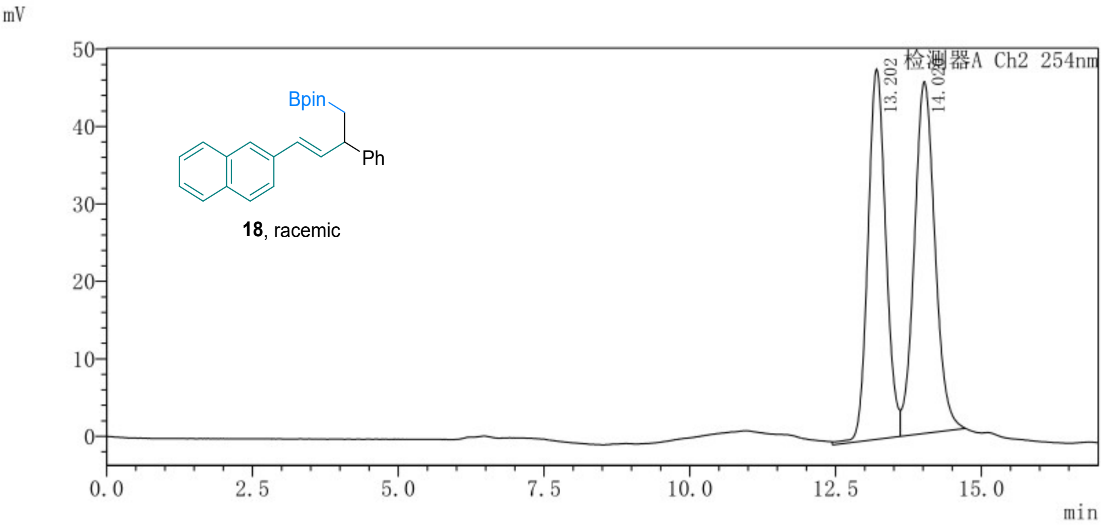

PDA Ch2 254nm

Peak#	Resolution Time	Area	Height	Area %	Height %
1	13.202	1017566	47794	47.638	51.280
2	14.020	1118485	45408	52.362	48.720
Total		2136052	93202	100.000	100.000

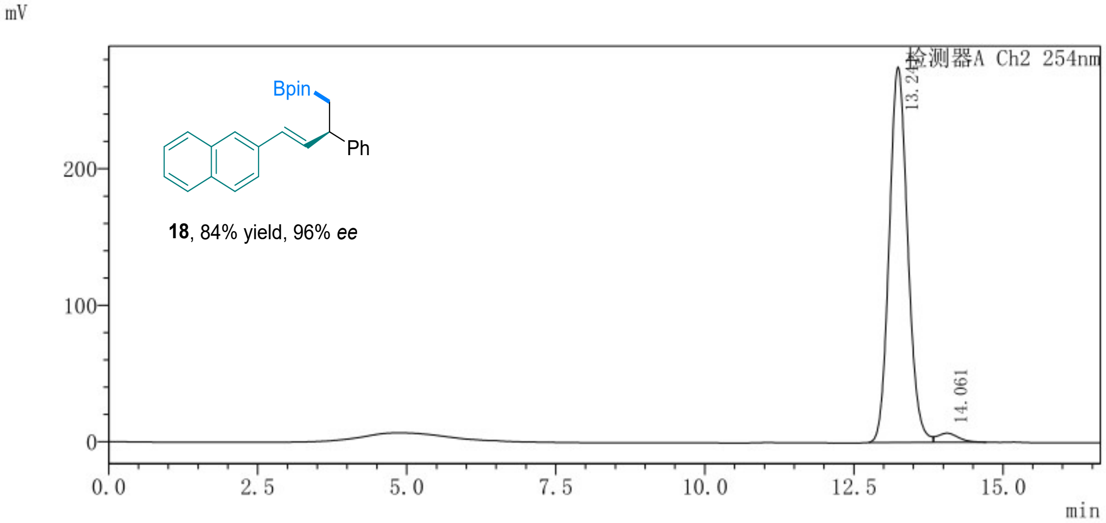

PDA Ch2 254nm

Peak#	Resolution Time	Area	Height	Area %	Height %
1	13.241	5917737	274935	97.334	97.644
2	14.061	162092	6634	2.666	2.356
Total		6079829	281569	100.000	100.000

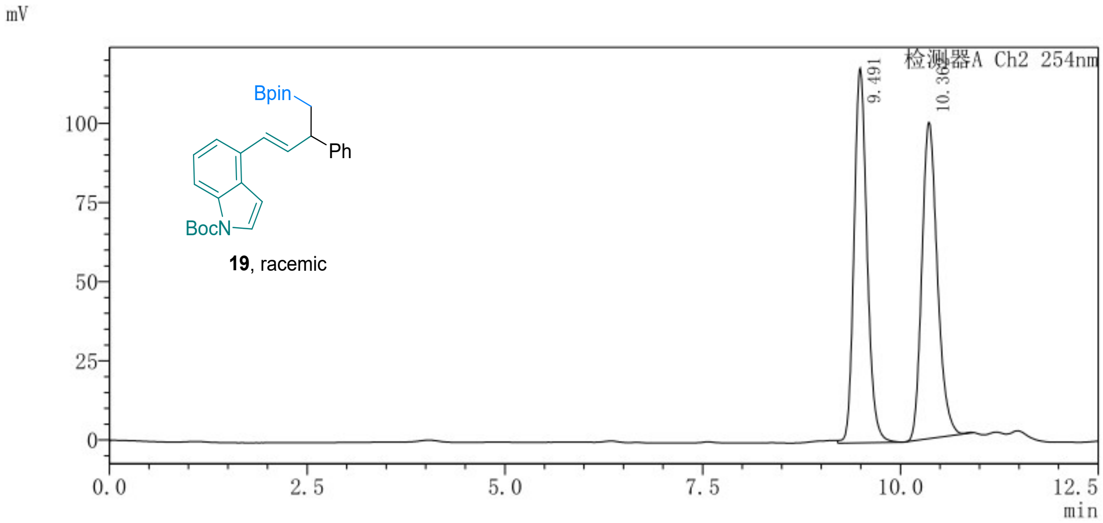

PDA Ch2 254nm

Peak#	Resolution Time	Area	Height	Area %	Height %
1	9. 491	1347027	118316	49. 703	54. 210
2	10. 362	1363138	99940	50. 297	45. 790
Total		2710165	218256	100. 000	100. 000

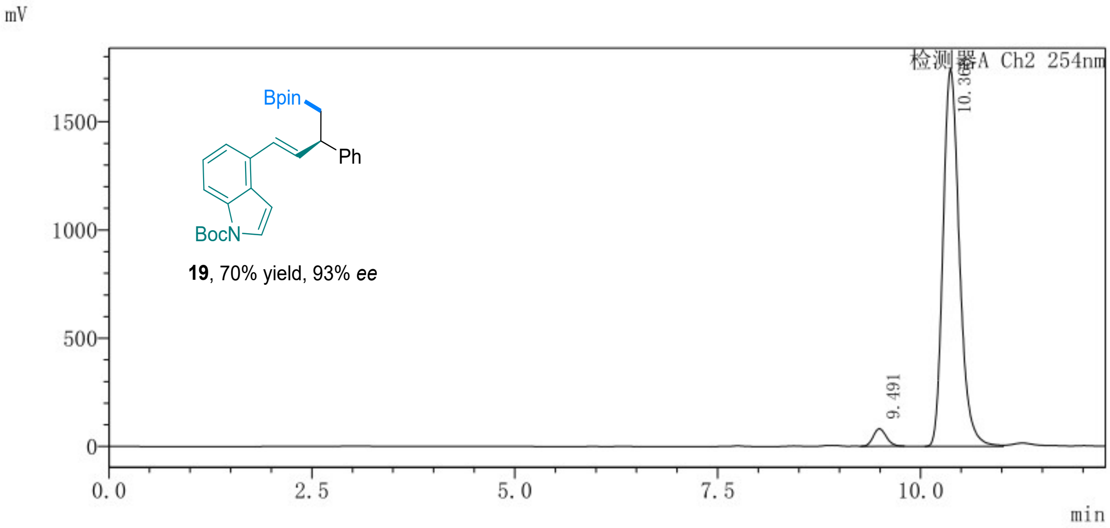

PDA Ch2 254nm

Peak#	Resolution Time	Area	Height	Area %	Height %
1	9. 491	931140	81806	3. 674	4. 485
2	10. 368	24413060	1742264	96. 326	95. 515
Total		25344200	1824070	100. 000	100. 000

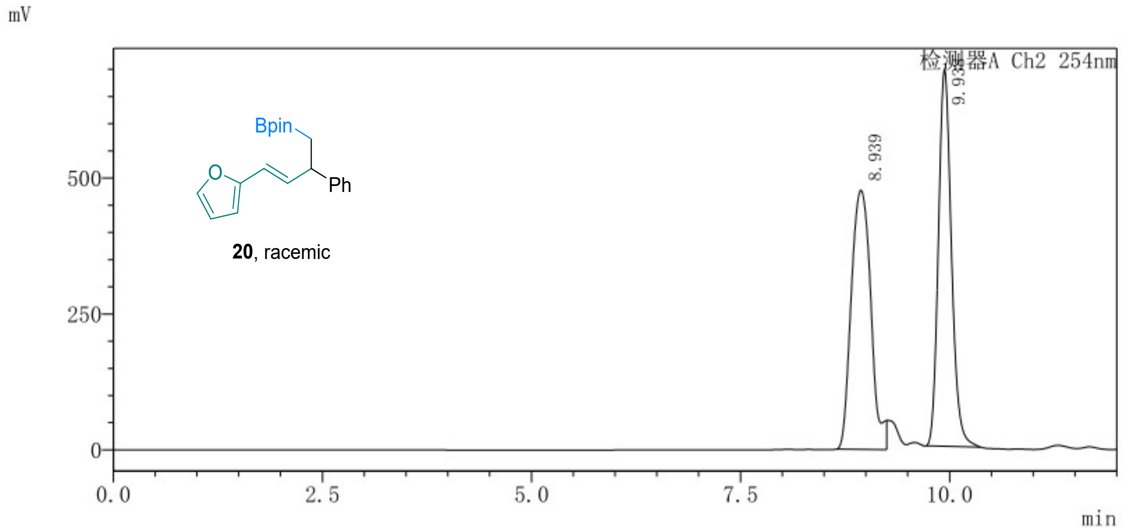

PDA Ch2 254nm

Peak#	Resolution Time	Area	Height	Area %	Height %
1	8.939	7782772	476629	50.903	40.769
2	9.938	7506776	692477	49.097	59.231
Total		15289547	1169106	100.000	100.000

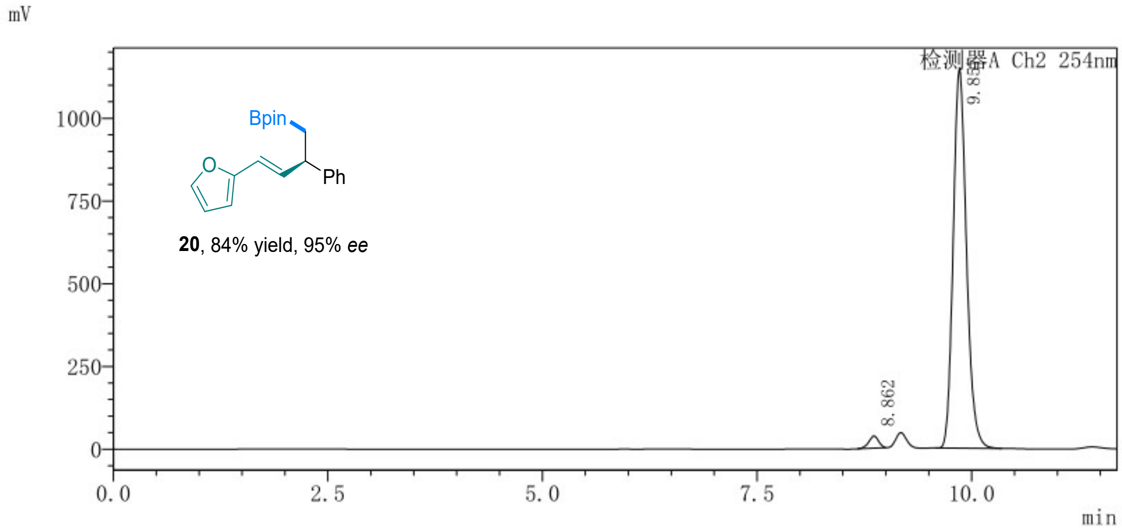

PDA Ch2 254nm

Peak#	Resolution Time	Area	Height	Area %	Height %
1	8.862	293476	36427	2.318	3.082
2	9.857	12368352	1145446	97.682	96.918
Total		12661827	1181873	100.000	100.000

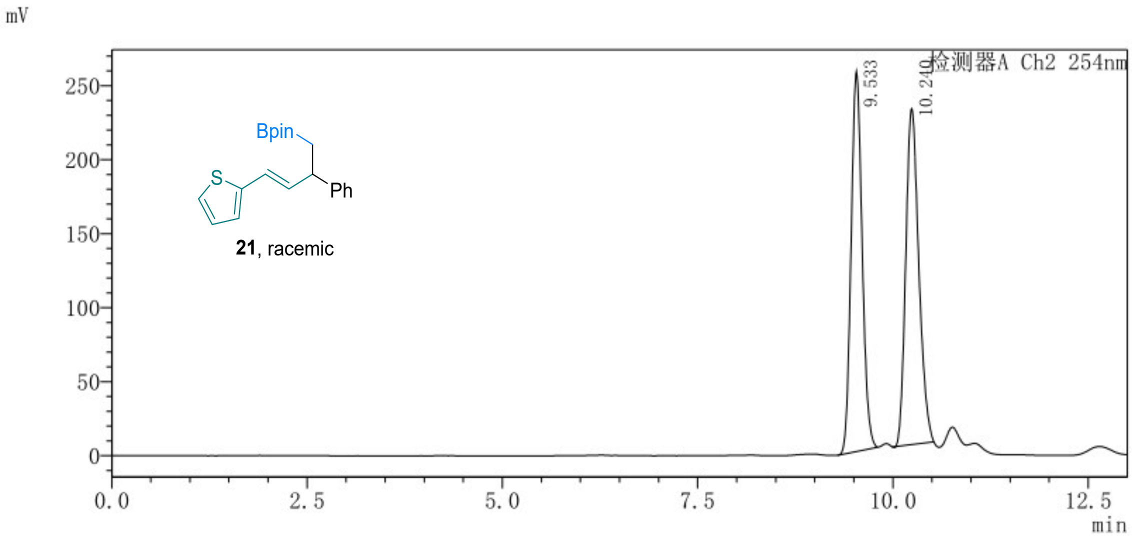

PDA Ch2 254nm

Peak#	Resolution Time	Area	Height	Area %	Height %
1	9. 533	2519904	256949	48. 905	53. 083
2	10. 240	2632698	227101	51. 095	46. 917
Total		5152602	484050	100. 000	100. 000

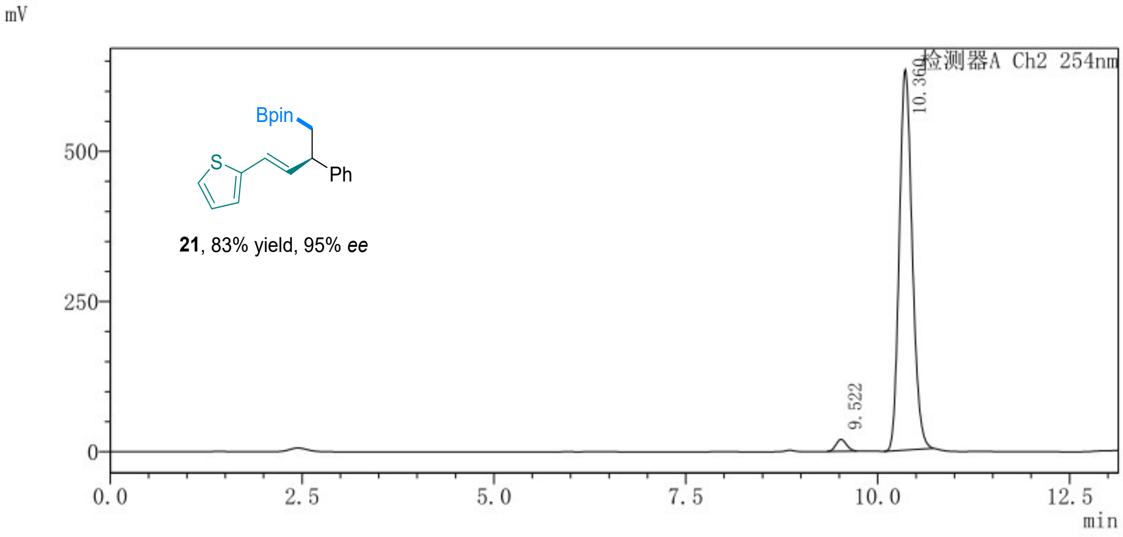

PDA Ch2 254nm

Peak#	Resolution Time	Area	Height	Area %	Height %
1	9. 522	191095	19875	2. 519	3. 044
2	10. 360	7395228	633122	97. 481	96. 956
Total		7586323	652997	100. 000	100. 000

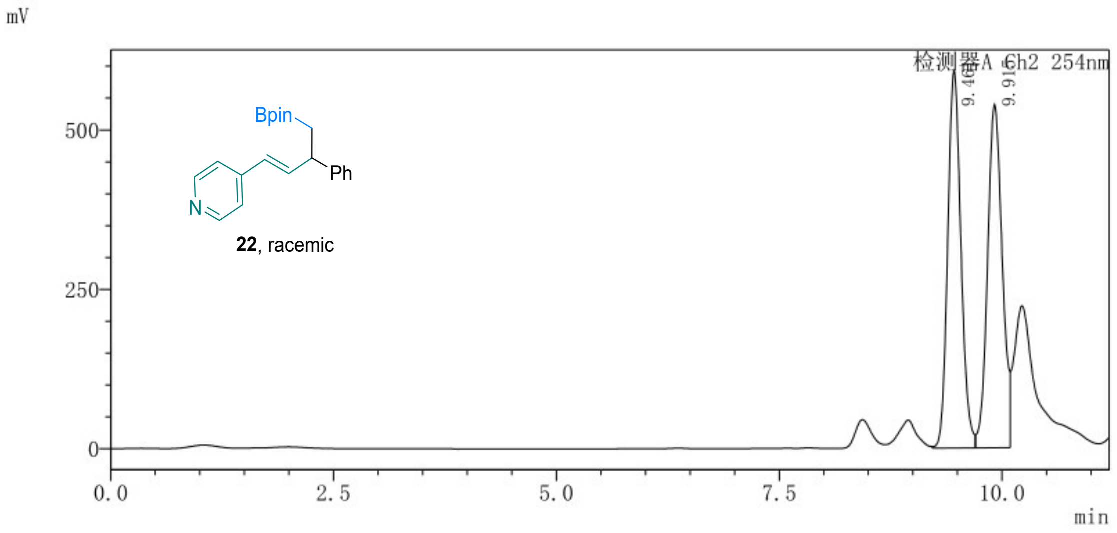

PDA Ch2 254nm

Peak#	Resolution Time	Area	Height	Area %	Height %
1	9. 461	6107176	591485	49. 631	52. 330
2	9. 915	6197868	538803	50. 369	47. 670
Total		12305044	1130288	100. 000	100. 000

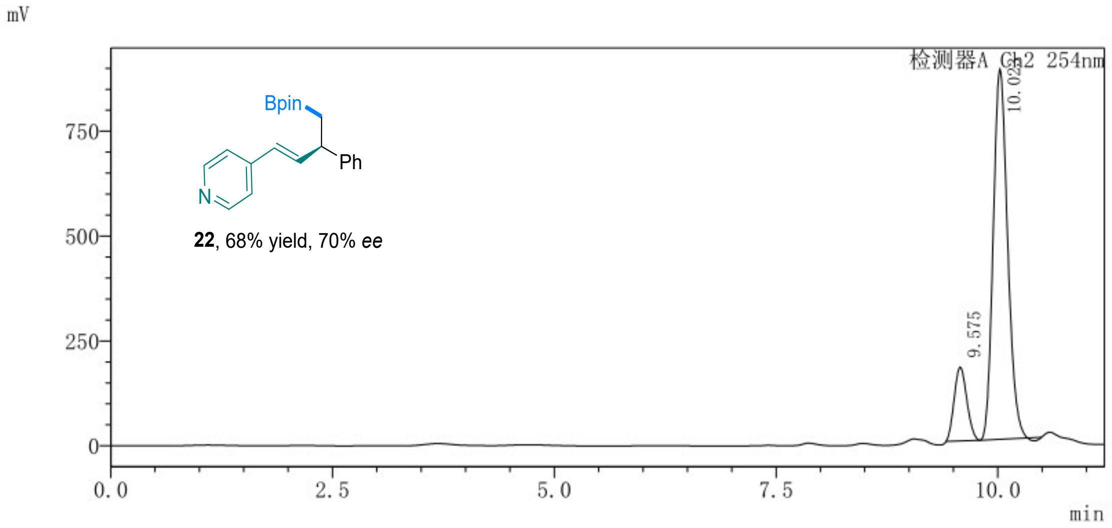

PDA Ch2 254nm

Peak#	Resolution Time	Area	Height	Area %	Height %
1	9. 575	1740986	175953	14. 916	16. 622
2	10. 023	9931177	882577	85. 084	83. 378
Total		11672163	1058530	100. 000	100. 000

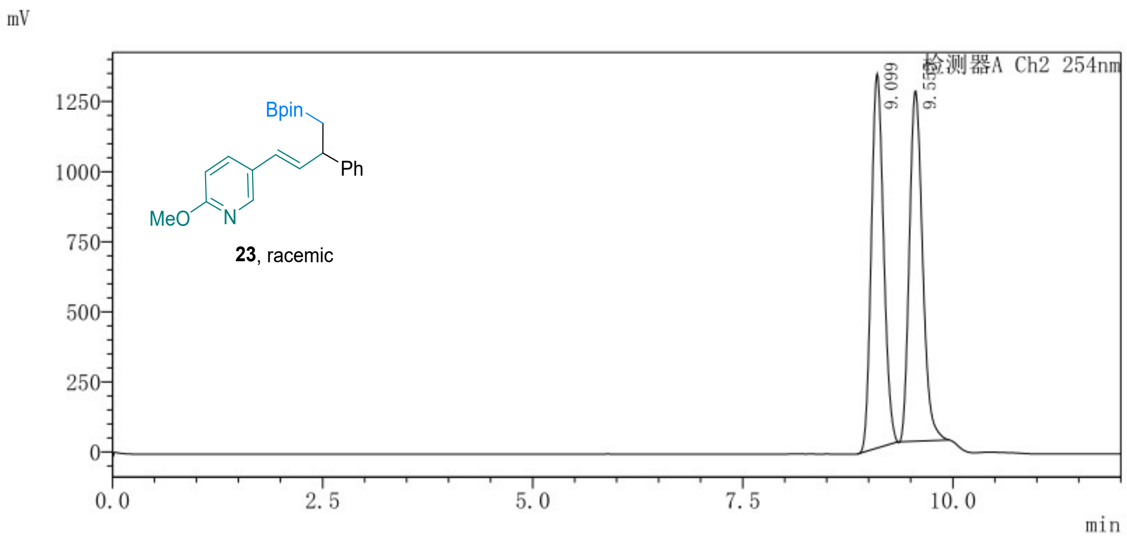

PDA Ch2 254nm

Peak#	Resolution Time	Area	Height	Area %	Height %
1	9.099	13609527	1334911	50.186	51.662
2	9.555	13508680	1249026	49.814	48.338
Total		27118207	2583937	100.000	100.000

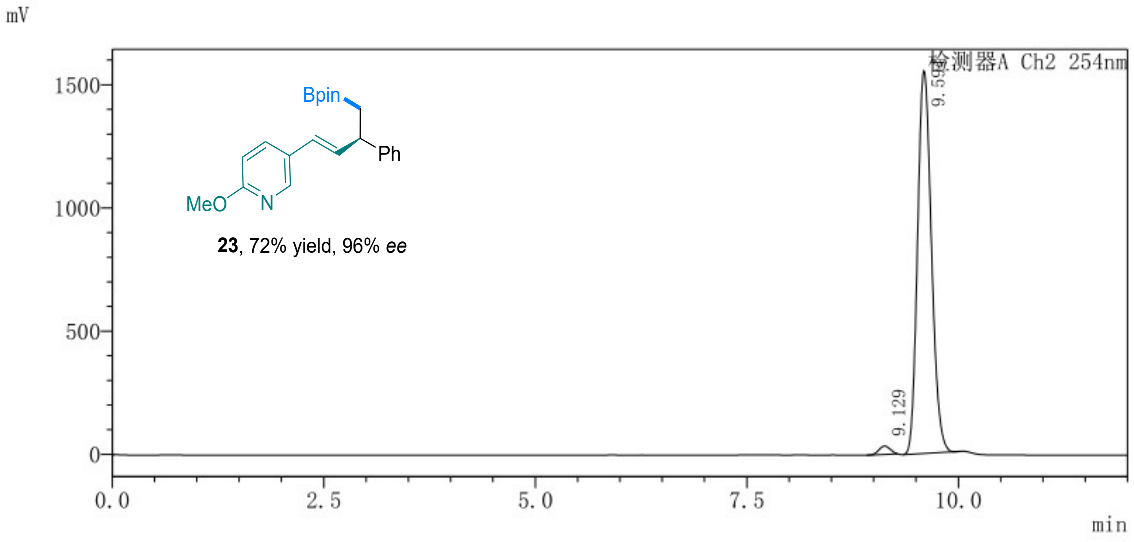

PDA Ch2 254nm

Peak#	Resolution Time	Area	Height	Area %	Height %
1	9.129	319340	34309	1.788	2.162
2	9.594	17542385	1552759	98.212	97.838
Total		17861725	1587068	100.000	100.000

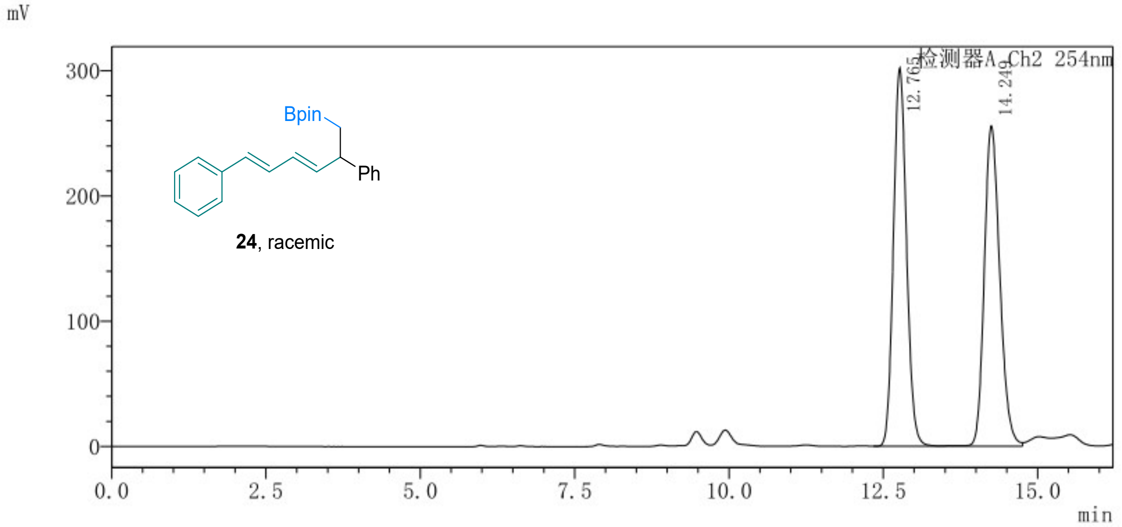

PDA Ch2 254nm

Peak#	Resolution Time	Area	Height	Area %	Height %
1	12.765	4453821	302316	49.948	54.154
2	14.249	4463034	255936	50.052	45.846
Total		8916855	558252	100.000	100.000

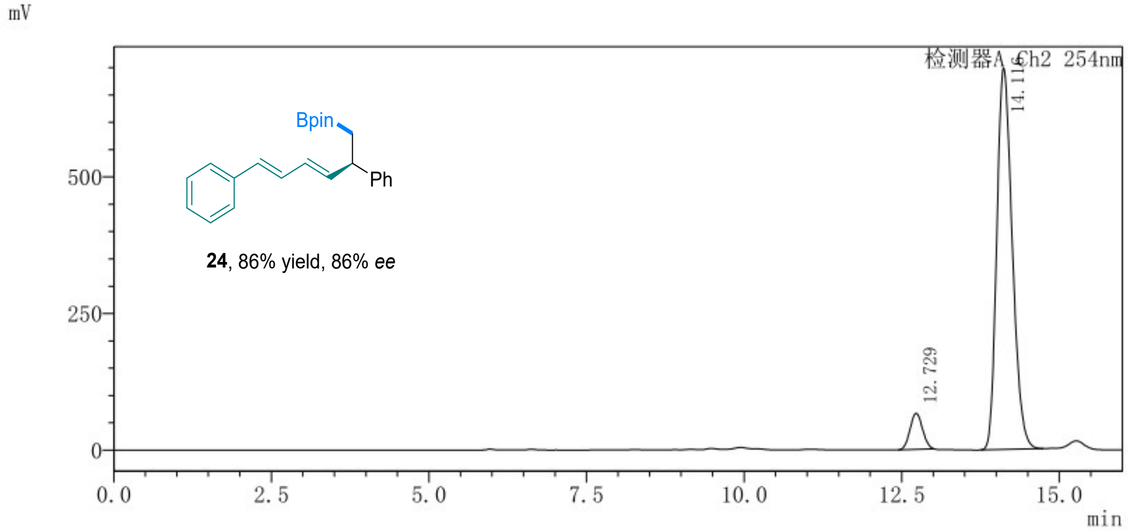

PDA Ch2 254nm

Peak#	Resolution Time	Area	Height	Area %	Height %
1	12.729	916440	66416	7.124	8.685
2	14.116	11947556	698315	92.876	91.315
Total		12863996	764732	100.000	100.000

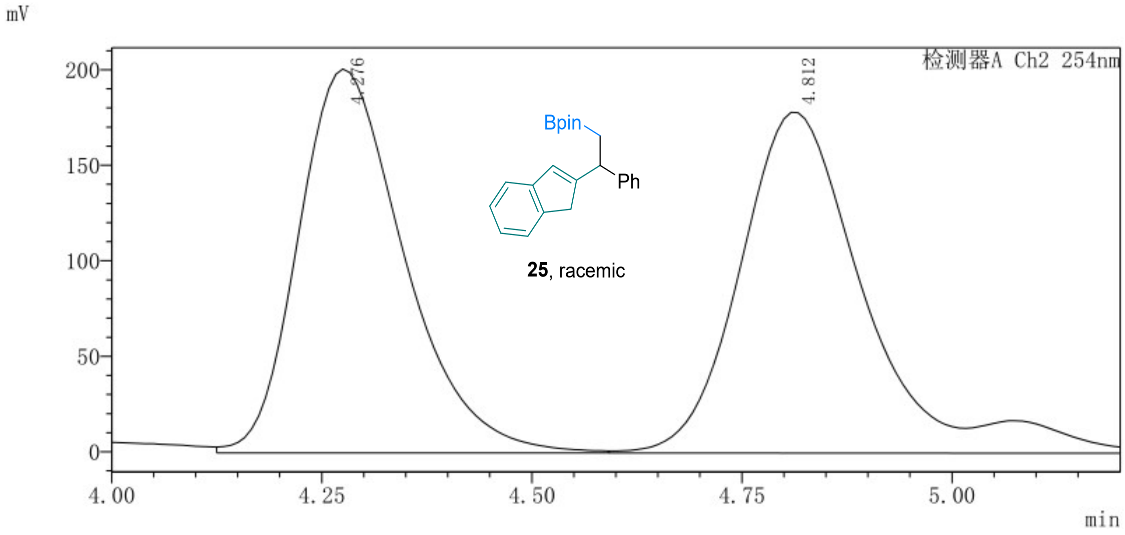

PDA Ch2 254nm

Peak#	Resolution Time	Area	Height	Area %	Height %
1	4. 276	1758894	200924	48. 558	52. 985
2	4. 812	1863379	178284	51. 442	47. 015
Total		3622274	379208	100. 000	100. 000

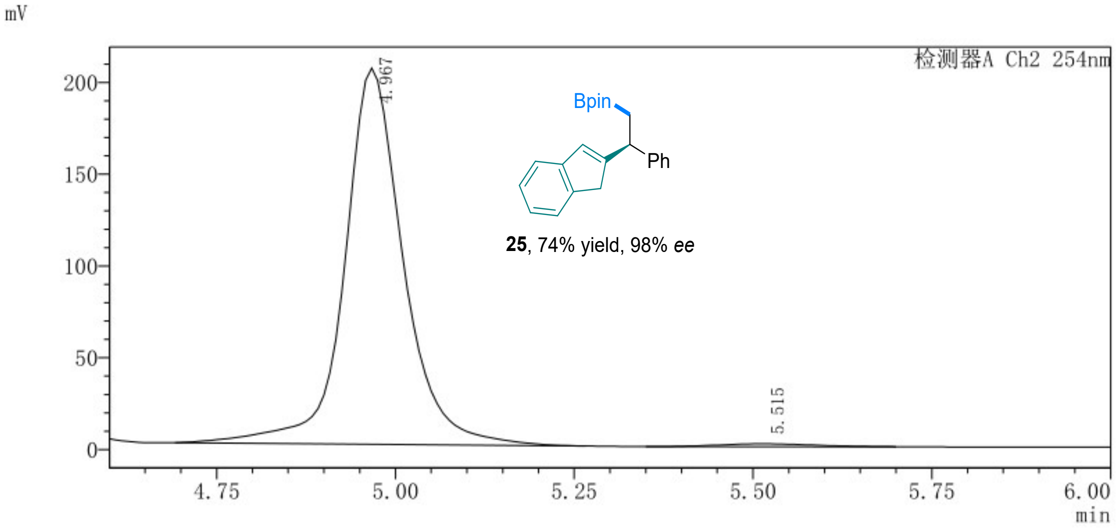

PDA Ch2 254nm

Peak#	Resolution Time	Area	Height	Area %	Height %
1	4. 967	1171724	204840	98. 811	99. 261
2	5. 515	14103	1526	1. 189	0. 739
Total		1185827	206366	100. 000	100. 000

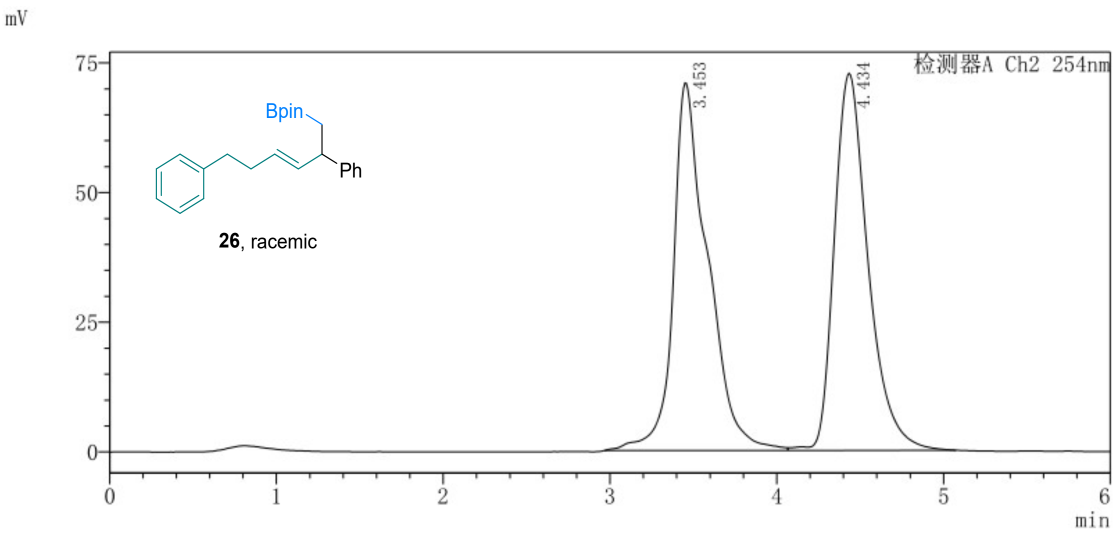

PDA Ch2 254nm

Peak#	Resolution Time	Area	Height	Area %	Height %
1	3. 453	1019388	70833	49. 880	49. 353
2	4. 434	1024274	72689	50. 120	50. 647
Total		2043662	143522	100. 000	100. 000

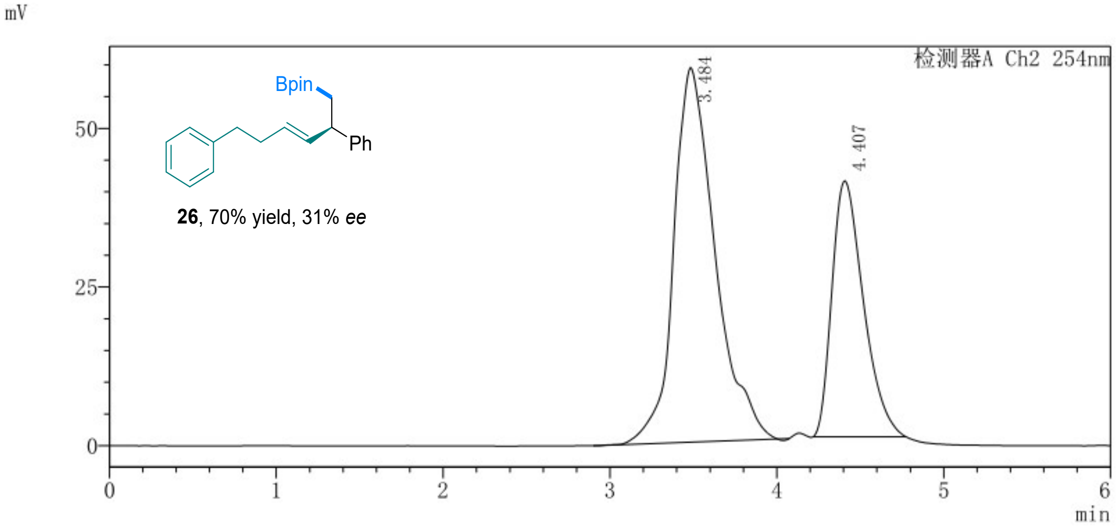

PDA Ch2 254nm

Peak#	Resolution Time	Area	Height	Area %	Height %
1	3. 484	1014445	59040	65. 698	59. 396
2	4. 407	529650	40360	34. 302	40. 604
Total		1544095	99401	100. 000	100. 000

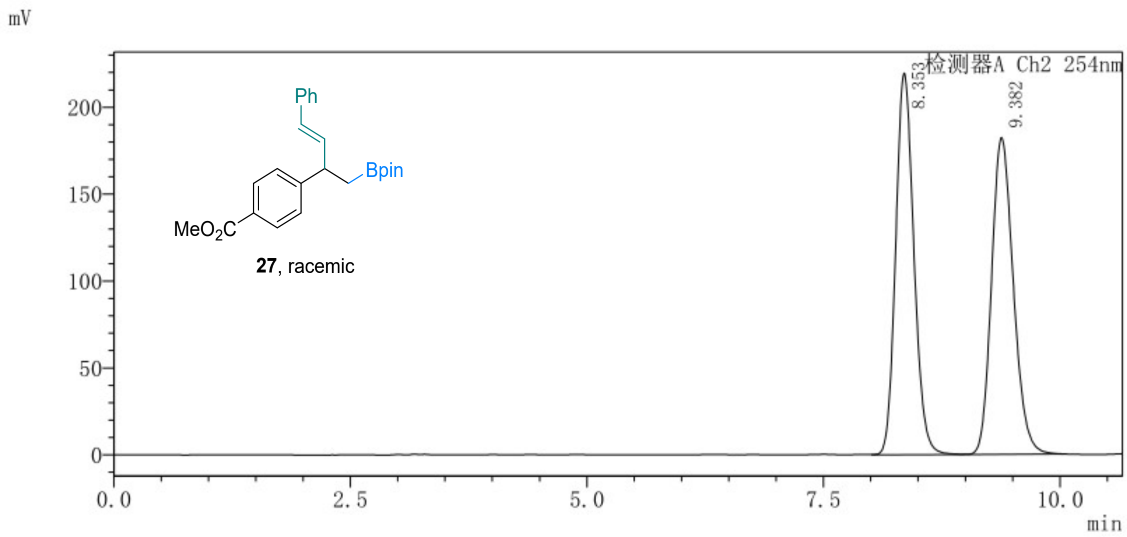

PDA Ch2 254nm

Peak#	Resolution Time	Area	Height	Area %	Height %
1	8.353	2935746	219426	50.100	54.624
2	9.382	2924043	182275	49.900	45.376
Total		5859789	401701	100.000	100.000

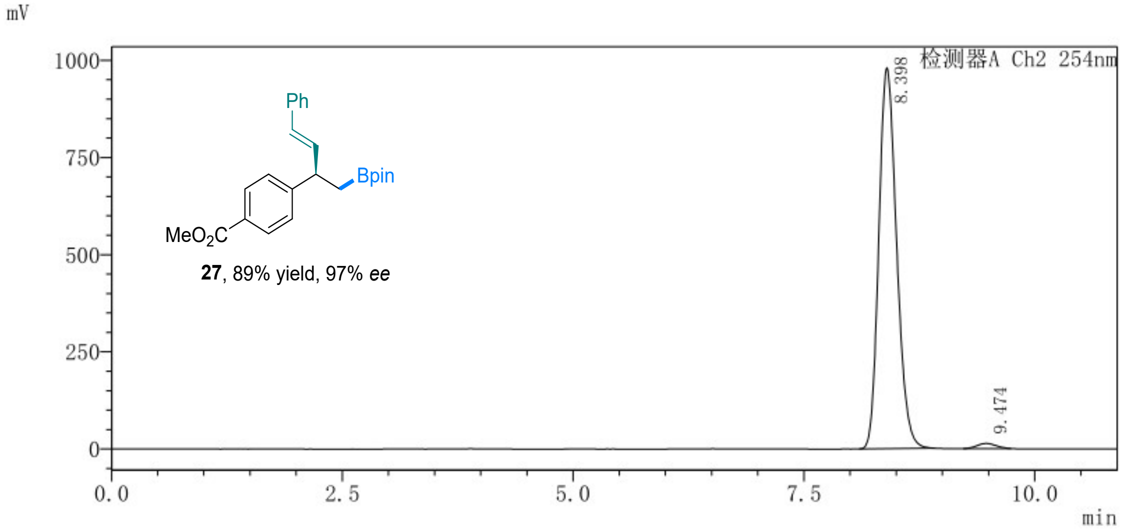

PDA Ch2 254nm

Peak#	Resolution Time	Area	Height	Area %	Height %
1	8.398	13134897	979437	98.552	98.666
2	9.474	193017	13245	1.448	1.334
Total		13327915	992682	100.000	100.000

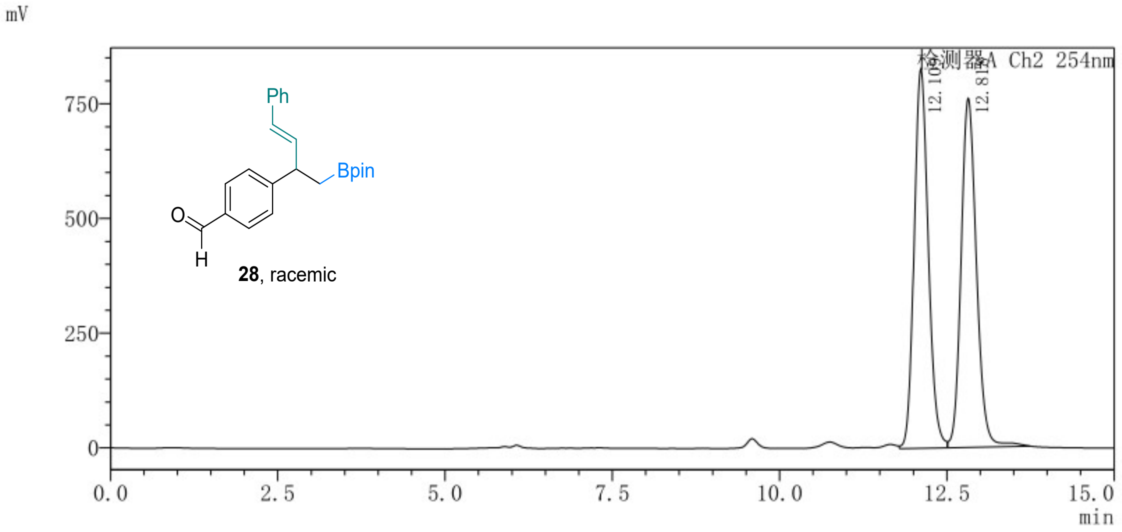

PDA Ch2 254nm

Peak#	Resolution Time	Area	Height	Area %	Height %
1	12.109	12097070	826554	49.862	52.064
2	12.818	12164191	761034	50.138	47.936
Total		24261261	1587589	100.000	100.000

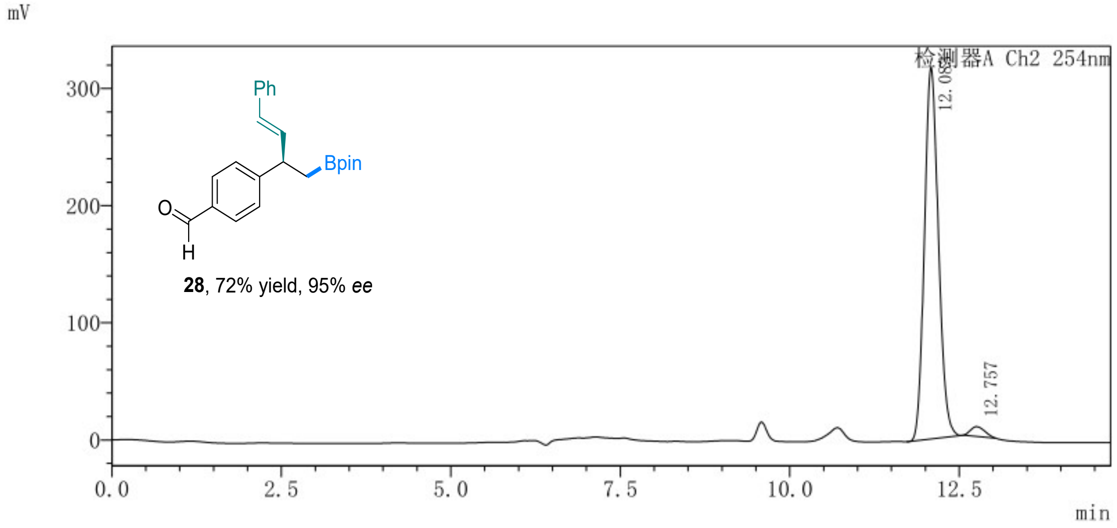

PDA Ch2 254nm

Peak#	Resolution Time	Area	Height	Area %	Height %
1	12.083	4547876	317320	97.411	97.465
2	12.757	120851	8254	2.589	2.535
Total		4668727	325573	100.000	100.000

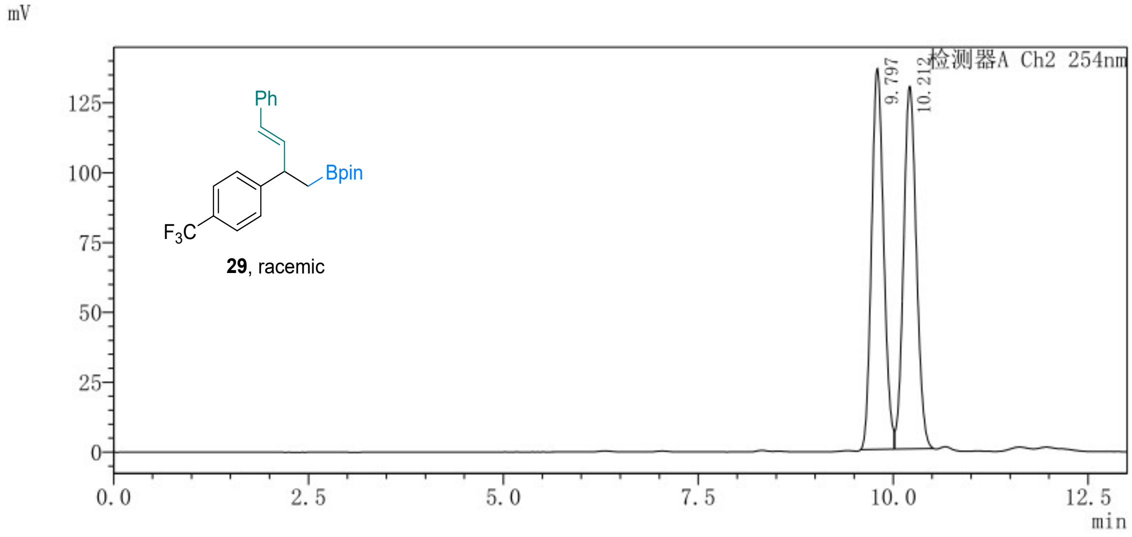

PDA Ch2 254nm

Peak#	Resolution Time	Area	Height	Area %	Height %
1	9.797	1473180	136301	49.823	51.237
2	10.212	1483658	129720	50.177	48.763
Total		2956838	266021	100.000	100.000

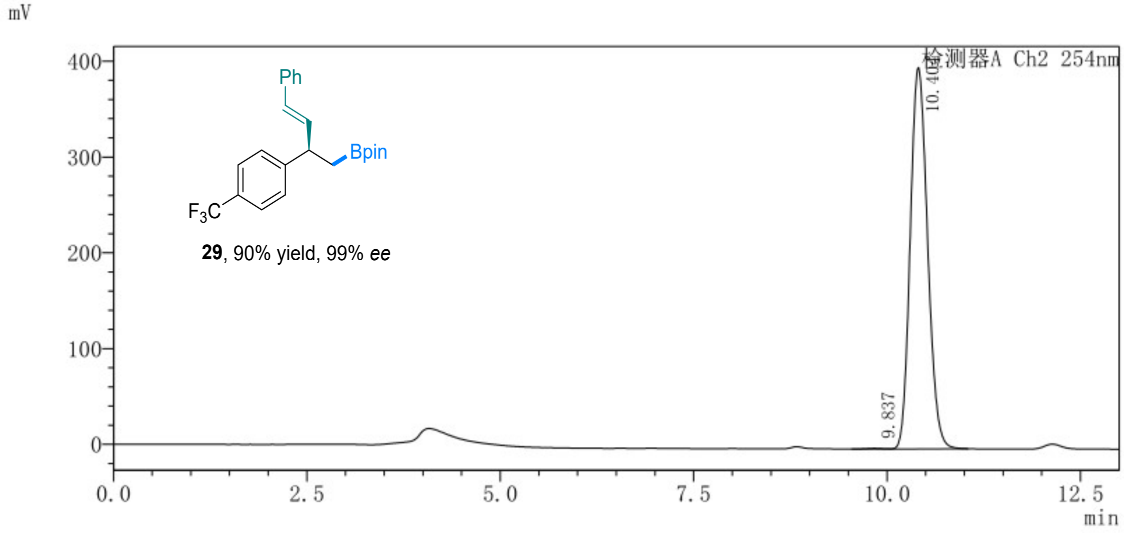

PDA Ch2 254nm

Peak#	Resolution Time	Area	Height	Area %	Height %
1	9.837	7644	636	0.123	0.160
2	10.404	6195807	397837	99.877	99.840
Total		6203450	398473	100.000	100.000

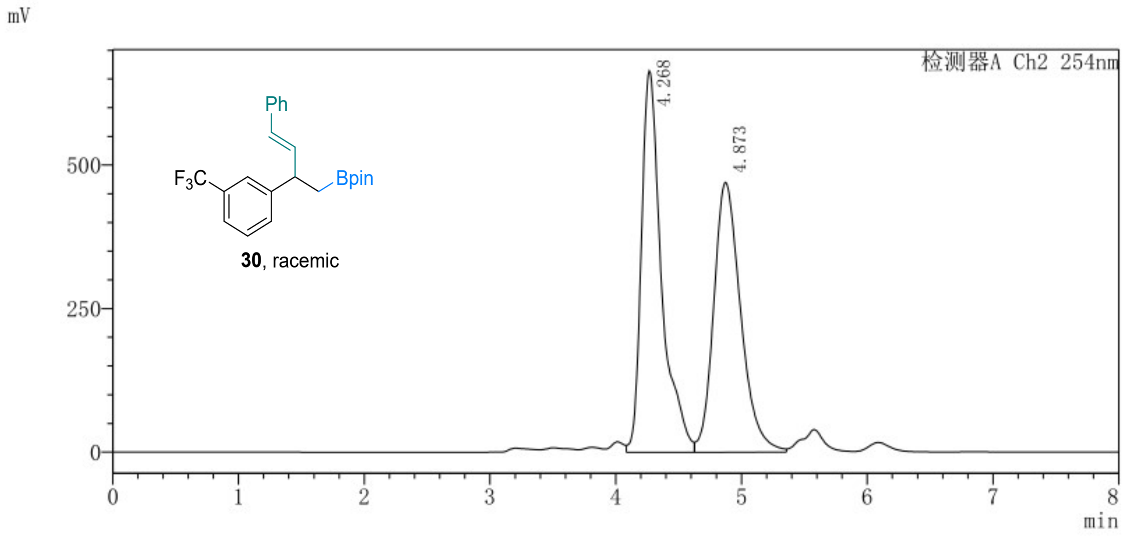

PDA Ch2 254nm

Peak#	Resolution Time	Area	Height	Area %	Height %
1	4.268	7426112	664273	51.403	58.553
2	4.873	7020649	470204	48.597	41.447
Total		14446761	1134476	100.000	100.000

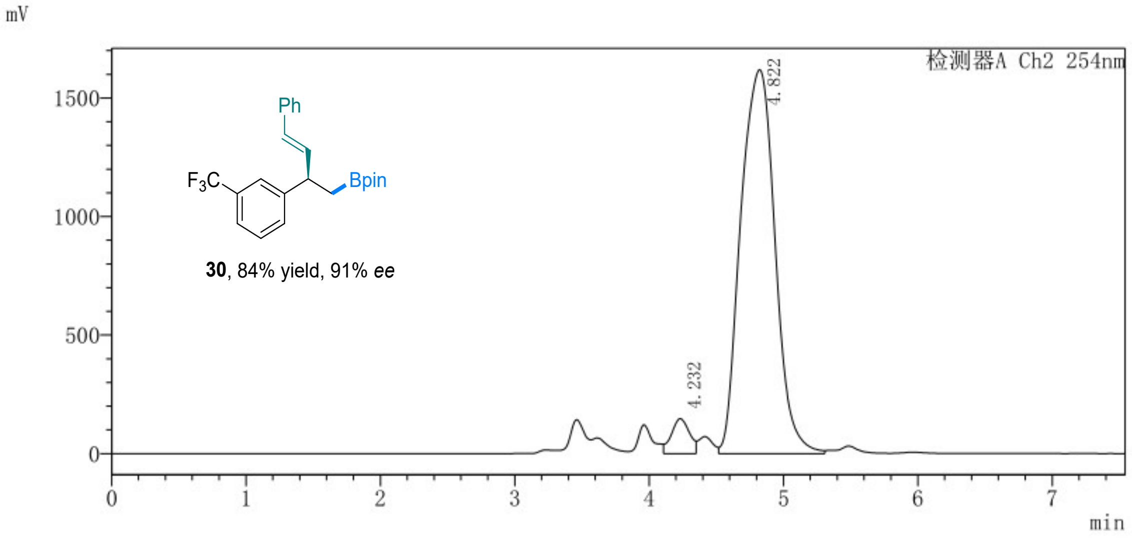

PDA Ch2 254nm

Peak#	Resolution Time	Area	Height	Area %	Height %
1	4.232	1376523	147370	4.583	8.341
2	4.822	28659838	1619464	95.417	91.659
Total		30036361	1766834	100.000	100.000

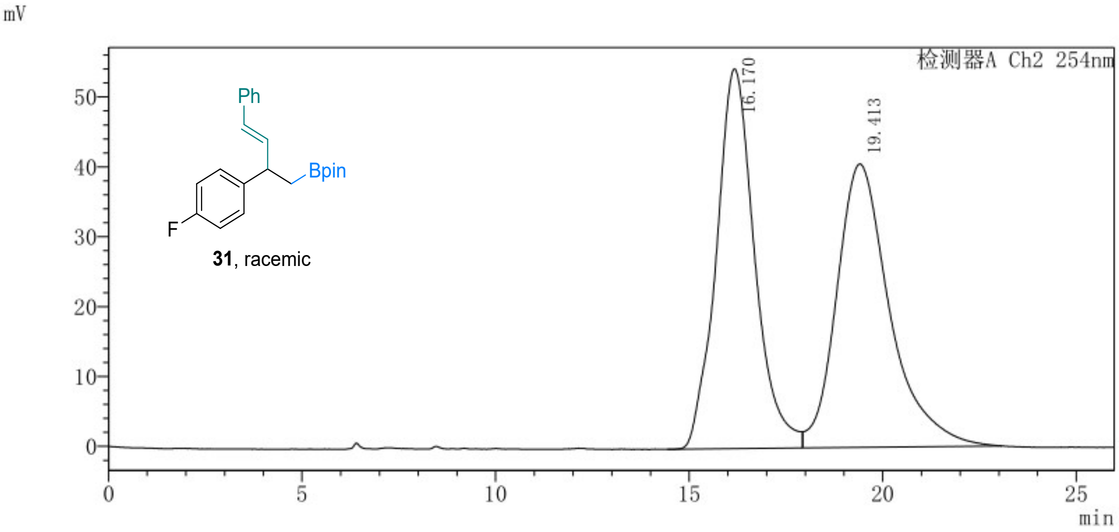

PDA Ch2 254nm

Peak#	Resolution Time	Area	Height	Area %	Height %
1	16. 170	3754271	54366	49. 277	57. 273
2	19. 413	3864419	40558	50. 723	42. 727
Total		7618690	94925	100. 000	100. 000

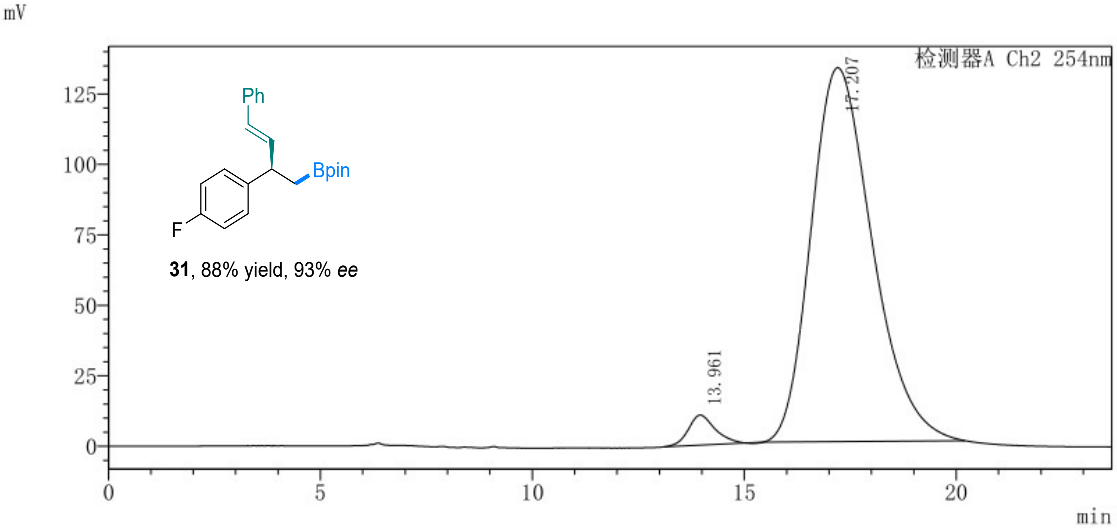

PDA Ch2 254nm

Peak#	Resolution Time	Area	Height	Area %	Height %
1	13. 961	467983	10699	3. 383	7. 461
2	17. 207	13364270	132700	96. 617	92. 539
Total		13832252	143399	100. 000	100. 000

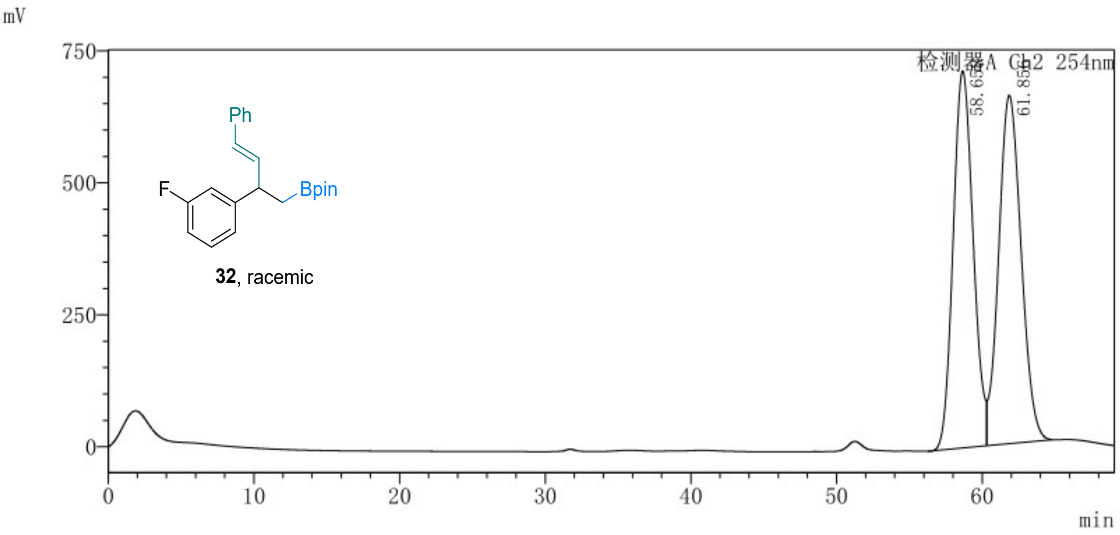

PDA Ch2 254nm

Peak#	Resolution Time	Area	Height	Area %	Height %
1	58.658	68146188	714064	49.020	51.975
2	61.856	70872198	659788	50.980	48.025
Total		139018386	1373853	100.000	100.000

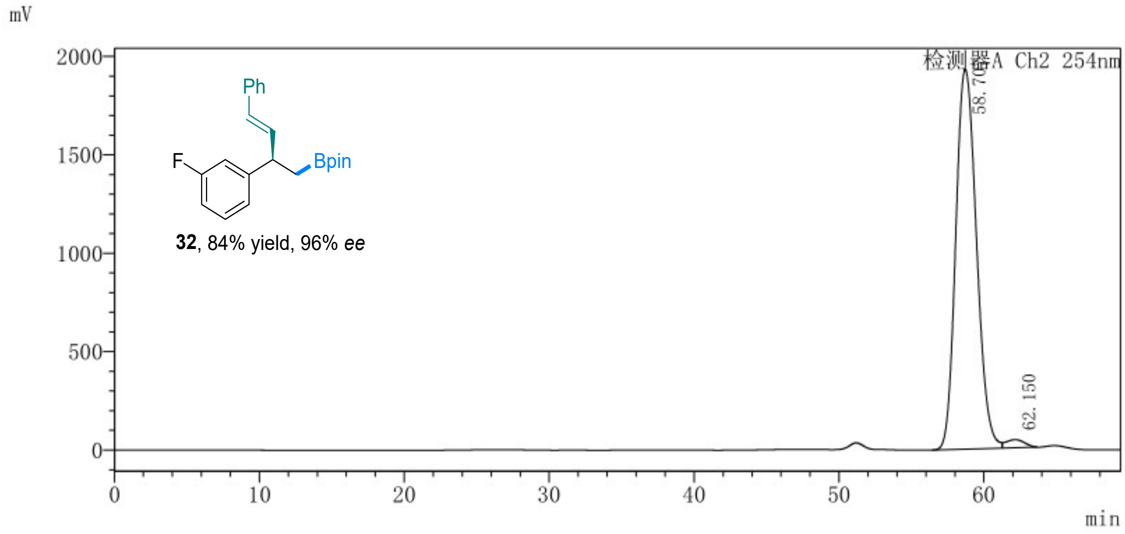

PDA Ch2 254nm

Peak#	Resolution Time	Area	Height	Area %	Height %
1	58.707	191309356	1928697	98.104	97.910
2	62.150	3697668	41175	1.896	2.090
Total		195007024	1969872	100.000	100.000

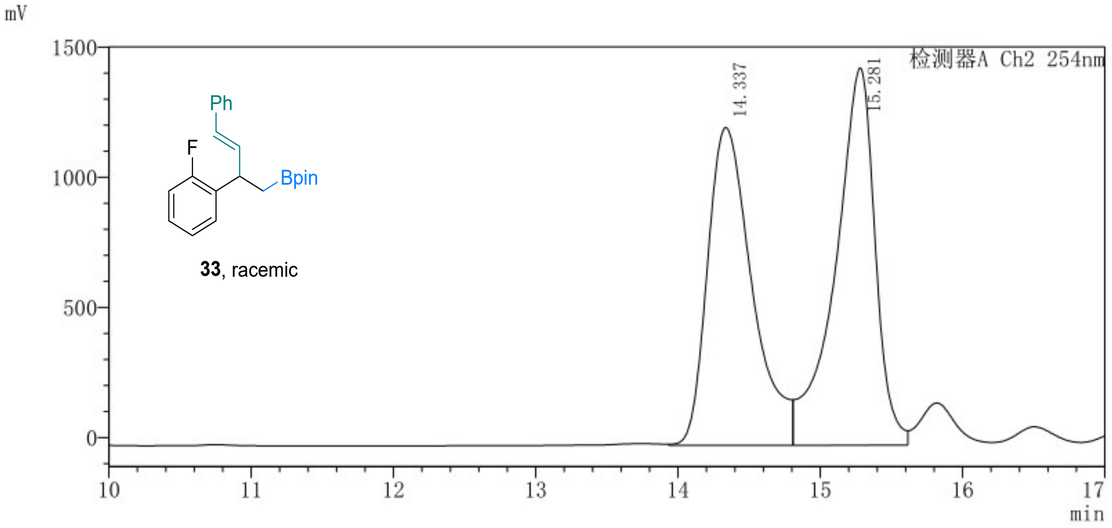

PDA Ch2 254nm

Peak#	Resolution Time	Area	Height	Area %	Height %
1	14.337	26608290	1220899	48.545	45.729
2	15.281	28203036	1448956	51.455	54.271
Total		54811326	2669855	100.000	100.000

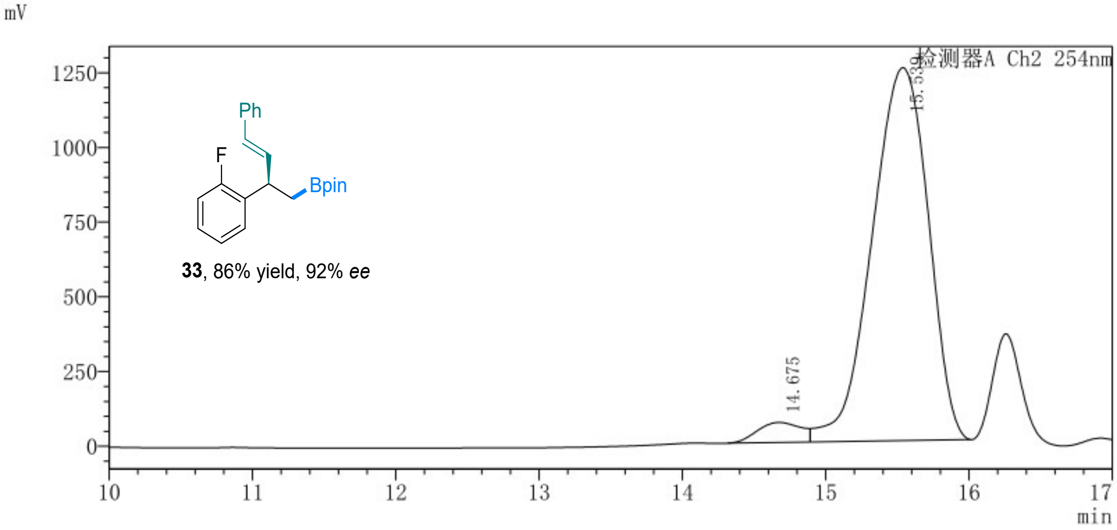

PDA Ch2 254nm

Peak#	Resolution Time	Area	Height	Area %	Height %
1	14.675	1436401	66873	4.046	5.085
2	15.539	34060977	1248146	95.954	94.915
Total		35497378	1315019	100.000	100.000

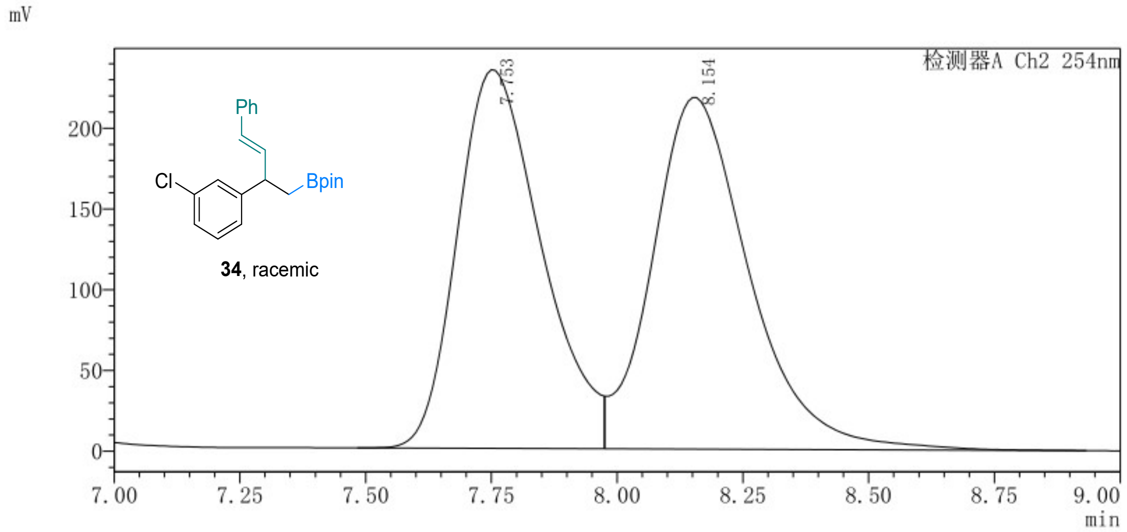

PDA Ch2 254nm

Peak#	Resolution Time	Area	Height	Area %	Height %
1	7.753	2735757	234435	48.438	51.853
2	8.154	2912166	217681	51.562	48.147
Total		5647922	452117	100.000	100.000

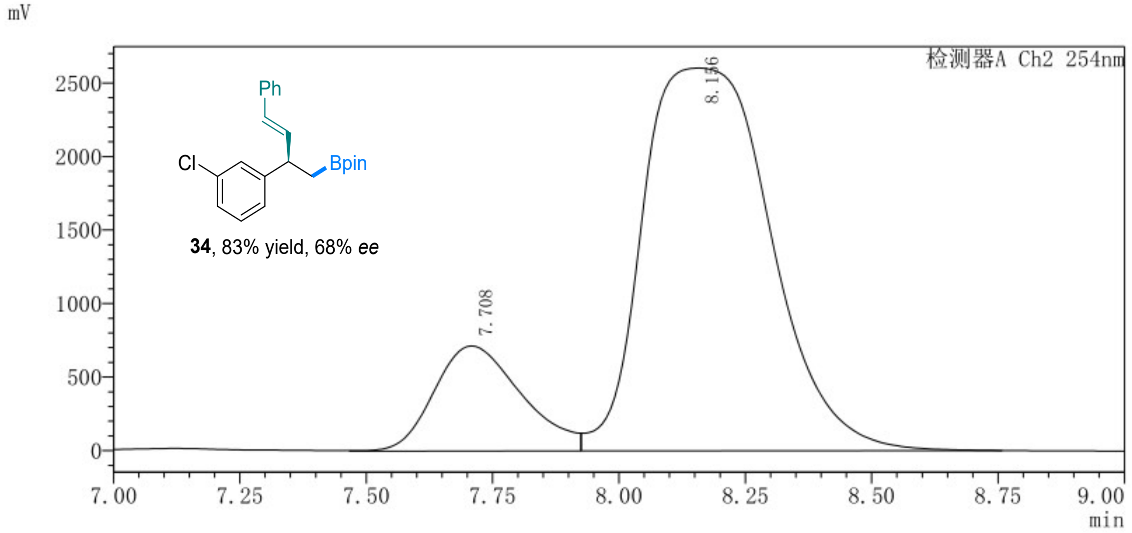

PDA Ch2 254nm

Peak#	Resolution Time	Area	Height	Area %	Height %
1	7.708	8636636	715810	15.918	21.567
2	8.156	45621327	2603235	84.082	78.433
Total		54257963	3319044	100.000	100.000

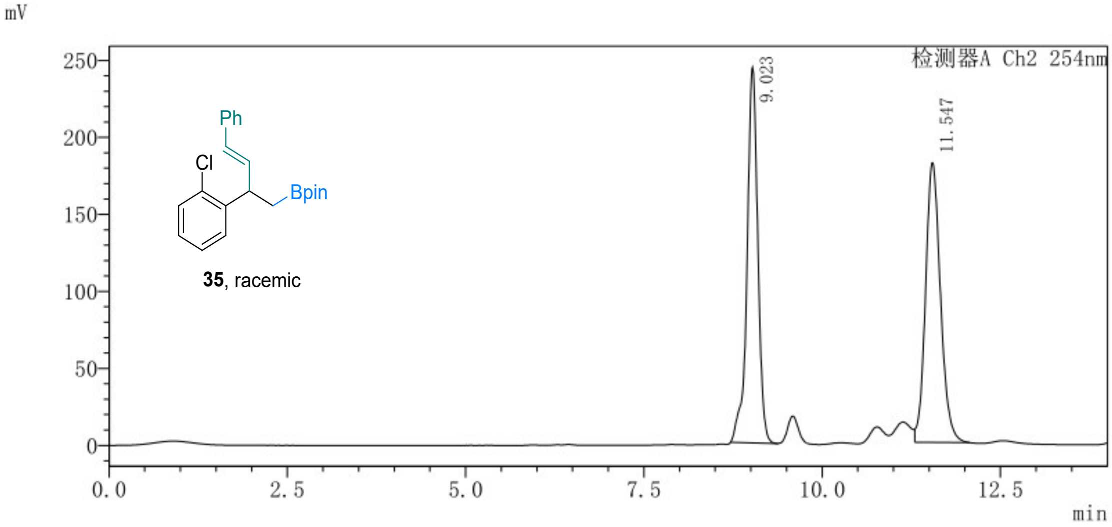

PDA Ch2 254nm

Peak#	Resolution Time	Area	Height	Area %	Height %
1	9.023	2560640	243811	49.212	57.317
2	11.547	2642683	181565	50.788	42.683
Total		5203323	425376	100.000	100.000

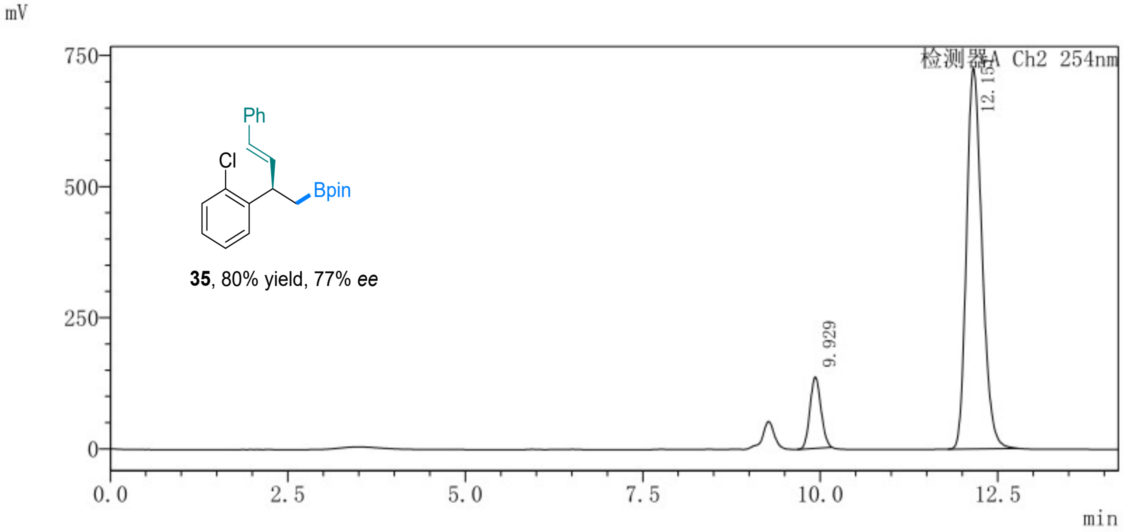

PDA Ch2 254nm

Peak#	Resolution Time	Area	Height	Area %	Height %
1	9.929	1469691	135677	11.761	15.734
2	12.157	11026629	726651	88.239	84.266
Total		12496320	862329	100.000	100.000

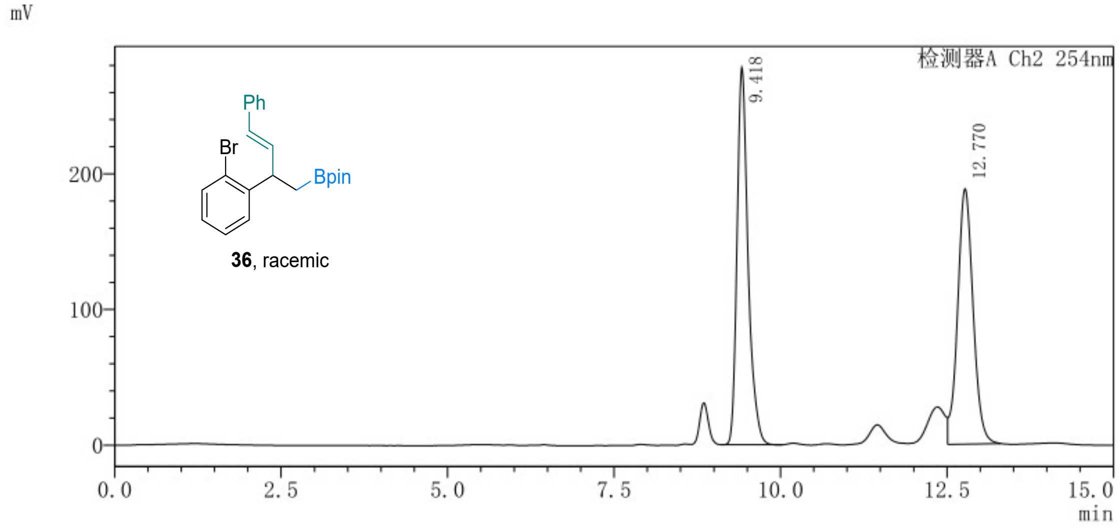

PDA Ch2 254nm

Peak#	Resolution Time	Area	Height	Area %	Height %
1	9.418	3207499	278109	51.350	59.638
2	12.770	3038897	188220	48.650	40.362
Total		6246396	466329	100.000	100.000

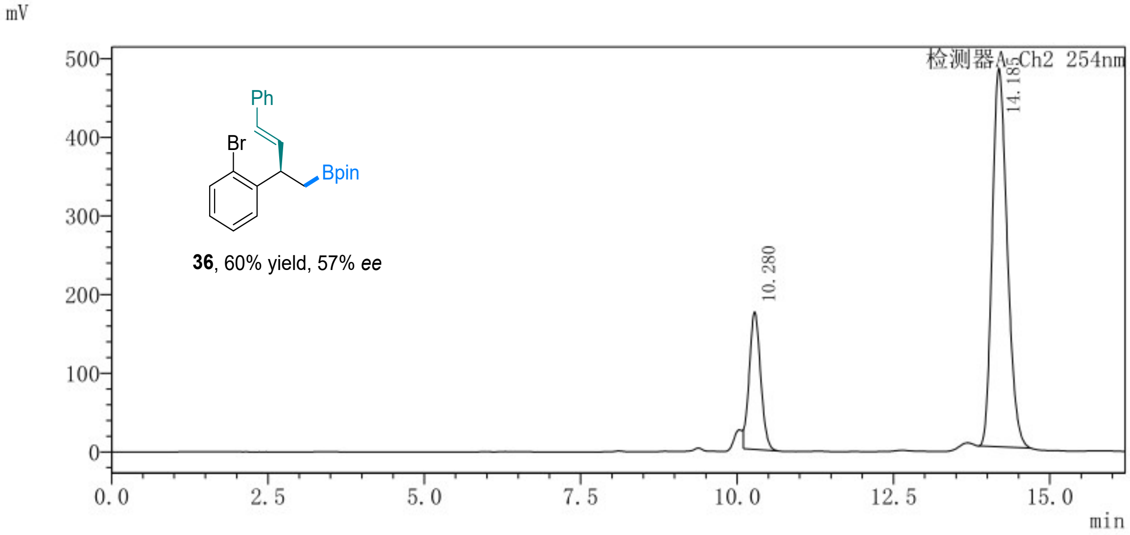

PDA Ch2 254nm

Peak#	Resolution Time	Area	Height	Area %	Height %
1	10.280	2214416	174677	21.627	26.629
2	14.185	8024484	481295	78.373	73.371
Total		10238900	655972	100.000	100.000

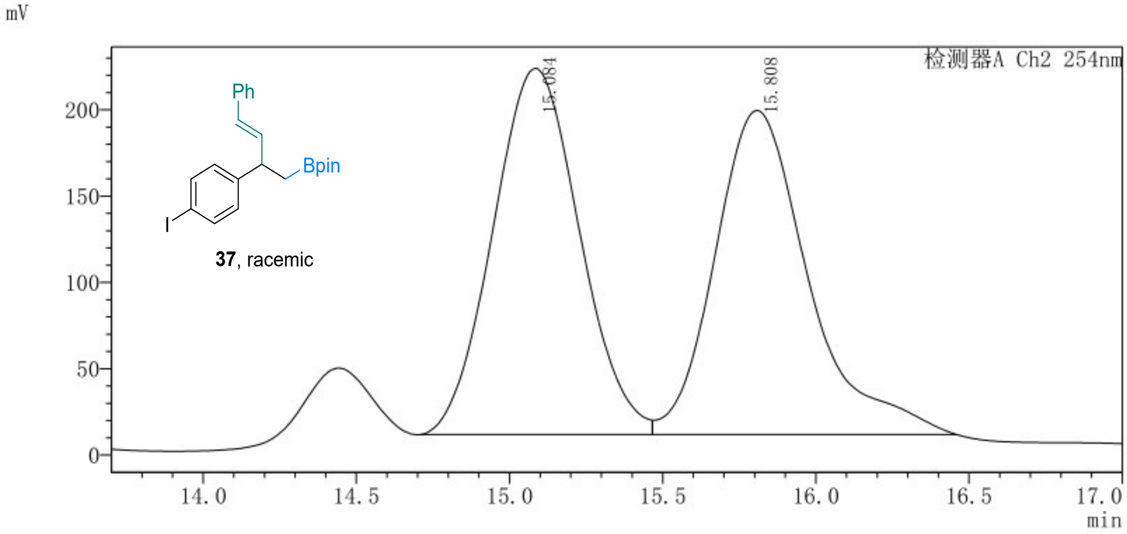

PDA Ch2 254nm

Peak#	Resolution Time	Area	Height	Area %	Height %
1	15.084	4170816	212163	51.196	53.050
2	15.808	3975927	187764	48.804	46.950
Total		8146743	399926	100.000	100.000

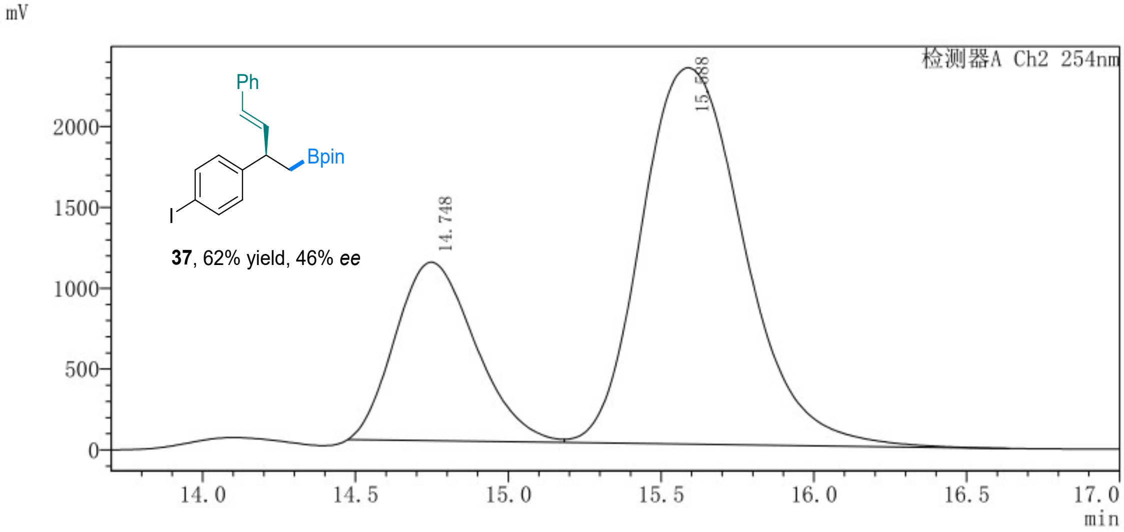

PDA Ch2 254nm

Peak#	Resolution Time	Area	Height	Area %	Height %
1	14.748	20158578	1104073	27.023	32.172
2	15.588	54438175	2327672	72.977	67.828
Total		74596753	3431745	100.000	100.000

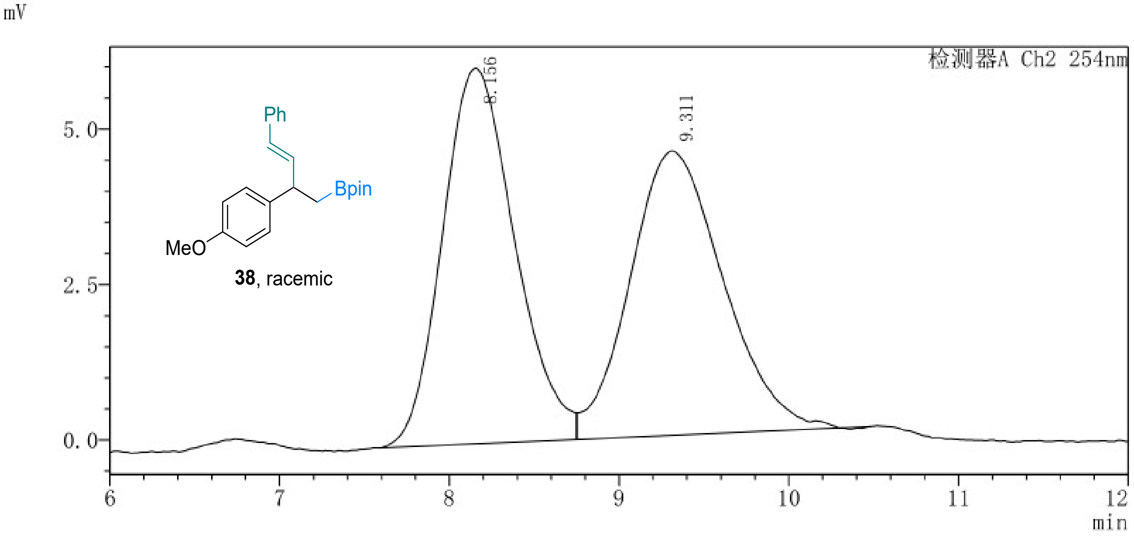

PDA Ch2 254nm

Peak#	Resolution Time	Area	Height	Area %	Height %
1	8.156	178849	6041	50.619	56.935
2	9.311	174478	4570	49.381	43.065
Total		353326	10611	100.000	100.000

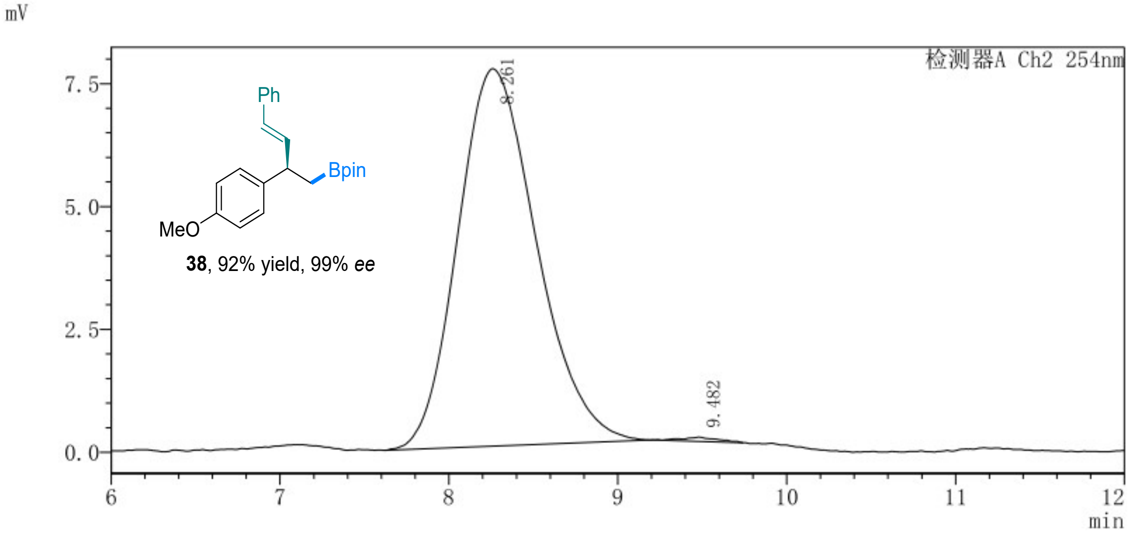

PDA Ch2 254nm

Peak#	Resolution Time	Area	Height	Area %	Height %
1	8.261	248888	7679	99.492	98.894
2	9.482	1272	86	0.508	1.106
Total		250159	7764	100.000	100.000

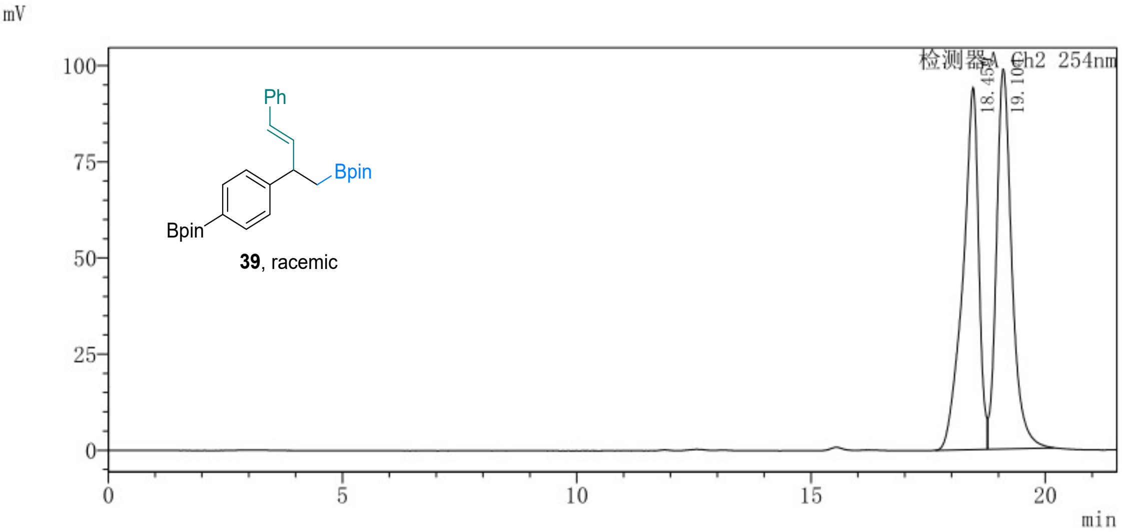

PDA Ch2 254nm

Peak#	Resolution Time	Area	Height	Area %	Height %
1	18. 459	2204116	94147	49. 532	48. 814
2	19. 104	2245737	98721	50. 468	51. 186
Total		4449853	192868	100. 000	100. 000

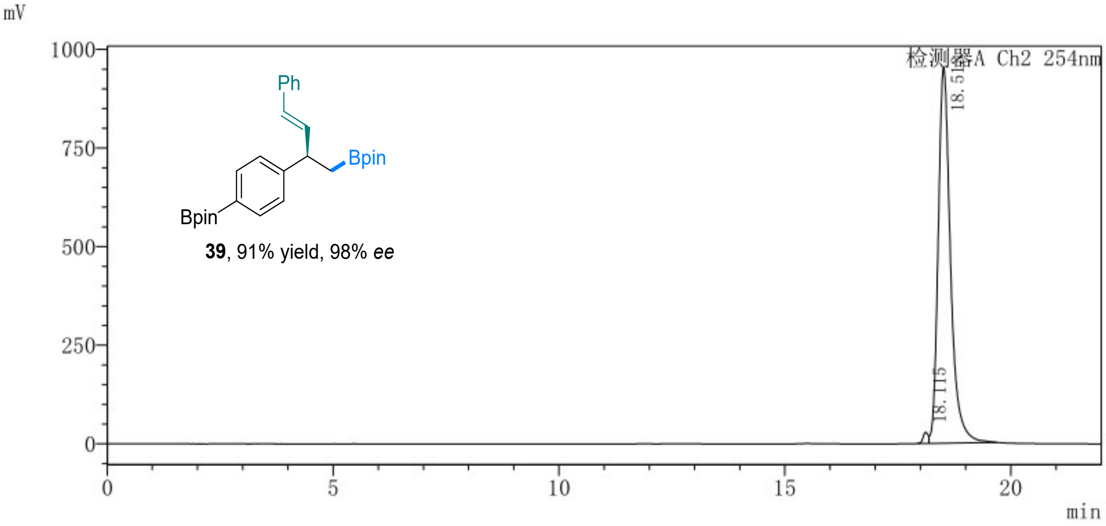

PDA Ch2 254nm

Peak#	Resolution Time	Area	Height	Area %	Height %
1	18. 115	225617	28172	1. 237	2. 871
2	18. 513	18013999	953185	98. 763	97. 129
Total		18239616	981357	100. 000	100. 000

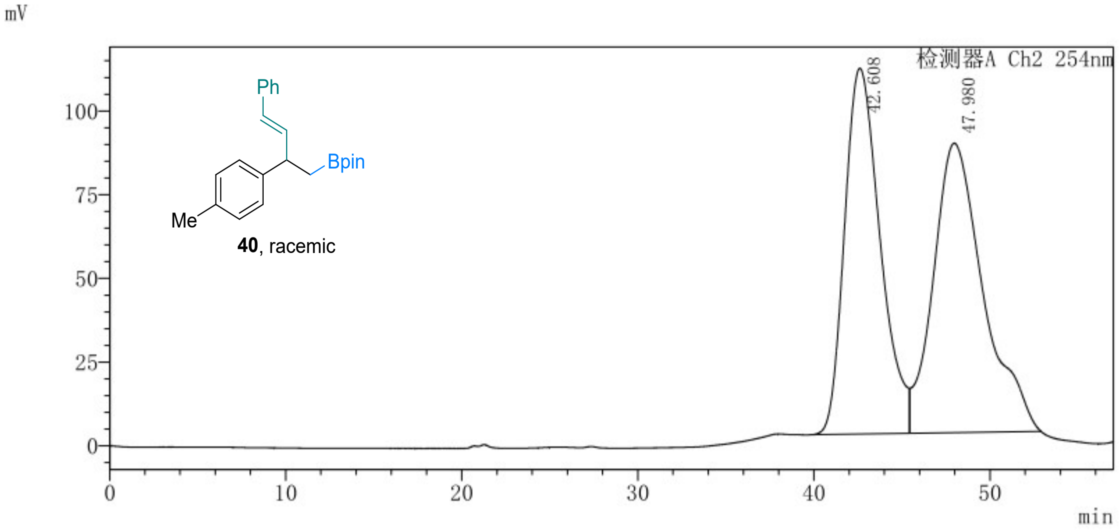

PDA Ch2 254nm

Peak#	Resolution Time	Area	Height	Area %	Height %
1	42. 608	15381357	109227	47. 459	55. 837
2	47. 980	17028341	86392	52. 541	44. 163
Total		32409698	195620	100. 000	100. 000

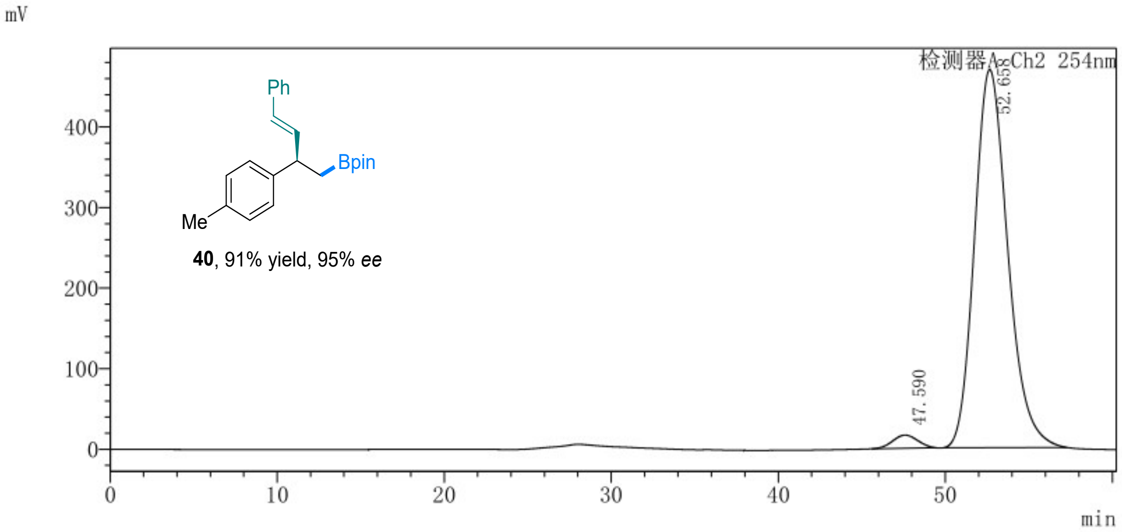

PDA Ch2 254nm

Peak#	Rsolution Time	Area	Height	Area %	Height %
1	47. 590	1687244	16263	2. 531	3. 350
2	52. 658	64977288	469205	97. 469	96. 650
Total		66664532	485468	100. 000	100. 000

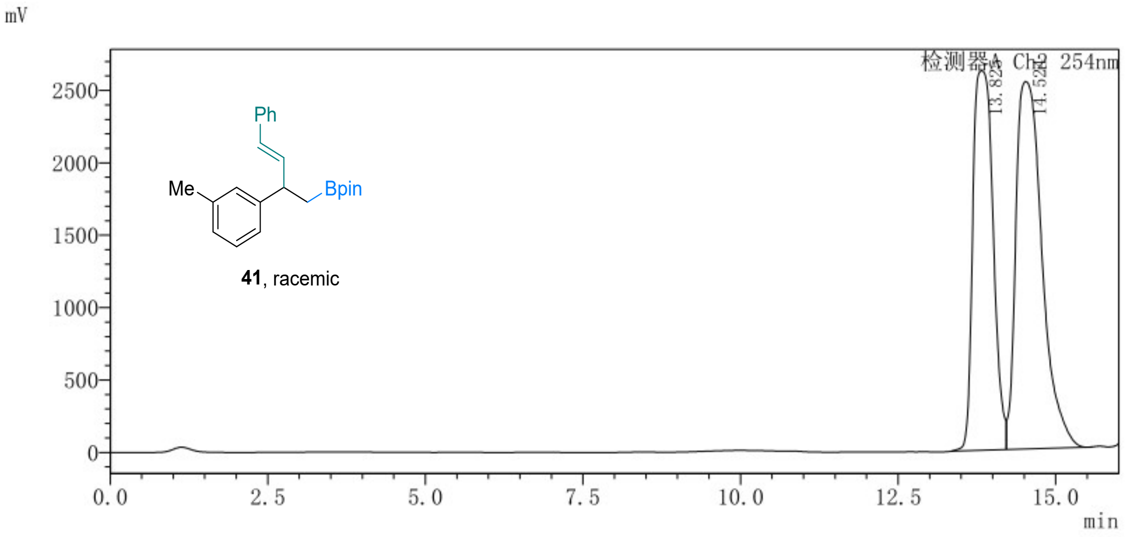

PDA Ch2 254nm

Peak#	Resolution Time	Area	Height	Area %	Height %
1	13.825	58543794	2621767	44.410	50.817
2	14.524	73282203	2537516	55.590	49.183
Total		131825997	5159282	100.000	100.000

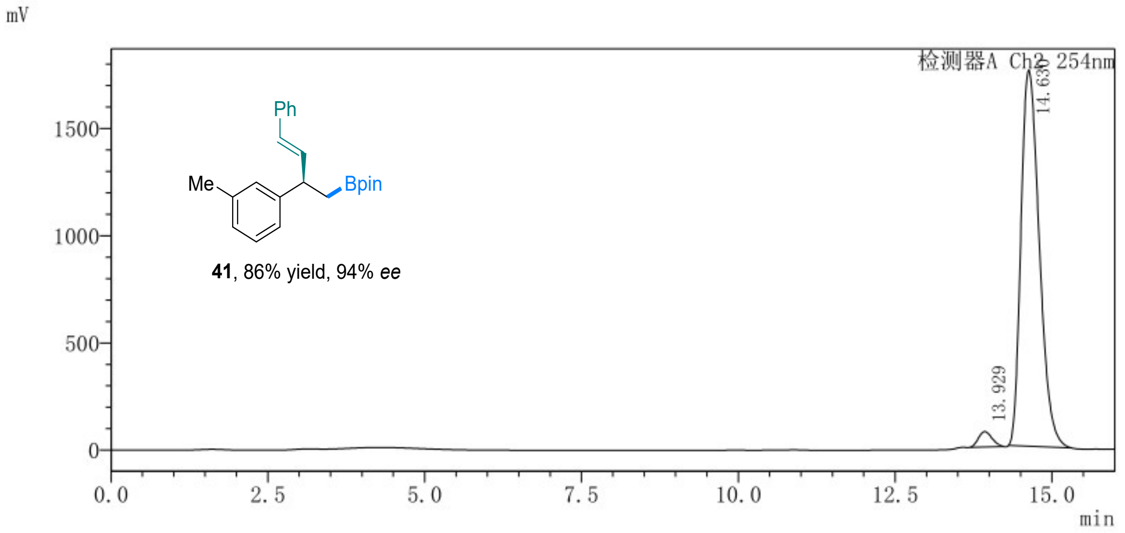

PDA Ch2 254nm

Peak#	Resolution Time	Area	Height	Area %	Height %
1	13.929	1074479	71616	2.927	3.924
2	14.630	35631674	1753400	97.073	96.076
Total		36706153	1825016	100.000	100.000

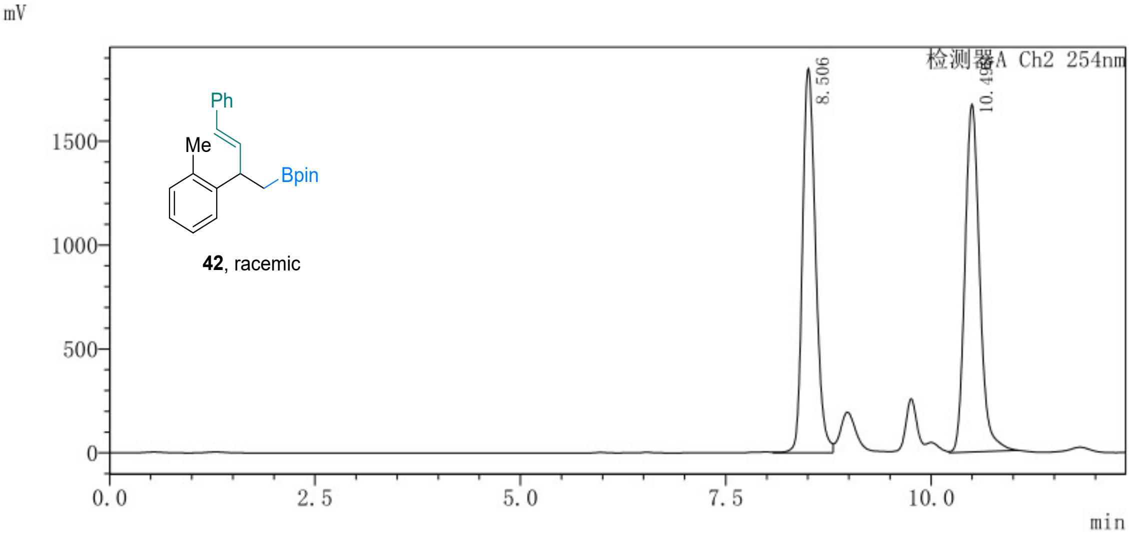

PDA Ch2 254nm

Peak#	Resolution Time	Area	Height	Area %	Height %
1	8.506	20411150	1848822	49.518	52.505
2	10.498	20808871	1672391	50.482	47.495
Total		41220021	3521214	100.000	100.000

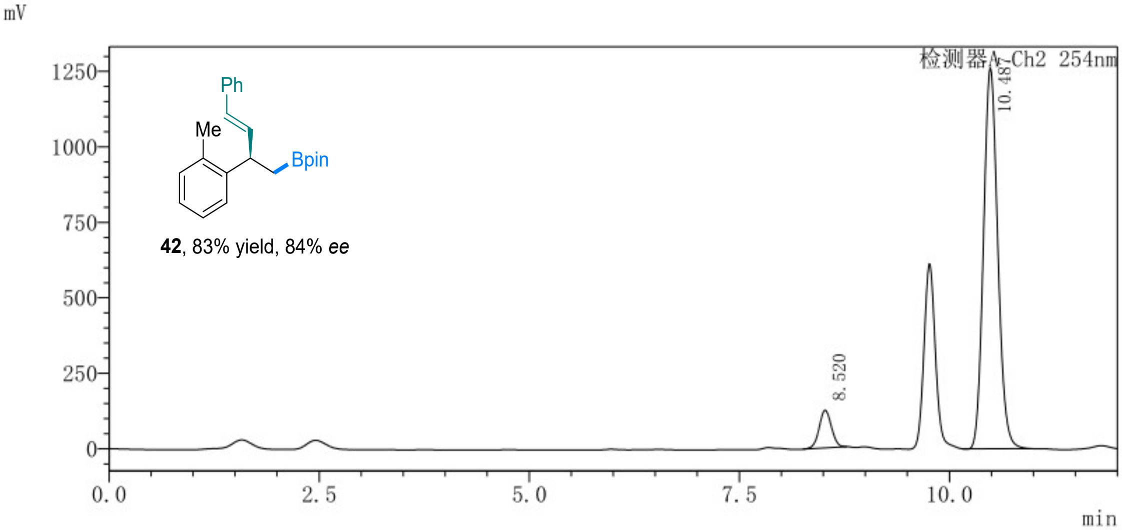

PDA Ch2 254nm

Peak#	Resolution Time	Area	Height	Area %	Height %
1	8.520	1276596	124851	7.888	9.007
2	10.487	14908274	1261334	92.112	90.993
Total		16184870	1386185	100.000	100.000

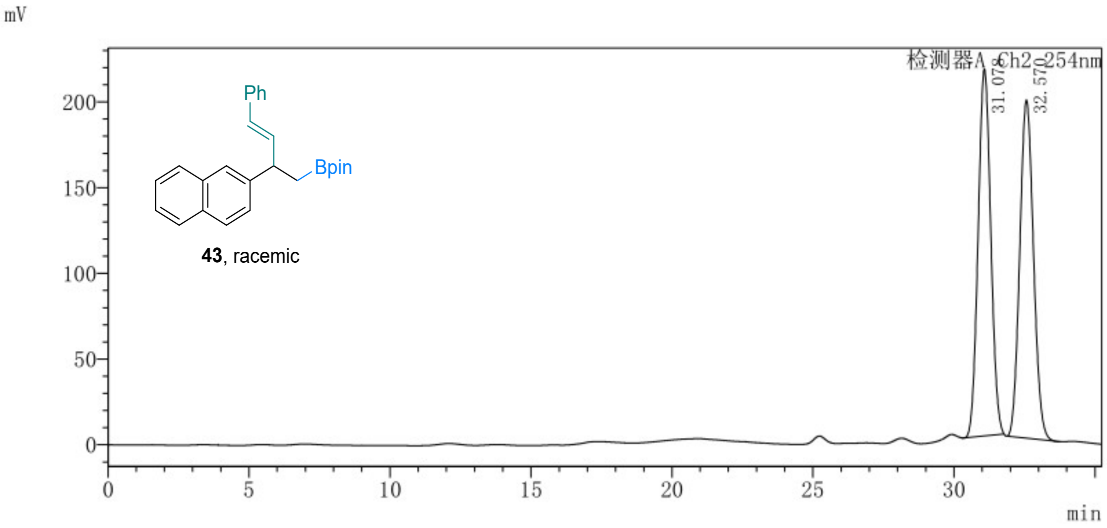

PDA Ch2 254nm

Peak#	Resolution Time	Area	Height	Area %	Height %
1	31.078	6659705	213887	49.935	52.032
2	32.570	6677147	197179	50.065	47.968
Total		13336852	411066	100.000	100.000

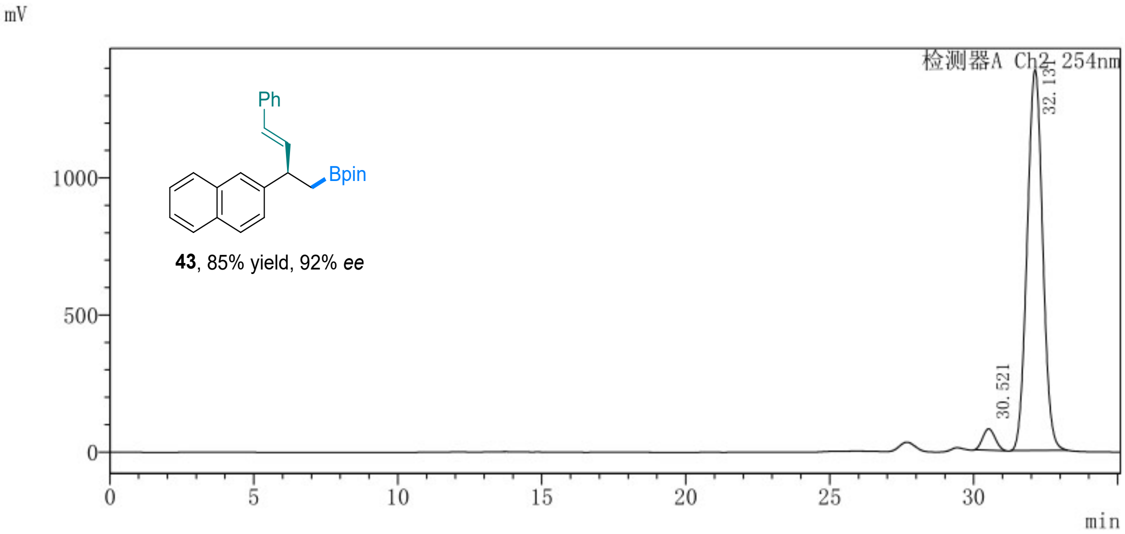

PDA Ch2 254nm

Peak#	Resolution Time	Area	Height	Area %	Height %
1	30.521	2327188	77592	4.279	5.292
2	32.131	52063040	1388693	95.721	94.708
Total		54390228	1466286	100.000	100.000

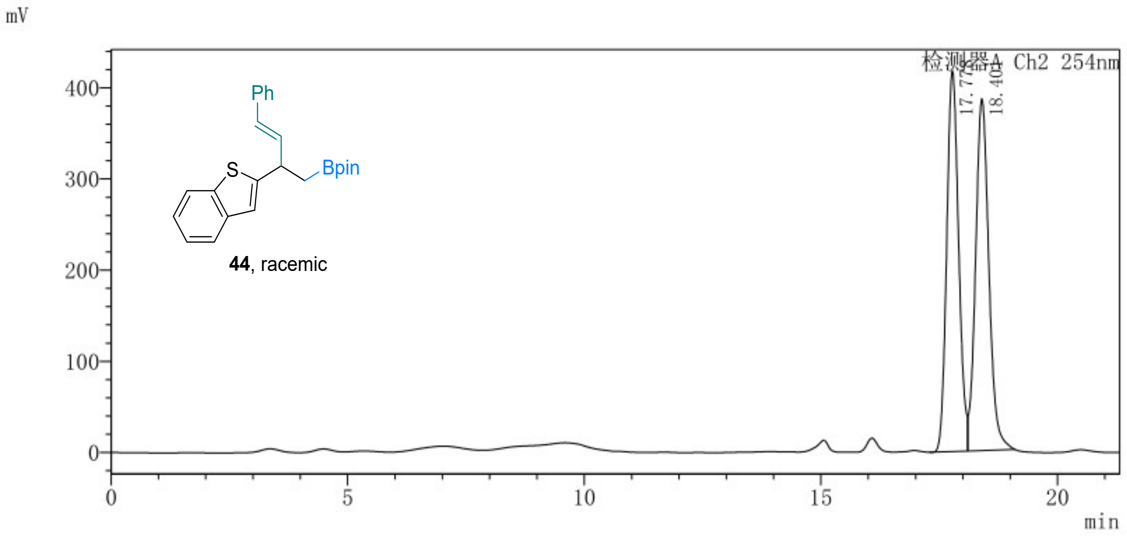

PDA Ch2 254nm

Peak#	Resolution Time	Area	Height	Area %	Height %
1	17.778	7304735	417480	49.177	51.975
2	18.401	7549211	385756	50.823	48.025
Total		14853946	803236	100.000	100.000

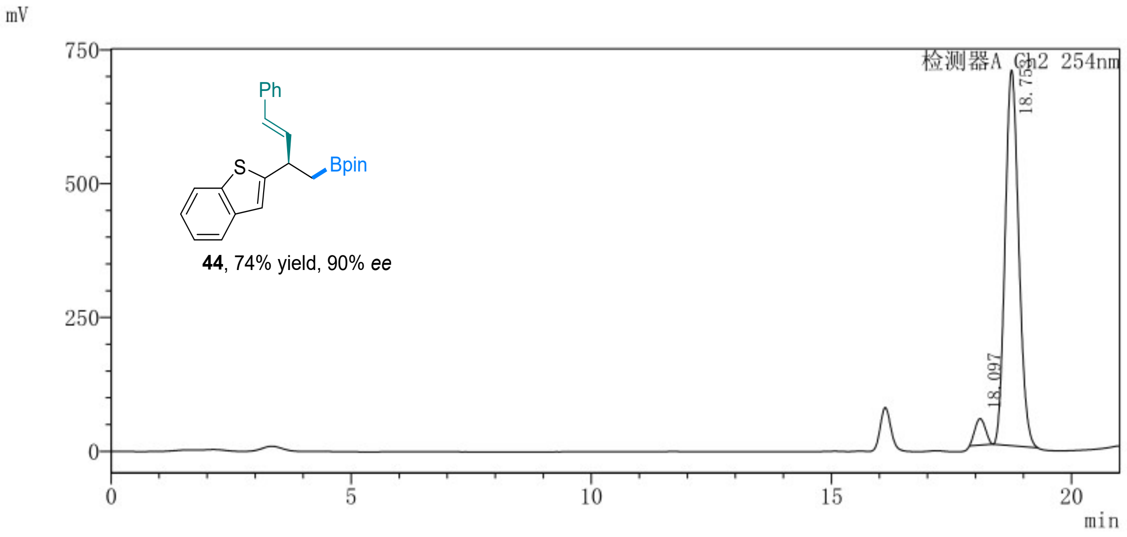

PDA Ch2 254nm

Peak#	Resolution Time	Area	Height	Area %	Height %
1	18.097	750442	49364	5.145	6.573
2	18.753	13835170	701638	94.855	93.427
Total		14585611	751002	100.000	100.000

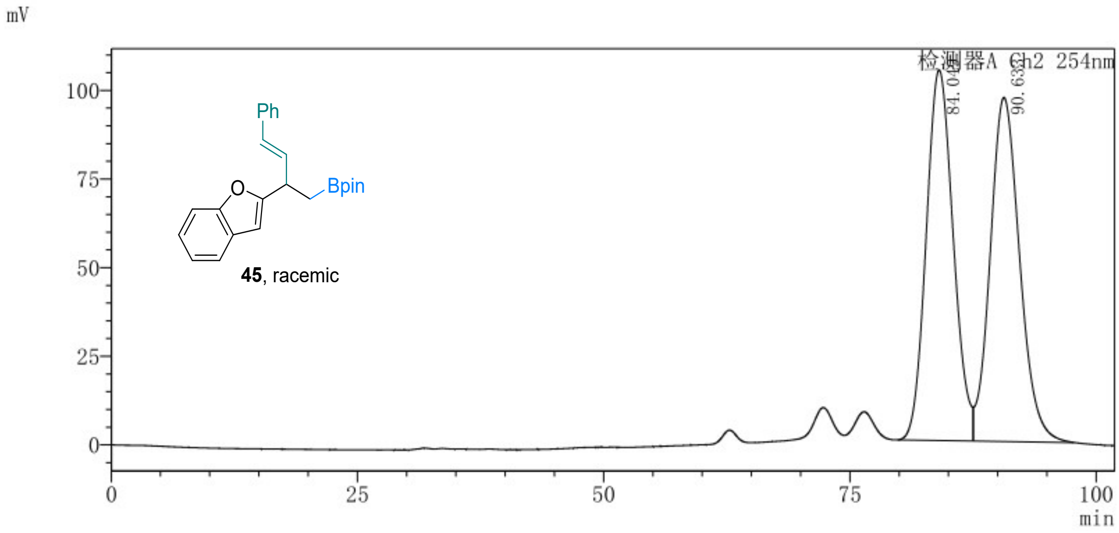

45, racemic

PDA Ch2 254nm

Peak#	Resolution Time	Area	Height	Area %	Height %
1	84.049	19929774	104556	49.633	51.859
2	90.633	20224398	97062	50.367	48.141
Total		40154172	201618	100.000	100.000

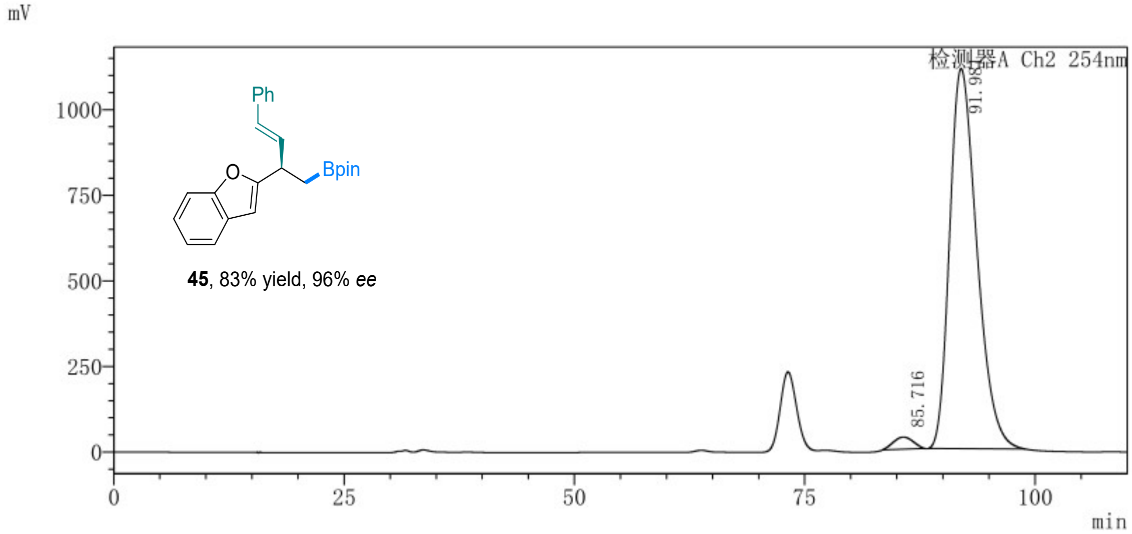

45, 83% yield, 96% *ee*

PDA Ch2 254nm

Peak#	Resolution Time	Area	Height	Area %	Height %
1	85.716	5162532	34959	2.210	3.052
2	91.981	228388023	1110366	97.790	96.948
Total		233550556	1145324	100.000	100.000

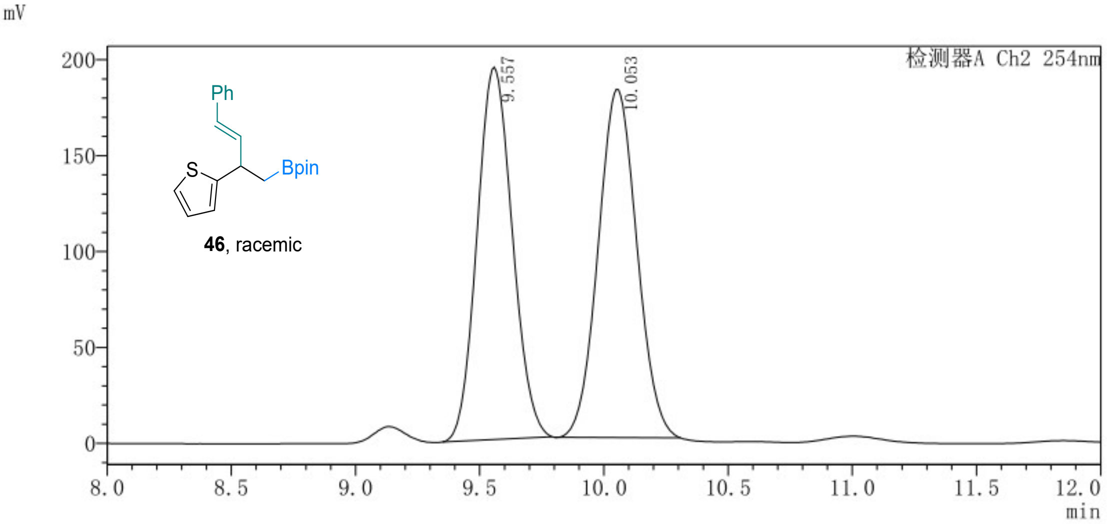

PDA Ch2 254nm

Peak#	Resolution Time	Area	Height	Area %	Height %
1	9.557	1912611	194073	49.315	51.659
2	10.053	1965767	181611	50.685	48.341
Total		3878378	375685	100.000	100.000

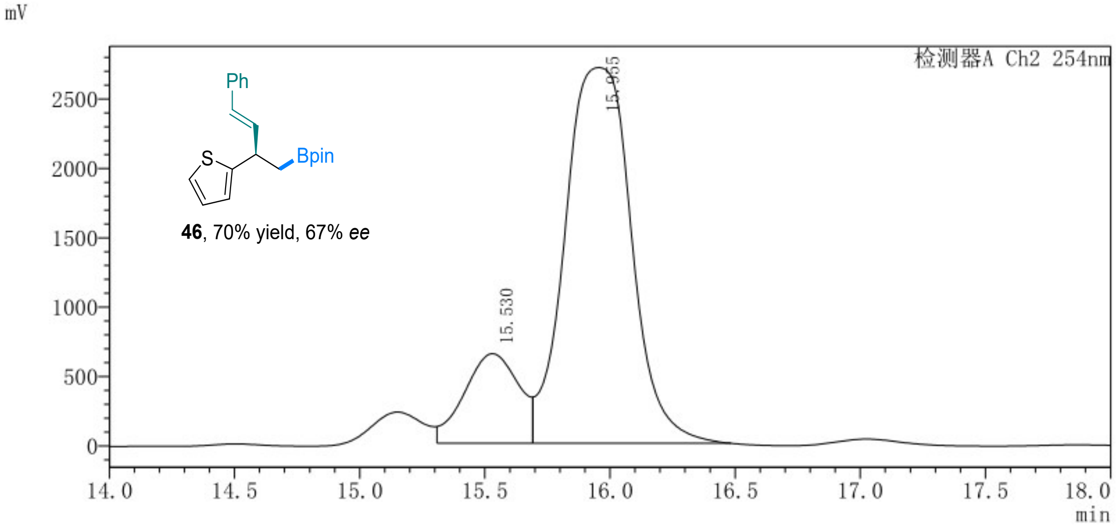

PDA Ch2 254nm

Peak#	Resolution Time	Area	Height	Area %	Height %
1	15.530	9805433	645289	16.455	19.238
2	15.955	49782949	2708960	83.545	80.762
Total		59588382	3354249	100.000	100.000

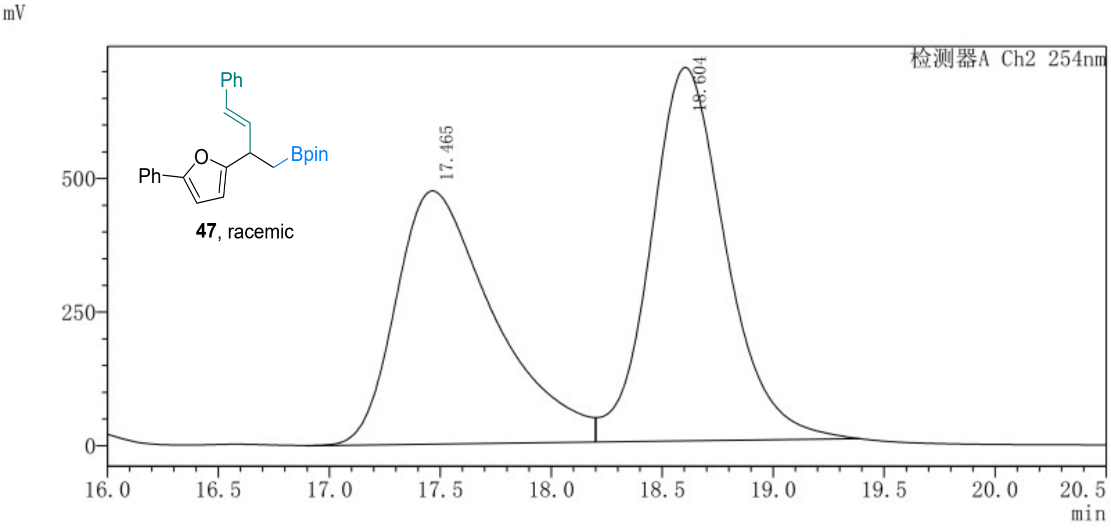

47, racemic

PDA Ch2 254nm

Peak#	Resolution Time	Area	Height	Area %	Height %
1	17.465	14630770	473784	47.034	40.417
2	18.604	16475957	698468	52.966	59.583
Total		31106727	1172253	100.000	100.000

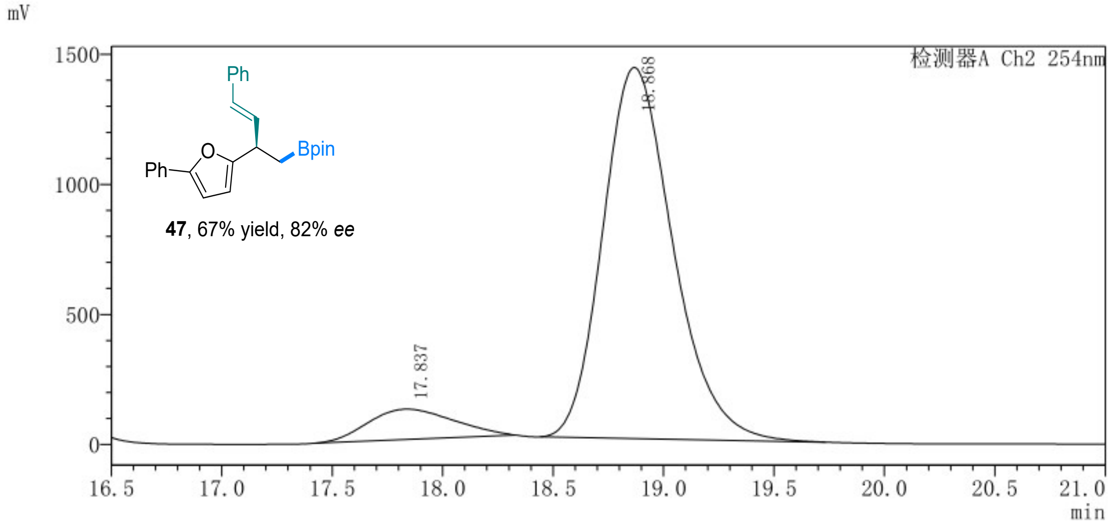

47, 67% yield, 82% *ee*

PDA Ch2 254nm

Peak#	Resolution Time	Area	Height	Area %	Height %
1	17.837	3133592	116303	9.028	7.536
2	18.868	31576897	1427003	90.972	92.464
Total		34710488	1543307	100.000	100.000

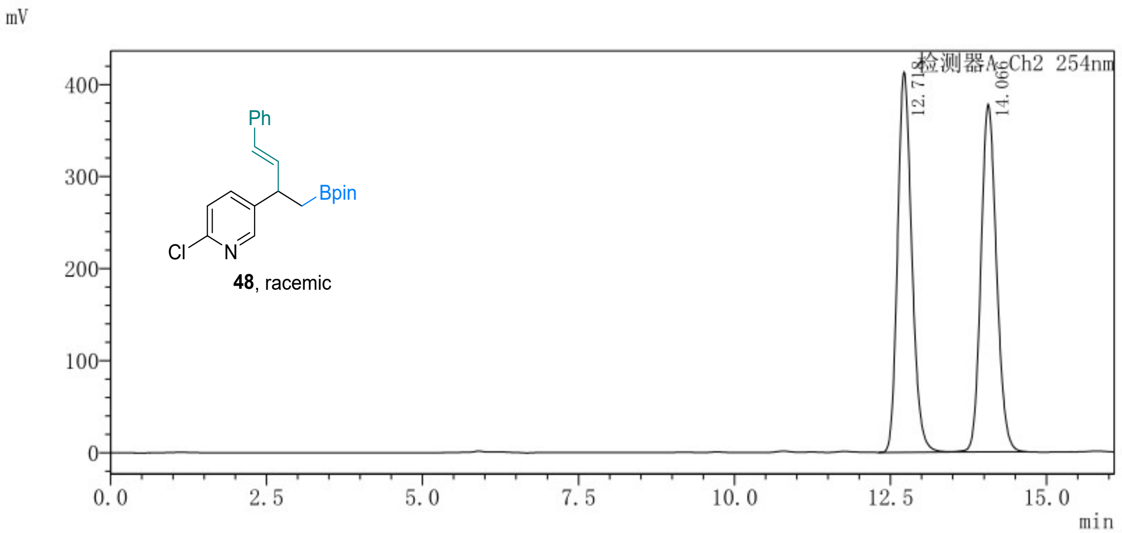

48, racemic

PDA Ch2 254nm

Peak#	Resolution Time	Area	Height	Area %	Height %
1	12.718	6545645	412855	49.939	52.227
2	14.066	6561749	377642	50.061	47.773
Total		13107394	790496	100.000	100.000

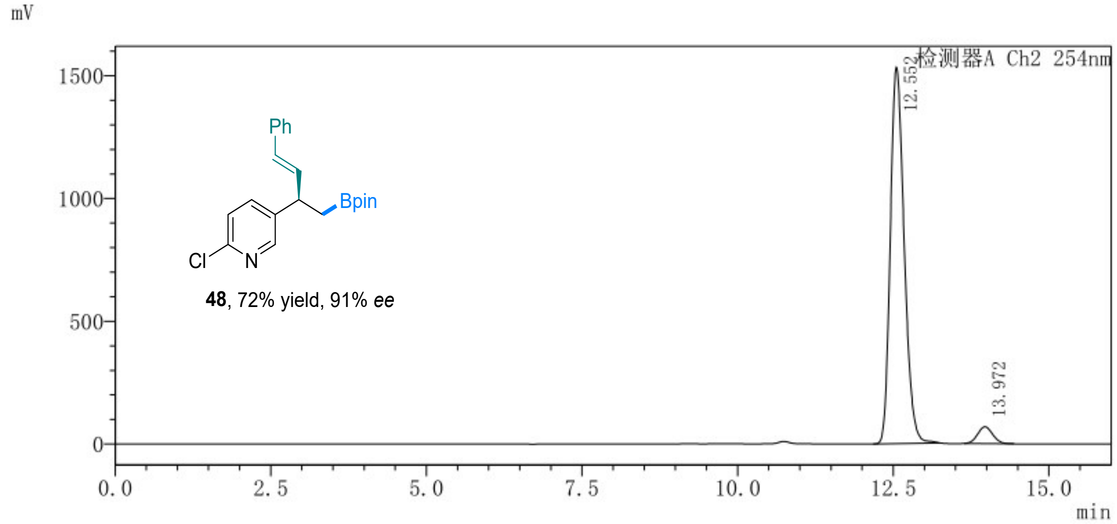

48, 72% yield, 91% *ee*

PDA Ch2 254nm

Peak#	Resolution Time	Area	Height	Area %	Height %
1	12.552	23760180	1532563	95.338	95.703
2	13.972	1161893	68809	4.662	4.297
Total		24922073	1601373	100.000	100.000

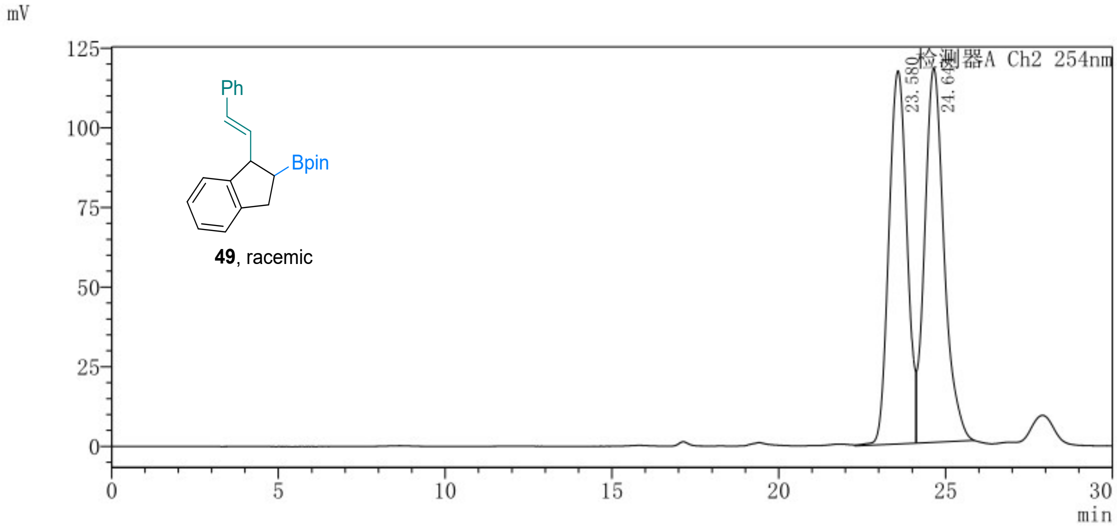

PDA Ch2 254nm

Peak#	Resolution Time	Area	Height	Area %	Height %
1	23.580	4401579	117128	47.994	49.927
2	24.644	4769482	117470	52.006	50.073
Total		9171061	234598	100.000	100.000

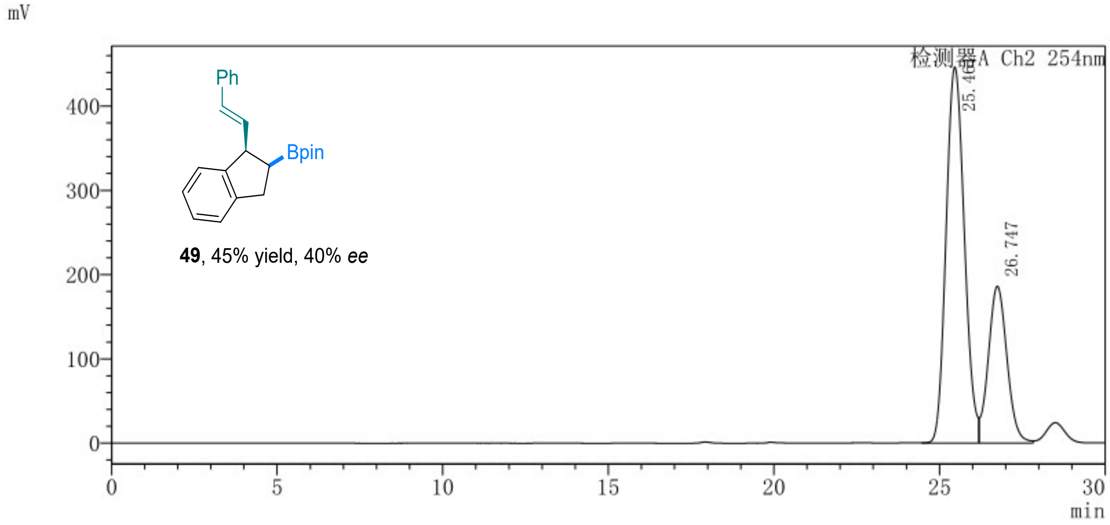

PDA Ch2 254nm

Peak#	Resolution Time	Area	Height	Area %	Height %
1	25.461	17141998	446385	70.084	70.551
2	26.747	7317134	186325	29.916	29.449
Total		24459132	632710	100.000	100.000

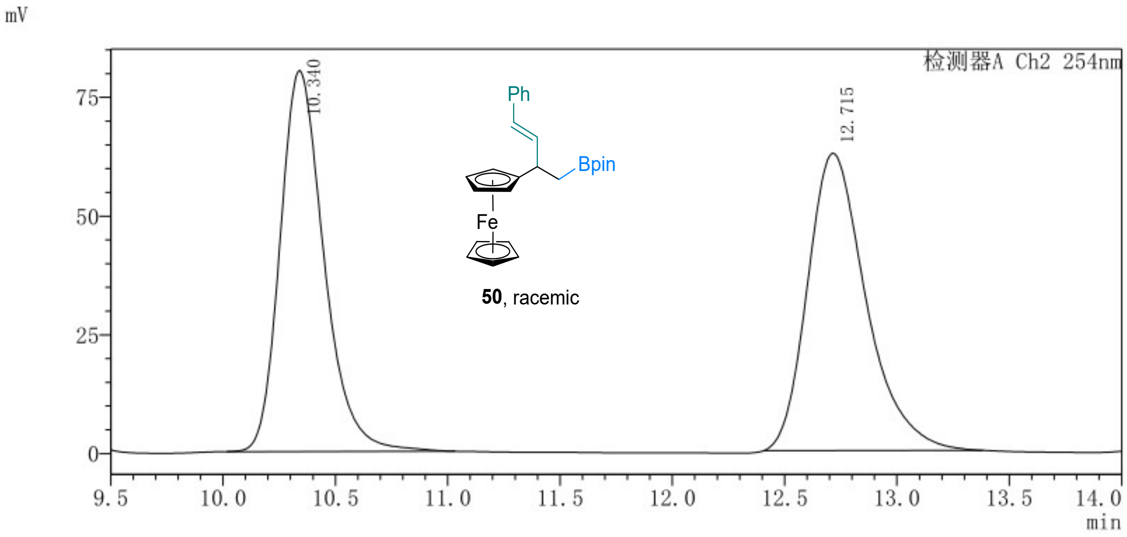

PDA Ch2 254nm

Peak#	Resolution Time	Area	Height	Area %	Height %
1	10.340	1090439	80239	49.002	56.168
2	12.715	1134859	62617	50.998	43.832
Total		2225297	142856	100.000	100.000

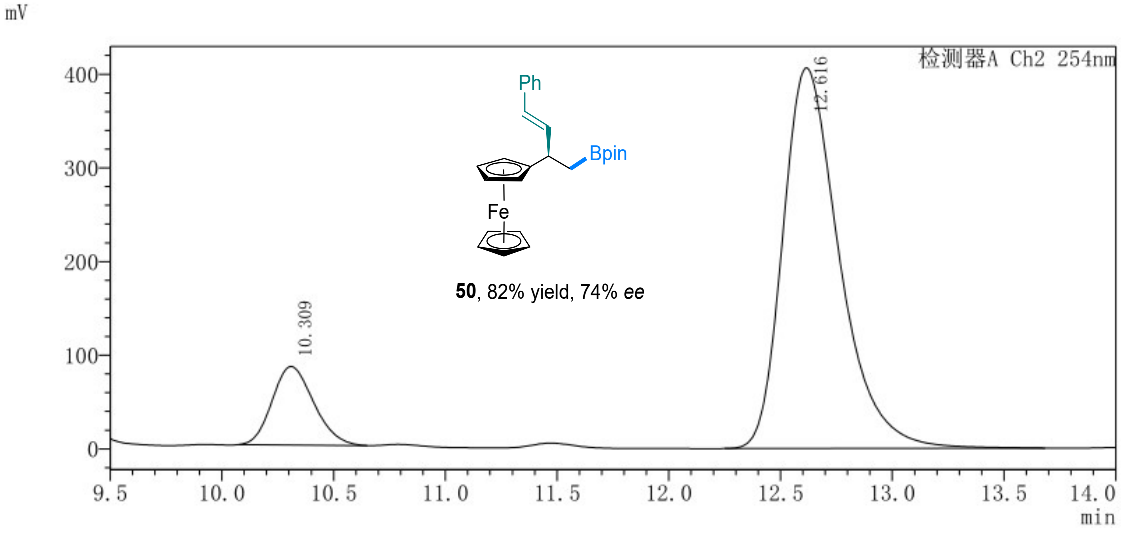

PDA Ch2 254nm

Peak#	Resolution Time	Area	Height	Area %	Height %
1	10.309	1085350	83925	13.158	17.116
2	12.616	7162985	406400	86.842	82.884
Total		8248335	490325	100.000	100.000

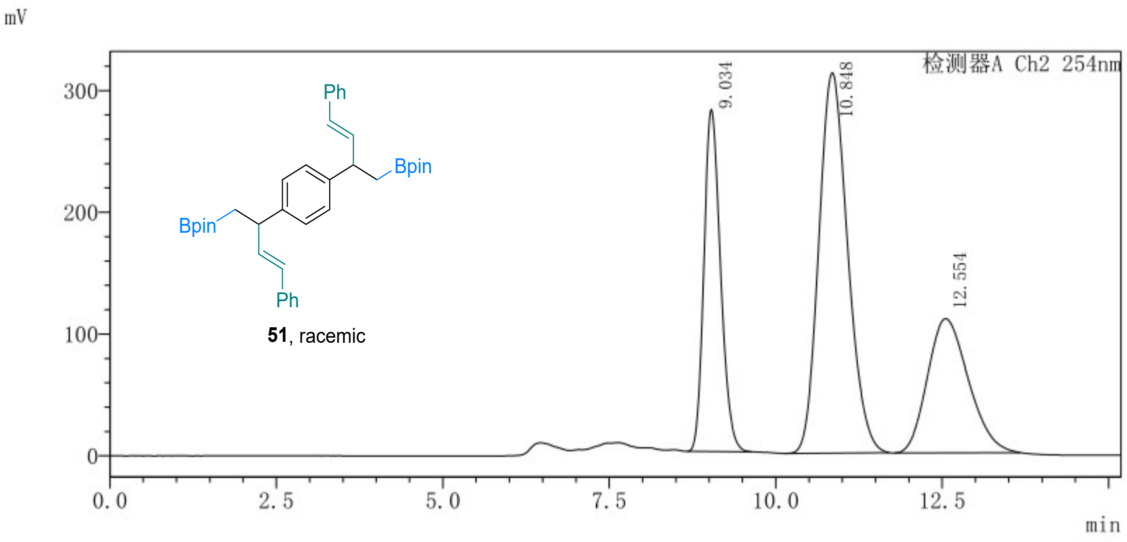

PDA Ch2 254nm

Peak#	Resolution Time	Area	Height	Area %	Height %
1	9.034	4880345	280877	25.665	39.931
2	10.848	9407677	312318	49.473	44.401
3	12.554	4727835	110211	24.863	15.668
Total		19015857	703406	100.000	100.000

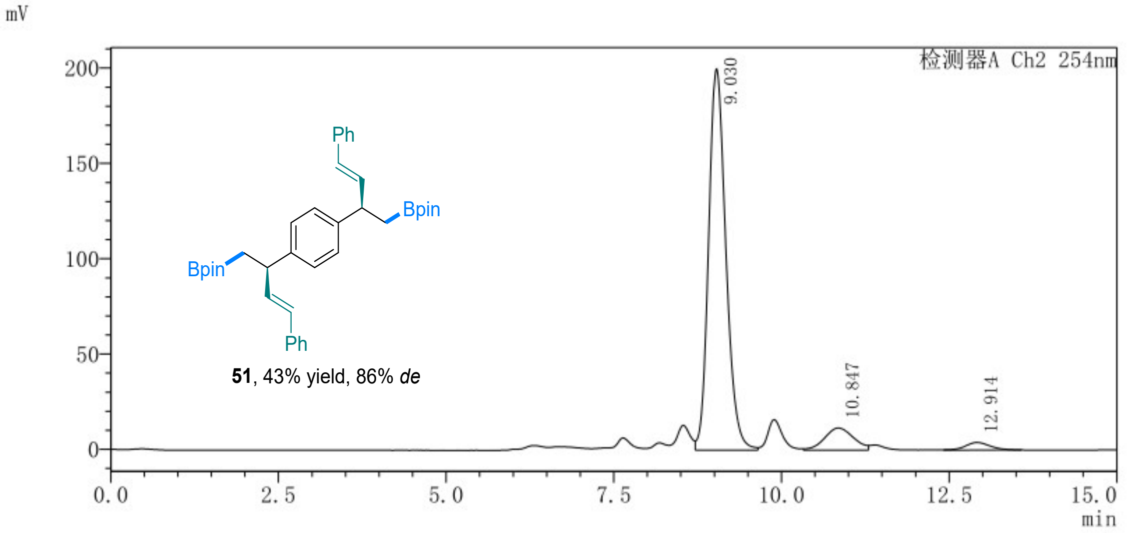

PDA Ch2 254nm

Peak#	Resolution Time	Area	Height	Area %	Height %
1	9.030	3542574	200037	88.544	92.738
2	10.847	353443	11651	8.834	5.402
3	12.914	104913	4012	2.622	1.860
Total		4000929	215701	100.000	100.000

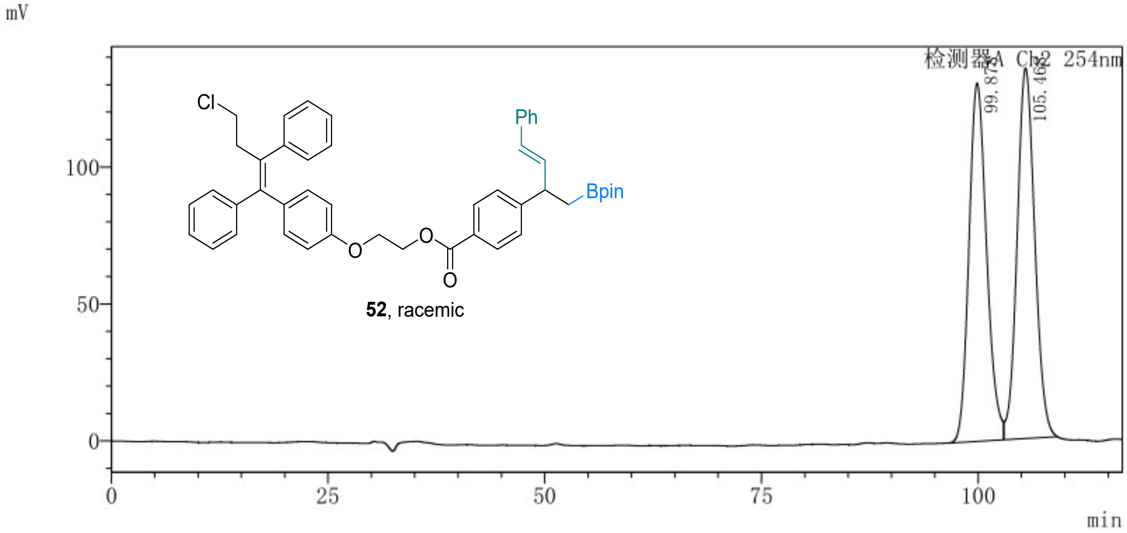

PDA Ch2 254nm

Peak#	Resolution Time	Area	Height	Area %	Height %
1	99. 878	18510859	130753	49. 243	49. 166
2	105. 468	19079676	135190	50. 757	50. 834
Total		37590535	265943	100. 000	100. 000

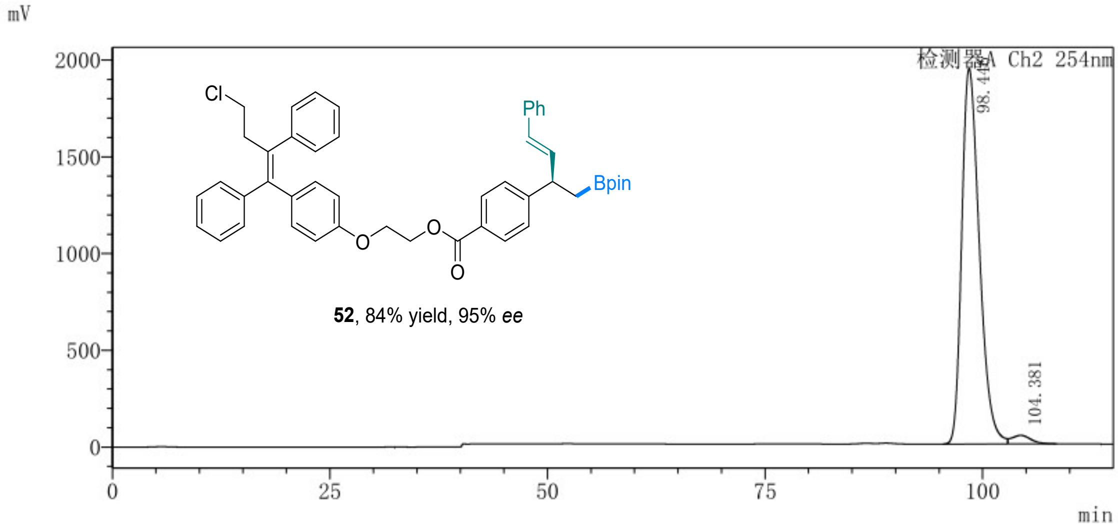

PDA Ch2 254nm

Peak#	Resolution Time	Area	Height	Area %	Height %
1	98. 446	273164657	1940641	97. 593	97. 775
2	104. 381	6736536	44168	2. 407	2. 225
Total		279901193	1984809	100. 000	100. 000

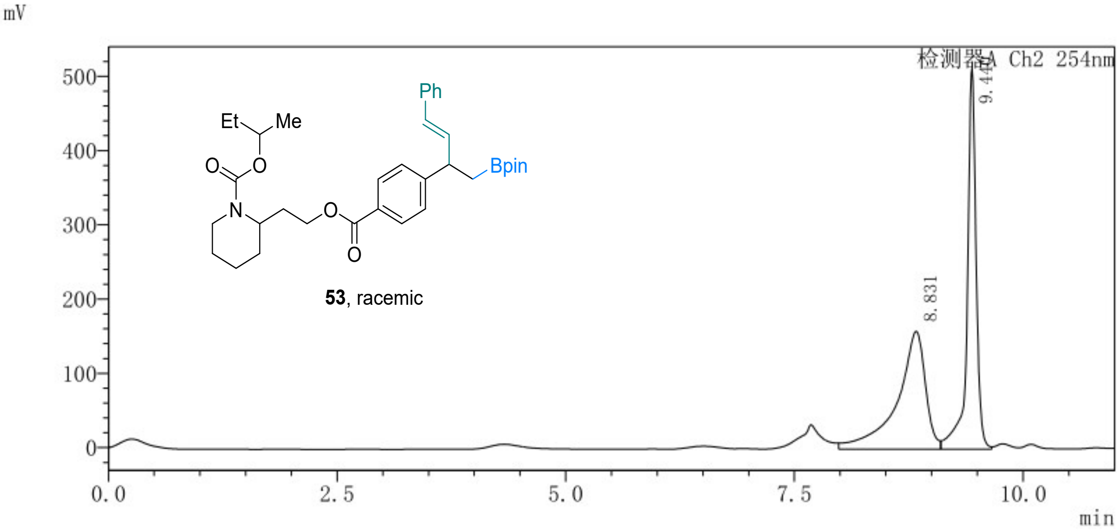

PDA Ch2 254nm

Peak#	Resolution Time	Area	Height	Area %	Height %
1	8.831	3432681	158890	49.579	23.637
2	9.440	3490993	513324	50.421	76.363
Total		6923674	672213	100.000	100.000

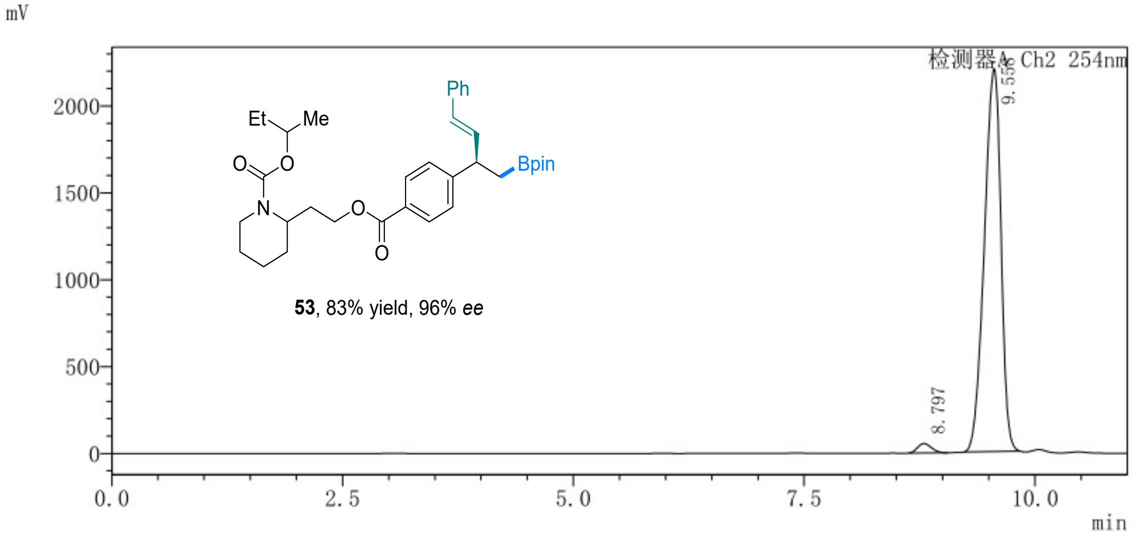

PDA Ch2 254nm

Peak#	Resolution Time	Area	Height	Area %	Height %
1	8.797	564971	54294	2.007	2.405
2	9.558	27586156	2203665	97.993	97.595
Total		28151127	2257958	100.000	100.000

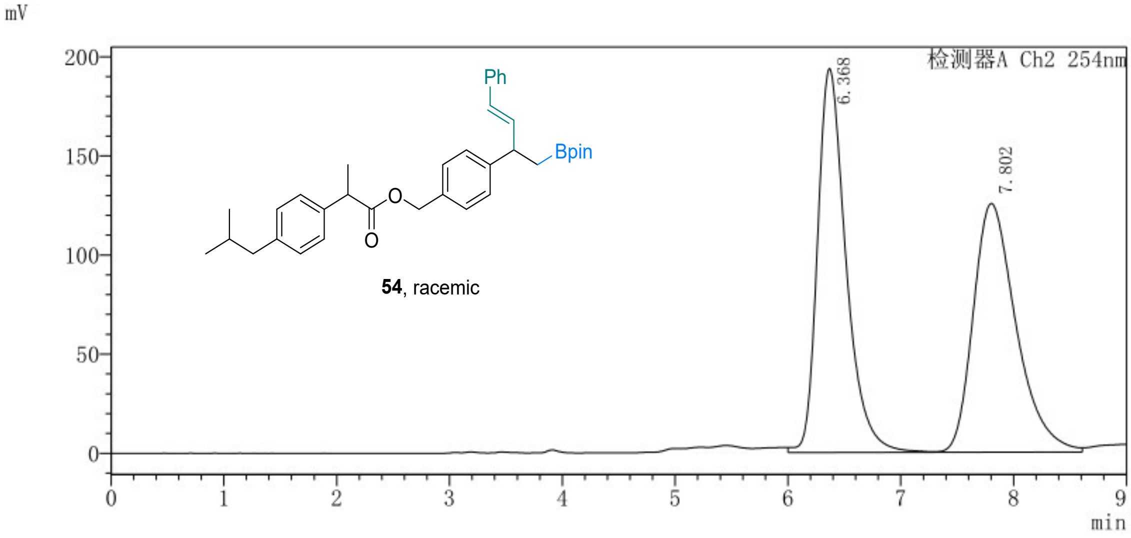

PDA Ch2 254nm

Peak#	Resolution Time	Area	Height	Area %	Height %
1	6. 368	3406116	193672	50. 715	60. 694
2	7. 802	3310140	125424	49. 285	39. 306
Total		6716256	319096	100. 000	100. 000

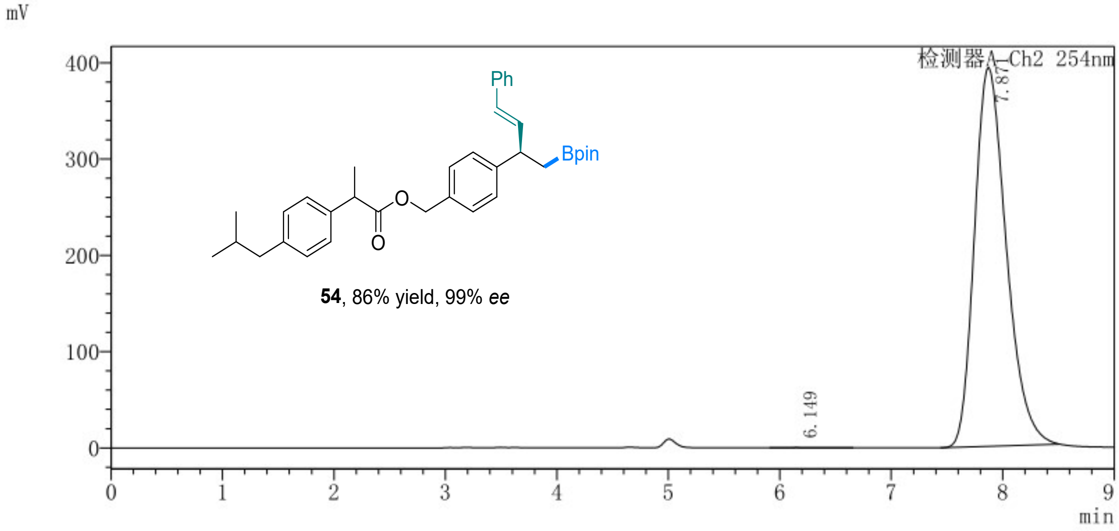

PDA Ch2 254nm

Peak#	Resolution Time	Area	Height	Area %	Height %
1	6. 149	1996	180	0. 026	0. 046
2	7. 871	7802542	393148	99. 974	99. 954
Total		7804538	393328	100. 000	100. 000

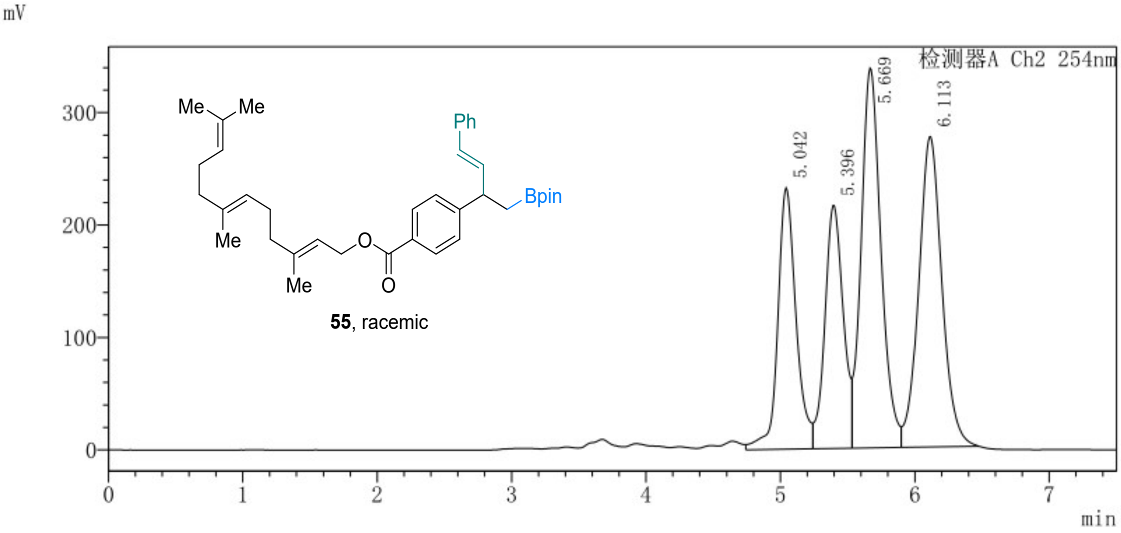

PDA Ch2 254nm

Peak#	Resolution Time	Area	Height	Area %	Height %
1	5.042	2200902	232463	19.951	21.883
2	5.396	2066265	216161	18.730	20.348
3	5.669	3460588	337749	31.369	31.794
4	6.113	3303981	275941	29.950	25.975
Total		11031737	1062313	100.000	100.000

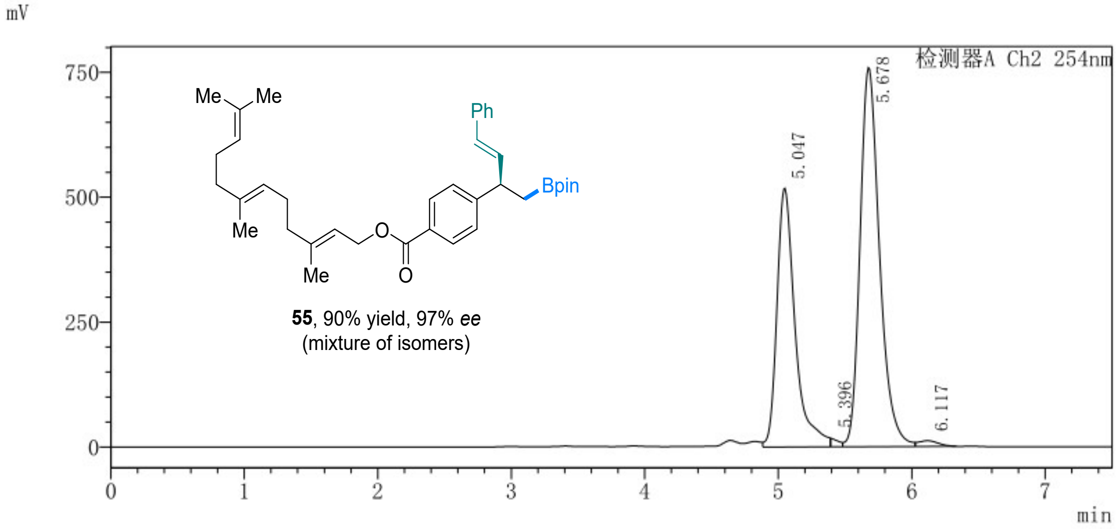

PDA Ch2 254nm

Peak#	Resolution Time	Area	Height	Area %	Height %
1	5.047	4896803	517640	38.271	39.664
2	5.396	66275	17290	0.518	1.325
3	5.678	7714460	758772	60.292	58.140
4	6.117	117687	11372	0.920	0.871
Total		12795225	1305074	100.000	100.000

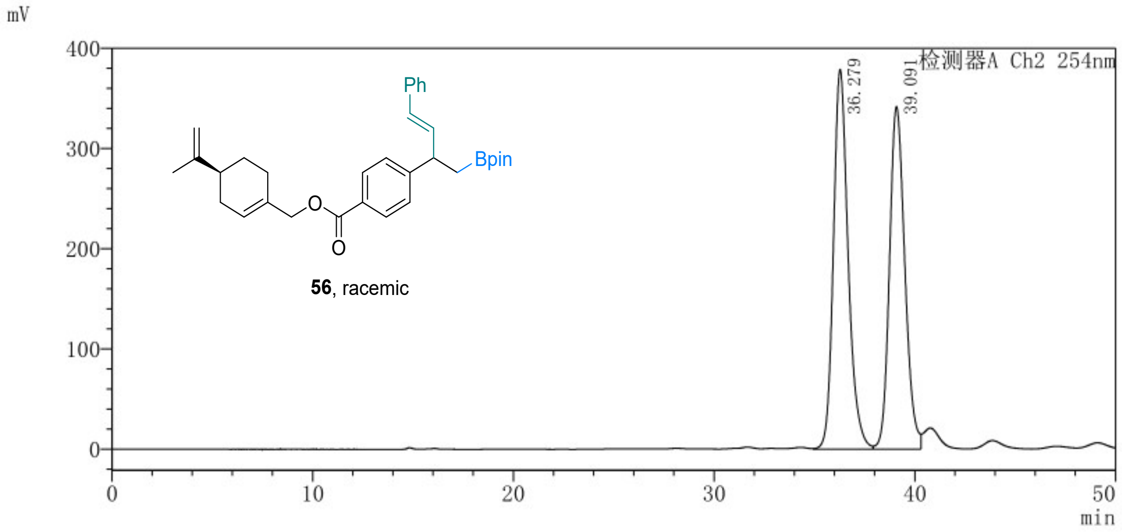

PDA Ch2 254nm

Peak#	Resolution Time	Area	Height	Area %	Height %
1	36.279	19291226	378589	51.428	52.564
2	39.091	18219930	341661	48.572	47.436
Total		37511156	720250	100.000	100.000

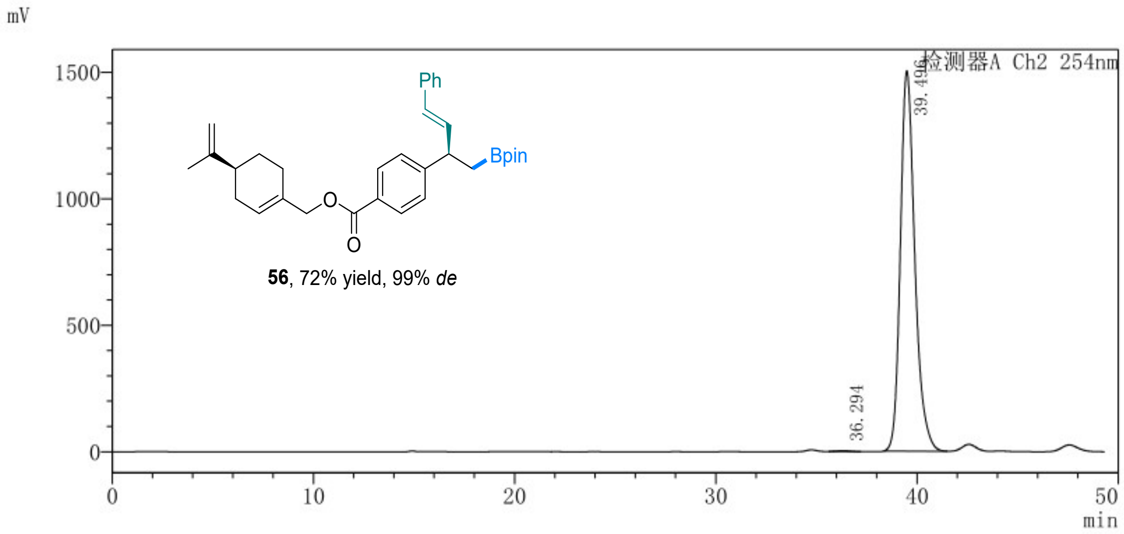

PDA Ch2 254nm

Peak#	Resolution Time	Area	Height	Area %	Height %
1	36.294	163410	3092	0.217	0.205
2	39.496	75015870	1504266	99.783	99.795
Total		75179281	1507357	100.000	100.000

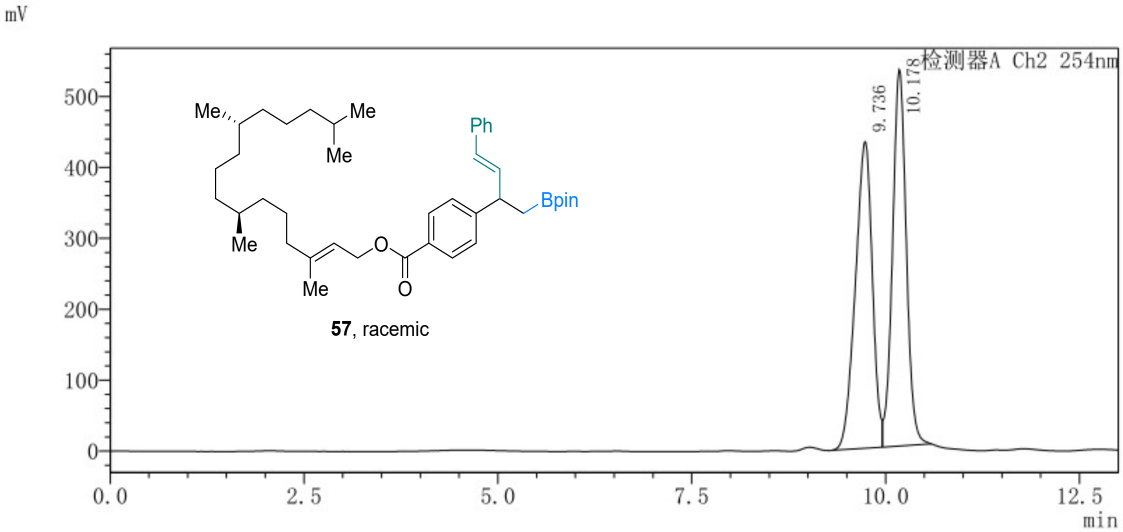

PDA Ch2 254nm

Peak#	Resolution Time	Area	Height	Area %	Height %
1	9. 736	6588725	432322	50. 166	44. 886
2	10. 178	6545101	530824	49. 834	55. 114
Total		13133826	963147	100. 000	100. 000

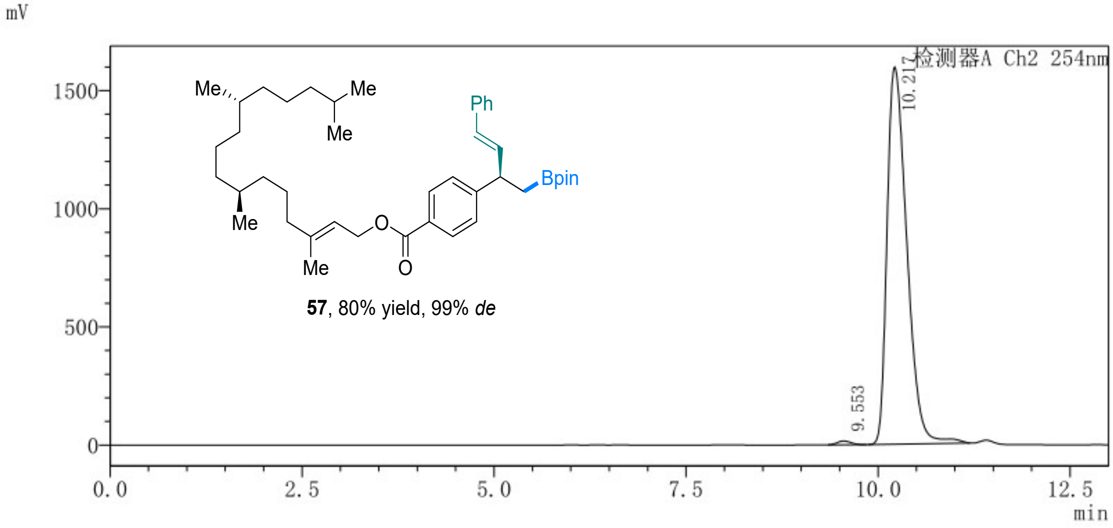

PDA Ch2 254nm

Peak#	Resolution Time	Area	Height	Area %	Height %
1	9. 553	203925	16262	0. 709	1. 009
2	10. 217	28565226	1595742	99. 291	98. 991
Total		28769151	1612004	100. 000	100. 000

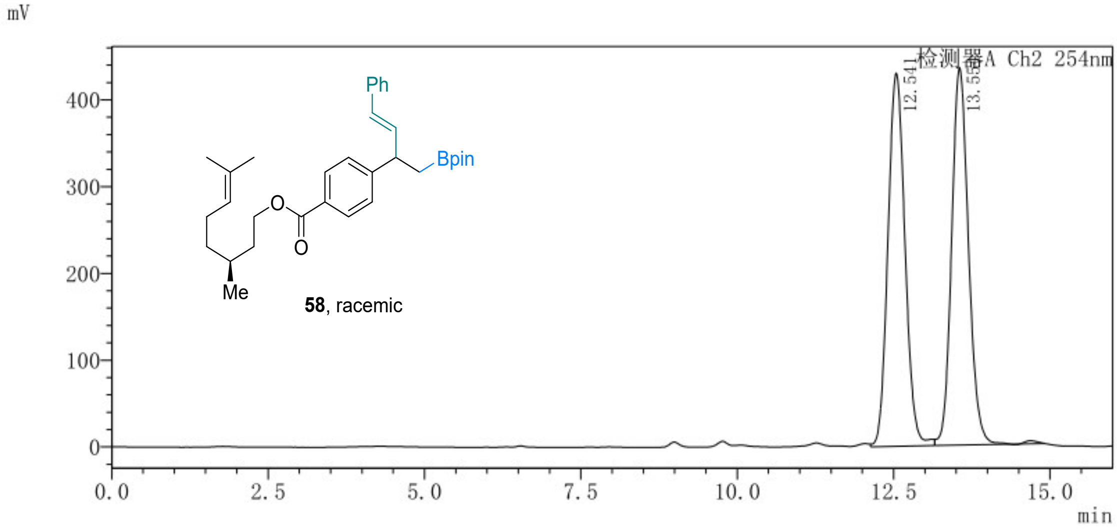

PDA Ch2 254nm

Peak#	Resolution Time	Area	Height	Area %	Height %
1	12. 541	8171282	430061	49. 968	49. 717
2	13. 554	8181871	434964	50. 032	50. 283
Total		16353153	865025	100. 000	100. 000

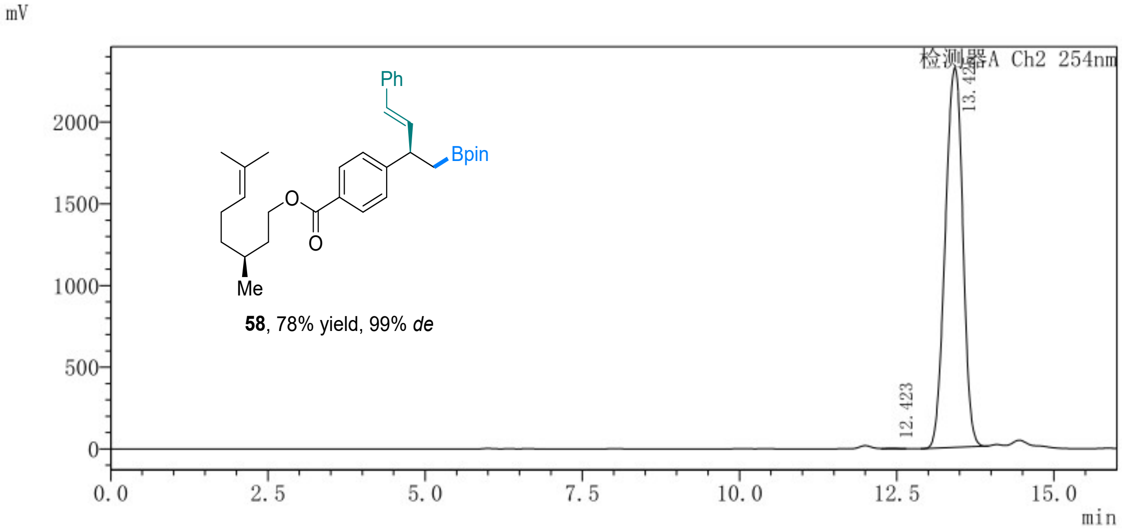

PDA Ch2 254nm

Peak#	Resolution Time	Area	Height	Area %	Height %
1	12. 423	12574	1067	0. 028	0. 046
2	13. 425	45142420	2322544	99. 972	99. 954
Total		45154995	2323611	100. 000	100. 000

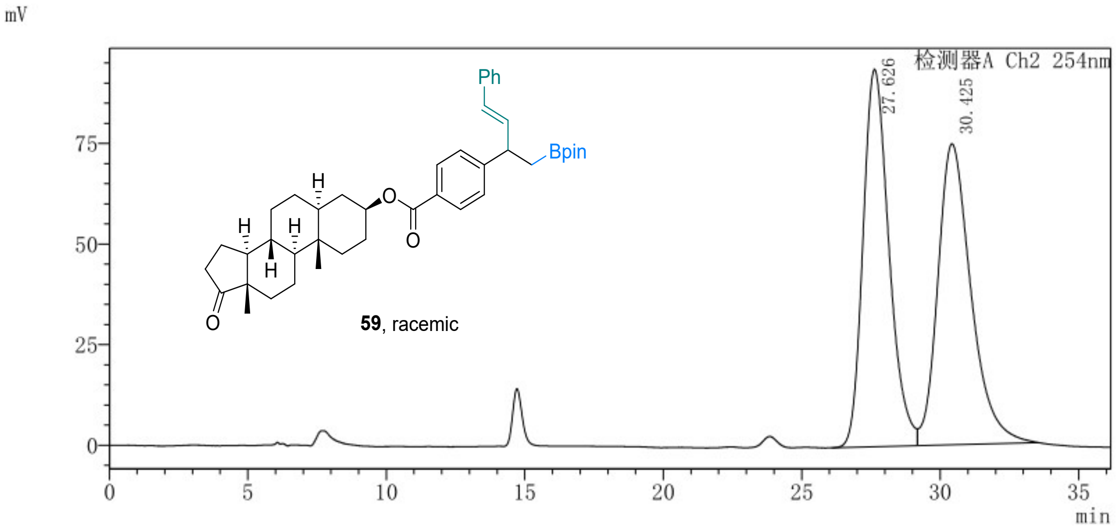

PDA Ch2 254nm

Peak#	Resolution Time	Area	Height	Area %	Height %
1	27.626	6117051	93770	49.509	55.622
2	30.425	6238442	74815	50.491	44.378
Total		12355493	168585	100.000	100.000

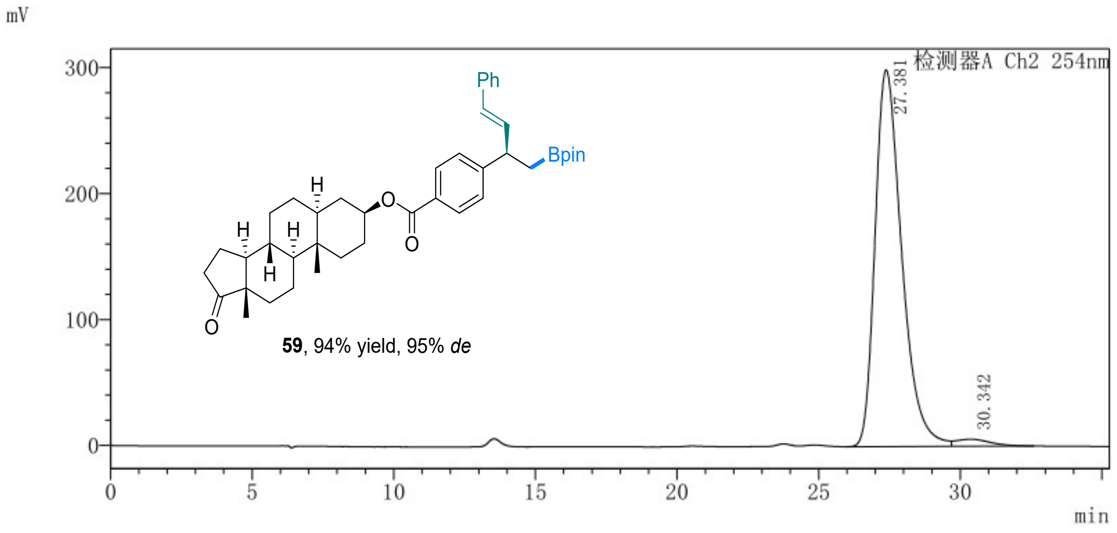

PDA Ch2 254nm

Peak#	Resolution Time	Area	Height	Area %	Height %
1	27.381	19570605	299029	97.653	98.172
2	30.342	470357	5569	2.347	1.828
Total		20040962	304598	100.000	100.000

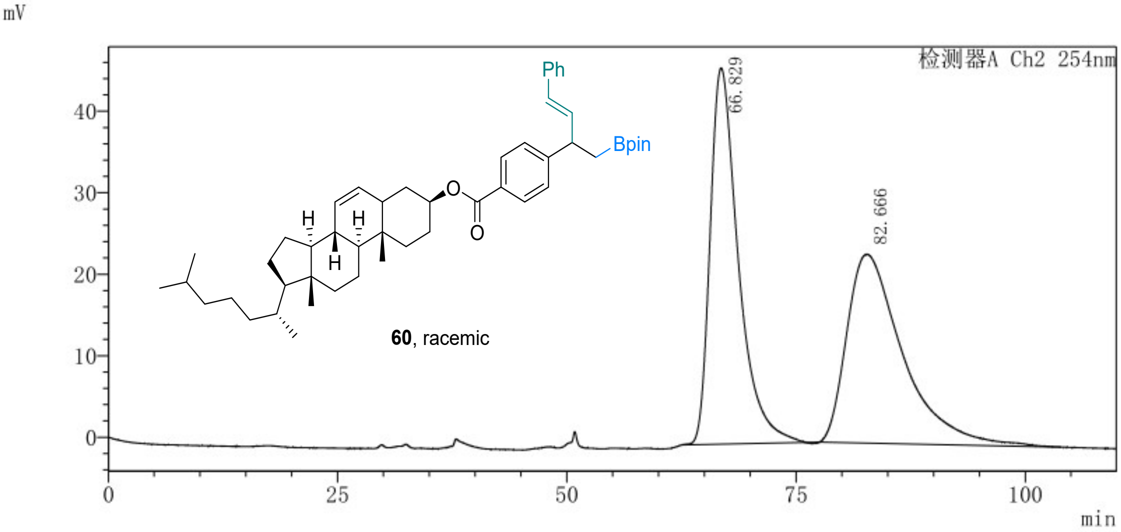

PDA Ch2 254nm

Peak#	Resolution Time	Area	Height	Area %	Height %
1	66. 829	9852206	46125	50. 061	66. 566
2	82. 666	9828171	23167	49. 939	33. 434
Total		19680377	69292	100. 000	100. 000

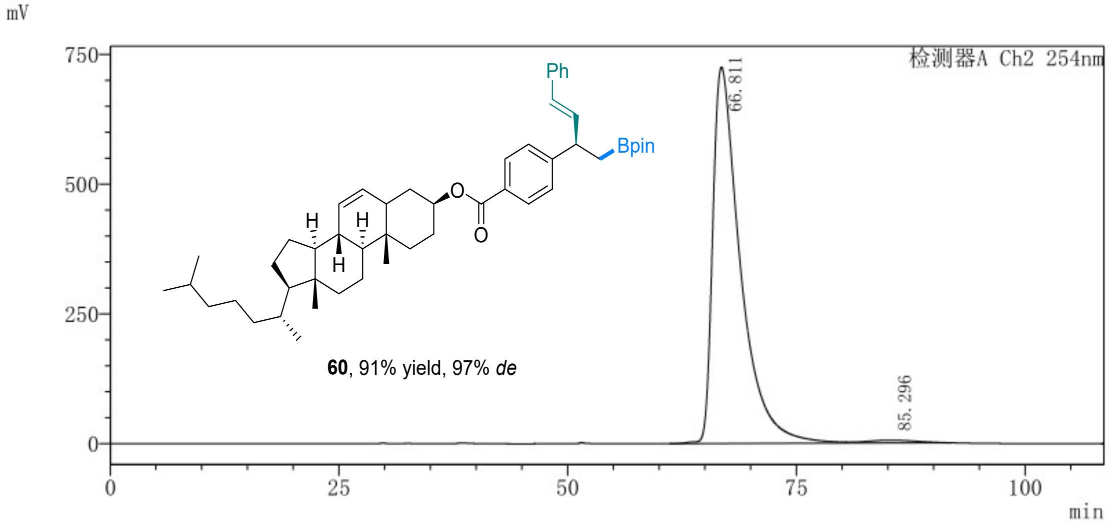

PDA Ch2 254nm

Peak#	Resolution Time	Area	Height	Area %	Height %
1	66. 811	152280586	725036	98. 600	99. 262
2	85. 296	2161922	5389	1. 400	0. 738
Total		154442509	730425	100. 000	100. 000
